# Ligand Relay Catalysis Enables Asymmetric Migratory Hydroarylation for the Concise Synthesis of Chiral α‐(Hetero)Aryl‐Substituted Amines

**DOI:** 10.1002/advs.202306447

**Published:** 2024-02-28

**Authors:** Junqian Zhou, Yuli He, Zihao Liu, You Wang, Shaolin Zhu

**Affiliations:** ^1^ State Key Laboratory of Coordination Chemistry Jiangsu Key Laboratory of Advanced Organic Materials Chemistry and Biomedicine Innovation Center (ChemBIC) School of Chemistry and Chemical Engineering Nanjing University Nanjing 210093 P. R. China; ^2^ School of Chemistry and Chemical Engineering Henan Normal University Xinxiang 453007 P. R. China; ^3^ Shanghai Key Laboratory for Molecular Engineering of Chiral Drugs Shanghai Jiao Tong University Shanghai 200240 P. R. China

**Keywords:** alkenes, arylation, asymmetric catalysis, ligand relay catalysis, nickel

## Abstract

Complementary to the design of a single structurally complex chiral ligand to promote each step in transition‐metal catalysis, multiligand relay catalysis through dynamic ligand exchange with each step in the catalytic cycle promoted by its best ligand provides an attractive approach to enhance the whole reaction reactivity and selectivity. Herein, a regio‐ and enantioselective NiH‐catalyzed migratory hydroarylation process with a simple combination of a chain‐walking ligand and an asymmetric arylation ligand, producing high‐value chiral *α*‐(hetero)aryl‐substituted amines and their derivatives under mild conditions, is reported. The potential synthetic applications of this transformation are demonstrated by the concise synthesis of (*S*)‐nicotine and a CDK8 inhibitor.

## Graphical abstract

Enabled by a ligand relay catalytic strategy, a regio‐ and enantioselective migratory hydroarylation of alkene‐contained cyclic or acyclic amines with (hetero)aryl bromides is reported. A wide variety of chiral *α*‐(hetero)aryl‐substituted, both cyclic and acyclic amines, and their derivatives are obtained in high yields with well control of both rr and *ee* under mild conditions.

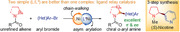

## 1 Introduction

**Figure 1:**
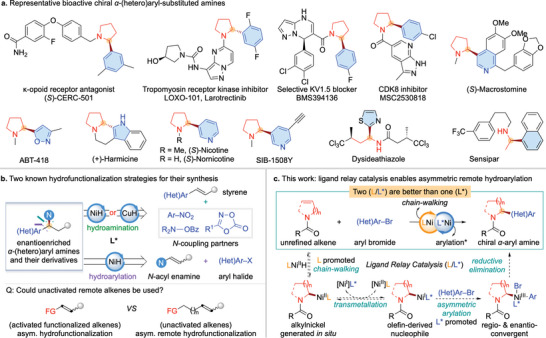
Representative chiral *α*‐(hetero)aryl‐substituted amines and asymmetric migratory hydroarylation strategy for their preparation.

As a structural motif, enantioenriched *α*‐(hetero)aryl‐substituted amines and their derivatives widely exist in various natural products, pharmaceuticals, and catalysts (**Figure** **1**a).^[^ ^1^ ^]^ Starting from activated functionalized alkene starting materials, asymmetric hydroamination^[^ ^2^, ^3^ ^]^ and hydroarylation^[^ ^4^ ^]^ catalyzed by metal‐hydride, especially by nickel‐hydride or copper‐hydride, are two attractive approaches for the efficient synthesis of *α*‐(hetero)aryl‐substituted amines and their derivatives^[^ ^5^ ^]^ (Figure 1b). In these two processes, with a stoichiometric amount of hydrosilane, styrenes^[^ ^2^, ^3^ ^]^ or protected (acyl or carbamate) enamines^[^ ^4^ ^]^ are used as latent alkyl carbanion equivalents through the catalytic generation of alkylcopper or alkylnickel intermediates, avoiding the pre‐generation of organometallic reagents. However, in terms of olefin starting materials, the need to prepare activated functionalized alkenes in asymmetric hydrofunctionalization reactions^[^ ^6^, ^7^ ^]^ is still less than ideal. It would be ideal if the unactivated alkenes which generally are commercially available or more easily prepared. We recognized that if asymmetric migratory hydrofunctionalization could be achieved,^[^ ^8^, ^9^, ^10^, ^11^ ^]^ unactivated remote alkenes or isomeric mixtures of alkenes would be suitable starting materials, generating the required alkylnickel species in situ through chain‐walking to participate in the subsequent asymmetric functionalization.

However, in NiH‐catalyzed asymmetric remote functionalization, the design of a single chiral ligand to promote both chain‐walking and subsequent asymmetric coupling is highly challenging.^[^ ^11^ ^]^ Recently, a synergistic combination of two structurally simple ligands, one for chain‐walking and the other for asymmetric coupling, ligand relay catalysis^[^ ^12^ ^]^ through dynamic ligand exchange provides a particularly appealing approach to address this regio‐ and stereochemical challenge in this area. We hypothesized that asymmetric migratory hydroarylation^[^ ^10^, ^13^ ^]^ might analogously be realized using our ligand relay catalytic strategy, thereby allowing access to chiral *α*‐(hetero)aryl‐substituted amines from unactivated remote alkenes or their isomeric mixtures (Figure 1c). Specifically, with a suitable chain‐walking ligand (L) to generate a series of alkylnickel species along an alkyl chain, the subsequent regio‐ and enantioselective arylation could be realized efficiently and selectively with an appropriate asymmetric arylation ligand (L*). A suitable ligand combination must fulfill a number of requirements. First, chain‐walking ligand‐promoted isomerization between alkylnickel isomers must be rapid compared to subsequent arylation. Second, to avoid the racemic background reaction in the arylation step, the chain‐walking ligand or ligandless background reactivity must be relatively low. Third, asymmetric arylation promoted by the chiral ligand must be highly regio‐ and enantioselective.

In this work, we describe a high regio‐ and enantioselective Ni‐catalyzed hydroarylation process enabled by a ligand relay catalysis strategy under exceptionally mild conditions. A wide variety of enantioenriched *α*‐(hetero)aryl‐substituted amines and their derivatives were obtained in high yields with well control of both rr and *ee*. The utility of this protocol is illustrated by the concise three‐step synthesis of (*S*)‐nicotine and a CDK8 inhibitor.

## 2 Results and Discussion

To test our hypothesis, the asymmetric migratory hydroarylation reaction of *N*‐(but‐3‐en‐1‐yl)benzamide (**1a**) and methyl 4‐bromobenzoate (**2a**) was chosen as the model reaction under a combination of a chain‐walking ligand and an asymmetric arylation ligand (**Table** **1**). Systematic optimization revealed that both the regioselectivity and enantioselectivity could be well‐controlled with a combination of a chain‐walking phenanthroline ligand (**L1**) and an asymmetric arylation biimidazoline (BIIM) ligand (L1*), delivering product **3a** in 72% isolated yield with a 97:3 regioisomeric ratio (*rr*) and 92% enantiomeric excess (*ee*) (**Table** **1**, entry 1). In line with our expectations, both ligands are necessary for this migratory asymmetric arylation, and essentially no desired product formation was observed when either ligand was omitted (entries 2 and 3). Decreasing the loading of chain‐walking ligand to 0.3 mol% led to somewhat lower regioselectivity (entry 4). An alternative chain‐walking ligand (**L2**) was found to be considerably less effective than **L1** (entry 5). On the other hand, a chiral BIIM (bisimidazoline) ligand (L1*) turned out to be generally more effective on both reactivity and enantioselectivity than that with a BiOx (bi‐oxazoline) scaffold (entry 6). Reducing the loading of nickel salt to 0.5 mol% (same amount of **L1**) led to low conversion (entry 7). This preliminary result indicates that the required chiral ligand (L1*) ligated *N*‐protected *α*‐amino alkylnickel(I) species is mainly obtained through reversible transmetallation between alkylnickel(II)**L1** species and nickel(I)L1* species (L1*Ni^I^I or L1*Ni^I^H).^[^ ^14^ ^]^ The use of a different nickel source (NiBr_2_∙DME) or a silane source (diethoxymethylsilane) led to a slightly diminished yield (entries 8, 9). Interestingly, by changing the cation of a base from sodium to potassium (K_2_CO_3_ or K_3_PO_4_), the **L1**‐promoted background arylation reaction could be enhanced, resulting in only moderate *ee* (entries 10 and 11). The addition of a catalytic amount of iodide salt could facilitate the chain‐walking process, resulting in better yield and rr (entries 1, 12 vs entry 13). Screening of solvents revealed that a single solvent led to either complete failure of the reaction (entry 14) or a diminished yield (entry 15).^[^ ^15^ ^]^ Notably, a slightly lower yield was observed when using the more reactive aryl iodide as an electrophile (entry 16).

**Table 1:** Variation of reaction parameters.

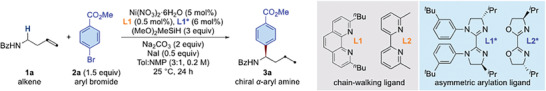				
Entry	Variation	Yield [%]^a)^	rr^b)^	*ee* [%]^c)^
1	None	73 (72)	97:3	92
2	w/o **L1**	0	–	–
3	w/o L1*	0	–	–
4	**L1** reduced to 0.3 mol%	69	92:8	96
5	**L2** instead of **L1**	12	>99:1	89
6	L2* instead of L1*	13	>99:1	–36
7	0.5 mol% Ni(NO_3_)_2_∙6H_2_O used	7	77:23	92
8	NiBr_2_·DME used	69	>99:1	86
9	(EtO)_2_MeSiH used	69	97:3	92
10	K_2_CO_3_ instead of Na_2_CO_3_	70	>99:1	60
11	K_3_PO_4_ instead of Na_2_CO_3_	28	86:14	56
12	TBAI instead of NaI	67	91:9	94
13	w/o NaI	14	60:40	95
14	Tol only	0	–	–
15	NMP only	19	>99:1	87
16	ArI instead of ArBr	59	94:6	92

^a)^ Yields determined by GC using *n*‐dodecane as the internal standard, the yield in parentheses is the isolated yield (0.20 mmol scale)

^b)^ rr, regioisomeric ratio, represents the ratio of *α*‐arylation product (**3a**) to all other regioisomers, determined by GC and GC‐MS analysis

^c)^ Enantioselectivities were determined by chiral HPLC analysis. Bz, benzoyl; Tol, toluene; NMP, *N*‐methylpyrrolidone.

**Figure 2:**
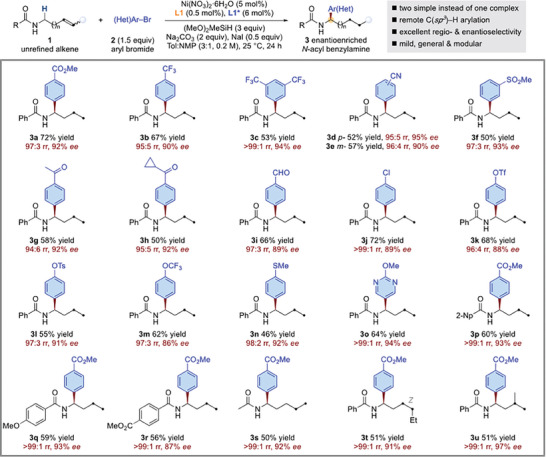
Asymmetric migratory hydroarylation to access enantioenriched acyclic *α*‐(hetero)aryl‐substituted amines. *^a^* Yield under each product refers to the isolated yield of purified product (0.20 mmol scale, average of two runs), regioisomeric ratio (*rr*) was determined by GC and GC‐MS analysis, enantioselectivities were determined by chiral HPLC analysis. Please see Figures S1 and S2 for test tube and cap used.

Under the optimal reaction conditions, a wide variety of alkene‐contained acyclic amines and aryl electrophiles serve as suitable coupling partners, generally leading to asymmetric arylation at *α*‐amino C−H position (**Figure** **2**). With respect to the aryl electrophile, both electron‐withdrawing (**3a**–**3m**) and electron‐rich (**3n**) aryl bromides were tolerated. A variety of functional groups were well accommodated, including an ester (**3a**), trifluoromethyl groups (**3b**, **3c**), nitriles (**3d**, **3e**), and ethers (**3m**–**3o**). Under these exceptionally mild conditions, easily reduced sulfone (**3f**), ketones (**3g**, **3h**), and aldehyde (**3i**) were left intact. Notably, the reaction was orthogonal to aryl chlorides (**3j**) and aryl triflates (**3k**, **3l**), functional groups amenable for further cross‐coupling. It was also found that heteroaryl bromides such as those containing a pyrimidine (**3o**) are also competent electrophiles. Furthermore, the acyl substituent in the alkene‐tethered acyclic amine partners may vary from aryl (**3p**–**3r**) to alkyl (**3s**), and various substituents may be present (**3p**–**3r**). Not limited to mono‐substituted terminal alkenes, unactivated internal alkene (**3t**) and 1,1‐disubstituted terminal alkene (**3u**) were equally applicable.

**Figure 3:**
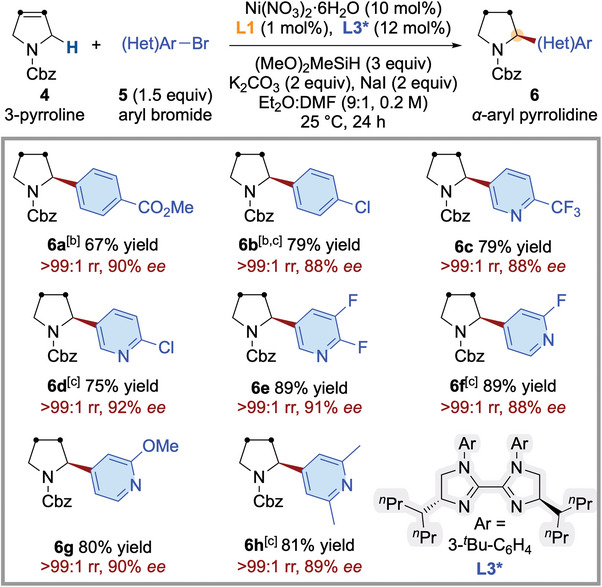
Asymmetric migratory hydroarylation to access enantioenriched *α*‐(hetero)aryl‐substituted *N*‐heterocycles. *^a^* Yield, rr, and ee are as defined in Figure 2. *^b^* 5 mol% cat, DME:DMA (9:1, 0.2 m). *^c^* (EtO)_2_MeSiH was used.

Not limited to alkene‐contained acyclic amines, our protocol was applicable across many commercially available *N*‐carbamate protected heterocyclic alkene substrates, affording corresponding biologically important enantioenriched *α*‐(hetero)aryl‐substituted *N*‐heterocycles as a single regioisomer in good yields with excellent *ee* (**Figure** **3**). Unfortunately, six‐membered *N*‐heterocyclic alkenes did not participate in the desired arylation event, with substantial amounts of the alkene starting material being recovered. In addition to aryl bromides (**6a**, **6b**), a wide variety of structurally diverse heteroaryl bromides (**6c**–**6h**) were also legitimate electrophiles. For example, pyridines with various substitution patterns which are commonly found in bioactive molecules, were shown to be viable substrates, delivering the chiral *α*‐heteroaryl‐substituted *N*‐heterocycles in good yields with excellent enantioselectivity (**6c**–**6h**).

**Scheme 1:**
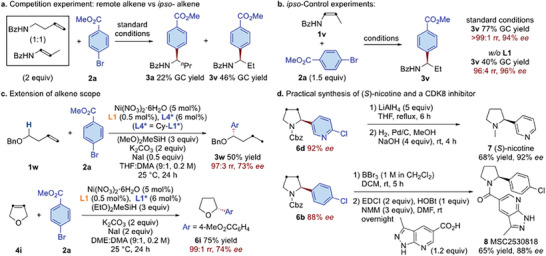
Control and competition experiment, extension of alkene scope, and synthetic application.

A competition experiment between a 1:1 ratio of *ipso*‐ and remote alkenes was carried out (**Scheme** **1**a). Both *ipso‐* and migratory arylation products (**3a** and **3v**) were obtained with similar selectivity. This result indicated that the reaction rate of chain‐walking is faster than that of subsequent arylation. The ligand relay catalysis strategy could also facilitate the asymmetric *ipso*‐hydroarylation (Scheme 1b). In contrast, lower yield and *rr* were obtained with a single chiral ligand. The alkene scope could also be extended to heteroatom *O*‐substituted olefins (**1w** and **4i**), although only moderate *ee* was obtained in these cases (Scheme 1c). The practicality and synthetic flexibility of the method were further illustrated by the facile synthesis of two bioactive molecules, (*S*)‐nicotine (**7**) and a CDK8 inhibitor (**8**), from the corresponding chiral *α*‐arylated pyrrolidine cores (**6d** and **6b**) which were successfully obtained through the key migratory hydroarylation approach from commercially available starting materials (Scheme 1d).

## 3 Conclusion

In summary, we have described herein a NiH‐catalyzed asymmetric migratory hydroarylation to access both cyclic and acyclic, enantioenriched *α*‐(hetero)aryl‐substituted amines and their derivatives under mild conditions. Significantly, through a key dynamic ligand exchange process, this ligand relay catalytic strategy enhances whole reaction efficiency with simultaneous control of regio‐ and enantioselectivity, allowing the employment of readily available unactivated remote alkenes or their isomeric mixtures as starting materials. The synthetic merits of this method were demonstrated by the facile synthesis of (*S*)‐nicotine and a pharmaceutical agent.

## Conflict of Interest

The authors declare the following competing financial interest: A provisional patent application has been filed.

## Supporting information

Supporting Information

## Data Availability

The data that support the findings of this study are available in the supplementary material of this article.
