# Supplementary material for: Ligand Relay Catalysis Enables Asymmetric Migratory Hydroarylation for the Concise Synthesis of Chiral α‐(Hetero)Aryl‐Substituted Amines
Source: Adv Sci (Weinh). 2024 Feb 28;11(16):2306447. doi: 10.1002/advs.202306447 (PMC11040341; doi:10.1002/advs.202306447)
Supplement: Supplementary file 1 — Supporting Information [file ADVS-11-2306447-s001.pdf]

ADVANCED SCIENCE
Open Access

Supporting Information

for *Adv. Sci.*, DOI 10.1002/advs.202306447

Ligand Relay Catalysis Enables Asymmetric Migratory Hydroarylation for the Concise Synthesis of Chiral α-(Hetero)Aryl-Substituted Amines

Junqian Zhou, Yuli He, Zihao Liu, You Wang and Shaolin Zhu**

Supporting Information

Ligand Relay Catalysis Enables Asymmetric Migratory Hydroarylation for the Concise Synthesis of Chiral α-(Hetero)Aryl-Substituted Amines

Junqian Zhou, Yuli He, Zihao Liu, You Wang, and Shaolin Zhu**

J. Zhou, Y. He, Z. Liu, Y. Wang, S. Zhu

State Key Laboratory of Coordination Chemistry, Jiangsu Key Laboratory of Advanced Organic Materials, Chemistry and Biomedicine Innovation Center (ChemBIC), School of Chemistry and Chemical Engineering, Nanjing University

Nanjing 210093, P. R. China

S. Zhu

School of Chemistry and Chemical Engineering, Henan Normal University

Xinxiang 453007, P. R. China

S. Zhu

Shanghai Key Laboratory for Molecular Engineering of Chiral Drugs, Shanghai Jiao Tong University

Shanghai 200240, P. R. China

E-mail: wangyou@nju.edu.cn; shaolinzhu@nju.edu.cn

Table of Contents

1. General Information 1

2. Asymmetric Migratory Hydroarylation to Access Enantioenriched α-(Hetero)Aryl-Substituted Amines...3

3. Asymmetric Migratory Hydroarylation to Access Enantioenriched α-(Hetero)Aryl-Substituted *N*-heterocycles 22

4. Competition Experiment: Remote Alkene vs *Ipso*- Alkene.............30

5. *Ipso*-Control Experiments 31

6. Extension of Alkene Scope 31

7. Practical synthesis of (S)-nicotine and a CDK8 inhibitor...............34

8. Conditions Optimization 37

9. References 39

10. Spectroscopic Data (NMR Spectrum)...39

11. Spectroscopic Data (HPLC Spectrum) 115

1. General Information

General reagent information. Solvents were either purified and dried by passage through alumina and Q5 reactant-packed columns on a solvent purification system or bought from the commercial sources and transferred to the glovebox without exposure to air. Other commercial reagents were purchased from Sigma-Aldrich, Acros, Alfa Aesar, TCI, Aladdin, J&K, Energy Chemical, Bide Pharmatech Ltd. and were used as received. Flash chromatography was performed using glass columns with silica gel (*SiliaFlash*® P60, particle size 40-63 µm, SiliCycle). **$Ni(NO_3)_2·6H_2O$** (CAS 13478-00-7) was purchased from Sigma-Aldrich and stored under nitrogen in glovebox; **Na_2CO_3** (CAS 497-19-8, Cat. No. 11552) was purchased from Alfa Aesar (anhydrous, ACS, 99.5% min); **K_2CO_3** (CAS 584-08-7, Cat. No. 012609.22) was purchased from Alfa Aesar (ACS, 99.0% min); **NaI** (CAS 7681-82-5) was purchased from 3A Materials (ultra dry, 99.99% metals basis); **DMMS** (Dimethoxymethylsilane, CAS 16881-77-9) was purchased from TCI and stored under nitrogen at –20 °C in glove box; **DEMS** (Diethoxymethylsilane, CAS 2031-62-1) was purchased from TCI and stored under nitrogen at –20 °C in glove box; **NMP** (*N*-Methyl-2-pyrrolidone, CAS 872-50-4) was purchased from J&K (99.5%, SuperDry, with molecular sieves, J&KSeal) and stored under nitrogen in glove box; **DMF** (*N*,*N*-Dimethylformamide, CAS 68-12-2) was purchased from Energy Chemical (99.9%, Extra Dry, with molecular sieves, EnergySeal) and stored under nitrogen in glove box; **DMA** (*N*,*N*-Dimethylacetamide, CAS 127-19-5) was purchased from J&K (99.8%, SuperDry, with molecular sieves, J&KSeal) and stored under nitrogen in glove box; **DME** (1,2-Dimethoxyethane, CAS 110-71-4) was purchased from J&K (for synthesis, 99.5%) and stored under nitrogen in glove box; **Tol** (Toluene, CAS 108-88-3) and **Et_2O** (Diethyl Ether, CAS 60-29-7) were purchased from Nanjing Reagent and were purified and dried by passage through alumina and Q5 reactant-packed columns on a solvent purification system.

General analytical information. All compounds (starting materials and products) were characterized by ^{1}H NMR, ^{13}C NMR, IR spectroscopy and high-resolution mass spectrometry. ^{1}H NMR spectra were recorded on Bruker 500 MHz spectrometer and

are referenced relative to residual $CDCl_3$ proton signals at δ 7.26 ppm. ^{19}F NMR spectra were recorded on a Bruker 500 MHz spectrometer and are referenced to $CFCl_3$ (δ 0.0 ppm). Data for ^1H and ^{19}F NMR are reported as follows: chemical shift (δ ppm), multiplicity (s = singlet, d = doublet, t = triplet, q = quartet, m = multiplet, br = broad), integration, and coupling constant (Hz). ^{13}C NMR spectra were recorded on a Bruker 500 MHz spectrometer and are referenced to $CDCl_3$ at δ 77.16 ppm. The ^{13}C NMR spectra were obtained with ^1H decoupling. Data for ^{13}C NMR are reported in terms of chemical shift and multiplicity where appropriate. IR spectra were obtained on a Bruker Alpha and was reported in terms of frequency of absorption (cm^{-1}). GC analysis were performed on Agilent 7890 or 8890 gas chromatograph with an FID detector using a J&W DB-1 column (10 m, 0.1 mm I.D.). Low Resolution Mass spectra were obtained from on an Agilent 5977A GC-MS. High Resolution Mass Spectra were obtained from on an Agilent 6540 Q-TOF mass spectrometer, operating electrospray ionization (ESI) mode. High pressure liquid chromatography (HPLC) was performed on Agilent 1260 Series chromatographs using Daicel Chiralcel or Chiralpak columns (250 mm). Optical rotations were measured on a Rudolph Research Analytical Autopol VI automatic polarimeter using a 50 mm pathlength cell at 589 nm with $[\alpha]_D$ values reported in degrees; concentration (c) is in g/100 mL.

Medium-sized screw-cap test tubes (8 mL) were used for all 0.20 mmol scale reactions:

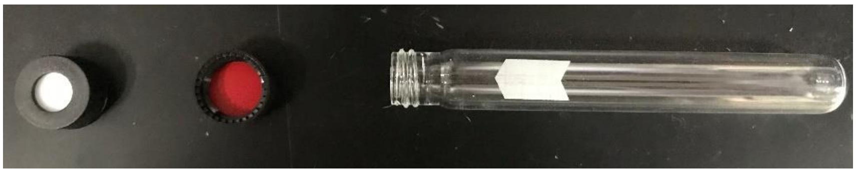

Figure S1. Fisher 13×100 mm tube (Cat. No. 14-959-35C)

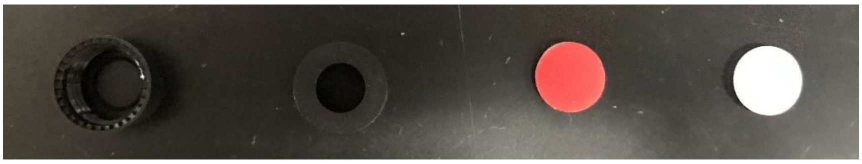

Figure S2. Cap with Septa: Thermo Scientific ASM PHN CAP w/PTFE/SIL (Cat. No. 03378316)

2. Asymmetric Migratory Hydroarylation to Access Enantioenriched α-(Hetero)Aryl-Substituted Amines

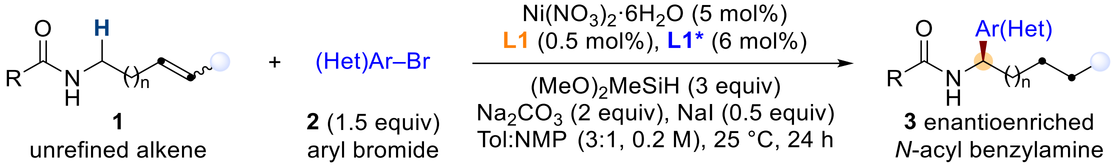

General procedure (A) for the asymmetric migratory hydroarylation. In a nitrogen-filled glove box, to an oven-dried 8 mL screw-cap vial equipped with a magnetic stir bar was added $Ni(NO_3)_2 \cdot 6H_2O$ (2.9 mg, 5.0 mol%), **L1*** (5.8 mg, 6.0 mol%), Na_2CO_3 (42.4 mg, 2.0 equiv), NaI (15.0 mg, 0.5 equiv), **L1** [0.29 mg, 0.75 mL stock solution (11.8 mg/ 30 mL in toluene)] and anhydrous NMP (0.25 mL). The mixture was stirred for 5 min at room temperature, at which time *N*-(but-3-en-1-yl)benzamide (35.0 mg, 0.20 mmol, 1.0 equiv), methyl 4-bromobenzoate (64.5 mg, 0.30 mmol, 1.5 equiv) and DMMS (74 µL, 0.60 mmol, 3.0 equiv) were added to the resulting mixture in this order. The tube was sealed with a teflon-lined screw cap, removed from the glove box and the reaction was stirred at 25 °C for up to 24 h (the mixture was stirred at 800 rpm, ensuring that the base was uniformly suspended). After the reaction was complete, the reaction mixture was directly filtered through a short pad of silica gel [EtOAc in petroleum ether (PE)] to give the crude product. *n*-Dodecane (20 µL) was added as an internal standard for GC analysis. The product was purified by chromatography on silica gel for each substrate. The yields reported are the average of at least two experiments, unless otherwise indicated. The enantiomeric excesses (% *ee*) were determined by HPLC analysis using chiral stationary phases.

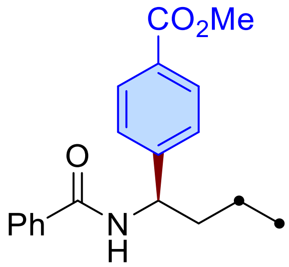

Methyl (*R*)-4-(1-benzamidobutyl)benzoate (Figure 2, **3a**). From methyl 4-bromobenzoate (64.5 mg, 0.30 mmol, 1.5 equiv), the title compound was prepared following the general procedure **A** using $Ni(NO_3)_2 \cdot 6H_2O$ (2.9 mg, 5.0 mol%), **L1*** (5.8 mg, 6.0 mol%), **L1** [0.29 mg, 0.75 mL (11.8 mg/ 30 mL in toluene)], Na_2CO_3 (42.4 mg, 2.0 equiv), NaI (15.0 mg, 0.5 equiv), *N*-(but-3-en-1-yl)benzamide (35.0 mg, 0.20 mmol, 1.0 equiv), DMMS (74 μL, 0.60 mmol, 3.0 equiv) and anhydrous NMP (0.25 mL). The reaction mixture was stirred for 24 h at 25 °C. The crude material was purified by flash column chromatography (petroleum ether/EtOAc = 5:1) to provide the title compound as a white solid in 72% yield (44.1 mg) with 97:3 rr.

^{1}H NMR (500 MHz, $CDCl_3$) δ 8.01 (d, *J* = 8.3 Hz, 2H), 7.81 – 7.70 (m, 2H), 7.58 – 7.46 (m, 1H), 7.48 – 7.37 (m, 4H), 6.39 (d, *J* = 7.6 Hz, 1H), 5.21 (q, *J* = 7.6 Hz, 1H), 3.90 (s, 3H), 2.03 – 1.73 (m, 2H), 1.55 – 1.31 (m, 2H), 0.96 (t, *J* = 7.3 Hz, 3H);

^{13}C NMR (126 MHz, $CDCl_3$) δ 167.0, 147.9, 134.5, 131.8, 130.2, 129.4, 128.8, 127.0, 126.7, 53.7, 52.2, 38.5, 19.6, 14.0;

HRMS (ESI) calcd. for $C_{19}H_{21}NO_3Na$ $[M+Na]^+$ *m/z* 334.1413, found 334.1414;

IR (neat, cm^{-1}) 3356, 2920, 1632, 1282, 709;

m.p. 131.1 – 132.4 °C;

$[\alpha]_D^{17}$ = +2.0 (c = 0.51, $CHCl_3$); 92% *ee*;

HPLC analysis CHIRALPAK® AD-H column, 20% ^{i}PrOH in hexane, 0.8 mL/min, 254 nm UV detector, t_R (major) = 11.2 min, t_R (minor) = 16.9 min.

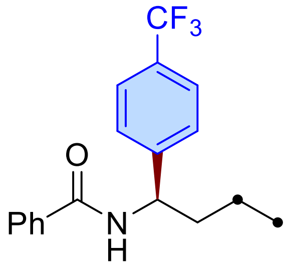

(*R*)-*N*-(1-(4-(Trifluoromethyl)phenyl)butyl)benzamide[1] (Figure 2, **3b**). From 1-bromo-4-(trifluoromethyl)benzene (67.5 mg, 0.30 mmol, 1.5 equiv), the title compound was prepared following the general procedure **A** using $Ni(NO_3)_2·6H_2O$ (2.9 mg, 5.0 mol%), **L1*** (5.8 mg, 6.0 mol%), **L1** [0.29 mg, 0.75 mL (11.8 mg/ 30 mL in toluene)], Na_2CO_3 (42.4 mg, 2.0 equiv), NaI (15.0 mg, 0.5 equiv), *N*-(but-3-en-1-yl)benzamide (35.0 mg, 0.20 mmol, 1.0 equiv), DMMS (74 µL, 0.60 mmol, 3.0 equiv) and anhydrous NMP (0.25 mL). The reaction mixture was stirred for 24 h at 25 °C. The crude material was purified by flash column chromatography (petroleum ether/EtOAc = 5:1) to provide the title compound as a white solid in 67% yield (42.8 mg) with 95:5 rr.

^{1}H NMR (500 MHz, $CDCl_3$) δ 7.77 (d, *J* = 7.5 Hz, 2H), 7.63 – 7.54 (m, 2H), 7.52 – 7.38 (m, 5H), 6.56 (d, *J* = 7.6 Hz, 1H), 5.19 (q, *J* = 7.5 Hz, 1H), 1.93 – 1.73 (m, 2H), 1.51 – 1.25 (m, 2H), 0.95 (t, *J* = 7.4 Hz, 3H);

^{13}C NMR (126 MHz, $CDCl_3$) δ 167.1, 146.8, 134.4, 131.8, 129.6 (q, *J* = 32.7 Hz), 128.7, 127.1, 127.0, 125.7 (q, *J* = 3.7 Hz), 124.2 (q, *J* = 272.2 Hz), 53.6, 38.5, 19.6, 13.9;

^{19}F NMR (471 MHz, $CDCl_3$) δ –62.5;

HRMS (ESI) calcd. for $C_{18}H_{18}F_3NONa$ $[M+Na]^+$ *m/z* 344.1232, found 344.1236;

IR (neat, cm^{-1}) 3322, 1633, 1330, 1121, 760;

m.p. 148.9 – 150.1 °C;

$[\alpha]_D^{17}$ = –3.5 (c = 1.44, $CHCl_3$); 90% *ee*; **$[\alpha]_D^{17}$** = –10.6 (c = 1.0, CH_2Cl_2)

HPLC analysis CHIRALPAK® AD-H column, 20% ^{i}PrOH in hexane, 0.8 mL/min, 220 nm UV detector, t_R (major) = 6.0 min, t_R (minor) = 7.6 min.

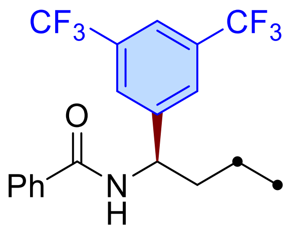

(*R*)-*N*-(1-(3,5-Bis(trifluoromethyl)phenyl)butyl)benzamide (Figure 2, **3c**). From 1-bromo-3,5-bis(trifluoromethyl)benzene (87.9 mg, 0.30 mmol, 1.5 equiv), the title compound was prepared following the general procedure **A** using $Ni(NO_3)_2·6H_2O$ (2.9 mg, 5.0 mol%), **L1*** (5.8 mg, 6.0 mol%), **L1** [0.29 mg, 0.75 mL (11.8 mg/ 30 mL in

toluene)], Na_2CO_3 (42.4 mg, 2.0 equiv), NaI (15.0 mg, 0.5 equiv), *N*-(but-3-en-1-yl)benzamide (35.0 mg, 0.20 mmol, 1.0 equiv), DMMS (74 µL, 0.60 mmol, 3.0 equiv) and anhydrous NMP (0.25 mL). The reaction mixture was stirred for 24 h at 25 °C. The crude material was purified by flash column chromatography (petroleum ether/EtOAc = 5:1) to provide the title compound as a white solid in 53% yield (41.2 mg) with >99:1 rr.

^{1}H NMR (500 MHz, $CDCl_3$) δ 7.82 – 7.70 (m, 5H), 7.61 – 7.48 (m, 1H), 7.46 – 7.38 (m, 2H), 6.63 (d, *J* = 7.5 Hz, 1H), 5.23 (q, *J* = 7.5 Hz, 1H), 1.97 – 1.76 (m, 2H), 1.57 – 1.29 (m, 2H), 0.97 (t, *J* = 7.4 Hz, 3H);

^{13}C NMR (126 MHz, $CDCl_3$) δ 167.2, 145.7, 134.0, 132.1, 132.0 (q, *J* = 34.0 Hz), 128.9, 127.1, 126.9 (d, *J* = 2.5 Hz), 123.4 (q, *J* = 273.4 Hz), 121.5 (p, *J* = 3.8 Hz), 53.5, 38.5, 19.7, 13.9;

^{19}F NMR (471 MHz, $CDCl_3$) δ –62.8;

HRMS (ESI) calcd. for $C_{19}H_{17}F_6NONa$ $[M+Na]^+$ *m/z* 412.1106, found 412.1108;

IR (neat, cm^{-1}) 3323, 1264, 904, 724;

m.p. 135.9 – 136.0 °C;

$[\alpha]_D^{17}$ = –19.0 (c = 1.16, $CHCl_3$); 94% *ee*;

HPLC analysis CHIRALPAK® AD-H column, 5% ^{i}PrOH in hexane, 0.8 mL/min, 220 nm UV detector, t_R (major) = 7.8 min, t_R (minor) = 8.7 min.

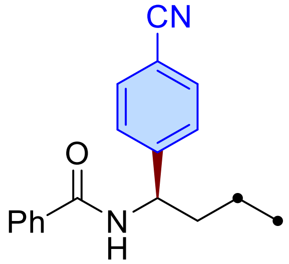

(*R*)-*N*-(1-(4-Cyanophenyl)butyl)benzamide (Figure 2, **3d**). From 4-bromobenzonitrile (54.3 mg, 0.30 mmol, 1.5 equiv), the title compound was prepared following the general procedure **A** using $Ni(NO_3)_2·6H_2O$ (2.9 mg, 5.0 mol%), **L1*** (5.8 mg, 6.0 mol%), **L1** [0.29 mg, 0.75 mL (11.8 mg/ 30 mL in toluene)], Na_2CO_3 (42.4 mg, 2.0 equiv), NaI (15.0 mg, 0.5 equiv), *N*-(but-3-en-1-yl)benzamide (35.0 mg, 0.20 mmol, 1.0 equiv), DMMS (74 µL, 0.60 mmol, 3.0 equiv) and anhydrous NMP (0.25 mL). The

reaction mixture was stirred for 24 h at 25 °C. The crude material was purified by flash column chromatography (petroleum ether/EtOAc = 5:1) to provide the title compound as a white solid in 52% yield (29.1 mg) with 95:5 rr.

^{1}H NMR (500 MHz, $CDCl_3$) δ 7.79 – 7.74 (m, 2H), 7.63 – 7.63 (m, 2H), 7.55 – 7.48 (m, 1H), 7.48 – 7.39 (m, 4H), 6.45 (d, J = 7.6 Hz, 1H), 5.16 (q, J = 7.5 Hz, 1H), 1.93 – 1.78 (m, 2H), 1.50 – 1.31 (m, 2H), 0.96 (t, J = 7.3 Hz, 3H);

^{13}C NMR (126 MHz, $CDCl_3$) δ 167.1, 148.3, 134.2, 132.6, 131.9, 128.8, 127.4, 127.1, 118.9, 111.3, 53.7, 38.3, 19.6, 13.9;

HRMS (ESI) calcd. for $C_{18}H_{18}N_2ONa$ $[M+Na]^+$ m/z 301.1311, found 301.1309;

IR (neat, cm^{-1}) 3019, 2229, 1214, 744;

m.p. 148.0 – 149.2 °C;

$[\alpha]_D^{17}$ = –16.2 (c = 0.58, $CHCl_3$); 95% *ee*;

HPLC analysis CHIRALPAK® AD-H column, 20% ^{i}PrOH in hexane, 0.8 mL/min, 220 nm UV detector, t_R (major) = 10.7 min, t_R (minor) = 14.9 min.

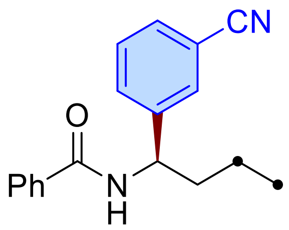

(*R*)-*N*-(1-(3-Cyanophenyl)butyl)benzamide (Figure 2, **3e**). From 3-bromobenzonitrile (54.3 mg, 0.30 mmol, 1.5 equiv), the title compound was prepared following the general procedure **A** using $Ni(NO_3)_2 \cdot 6H_2O$ (2.9 mg, 5.0 mol%), **L1*** (5.8 mg, 6.0 mol%), **L1** [0.29 mg, 0.75 mL (11.8 mg/ 30 mL in toluene)], Na_2CO_3 (42.4 mg, 2.0 equiv), NaI (15.0 mg, 0.5 equiv), *N*-(but-3-en-1-yl)benzamide (35.0 mg, 0.20 mmol, 1.0 equiv), DMMS (74 μL, 0.60 mmol, 3.0 equiv) and anhydrous NMP (0.25 mL). The reaction mixture was stirred for 24 h at 25 °C. The crude material was purified by flash column chromatography (petroleum ether/EtOAc = 5:1) to provide the title compound as a white solid in 57% yield (31.9 mg) with 96:4 rr.

^{1}H NMR (500 MHz, $CDCl_3$) δ 7.79 – 7.75 (m, 2H), 7.64 (s, 1H), 7.60 (d, J = 8.1 Hz, 1H), 7.56 – 7.49 (m, 2H), 7.48 – 7.41 (m, 3H), 6.46 (d, J = 7.7 Hz, 1H), 5.15 (q, J = 7.5 Hz, 1H), 1.95 – 1.80 (m, 2H), 1.51 – 1.30 (m, 2H), 0.97 (t, J = 7.3 Hz, 3H);

^{13}C NMR (126 MHz, $CDCl_3$) δ 167.1, 144.5, 134.1, 132.0, 131.6, 131.1, 130.1, 129.6, 128.8, 127.1, 119.0, 112.9, 53.4, 38.4, 19.6, 13.9;

HRMS (ESI) calcd. for $C_{18}H_{18}N_2ONa$ $[M+Na]^+$ *m/z* 301.1311, found 301.1310;

IR (neat, cm^{-1}) 3019, 1634, 1214, 745;

m.p. 120.6 – 121.7 °C;

$[\alpha]_D^{17}$ = –10.0 (c = 0.34, $CHCl_3$); 90% *ee*;

HPLC analysis CHIRALPAK® AD-H column, 20% ^{i}PrOH in hexane, 0.8 mL/min, 220 nm UV detector, t_R (major) = 7.2 min, t_R (minor) = 9.8 min.

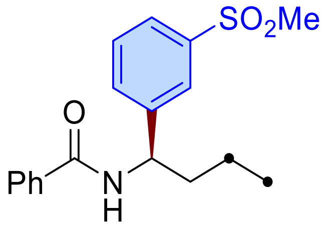

(*R*)-*N*-(1-(3-(Methylsulfonyl)phenyl)butyl)benzamide (Figure 2, **3f**). From 1-bromo-3-(methylsulfonyl)benzene (70.2 mg, 0.30 mmol, 1.5 equiv), the title compound was prepared following the general procedure **A** using $Ni(NO_3)_2 \cdot 6H_2O$ (2.9 mg, 5.0 mol%), **L1*** (5.8 mg, 6.0 mol%), **L1** [0.29 mg, 0.75 mL (11.8 mg/ 30 mL in toluene)], Na_2CO_3 (42.4 mg, 2.0 equiv), NaI (15.0 mg, 0.5 equiv), *N*-(but-3-en-1-yl)benzamide (35.0 mg, 0.20 mmol, 1.0 equiv), DMMS (74 μL, 0.60 mmol, 3.0 equiv) and anhydrous NMP (0.25 mL). The reaction mixture was stirred for 24 h at 25 °C. The crude material was purified by flash column chromatography (petroleum ether/EtOAc = 5:1) to provide the title compound as a white solid in 50% yield (32.5 mg) with 97:3 rr.

^{1}H NMR (500 MHz, $CDCl_3$) δ 7.97 – 7.91 (m, 1H), 7.87 – 7.75 (m, 3H), 7.70 – 7.63 (m, 1H), 7.57 – 7.46 (m, 2H), 7.45 – 7.38 (m, 2H), 6.65 (d, *J* = 7.7 Hz, 1H), 5.22 (q, *J* = 7.6 Hz, 1H), 3.03 (s, 3H), 1.96 – 1.78 (m, 2H), 1.59 – 1.29 (m, 2H), 0.94 (t, *J* = 7.3 Hz, 3H);

^{13}C NMR (126 MHz, $CDCl_3$) δ 167.1, 145.1, 141.0, 134.2, 132.6, 131.9, 129.8, 128.8, 127.1, 126.3, 125.0, 53.6, 44.6, 38.6, 19.7, 13.9;

HRMS (ESI) calcd. for $C_{18}H_{21}NO_3SNa$ $[M+Na]^+$ *m/z* 354.1134, found 354.1132;

IR (neat, cm^{-1}) 3329, 1650, 1214, 743;

m.p. 179.1 – 180.6 °C;

[α]$_D$^{17} = –7.6 (c = 0.55, $CHCl_3$); 93% *ee*;

HPLC analysis CHIRALPAK® AD-H column, 20% ^{i}PrOH in hexane, 0.8 mL/min, 220 nm UV detector, t_R (major) = 9.4 min, t_R (minor) = 13.0 min.

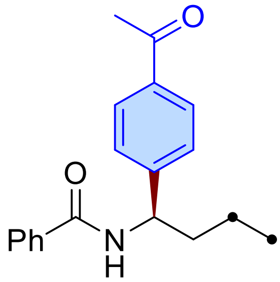

(*R*)-*N*-(1-(4-Acetylphenyl)butyl)benzamide (Figure 2, **3g**). From 1-(4-bromophenyl)ethan-1-one (59.4 mg, 0.30 mmol, 1.5 equiv), the title compound was prepared following the general procedure **A** using $Ni(NO_3)_2·6H_2O$ (2.9 mg, 5.0 mol%), **L1*** (5.8 mg, 6.0 mol%), **L1** [0.29 mg, 0.75 mL (11.8 mg/ 30 mL in toluene)], Na_2CO_3 (42.4 mg, 2.0 equiv), NaI (15.0 mg, 0.5 equiv), *N*-(but-3-en-1-yl)benzamide (35.0 mg, 0.20 mmol, 1.0 equiv), DMMS (74 µL, 0.60 mmol, 3.0 equiv) and anhydrous NMP (0.25 mL). The reaction mixture was stirred for 24 h at 25 °C. The crude material was purified by flash column chromatography (petroleum ether/EtOAc = 5:1) to provide the title compound as a white solid in 58% yield (34.4 mg) with 94:6 rr.

^{1}H NMR (500 MHz, $CDCl_3$) δ 7.98 – 7.89 (m, 2H), 7.82 – 7.73 (m, 2H), 7.52 – 7.45 (m, 1H), 7.45 – 7.36 (m, 4H), 6.55 (d, *J* = 7.6 Hz, 1H), 5.19 (q, *J* = 7.6 Hz, 1H), 2.57 (s, 3H), 1.96 – 1.80 (m, 2H), 1.52 – 1.29 (m, 2H), 0.95 (t, *J* = 7.4 Hz, 3H);

^{13}C NMR (126 MHz, $CDCl_3$) δ 197.8, 167.0, 148.2, 136.4, 134.5, 131.8, 129.0, 128.8, 127.1, 126.9, 53.7, 38.5, 26.8, 19.6, 13.9;

HRMS (ESI) calcd. for $C_{19}H_{21}NO_2Na$ $[M+Na]^+$ *m/z* 318.1464, found 318.1466;

IR (neat, cm^{-1}) 3019, 1633, 1214, 745;

m.p. 157.2 – 158.6 °C;

[α]$_D$^{17} = +5.5 (c = 0.62, $CHCl_3$); 92% *ee*;

HPLC analysis CHIRALPAK® AD-H column, 20% ^{i}PrOH in hexane, 0.8 mL/min, 220 nm UV detector, t_R (major) = 10.5 min, t_R (minor) = 18.2 min.

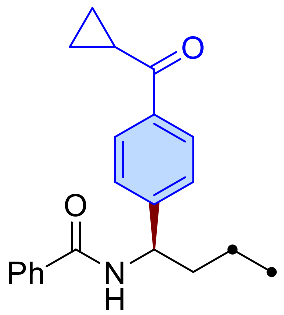

(*R*)-*N*-(1-(4-(Cyclopropanecarbonyl)phenyl)butyl)benzamide (Figure 2, **3h**). From (4-bromophenyl)(cyclopropyl)methanone (67.5 mg, 0.30 mmol, 1.5 equiv), the title compound was prepared following the general procedure **A** using $Ni(NO_3)_2 \cdot 6H_2O$ (2.9 mg, 5.0 mol%), **L1*** (5.8 mg, 6.0 mol%), **L1** [0.29 mg, 0.75 mL (11.8 mg/ 30 mL in toluene)], Na_2CO_3 (42.4 mg, 2.0 equiv), NaI (15.0 mg, 0.5 equiv), *N*-(but-3-en-1-yl)benzamide (35.0 mg, 0.20 mmol, 1.0 equiv), DMMS (74 μL, 0.60 mmol, 3.0 equiv) and anhydrous NMP (0.25 mL). The reaction mixture was stirred for 24 h at 25 °C. The crude material was purified by flash column chromatography (petroleum ether/EtOAc = 5:1) to provide the title compound as a white solid in 50% yield (32.2 mg) with 95:5 rr.

^{1}H NMR (500 MHz, $CDCl_3$) δ 8.00 (d, *J* = 8.4 Hz, 2H), 7.84 – 7.70 (m, 2H), 7.52 – 7.38 (m, 5H), 6.35 (d, *J* = 7.8 Hz, 1H), 5.22 (q, *J* = 7.5 Hz, 1H), 2.74 – 2.61 (m, 1H), 2.02 – 1.81 (m, 2H), 1.53 – 1.32 (m, 2H), 1.25 – 1.13 (m, 2H), 1.09 – 1.00 (m, 2H), 0.97 (t, *J* = 7.4 Hz, 3H);

^{13}C NMR (126 MHz, $CDCl_3$) δ 200.3, 166.9, 147.6, 137.3, 131.8, 128.8, 128.7, 127.0, 126.8, 53.7, 38.5, 19.7, 17.3, 14.0, 11.7;

HRMS (ESI) calcd. for $C_{21}H_{23}NO_2Na$ $[M+Na]^+$ *m/z* 344.1621, found 344.1626;

IR (neat, cm^{-1}) 3018, 1633, 1215, 747;

m.p. 183.3 – 184.7 °C;

$[\alpha]_D^{17}$ = +4.9 (c = 0.57, $CHCl_3$); 92% *ee*;

HPLC analysis CHIRALPAK® AD-H column, 20% ^{i}PrOH in hexane, 0.8 mL/min, 254 nm UV detector, t_R (major) = 13.9 min, t_R (minor) = 24.2 min.

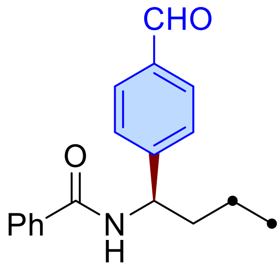

(*R*)-*N*-(1-(4-Formylphenyl)butyl)benzamide (Figure 2, **3i**). From 4-bromobenzaldehyde (55.5 mg, 0.30 mmol, 1.5 equiv), the title compound was prepared following the general procedure **A** using $Ni(NO_3)_2 \cdot 6H_2O$ (2.9 mg, 5.0 mol%), **L1*** (5.8 mg, 6.0 mol%), **L1** [0.29 mg, 0.75 mL (11.8 mg/ 30 mL in toluene)], Na_2CO_3 (42.4 mg, 2.0 equiv), NaI (15.0 mg, 0.5 equiv), *N*-(but-3-en-1-yl)benzamide (35.0 mg, 0.20 mmol, 1.0 equiv), DMMS (74 μL, 0.60 mmol, 3.0 equiv) and anhydrous NMP (0.25 mL). The reaction mixture was stirred for 24 h at 25 °C. The crude material was purified by flash column chromatography (petroleum ether/EtOAc = 5:1) to provide the title compound as a white solid in 66% yield (37.4 mg) with 97:3 rr.

^{1}H NMR (500 MHz, $CDCl_3$) δ 9.99 (s, 1H), 7.86 (d, *J* = 8.3 Hz, 2H), 7.81 – 7.74 (m, 2H), 7.55 – 7.48 (m, 3H), 7.48 – 7.40 (m, 2H), 6.39 (d, *J* = 7.7 Hz, 1H), 5.22 (q, *J* = 7.6 Hz, 1H), 2.00 – 1.80 (m, 2H), 1.51 – 1.33 (m, 2H), 0.97 (t, *J* = 7.3 Hz, 3H);

^{13}C NMR (126 MHz, $CDCl_3$) δ 192.0, 167.0, 149.8, 135.7, 134.4, 131.9, 130.4, 128.8, 127.3, 127.1, 53.8, 38.5, 19.7, 14.0;

HRMS (ESI) calcd. for $C_{18}H_{19}NO_2Na$ $[M+Na]^+$ *m/z* 304.1308, found 304.1305;

IR (neat, cm^{-1}) 3355, 2921, 1635, 1264, 729;

$[\alpha]_D^{17}$ = –1.6 (c = 0.37, $CHCl_3$); 89% *ee*;

m.p. 125.3 – 126.1 °C;

HPLC analysis CHIRALPAK® AD-H column, 20% ^{i}PrOH in hexane, 0.8 mL/min, 220 nm UV detector, t_R (major) = 10.6 min, t_R (minor) = 16.3 min.

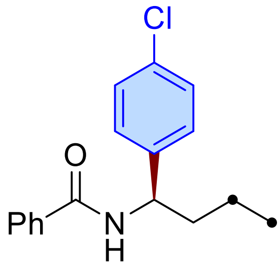

(*R*)-*N*-(1-(4-Chlorophenyl)butyl)benzamide (Figure 2, **3j**). From 1-bromo-4-chlorobenzene (57.0 mg, 0.30 mmol, 1.5 equiv), the title compound was prepared following the general procedure **A** using $Ni(NO_3)_2 \cdot 6H_2O$ (2.9 mg, 5.0 mol%), **L1*** (5.8 mg, 6.0 mol%), **L1** [0.29 mg, 0.75 mL (11.8 mg/ 30 mL in toluene)], Na_2CO_3 (42.4 mg, 2.0 equiv), NaI (15.0 mg, 0.5 equiv), *N*-(but-3-en-1-yl)benzamide (35.0 mg, 0.20 mmol, 1.0 equiv), DMMS (74 μL, 0.60 mmol, 3.0 equiv) and anhydrous NMP (0.25 mL). The reaction mixture was stirred for 24 h at 25 °C. The crude material was purified by flash column chromatography (petroleum ether/EtOAc = 5:1) to provide the title compound as a white solid in 72% yield (41.1 mg) with >99:1 rr.

^{1}H NMR (500 MHz, $CDCl_3$) δ 7.80 – 7.72 (m, 2H), 7.56 – 7.46 (m, 1H), 7.46 – 7.39 (m, 2H), 7.34 – 7.26 (m, 4H), 6.29 (d, *J* = 7.7 Hz, 1H), 5.14 (q, *J* = 7.6 Hz, 1H), 2.01 – 1.77 (m, 2H), 1.50 – 1.29 (m, 2H), 0.96 (t, *J* = 7.3 Hz, 3H);

^{13}C NMR (126 MHz, $CDCl_3$) δ 166.9, 141.2, 134.6, 133.2, 131.7, 129.0, 128.8, 128.1, 127.0, 53.3, 38.5, 19.7, 14.0;

HRMS (ESI) calcd. for $C_{17}H_{18}ClNONa$ $[M+Na]^+$ *m/z* 310.0969, found 310.0967;

IR (neat, cm^{-1}) 3326, 2926, 1632, 1214, 744;

m.p. 141.5 – 142.6 °C;

$[\alpha]_D^{17}$ = +1.6 (c = 0.37, $CHCl_3$); 89% *ee*;

HPLC analysis CHIRALPAK® AD-H column, 20% ^{i}PrOH in hexane, 0.8 mL/min, 220 nm UV detector, t_R (major) = 7.1 min, t_R (minor) = 9.5 min.

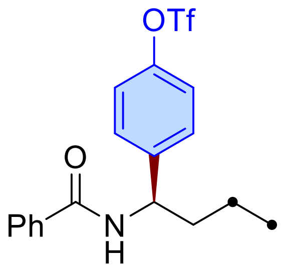

(*R*)-4-(1-Benzamidobutyl)phenyl trifluoromethanesulfonate (Figure 2, **3k**). From 4-bromophenyl trifluoromethanesulfonate (91.5 mg, 0.30 mmol, 1.5 equiv), the title compound was prepared following the general procedure **A** using $Ni(NO_3)_2 \cdot 6H_2O$ (2.9 mg, 5.0 mol%), **L1*** (5.8 mg, 6.0 mol%), **L1** [0.29 mg, 0.75 mL (11.8 mg/ 30 mL in toluene)], Na_2CO_3 (42.4 mg, 2.0 equiv), NaI (15.0 mg, 0.5 equiv), *N*-(but-3-en-1-

yl)benzamide (35.0 mg, 0.20 mmol, 1.0 equiv), DMMS (74 μL, 0.60 mmol, 3.0 equiv) and anhydrous NMP (0.25 mL). The reaction mixture was stirred for 24 h at 25 °C. The crude material was purified by flash column chromatography (petroleum ether/EtOAc = 5:1) to provide the title compound as a white solid in 68% yield (54.4 mg) with 96:4 rr.

^{1}H NMR (500 MHz, $CDCl_3$) δ 7.76 (d, J = 7.1 Hz, 2H), 7.57 – 7.48 (m, 1H), 7.46 – 7.38 (m, 4H), 7.24 – 7.20 (m, 2H), 6.49 (d, J = 7.6 Hz, 1H), 5.18 (q, J = 7.6 Hz, 1H), 1.93 – 1.77 (m, 2H), 1.52 – 1.29 (m, 2H), 0.95 (t, J = 7.3 Hz, 3H);

^{13}C NMR (126 MHz, $CDCl_3$) δ 167.0, 148.7, 143.4, 134.3, 131.8, 128.8, 128.6, 127.1, 121.6, 118.8 (q, J = 321.3 Hz), 53.1, 38.4, 19.7, 13.9;

^{19}F NMR (471 MHz, $CDCl_3$) δ -72.9;

HRMS (ESI) calcd. for $C_{18}H_{18}F_3NO_4SNa$ $[M+Na]^+$ *m/z* 424.0801, found 424.0801;

IR (neat, cm^{-1}) 3294, 1634, 1422, 1209, 1139, 888;

m.p. 110.0 – 111.4 °C;

$[\alpha]_D^{17}$ = +1.6 (c = 1.01, $CHCl_3$); 88% *ee*;

HPLC analysis CHIRALCEL® OD-H column, 20% ^{i}PrOH in hexane, 0.8 mL/min, 254 nm UV detector, t_R (minor) = 6.6 min, t_R (major) = 7.7 min.

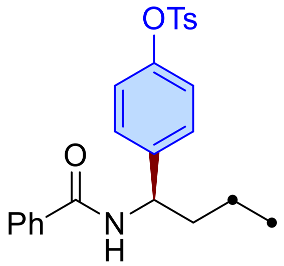

(*R*)-4-(1-Benzamidobutyl)phenyl 4-methylbenzenesulfonate (Figure 2, **3l**). From 4-bromophenyl 4-methylbenzenesulfonate (98.2 mg, 0.30 mmol, 1.5 equiv), the title compound was prepared following the general procedure **A** using $Ni(NO_3)_2{\cdot}6H_2O$ (2.9 mg, 5.0 mol%), **L1*** (5.8 mg, 6.0 mol%), **L1** [0.29 mg, 0.75 mL (11.8 mg/ 30 mL in toluene)], Na_2CO_3 (42.4 mg, 2.0 equiv), NaI (15.0 mg, 0.5 equiv), *N*-(but-3-en-1-yl)benzamide (35.0 mg, 0.20 mmol, 1.0 equiv), DMMS (74 μL, 0.60 mmol, 3.0 equiv) and anhydrous NMP (0.25 mL). The reaction mixture was stirred for 24 h at 25 °C. The crude material was purified by flash column chromatography (petroleum ether/EtOAc

= 5:1) to provide the title compound as colorless oil in 55% yield (46.2 mg) with 97:3 rr.

^{1}H NMR (500 MHz, $CDCl_3$) δ 7.77 – 7.68 (m, 4H), 7.54 – 7.40 (m, 3H), 7.33 – 7.25 (m, 4H), 6.99 – 6.93 (m, 2H), 6.25 (d, *J* = 7.8 Hz, 1H), 5.15 (q, *J* = 7.7 Hz, 1H), 2.44 (s, 3H), 1.92 – 1.76 (m, 2H), 1.45 – 1.30 (m, 2H), 0.95 (t, *J* = 7.3 Hz, 3H);

^{13}C NMR (126 MHz, $CDCl_3$) δ 166.9, 148.8, 145.5, 141.6, 134.5, 131.8, 129.9, 128.8, 128.6, 127.9, 127.0, 122.7, 53.1, 38.5, 21.9, 19.6, 13.9;

HRMS (ESI) calcd. for $C_{24}H_{25}NO_4SNa$ $[M+Na]^+$ *m/z* 446.1396, found 446.1393;

IR (neat, cm^{-1}) 3327, 2928, 1638, 1306,1149, 769;

$[\alpha]_D^{17}$ = – 11.2 (c = 0.34, $CHCl_3$); 91% *ee*;

HPLC analysis CHIRALPAK® AD-H column, 30% ^{i}PrOH in hexane, 0.8 mL/min, 254 nm UV detector, t_R (minor) = 11.6 min, t_R (major) = 14.5 min.

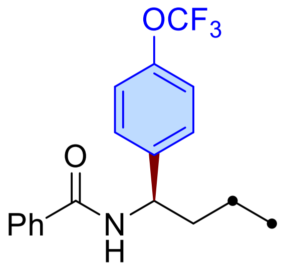

(*R*)-*N*-(1-(4-(Trifluoromethoxy)phenyl)butyl)benzamide (Figure 2, **3m**). From 1-bromo-4-(trifluoromethoxy)benzene (72.3 mg, 0.30 mmol, 1.5 equiv), the title compound was prepared following the general procedure **A** using $Ni(NO_3)_2·6H_2O$ (2.9 mg, 5.0 mol%), **L1*** (5.8 mg, 6.0 mol%), **L1** [0.29 mg, 0.75 mL (11.8 mg/ 30 mL in toluene)], Na_2CO_3 (42.4 mg, 2.0 equiv), NaI (15.0 mg, 0.5 equiv), *N*-(but-3-en-1-yl)benzamide (35.0 mg, 0.20 mmol, 1.0 equiv), DMMS (74 μL, 0.60 mmol, 3.0 equiv) and anhydrous NMP (0.25 mL). The reaction mixture was stirred for 24 h at 25 °C. The crude material was purified by flash column chromatography (petroleum ether/EtOAc = 5:1) to provide the title compound as a white solid in 62% yield (42.0 mg) with 97:3 rr.

^{1}H NMR (500 MHz, $CDCl_3$) δ 7.76 (d, *J* = 7.3 Hz, 2H), 7.54 – 7.46 (m, 1H), 7.45 – 7.33 (m, 4H), 7.18 (d, *J* = 8.2 Hz, 2H), 6.40 (d, *J* = 8.0 Hz, 1H), 5.17 (q, *J* = 7.6 Hz, 1H), 2.34 – 1.64 (m, 2H), 1.67 – 1.16 (m, 2H), 0.96 (t, *J* = 7.3 Hz, 3H);

^{13}C NMR (126 MHz, $CDCl_3$) δ 166.9, 148.5, 141.4, 134.5, 131.8, 128.7, 128.1, 127.1, 121.3, 120.6 (q, J = 258.3 Hz), 53.2, 38.5, 19.7, 13.9;

^{19}F NMR (471 MHz, $CDCl_3$) δ – 57.9;

HRMS (ESI) calcd. for $C_{18}H_{18}F_3NO_2Na$ $[M+Na]^+$ m/z 360.1182, found 360.1179;

IR (neat, cm^{-1}) 3314, 2924, 1638, 1215, 745;

m.p. 127.7 – 128.7 °C;

$[\alpha]_D^{17}$ = +2.8 (c = 0.50, $CHCl_3$); 86% *ee*;

HPLC analysis CHIRALPAK® AD-H column, 20% ^{i}PrOH in hexane, 0.8 mL/min, 220 nm UV detector, t_R (major) = 5.7 min, t_R (minor) = 6.9 min.

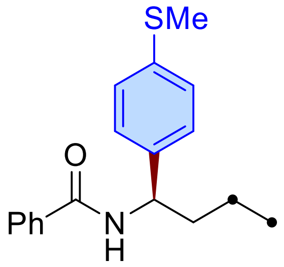

(*R*)-*N*-(1-(4-(Methylthio)phenyl)butyl)benzamide (Figure 2, **3n**). From (4-bromophenyl)(methyl)sulfane (60.9 mg, 0.30 mmol, 1.5 equiv), the title compound was prepared following the general procedure **A** using $Ni(NO_3)_2·6H_2O$ (2.9 mg, 5.0 mol%), **L1*** (5.8 mg, 6.0 mol%), **L1** [0.29 mg, 0.75 mL (11.8 mg/ 30 mL in toluene)], Na_2CO_3 (42.4 mg, 2.0 equiv), NaI (15.0 mg, 0.5 equiv), *N*-(but-3-en-1-yl)benzamide (35.0 mg, 0.20 mmol, 1.0 equiv), DMMS (74 μL, 0.60 mmol, 3.0 equiv) and anhydrous NMP (0.25 mL). The reaction mixture was stirred for 24 h at 25 °C. The crude material was purified by flash column chromatography (petroleum ether/EtOAc = 5:1) to provide the title compound as a white solid in 46% yield (27.6 mg) with 98:2 rr.

^{1}H NMR (500 MHz, $CDCl_3$) δ 7.83 – 7.71 (m, 2H), 7.55 – 7.40 (m, 1H), 7.44 – 7.37 (m, 2H), 7.33 – 7.24 (m, 2H), 7.26 – 7.20 (m, 2H), 6.37 (d, J = 8.1 Hz, 1H), 5.13 (q, J = 7.6 Hz, 1H), 2.46 (s, 3H), 1.95 – 1.78 (m, 2H), 1.53 – 1.29 (m, 2H), 0.94 (t, J = 7.4 Hz, 3H);

^{13}C NMR (126 MHz, $CDCl_3$) δ 166.8, 139.5, 137.5, 134.7, 131.6, 128.7, 127.3, 127.1, 127.0, 53.4, 38.4, 19.7, 16.1, 14.0;

HRMS (ESI) calcd. for $C_{18}H_{21}NOSNa$ $[M+Na]^+$ m/z 322.1236, found 322.1233;

IR (neat, cm^{-1}) 3321, 1631, 1214, 744;

m.p. 177.3 – 179.3 °C;

$[\alpha]_D^{17}$ = +21.4 (c = 0.72, $CHCl_3$); 92% *ee*;

HPLC analysis CHIRALPAK® AD-H column, 20% ^{i}PrOH in hexane, 0.8 mL/min, 254 nm UV detector, t_R (major) = 8.8 min, t_R (minor) = 12.4 min.

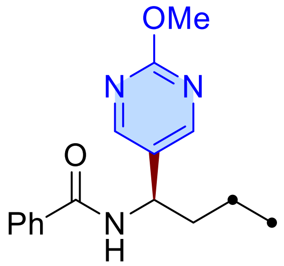

(*R*)-*N*-(1-(2-Methoxypyrimidin-5-yl)butyl)benzamide (Figure 2, **3o**). From 5-bromo-2-methoxypyrimidine (56.7 mg, 0.30 mmol, 1.5 equiv), the title compound was prepared following the general procedure **A** using $Ni(NO_3)_2{\cdot}6H_2O$ (2.9 mg, 5.0 mol%), **L1*** (5.8 mg, 6.0 mol%), **L1** [0.29 mg, 0.75 mL (11.8 mg/ 30 mL in toluene)], Na_2CO_3 (42.4 mg, 2.0 equiv), NaI (15.0 mg, 0.5 equiv), *N*-(but-3-en-1-yl)benzamide (35.0 mg, 0.20 mmol, 1.0 equiv), DMMS (74 µL, 0.60 mmol, 3.0 equiv) and anhydrous NMP (0.25 mL). The reaction mixture was stirred for 24 h at 25 °C. The crude material was purified by flash column chromatography (petroleum ether/EtOAc = 5:1) to provide the title compound as a white solid in 64% yield (36.2 mg) with >99:1 rr.

^{1}H NMR (500 MHz, $CDCl_3$) δ 8.50 (s, 2H), 7.75 – 7.70 (m, 2H), 7.51 – 7.44 (m, 1H), 7.42 – 7.35 (m, 2H), 6.67 – 6.62 (m, 1H), 5.09 (q, *J* = 7.6 Hz, 1H), 3.97 (s, 3H), 1.97 – 1.78 (m, 2H), 1.50 – 1.29 (m, 2H), 0.95 (t, *J* = 7.3 Hz, 3H);

^{13}C NMR (126 MHz, $CDCl_3$) δ 167.2, 165.1, 158.1, 134.1, 131.9, 129.2, 128.7, 127.1, 55.1, 49.4, 37.6, 19.6, 13.8;

HRMS (ESI) calcd. for $C_{16}H_{20}N_3O_2$ $[M+H]^+$ *m/z* 286.1550, found 286.1541;

IR (neat, cm^{-1}) 3309, 2957, 1479, 1311, 1032, 802, 694;

m.p. 132.9 – 133.1°C;

$[\alpha]_D^{25}$ = -4.7 (c = 0.80, $CHCl_3$); 94% *ee*;

HPLC analysis CHIRALCEL® OD-H column, 20% ^{i}PrOH in hexane, 0.8 mL/min, 254 nm UV detector, t_R (major) = 8.1 min, t_R (minor) = 10.4 min.

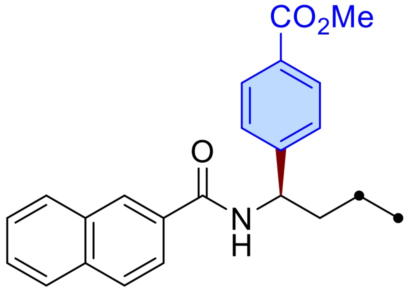

Methyl (*R*)-4-(1-(2-naphthamido)butyl)benzoate (Figure 2, **3p**). From methyl 4-bromobenzoate (64.5 mg, 0.30 mmol, 1.5 equiv), the title compound was prepared following the general procedure **A** using $Ni(NO_3)_2 \cdot 6H_2O$ (2.9 mg, 5.0 mol%), **L1*** (5.8 mg, 6.0 mol%), **L1** [0.29 mg, 0.75 mL (11.8 mg/ 30 mL in toluene)], Na_2CO_3 (42.4 mg, 2.0 equiv), NaI (15.0 mg, 0.5 equiv), *N*-(but-3-en-1-yl)-2-naphthamide (45.0 mg, 0.20 mmol, 1.0 equiv), DMMS (74 μL, 0.60 mmol, 3.0 equiv) and anhydrous NMP (0.25 mL). The reaction mixture was stirred for 24 h at 25 °C. The crude material was purified by flash column chromatography (petroleum ether/EtOAc = 5:1) to provide the title compound as a white solid in 60% yield (43.0 mg) with >99:1 rr.

^{1}H NMR (500 MHz, $CDCl_3$) δ 8.27 (s, 1H), 8.04 – 8.00 (m, 2H), 7.93 – 7.79 (m, 4H), 7.64 – 7.49 (m, 2H), 7.46 (d, *J* = 8.4 Hz, 2H), 6.61 (d, *J* = 7.9 Hz, 1H), 5.27 (q, *J* = 7.6 Hz, 1H), 3.90 (s, 3H), 2.01 – 1.81 (m, 2H), 1.51 – 1.34 (m, 2H), 0.97 (t, *J* = 7.3 Hz, 3H);

^{13}C NMR (126 MHz, $CDCl_3$) δ 167.1, 167.0, 147.9, 134.9, 132.7, 131.7, 130.2, 129.4, 129.0, 128.7, 127.9, 127.8, 127.5, 127.0, 126.8, 123.7, 53.8, 52.2, 38.5, 19.7, 14.0;

HRMS (ESI) calcd. for $C_{23}H_{23}NO_3Na$ $[M+Na]^+$ *m/z* 384.1570, found 384.1563;

IR (neat, cm^{-1}) 3302, 2956, 1721, 1279, 906, 731;

m.p. 148.5 – 150.2 °C;

$[\alpha]_D^{17}$ = –47.8 (c = 1.0, $CHCl_3$); 93% *ee*;

HPLC analysis CHIRALPAK® AD-H column, 30% ^{i}PrOH in hexane, 0.8 mL/min, 254 nm UV detector, t_R (major) = 8.8 min, t_R (minor) = 11.6 min.

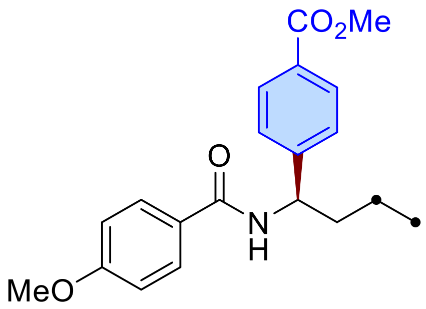

Methyl (*R*)-4-(1-(4-methoxybenzamido)butyl)benzoate (Figure 2, **3q**). From methyl 4-bromobenzoate (64.5 mg, 0.30 mmol, 1.5 equiv), the title compound was prepared following the general procedure **A** using $Ni(NO_3)_2 \cdot 6H_2O$ (2.9 mg, 5.0 mol%), **L1*** (5.8 mg, 6.0 mol%), **L1** [0.29 mg, 0.75 mL (11.8 mg/ 30 mL in toluene)], Na_2CO_3 (42.4 mg, 2.0 equiv), NaI (15.0 mg, 0.5 equiv), *N*-(but-3-en-1-yl)-4-methoxybenzamide (41.0 mg, 0.20 mmol, 1.0 equiv), DMMS (74 μL, 0.60 mmol, 3.0 equiv) and anhydrous NMP (0.25 mL). The reaction mixture was stirred for 24 h at 25 °C. The crude material was purified by flash column chromatography (petroleum ether/EtOAc = 5:1) to provide the title compound as a white solid in 59% yield (39.9 mg) with >99:1 rr.

^{1}H NMR (500 MHz, $CDCl_3$) δ 7.98 (d, *J* = 8.4 Hz, 2H), 7.73 (d, *J* = 8.8 Hz, 2H), 7.40 (d, *J* = 8.4 Hz, 2H), 6.88 (d, *J* = 8.9 Hz, 2H), 6.52 (d, *J* = 8.0 Hz, 1H), 5.17 (q, *J* = 7.6 Hz, 1H), 3.89 (s, 3H), 3.82 (s, 3H), 1.95 – 1.75 (m, 2H), 1.47 – 1.27 (m, 2H), 0.93 (t, *J* = 7.3 Hz, 3H);

^{13}C NMR (126 MHz, $CDCl_3$) δ 167.0, 166.5, 162.4, 148.2, 130.1, 129.2, 128.9, 126.7, 126.6, 113.9, 55.5, 53.6, 52.2, 38.5, 19.6, 13.9;

HRMS (ESI) calcd. for $C_{20}H_{23}NO_4Na$ $[M+Na]^+$ *m/z* 364.1519, found 364.1517;

IR (neat, cm^{-1}) 3310, 3019, 1214, 744;

m.p. 193.0 – 194.3 °C;

$[\alpha]_D^{17}$ = –25.8 (c = 1.07, $CHCl_3$); 93% *ee*;

HPLC analysis CHIRALPAK® AD-H column, 30% ^{i}PrOH in hexane, 0.8 mL/min, 254 nm UV detector, t_R (major) = 10.8 min, t_R (minor) = 17.6 min.

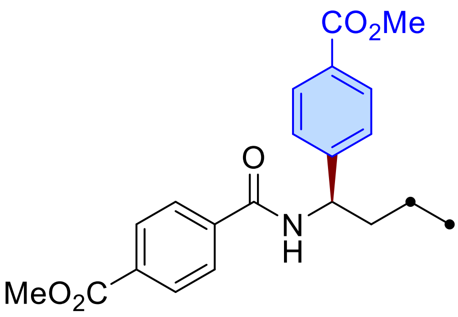

Methyl (*R*)-4-(1-(4-(methoxycarbonyl)benzamido)butyl)benzoate (Figure 2, **3r**). From methyl 4-bromobenzoate (64.5 mg, 0.30 mmol, 1.5 equiv), the title compound was prepared following the general procedure **A** using $Ni(NO_3)_2 \cdot 6H_2O$ (2.9 mg, 5.0 mol%), **L1*** (5.8 mg, 6.0 mol%), **L1** [0.29 mg, 0.75 mL (11.8 mg/ 30 mL in toluene)], Na_2CO_3 (42.4 mg, 2.0 equiv), NaI (15.0 mg, 0.5 equiv), methyl 4-(but-3-en-1-ylcarbamoyl)benzoate (46.6mg, 0.20 mmol, 1.0 equiv), DMMS (74 µL, 0.60 mmol, 3.0 equiv) and anhydrous NMP (0.25 mL). The reaction mixture was stirred for 24 h at 25 °C. The crude material was purified by flash column chromatography (petroleum ether/EtOAc = 5:1) to provide the title compound as a white solid in 56% yield (41.6 mg) with >99:1 rr.

^{1}H NMR (500 MHz, $CDCl_3$) δ 8.04 – 8.01 (m, 2H), 8.00 – 7.96 (m, 2H), 7.81 – 7.78 (m, 2H), 7.42 – 7.38 (m, 2H), 6.73 (d, *J* = 7.9 Hz, 1H), 5.18 (q, *J* = 7.6 Hz, 1H), 3.92 (s, 3H), 3.89 (s, 3H), 2.05 – 1.77 (m, 2H), 1.44 – 1.30 (m, 2H), 0.94 (t, *J* = 7.3 Hz, 3H);

^{13}C NMR (126 MHz, $CDCl_3$) δ 166.9, 166.4, 166.1, 147.5, 138.4, 133.0, 130.2, 130.0, 129.5, 127.1, 126.7, 53.9, 52.6, 52.3, 38.4, 19.7, 13.9;

HRMS (ESI) calcd. for $C_{21}H_{23}NO_5Na$ $[M+Na]^+$ *m/z* 392.1468, found 392.1466;

IR (neat, cm^{-1}) 3019, 1720, 1214, 744;

m.p. 178.3 – 179.9 °C;

$[\alpha]_D^{17}$ = –19.7 (c = 1.22, $CHCl_3$); 87% *ee*;

HPLC analysis CHIRALCEL® OD-H column, 20% ^{i}PrOH in hexane, 0.8 mL/min, 254 nm UV detector, t_R (minor) = 12.1 min, t_R (major) = 15.6 min.

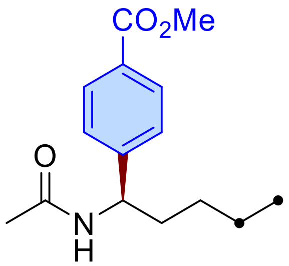

Methyl (*R*)-4-(1-acetamidopentyl)benzoate (Figure 2, **3s**). From methyl 4-bromobenzoate (64.5 mg, 0.30 mmol, 1.5 equiv), the title compound was prepared following the general procedure **A** using $Ni(NO_3)_2·6H_2O$ (2.9 mg, 5.0 mol%), **L1*** (5.8 mg, 6.0 mol%), **L1** [0.29 mg, 0.75 mL (11.8 mg/ 30 mL in toluene)], Na_2CO_3 (42.4 mg, 2.0 equiv), NaI (15.0 mg, 0.5 equiv), *N*-(pent-4-en-1-yl)acetamide (25.4 mg, 0.20 mmol, 1.0 equiv), DMMS (74 μL, 0.60 mmol, 3.0 equiv) and anhydrous NMP (0.25 mL). The reaction mixture was stirred for 24 h at 25 °C. The crude material was purified by flash column chromatography (petroleum ether/EtOAc = 5:1) to provide the title compound as a white solid in 50% yield (26.5 mg) with >99:1 rr.

^{1}H NMR (500 MHz, $CDCl_3$) δ 8.02 – 7.97 (m, 2H), 7.36 – 7.31 (m, 2H), 5.76 (d, *J* = 8.2 Hz, 1H), 4.98 (q, *J* = 7.6 Hz, 1H), 3.90 (s, 3H), 2.00 (s, 3H), 1.80 – 1.71 (m, 2H), 1.38 – 1.24 (m, 2H), 1.24 – 1.09 (m, 2H), 0.86 (t, *J* = 7.1 Hz, 3H);

^{13}C NMR (126 MHz, $CDCl_3$) δ 169.4, 167.0, 147.9, 130.1, 129.3, 126.7, 53.5, 52.2, 36.0, 28.4, 23.6, 22.6, 14.0;

HRMS (ESI) calcd. for $C_{15}H_{21}NO_3Na$ $[M+Na]^+$ *m/z* 286.1413, found 286.1409;

IR (neat, cm^{-1}) 3020, 1722, 1214, 748;

m.p. 121.1 – 122.0 °C;

$[α]_D^{25}$ = +96.6 (c = 0.21, $CHCl_3$); 92% *ee*;

HPLC analysis CHIRALPAK® AD-H column, 10% ^{i}PrOH in hexane, 0.8 mL/min, 254 nm UV detector, t_R (major) = 10.0 min, t_R (minor) = 12.1 min.

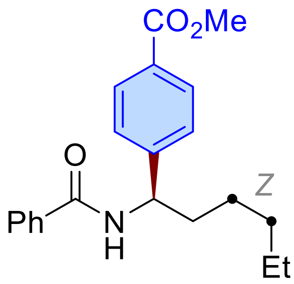

Methyl (*R*)-4-(1-benzamidohexyl)benzoate (Figure 2, **3t**). From methyl 4-bromobenzoate (64.5 mg, 0.30 mmol, 1.5 equiv), the title compound was prepared following the general procedure **A** using $Ni(NO_3)_2 \cdot 6H_2O$ (2.9 mg, 5.0 mol%), **L1*** (5.8 mg, 6.0 mol%), **L1** [0.29 mg, 0.75 mL (11.8 mg/ 30 mL in toluene)], Na_2CO_3 (42.4 mg, 2.0 equiv), NaI (15.0 mg, 0.5 equiv), (*Z*)-*N*-(hex-3-en-1-yl)benzamide (40.6 mg, 0.20 mmol, 1.0 equiv), DMMS (74 μL, 0.60 mmol, 3.0 equiv) and anhydrous NMP (0.25 mL). The reaction mixture was stirred for 24 h at 25 °C. The crude material was purified by flash column chromatography (petroleum ether/EtOAc = 5:1) to provide the title compound as a white solid in 51% yield (34.8 mg) with >99:1 rr.

^{1}H NMR (500 MHz, $CDCl_3$) δ 8.00 – 7.96 (m, 2H), 7.85 – 7.71 (m, 2H), 7.55 – 7.43 (m, 1H), 7.43 – 7.34 (m, 4H), 6.64 (d, *J* = 8.0 Hz, 1H), 5.17 (q, *J* = 7.6 Hz, 1H), 3.89 (s, 3H), 1.97 – 1.78 (m, 2H), 1.39 – 1.22 (m, 6H), 0.87 – 0.81 (m, 3H);

^{13}C NMR (126 MHz, $CDCl_3$) δ 167.0, 166.9, 147.9, 134.5, 131.8, 130.2, 129.3, 128.8, 127.1, 126.7, 53.9, 52.2, 36.3, 31.6, 26.0, 22.6, 14.1;

HRMS (ESI) calcd. for $C_{21}H_{25}NO_3Na$ $[M+Na]^+$ *m/z* 362.1726, found 362.1723;

IR (neat, cm^{-1}) 3287, 2929, 1636,1214, 748;

m.p. 131.1 – 132.1 °C;

$[\alpha]_D^{17}$ = +0.7 (c = 0.81, $CHCl_3$); 91% *ee*;

HPLC analysis CHIRALPAK® AD-H column, 20% ^{i}PrOH in hexane, 0.8 mL/min, 254 nm UV detector, t_R (major) = 10.9 min, t_R (minor) = 16.1 min.

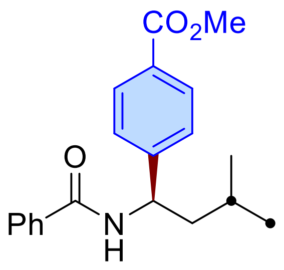

Methyl (*R*)-4-(1-benzamido-3-methylbutyl)benzoate (Figure 2, **3u**). From methyl 4-bromobenzoate (64.5 mg, 0.30 mmol, 1.5 equiv), the title compound was prepared following the general procedure A using $Ni(NO_3)_2 \cdot 6H_2O$ (2.9 mg, 5.0 mol%), **L*** (5.8 mg, 6.0 mol%), **L** [0.29 mg, 0.75 mL (11.8 mg/ 30 mL in toluene)], Na_2CO_3 (42.4 mg, 2.0 equiv), NaI (15.0 mg, 0.5 equiv), *N*-(3-methylbut-3-en-1-yl)benzamide (37.8 mg,

0.20 mmol, 1.0 equiv), DMMS (74 μL, 0.60 mmol, 3.0 equiv) and anhydrous NMP (0.25 mL). The reaction mixture was stirred for 24 h at rt (22~26 °C). The crude material was purified by flash column chromatography (petroleum ether/EtOAc = 5:1) to provide the title compound as a white solid in 51% yield (33.2 mg) with >99:1 rr.

^{1}H NMR (500 MHz, $CDCl_3$) δ 8.02 – 7.97 (m, 2H), 7.79 – 7.73 (m, 2H), 7.52 – 7.45 (m, 1H), 7.44 – 7.37 (m, 4H), 6.48 (d, J = 8.0 Hz, 1H), 5.32 – 5.24 (m, 1H), 3.89 (s, 3H), 1.86 – 1.76 (m, 1H), 1.75 – 1.66 (m, 1H), 1.66 – 1.56 (m, 1H), 1.00 – 0.94 (m, 6H);

^{13}C NMR (126 MHz, $CDCl_3$) δ 167.0, 166. 9, 148.3, 134.5, 131.7, 130.2, 129.3, 128.7, 127.1, 126.7, 52.2, 52.1, 45.6, 25.3, 22.9, 22.5;

HRMS (ESI) calcd. for $C_{20}H_{23}NO_3Na$ $[M+Na]^+$ m/z 348.1570, found 348.1569;

IR (neat, cm^{-1}) 3293, 2922, 1214, 749;

$[\alpha]_D^{17}$ = +2.6 (c = 0.62, $CHCl_3$); 97% *ee*;

HPLC analysis CHIRALPAK® AD-H column, 20% ^{i}PrOH in hexane, 0.8 mL/min, 254 nm UV detector, t_R (major) = 9.7 min, t_R (minor) = 14.7 min.

3. Asymmetric Migratory Hydroarylation to Access Enantioenriched *α*-(Hetero)Aryl-Substituted *N*-heterocycles

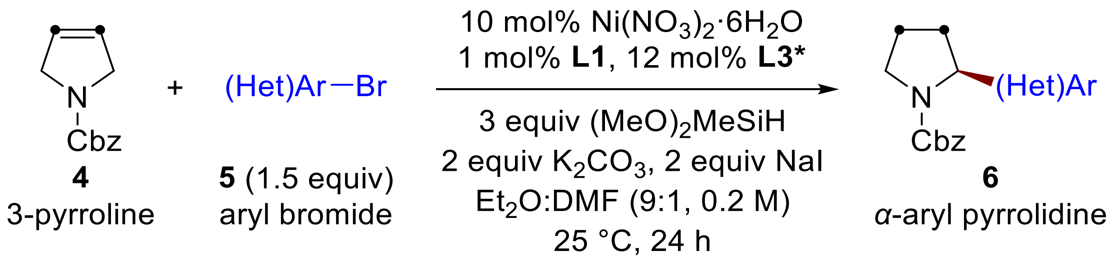

General procedure (B) for the asymmetric migratory hydroarylation of *N*-carbamate protected heterocyclic alkenes. In a nitrogen-filled glove box, to an oven-dried 8 mL screw-cap vial equipped with a magnetic stir bar was added $Ni(NO_3)_2·6H_2O$ (5.8 mg, 10.0 mol%), **L3*** (14.4 mg, 12.0 mol%), K_2CO_3 (55.4 mg, 2.0 equiv), NaI (60.0 mg, 2.0 equiv), **L1** [0.58 mg, 0.9 mL stock solution (6.5 mg/ 10 mL in ether)] and anhydrous DMF (0.1 mL). The mixture was stirred for 5 min at room temperature, at

which time benzyl 2,5-dihydro-1*H*-pyrrole-1-carboxylate (40.6 mg, 0.20 mmol, 1.0 equiv), aryl or hetero bromide (0.30 mmol, 1.5 equiv) and DMMS (74 μL, 0.60 mmol, 3.0 equiv) were added to the resulting mixture in this order. The tube was sealed with a teflon-lined screw cap, removed from the glove box and the reaction was stirred at 25 °C for up to 24 h (the mixture was stirred at 800 rpm, ensuring that the base was uniformly suspended). After the reaction was complete, the reaction mixture was directly filtered through a short pad of silica gel [EtOAc in petroleum ether (PE)] to give the crude product. *n*-Dodecane (20 μL) was added as an internal standard for GC analysis. The product was purified by chromatography on silica gel for each substrate. The yields reported are the average of at least two experiments, unless otherwise indicated. The enantiomeric excesses (% *ee*) were determined by HPLC analysis using chiral stationary phases.

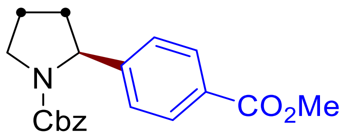

Benzyl (*S*)-2-(4-(methoxycarbonyl)phenyl)pyrrolidine-1-carboxylate (Figure 3, **6a**). From methyl 4-bromobenzoate (64.5 mg, 0.30 mmol, 1.5 equiv), the title compound was prepared following the general procedure **B** using $Ni(NO_3)_2·6H_2O$ (2.9 mg, 5.0 mol%), **L3*** (7.2 mg, 6.0 mol%), **L1** [0.29 mg, 0.9 mL (3.2 mg/ 10 mL in DME)], K_2CO_3 (55.4 mg, 2.0 equiv), NaI (60.0 mg, 2.0 equiv), benzyl 2,5-dihydro-1*H*-pyrrole-1-carboxylate (40.6 mg, 0.20 mmol, 1.0 equiv), DMMS (74 μL, 0.60 mmol, 3.0 equiv) and anhydrous DMA (0.1 mL). The reaction mixture was stirred for 24 h at 25 °C. The crude material was purified by flash column chromatography (petroleum ether/EtOAc = 5:1) to provide the title compound as yellow oil in 67% yield (45.5 mg) with >99:1 rr.

^{1}H NMR (500 MHz, $CDCl_3$) δ 8.01 – 7.93 (m, 2H), 7.44 – 7.10 (m, 6H), 6.88 (d, *J* = 7.3 Hz, 1H), 5.20 – 4.87 (m, 3H), 3.96 – 3.85 (m, 3H), 3.77 – 3.59 (m, 2H), 2.43 – 2.27 (m, 1H), 1.97 – 1.77 (m, 3H);

^{13}C NMR (126 MHz, $CDCl_3$) δ 167.0, 155.0, 149.8 & 149.0, 137.0 & 136.5, 129.9, 128.8, 128.5 & 128.3, 128.1 & 128.0, 127.7 & 127.5, 125.6, 67.0 & 66.8, 61.3 & 61.1, 52.2 & 52.1, 47.8 & 47.3, 35.9 & 34.8, 23.8 & 23.1;

HRMS (ESI) calcd. for $C_{20}H_{21}NO_4Na$ $[M+Na]^+$ *m/z* 362.1363, found 362.1366;

IR (neat, cm^{-1}) 2951, 1699, 1408, 1276, 1105, 770, 699;

$[\alpha]_D^{25}$ = –81.9 (c = 1.04, $CHCl_3$); 90% *ee*;

HPLC analysis CHIRALCEL® OD-H column, 20% ^{i}PrOH in hexane, 0.8 mL/min, 254 nm UV detector, t_R (major) = 10.2 min, t_R (minor) = 12.2 min.

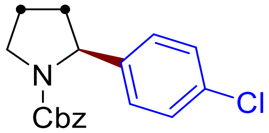

Benzyl (*S*)-2-(4-chlorophenyl)pyrrolidine-1-carboxylate (Figure 3, **6b**). From 1-bromo-4-chlorobenzene (57.0 mg, 0.30 mmol, 1.5 equiv), the title compound was prepared following the general procedure **B** using $Ni(NO_3)_2{\cdot}6H_2O$ (2.9 mg, 5.0 mol%), **L3*** (7.2 mg, 6.0 mol%), **L1** [0.29 mg, 0.9 mL (3.2 mg/ 10 mL in DME)], K_2CO_3 (55.4 mg, 2.0 equiv), NaI (60.0 mg, 2.0 equiv), benzyl 2,5-dihydro-1*H*-pyrrole-1-carboxylate (40.6 mg, 0.20 mmol, 1.0 equiv), DEMS (96 μL, 0.60 mmol, 3.0 equiv) and anhydrous DMA (0.1 mL). The reaction mixture was stirred for 24 h at 25 °C. The crude material was purified by flash column chromatography (petroleum ether/EtOAc = 5:1) to provide the title compound as yellow oil in 79% yield (49.7 mg) with >99:1 rr.

^{1}H NMR (500 MHz, $CDCl_3$) δ 7.52 – 7.01 (m, 8H), 6.91 (d, *J* = 5.8 Hz, 1H), 5.21 – 4.82 (m, 3H), 3.74 – 3.56 (m, 2H), 2.39 – 2.24 (m, 1H), 1.99 – 1.76 (m, 3H);

^{13}C NMR (126 MHz, $CDCl_3$) δ 155.0, 143.0 & 142.2, 137.0 & 136.7, 132.5, 128.6, 128.3 & 128.1, 127.8, 127.5, 127.1, 67.0 & 66.8, 61.0 & 60.7, 47.8 & 47.3, 36.0 & 34.9, 23.8 & 23.1;

HRMS (ESI) calcd. for $C_{20}H_{21}NO_4Na$ $[M+H]^+$ *m/z* 316.1099, found 316.1092;

IR (neat, cm^{-1}) 2952, 1697, 1406, 1089, 820, 696;

$[\alpha]_D^{25}$ = –46.5 (c = 1.44, $CHCl_3$); 88% *ee*;

HPLC analysis CHIRALCEL® OD-H column, 20% ^{i}PrOH in hexane, 0.8 mL/min, 254 nm UV detector, t_R (major) = 7.6 min, t_R (minor) = 9.1 min.

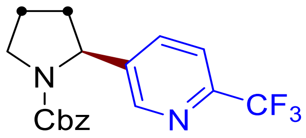

Benzyl (*S*)-2-(6-(trifluoromethyl)pyridin-3-yl)pyrrolidine-1-carboxylate (Figure 3, **6c**). From 5-bromo-2-(trifluoromethyl)pyridine (67.8 mg, 0.30 mmol, 1.5 equiv), the title compound was prepared following the general procedure **B** using $Ni(NO_3)_2{\cdot}6H_2O$ (5.8 mg, 10.0 mol%), **L3*** (14.4 mg, 12.0 mol%), **L1** [0.58 mg, 0.9 mL (6.5 mg/ 10 mL in ether)], K_2CO_3 (55.4 mg, 2.0 equiv), NaI (60.0 mg, 2.0 equiv), benzyl 2,5-dihydro-1*H*-pyrrole-1-carboxylate (40.6 mg, 0.20 mmol, 1.0 equiv), DMMS (74 μL, 0.60 mmol, 3.0 equiv) and anhydrous DMF (0.1 mL). The reaction mixture was stirred for 24 h at 25 °C. The crude material was purified by flash column chromatography (petroleum ether/EtOAc = 2:1) to provide the title compound as yellow oil in 79% yield (55.4 mg) with >99:1 rr.

^{1}H NMR (500 MHz, $CDCl_3$) δ 8.64 – 8.49 (m, 1H), 7.71 – 7.49 (m, 2H), 7.41 – 7.14 (m, 4H), 6.91 (d, J = 7.2 Hz, 1H), 5.22 – 4.84 (m, 3H), 3.76 – 3.66 (m, 2H), 2.46 – 2.38 (m, 1H), 2.01 – 1.81 (m, 3H);

^{13}C NMR (126 MHz, $CDCl_3$) δ 155.1 & 154.7, 148.1, 146.9 (q, J = 34.8 Hz), 143.2 & 142.4, 136.7 & 136.2, 134.7 & 134.4, 128.7 & 128.5, 128.3 & 128.2, 128.1 & 127.9, 121.7 (q, J = 273.9 Hz), 120.3, 67.3 & 67.2, 59.3 & 58.9, 47.9 & 47.4, 35.9 & 34.6, 23.9 & 23.3;

^{19}F NMR (471 MHz, $CDCl_3$) δ –67.7, –67.8;

HRMS (ESI) calcd. for $C_{18}H_{18}F_3N_2O_2$ $[M+H]^+$ *m/z* 351.1315, found 351.1310;

IR (neat, cm^{-1}) 2955, 1698, 1337, 1131, 1086, 698;

$[\alpha]_D^{25}$ = –44.6 (c = 1.93, $CHCl_3$); 88% *ee*;

HPLC analysis CHIRALCEL® OD-H column, 20% ^{i}PrOH in hexane, 0.8 mL/min, 254 nm UV detector, t_R (major) = 9.5 min, t_R (minor) = 12.6 min.

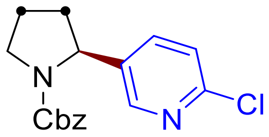

Benzyl (*S*)-2-(6-chloropyridin-3-yl)pyrrolidine-1-carboxylate (Figure 3, **6d**). From 5-bromo-2-chloropyridine (57.7 mg, 0.30 mmol, 1.5 equiv), the title compound was prepared following the general procedure **B** using $Ni(NO_3)_2\cdot6H_2O$ (5.8 mg, 10.0 mol%), **L3*** (14.4 mg, 12.0 mol%), **L1** [0.58 mg, 0.9 mL (6.5 mg/ 10 mL in ether)], K_2CO_3 (55.4 mg, 2.0 equiv), NaI (60.0 mg, 2.0 equiv), benzyl 2,5-dihydro-1*H*-pyrrole-1-carboxylate (40.6 mg, 0.20 mmol, 1.0 equiv), DEMS (96 μL, 0.60 mmol, 3.0 equiv) and anhydrous DMF (0.1 mL). The reaction mixture was stirred for 24 h at 25 °C. The crude material was purified by flash column chromatography (petroleum ether/EtOAc = 2:1) to provide the title compound as yellow oil in 75% yield (47.3 mg) with >99:1 rr.

^{1}H NMR (500 MHz, $CDCl_3$) δ 8.31 – 8.17 (m, 1H), 7.52 – 7.11 (m, 6H), 6.96 (s, 1H), 5.20 – 4.82 (m, 3H), 3.75 – 3.57 (m, 2H), 2.43 – 2.29 (m, 1H), 1.98 – 1.77 (m, 3H);

^{13}C NMR (126 MHz, $CDCl_3$) δ 155.0 & 154.8, 149.9, 147.6, 138.8 & 138.0, 136.7 & 136.4, 136.2 & 136.1, 128.6 & 128.4, 128.1 & 128.0, 127.8, 124.1, 67.1, 58.8 & 58.5, 47.7 & 47.2, 35.8 & 34.6, 23.9 & 23.2;

HRMS (ESI) calcd. for $C_{17}H_{17}ClN_2O_2Na$ $[M+Na]^+$ *m/z* 339.0871, found 339.0864;

IR (neat, cm^{-1}) 2954, 2879, 1700, 1409, 1104, 698;

$[\alpha]_D^{25}$ = –69.5 (c = 1.09, $CHCl_3$); 92% *ee*;

HPLC analysis CHIRALCEL® OD-H column, 20% ^{i}PrOH in hexane, 0.8 mL/min, 254 nm UV detector, t_R (major) = 11.9 min, t_R (minor) = 13.8 min.

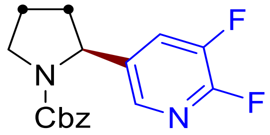

Benzyl (*S*)-2-(5,6-difluoropyridin-3-yl)pyrrolidine-1-carboxylate (Figure 3, **6e**). From 5-bromo-2,3-difluoropyridine (58.2 mg, 0.30 mmol, 1.5 equiv), the title compound was prepared following the general procedure **B** using $Ni(NO_3)_2\cdot6H_2O$ (5.8 mg, 10.0 mol%), **L3*** (14.4 mg, 12.0 mol%), **L1** [0.58 mg, 0.9 mL (6.5 mg/ 10 mL in ether)], K_2CO_3 (55.4 mg, 2.0 equiv), NaI (60.0 mg, 2.0 equiv), benzyl 2,5-dihydro-1*H*-pyrrole-1-carboxylate (40.6 mg, 0.20 mmol, 1.0 equiv), DEMS (96 μL, 0.60 mmol, 3.0 equiv) and anhydrous DMF (0.1 mL). The reaction mixture was stirred for 24 h at 25 °C.

The crude material was purified by flash column chromatography (petroleum ether/EtOAc = 2:1) to provide the title compound as yellow oil in 89% yield (56.7 mg) with >99:1 rr.

^{1}H NMR (500 MHz, $CDCl_3$) δ 7.90 – 7.74 (m, 1H), 7.45 – 7.19 (m, 5H), 7.01 (d, *J* = 6.8 Hz, 1H), 5.20 – 4.80 (m, 3H), 3.75 – 3.57 (m, 2H), 2.47 – 2.28 (m, 1H), 2.05 – 1.79 (m, 3H);

^{13}C NMR (126 MHz, $CDCl_3$) δ 155.1 & 154.7, 151.1 (d, *J* = 236.9 Hz), 145.4 (d, *J* = 260.8 Hz), 139.8, 138.9, 136.6 & 136.2, 128.6 & 128.5, 128.2, 128.1 & 128.0, 124.2 (d, *J* = 41.6 Hz), 67.3, 58.5 & 58.1, 47.7 & 47.3, 35.8 & 34.6, 23.9 & 23.3;

^{19}F NMR (471 MHz, $CDCl_3$) δ –90.0, –90.1, –90.2, –90.3, –139.6, –139.7, –140.0, –140.1;

HRMS (ESI) calcd. for $C_{17}H_{17}F_2N_2O_2$ $[M+H]^+$ *m/z* 313.1547, found 313.1548;

IR (neat, cm^{-1}) 2948, 1698, 1395, 1103, 1042, 697;

$[\alpha]_D^{25}$ = –66.1 (c = 1.08, $CHCl_3$); 91% *ee*;

HPLC analysis CHIRALCEL® OD-H column, 20% ^{i}PrOH in hexane, 0.8 mL/min, 254 nm UV detector, t_R (major) = 9.5 min, t_R (minor) = 11.1 min.

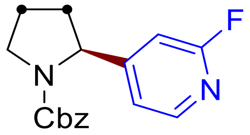

Benzyl (*S*)-2-(2-fluoropyridin-4-yl)pyrrolidine-1-carboxylate (Figure 3, **6f**). From 4-bromo-2-fluoropyridine (52.8 mg, 0.30 mmol, 1.5 equiv), the title compound was prepared following the general procedure **B** using $Ni(NO_3)_2 \cdot 6H_2O$ (5.8 mg, 10.0 mol%), **L3*** (14.4 mg, 12.0 mol%), **L1** [0.58 mg, 0.9 mL (6.5 mg/ 10 mL in ether)], K_2CO_3 (55.4 mg, 2.0 equiv), NaI (60.0 mg, 2.0 equiv), benzyl 2,5-dihydro-1*H*-pyrrole-1-carboxylate (40.6 mg, 0.20 mmol, 1.0 equiv), DMMS (74 μL, 0.60 mmol, 3.0 equiv) and anhydrous DMF (0.1 mL). The reaction mixture was stirred for 24 h at 25 °C. The crude material was purified by flash column chromatography (petroleum ether/EtOAc = 2:1) to provide the title compound as yellow oil in 89% yield (53.7 mg) with >99:1 rr.

^{1}H NMR (500 MHz, $CDCl_3$) δ 8.14 – 8.06 (m, 1H), 7.47 – 7.14 (m, 4H), 7.07 – 6.90 (m, 2H), 6.70 (d, *J* = 27.8 Hz, 1H), 5.23 – 4.81 (m, 3H), 3.87 – 3.50 (m, 2H), 2.48 – 2.27 (m, 1H), 2.02 – 1.73 (m, 3H);

^{13}C NMR (126 MHz, $CDCl_3$) δ 164.2 (d, *J* = 239.3 Hz), 159.2 (d, *J* = 98.5 Hz), 155.0 & 154.7, 147.8 & 147.7, 136.6 & 136.2, 128.6 & 128.4, 128.2 & 128.1, 127.7, 118.6 (d, *J* = 21.5 Hz), 106.6 & 106.3, 67.2 & 67.1, 60.4 & 60.1, 47.7 & 47.3, 35.3 & 34.2, 23.8 & 23.1;

^{19}F NMR (471 MHz, $CDCl_3$) δ –68.1, –68.3;

HRMS (ESI) calcd. for $C_{17}H_{17}FN_2O_2Na$ $[M+Na]^+$ *m/z* 323.1166, found 323.1166;

IR (neat, cm^{-1}) 2953, 1700, 1409, 1107, 772, 735;

$[\alpha]_D^{25}$ = –55.8 (c = 1.06, $CHCl_3$); 88% *ee*;

HPLC analysis CHIRALCEL® OD-H column, 30% ^{i}PrOH in hexane, 0.8 mL/min, 254 nm UV detector, t_R (major) = 8.5 min, t_R (minor) = 12.2 min.

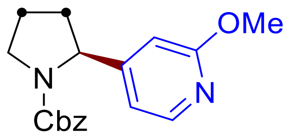

Benzyl (*S*)-2-(2-methoxypyridin-4-yl)pyrrolidine-1-carboxylate (Figure 3, **6g**). From 4-bromo-2-methoxypyridine (56.4 mg, 0.30 mmol, 1.5 equiv), the title compound was prepared following the general procedure **B** using $Ni(NO_3)_2·6H_2O$ (5.8 mg, 10.0 mol%), **L3*** (14.4 mg, 12.0 mol%), **L1** [0.58 mg, 0.9 mL (6.5 mg/ 10 mL in ether)], K_2CO_3 (55.4 mg, 2.0 equiv), NaI (60.0 mg, 2.0 equiv), benzyl 2,5-dihydro-1*H*-pyrrole-1-carboxylate (40.6 mg, 0.20 mmol, 1.0 equiv), DMMS (74 µL, 0.60 mmol, 3.0 equiv) and anhydrous DMF (0.1 mL). The reaction mixture was stirred for 24 h at 25 °C. The crude material was purified by flash column chromatography (petroleum ether/EtOAc = 2:1) to provide the title compound as yellow oil in 80% yield (50.1 mg) with >99:1 rr.

^{1}H NMR (500 MHz, $CDCl_3$) δ 8.07 (dd, *J* = 11.5, 5.3 Hz, 1H), 7.43 – 7.15 (m, 4H), 7.01 – 6.94 (m, 1H), 6.68 (dd, *J* = 23.4, 5.4 Hz, 1H), 6.53 (d, *J* = 21.9 Hz, 1H), 5.23 – 4.78 (m, 3H), 3.91 (s, 3H), 3.71 – 3.54 (m, 2H), 2.38 – 2.24 (m, 1H), 1.94 – 1.78 (m, 3H);

^{13}C NMR (126 MHz, $CDCl_3$) δ 164.6, 156.2 & 155.5, 155.0 & 154.9, 147.0, 136.9 & 136.6, 128.6 & 128.3, 128.1 & 128.0, 127.8 & 127.6, 114.5 & 114.3, 107.5, 67.1 & 66.9, 60.5 & 60.2, 53.5, 47.7 & 47.2, 35.2 & 34.1, 23.7 & 23.0;

HRMS (ESI) calcd. for $C_{18}H_{21}N_2O_3$ $[M+H]^+$ *m/z* 311.1754, found 311.1752;

IR (neat, cm^{-1}) 2952, 1700, 1409, 1355, 1110, 698;

$[\alpha]_D^{25}$ = –71.7 (c = 1.72, $CHCl_3$); 90% *ee*;

HPLC analysis CHIRALCEL® OD-H column, 20% ^{i}PrOH in hexane, 0.8 mL/min, 254 nm UV detector, t_R (major) = 8.7 min, t_R (minor) = 10.9 min.

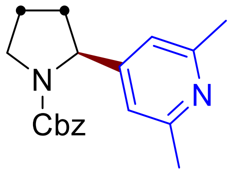

Benzyl (*S*)-2-(2,6-dimethylpyridin-4-yl)pyrrolidine-1-carboxylate (Figure 3, **6h**). From 4-bromo-2,6-dimethylpyridine (55.8 mg, 0.30 mmol, 1.5 equiv), the title compound was prepared following the general procedure **B** using $Ni(NO_3)_2·6H_2O$ (5.8 mg, 10.0 mol%), **L3*** (14.4 mg, 12.0 mol%), **L1** [0.58 mg, 0.9 mL (6.5 mg/ 10 mL in ether)], K_2CO_3 (55.4 mg, 2.0 equiv), NaI (60.0 mg, 2.0 equiv), benzyl 2,5-dihydro-1*H*-pyrrole-1-carboxylate (40.6 mg, 0.20 mmol, 1.0 equiv), DEMS (74 µL, 0.60 mmol, 3.0 equiv) and anhydrous DMF (0.1 mL). The reaction mixture was stirred for 24 h at 25 °C. The crude material was purified by flash column chromatography (petroleum ether/EtOAc = 2:1) to provide the title compound as yellow oil in 81% yield (50.0 mg) with >99:1 rr.

^{1}H NMR (500 MHz, $CDCl_3$) δ 7.41 – 7.13 (m, 4H), 6.93 (d, *J* = 5.4 Hz, 1H), 6.79 – 6.67 (m, 2H), 5.18 – 4.71 (m, 3H), 3.74 – 3.55 (m, 2H), 2.49 – 2.41 (m, 6H), 2.38 – 2.23 (m, 1H), 1.93 – 1.75 (m, 3H);

^{13}C NMR (126 MHz, $CDCl_3$) δ 157.9, 154.9, 153.9, 153.2, 136.9 & 136.5, 128.6 & 128.3, 128.1 & 128.0, 127.8 & 127.6, 118.2, 117.4, 67.0 & 66.9, 60.6 & 60.3, 47.7 & 47.3, 35.4 & 34.4, 24.6 & 24.5, 23.8 & 23.1;

HRMS (ESI) calcd. for $C_{19}H_{22}N_2O_2Na$ $[M+Na]^+$ *m/z* 341.1072, found 341.1069;

IR (neat, cm^{-1}) 2954, 1700, 1409, 1351, 1109, 750;

[α]$_D^{25}$ = –44.2 (c = 1.79, $CHCl_3$); 89% *ee*;

HPLC analysis CHIRALPAK® AD-H column, 20% ^{i}PrOH in hexane, 0.8 mL/min, 254 nm UV detector, t_R (minor) = 7.7 min, t_R (major) = 8.9 min.

4. Competition Experiment: Remote Alkene vs *Ipso*- Alkene

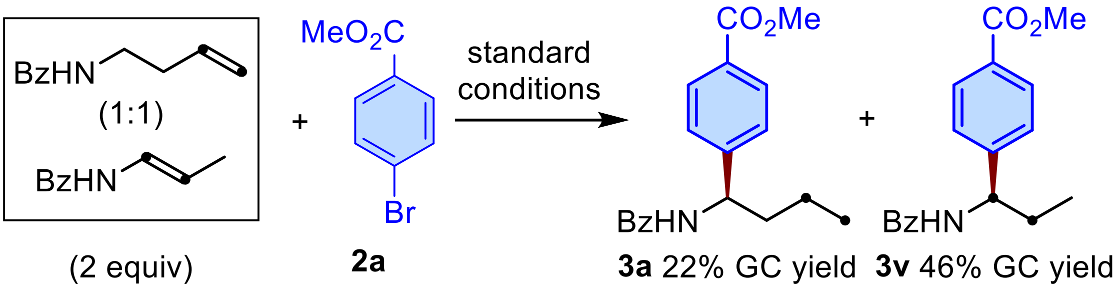

In a nitrogen-filled glove box, to an oven-dried 8 mL screw-cap vial equipped with a magnetic stir bar was added $Ni(NO_3)_2{\cdot}6H_2O$ (2.9 mg, 5.0 mol%), **L1*** (5.8 mg, 6.0 mol%), Na_2CO_3 (42.4 mg, 2.0 equiv), NaI (15.0 mg, 0.5 equiv), **L1** [0.29 mg, 0.75 mL stock solution (11.8 mg/ 30 mL in toluene)] and anhydrous NMP (0.25 mL). The mixture was stirred for 5 min at room temperature, at which time *N*-(but-3-en-1-yl)benzamide (35.0 mg, 0.20 mmol, 1.0 equiv), (*E*)-*N*-(prop-1-en-1-yl)benzamide (32.2 mg, 0.20 mmol, 1.0 equiv), methyl 4-bromobenzoate (43 mg, 0.2 mmol, 1.0 equiv) and DMMS (74 μL, 0.60 mmol, 3.0 equiv) were added to the resulting mixture in this order. The tube was sealed with a teflon-lined screw cap, removed from the glove box and the reaction was stirred at 25 °C for up to 24 h (the mixture was stirred at 800 rpm, ensuring that the base was uniformly suspended). The reaction was quenched upon the addition of H_2O and the mixture was extracted with ethylacetate. The organic layer was concentrated to give the crude product. *n*-Dodecane (20 μL) was added as an internal standard for GC analysis. Detected by GC, **3a** was obtained in 22% GC yield while **3v** was obtained in 46% GC yield.

5. *Ipso*-Control Experiments

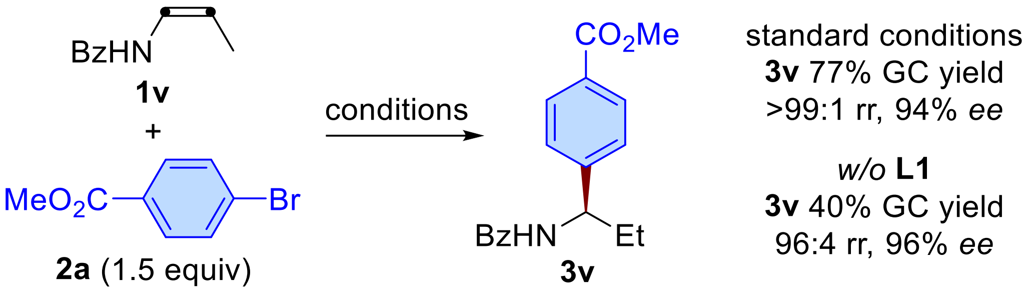

In a nitrogen-filled glove box, to an oven-dried 8 mL screw-cap vial equipped with a magnetic stir bar was added $Ni(NO_3)_2 \cdot 6H_2O$ (2.9 mg, 5.0 mol%), **L1*** (5.8 mg, 6.0 mol%), Na_2CO_3 (42.4 mg, 2.0 equiv), NaI (15.0 mg, 0.5 equiv), **L1** [0.29 mg, 0.75 mL stock solution (11.8 mg/ 30 mL in toluene)] and anhydrous NMP (0.25 mL). The mixture was stirred for 5 min at room temperature, at which time (*Z*)-*N*-(prop-1-en-1-yl)benzamide (32.2 mg, 0.20 mmol, 1.0 equiv), methyl 4-bromobenzoate (64.5 mg, 0.2 mmol, 1.0 equiv) and DMMS (74 µL, 0.60 mmol, 3.0 equiv) were added to the resulting mixture in this order. The tube was sealed with a teflon-lined screw cap, removed from the glove box and the reaction was stirred at 25 °C for up to 24 h (the mixture was stirred at 800 rpm, ensuring that the base was uniformly suspended). The reaction was quenched upon the addition of H_2O and the mixture was extracted with ethylacetate. The organic layer was concentrated to give the crude product. *n*-Dodecane (20 µL) was added as an internal standard for GC analysis. Detected by GC, **3v** (77% GC yield, >99:1 rr, 94% *ee*, on standard conditions; 40% GC yield, 96:4 rr, 96% *ee*, *w/o* **L1**) was obtained.

6. Extension of Alkene Scope

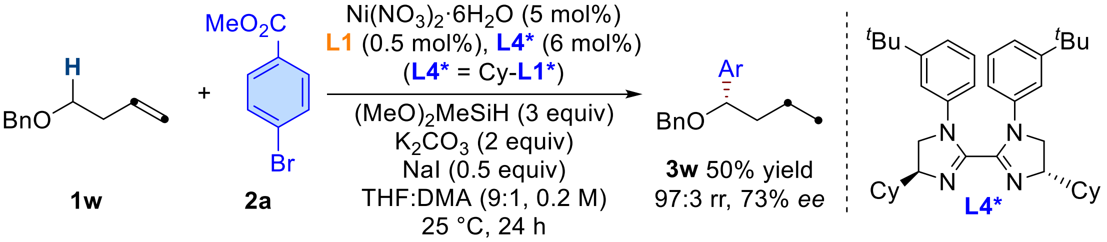

In a nitrogen-filled glove box, to an oven-dried 8 mL screw-cap vial equipped with a

magnetic stir bar was added $Ni(NO_3)_2{\cdot}6H_2O$ (5.8 mg, 10.0 mol%), **L4*** (6.8 mg, 6.0 mol%), K_2CO_3 (55.4 mg, 2.0 equiv), NaI (15.0 mg, 0.5 equiv), **L1** [0.29 mg, 0.9 mL stock solution (3.2 mg/ 10 mL in THF)] and anhydrous DMA (0.1 mL). The mixture was stirred for 5 min at room temperature, at which time ((but-3-en-1-yloxy)methyl)benzene (32.4 mg, 0.20 mmol, 1.0 equiv), methyl 4-bromobenzoate (64.5mg, 0.30 mmol, 1.5 equiv) and DMMS (74 μL, 0.60 mmol, 3.0 equiv) were added to the resulting mixture in this order. The tube was sealed with a teflon-lined screw cap, removed from the glove box and the reaction was stirred at 25 °C for up to 24 h (the mixture was stirred at 800 rpm, ensuring that the base was uniformly suspended). The crude material was purified by flash column chromatography (petroleum ether/EtOAc = 10:1) to provide the title compound as yellow oil in 50% yield (29.8 mg) with 97:3 rr and 73% *ee*.

^{1}H NMR (500 MHz, $CDCl_3$) δ 8.1 – 8.0 (m, 2H), 7.5 – 7.4 (m, 2H), 7.4 – 7.2 (m, 5H), 4.4 – 4.3 (m, 1H), 4.4 (m, 2H), 3.9 (s, 3H), 1.9 – 1.8 (m, 1H), 1.7 – 1.6 (m, 1H), 1.5 – 1.4 (m, 1H), 1.4 – 1.2 (m, 1H), 0.9 (t, J = 7.4 Hz, 3H).;

^{13}C NMR (126 MHz, $CDCl_3$) δ 167.1, 148.4, 138.4, 129.9, 129.5, 128.5, 127.9, 127.7, 126.9, 81.0, 70.8, 52.2, 40.5, 19.1, 14.0.;

HRMS (ESI) calcd. for $C_{19}H_{22}O_3Na$ $[M+Na]^+$ *m/z* 321.1461, found 321.1454;

$[\alpha]_D^{20}$ = –60.8 (c = 1.17, $CHCl_3$); 73% *ee*;

HPLC analysis CHIRALCEL® OD-H column, 3% ^{i}PrOH in hexane, 0.8 mL/min, 254 nm UV detector, t_R (major) = 12.0 min, t_R (minor) = 11.0 min.

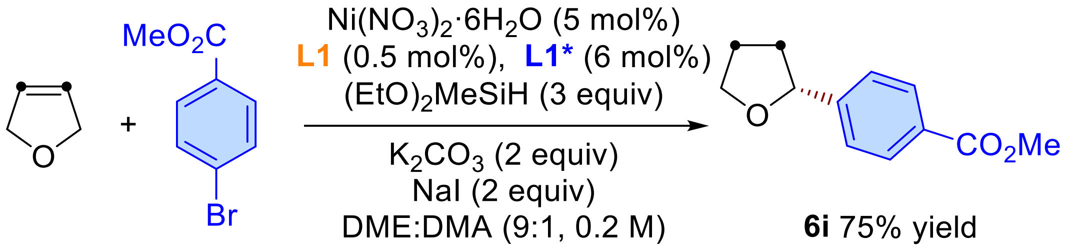

4i **2a** **6i** 75% yield
99:1 rr, 74% *ee*

In a nitrogen-filled glove box, to an oven-dried 8 mL screw-cap vial equipped with a magnetic stir bar was added $Ni(NO_3)_2{\cdot}6H_2O$ (5.8 mg, 10.0 mol%), **L1*** (5.8 mg, 6.0 mol%), K_2CO_3 (55.4 mg, 2.0 equiv), NaI (60.0 mg, 2.0 equiv), **L1** [0.29 mg, 0.9 mL

(3.2 mg/ 10 mL in DME)] and anhydrous DMA (0.1 mL). The mixture was stirred for 5 min at room temperature, at which time 2,5-dihydrofuran (14.0 mg, 0.20 mmol, 1.0 equiv), methyl 4-bromobenzoate (64.5mg, 0.30 mmol, 1.5 equiv) and DEMS (96 µL, 0.60 mmol, 3.0 equiv) were added to the resulting mixture in this order. The tube was sealed with a teflon-lined screw cap, removed from the glove box and the reaction was stirred at 25 °C for up to 24 h (the mixture was stirred at 800 rpm, ensuring that the base was uniformly suspended). The crude material was purified by flash column chromatography (petroleum ether/EtOAc = 5:1) to provide the title compound as colorless oil in 75% yield (30.9 mg) with 97:3 rr and 74% *ee*.

^{1}H NMR (500 MHz, $CDCl_3$) δ 8.02 – 7.96 (m, 2H), 7.41 – 7.36 (m, 2H), 4.93 (t, J = 7.2 Hz, 1H), 4.09 (q, 1H), 3.98 – 3.91 (m, 1H), 3.89 (s, 3H), 2.40 – 2.30 (m, 1H), 2.03 – 1.95 (m, 2H), 1.81 – 1.70 (m, 1H).

^{13}C NMR (126 MHz, $CDCl_3$) δ 167.1, 149.1, 129.8, 129.0, 125.5, 80.3, 69.0, 52.1, 34.8, 26.1.

HRMS (ESI) calcd. for $C_{18}H_{21}N_2O_3$ $[M-H]^-$ *m/z* 205.0870, found 205.0856;

$[\alpha]_D^{20}$ = –24.6 (c = 1.34, $CHCl_3$); 74% *ee*;

HPLC analysis CHIRALPAK® AD-H column, 3% ^{i}PrOH in hexane, 0.8 mL/min, 254 nm UV detector, t_R (major) = 13.8 min, t_R (minor) = 12.8 min.

Scheme S1. Unsuccessful substrates

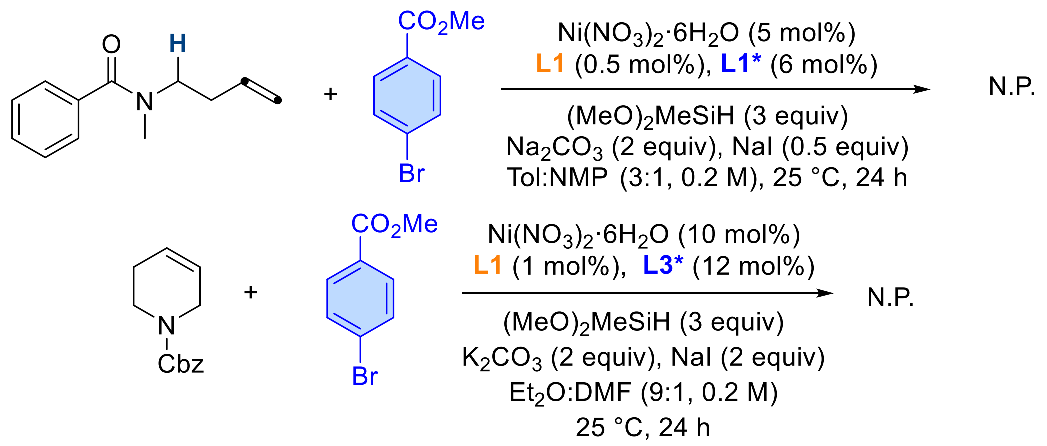

However, the alkenyl tertiary amides and the six-membered tetrahydropyridines were not applicable in this method.

7. Practical synthesis of (*S*)-nicotine and a CDK8 inhibitor

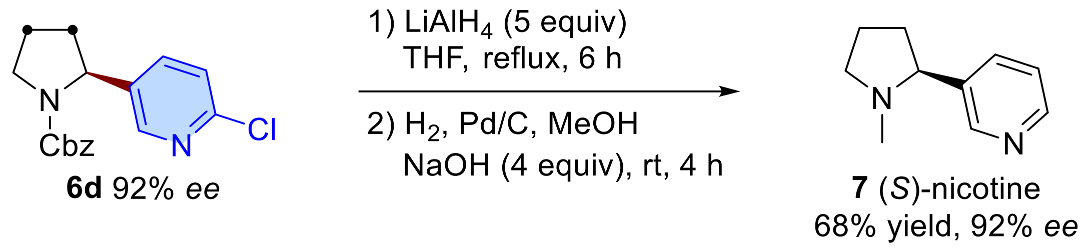

(*S*)-Nicotine [2] (Scheme 1d, **7**). The compound **6d** (316.1 mg, 1.0 mmol, 1.0 equiv) dissolved in THF (20 mL) was added to a stirred suspension of $LiAlH_4$ (200 mg, 5.0 equiv) in THF (5 mL) at 0 °C. The reaction mixture was refluxed for 6 h and then quenched with water at 0 °C. The mixture was filtered through the Celite pad, washed with chloroform and evaporated under vacuum to obtain the crude material. After adding methanol (5 mL) to a flask containing the crude material, Pd/C [Palladium 10% on Carbon (wetted with ca. 55% Water), 10.6 mg, 10 mol%] and sodium hydroxide (160.0 mg, 4.0 equiv) were added. Hydrogen was backfilled into the flask following evacuation under vacuum. The reaction was stirred at room temperature under balloon pressure of hydrogen for 4 hours. The mixture was then filtered through the Celite pad and concentrated. After dissolved in ethyl acetate and drying over potassium carbonate, the organic layer was filtered and concentrated. The crude material was purified by flash column chromatography (DCM/MeOH = 10:1) to provide the title compound as yellow oil in 68% yield (110.2 mg).

^{1}H NMR (500 MHz, $CDCl_3$) δ 8.57 – 8.43 (m, 2H), 7.76 – 7.62 (m, 1H), 7.29 – 7.22 (m, 1H), 3.29 – 3.21 (m, 1H), 3.09 (t, *J* = 8.3 Hz, 1H), 2.32 (q, *J* = 9.0 Hz, 1H), 2.24 – 2.18 (m, 1H), 2.17 (s, 3H), 2.02 – 1.91 (m, 1H), 1.88 – 1.79 (m, 1H), 1.77 – 1.68 (m, 1H);

^{13}C NMR (126 MHz, $CDCl_3$) δ 149.8, 148.8, 135.0, 123.7, 69.1, 57.2, 40.5, 35.3, 22.8;

HRMS (ESI) calcd. for $C_{10}H_{15}N_2$ $[M+H]^+$ *m/z* 163.1230, found 163.1237;

IR (neat, cm^{-1}) 2927, 1706, 1414, 1264, 731;

$[\alpha]_D^{25}$ = –12.0 (c = 1.00, $CHCl_3$); 92% *ee*; **$[\alpha]_D^{25}$** = -98.5 (c = 0.67, EtOH) [2];

HPLC analysis CHIRALCEL® OD-H column, 3% ^{i}PrOH in hexane, 0.8 mL/min, 254

nm UV detector, t_R (major) = 10.4 min, t_R (minor) = 12.8 min.

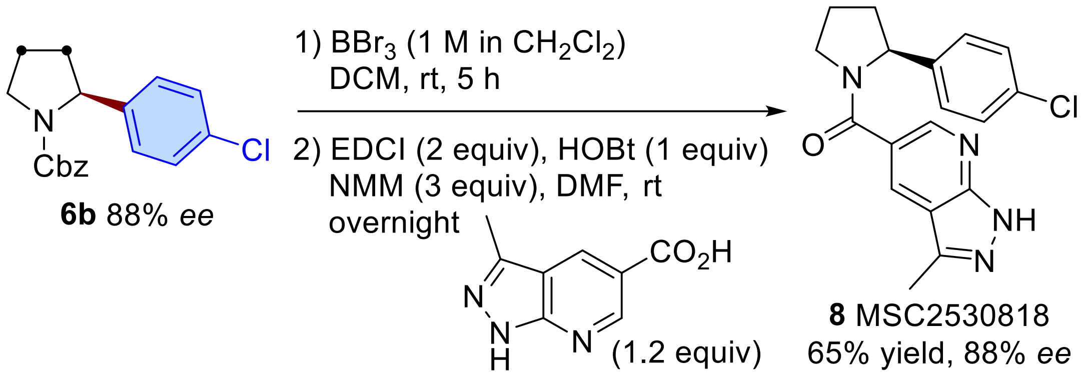

CDK8 inhibitor (Scheme 1d, **8**). The compound **6b** (315.0 mg, 1.0 mmol, 1 equiv) dissolved in dry CH_2Cl_2 (40 mL) was added BBr_3 (6 mL, 1 M solution in CH_2Cl_2, 6.0 mmol, 6 equiv) at 0 °C carefully[3]. The reaction mixture was warmed to room temperature and stirred for 5 h. Excess of BBr_3 was quenched by MeOH. The reaction mixture was concentrated under vacuum to give a crude product. The crude product was purified by flash column chromatography (EtOAc/MeOH/triethylamine = 9:1:0.1) to provide the targeted compound.

According to the literature[4], to the product, 3-methyl-1*H*pyrazolo[3,4-b]pyridine-5-carboxylic acid (212.4 mg, 1.2 mmol, 1.2 equiv), *N*-(3-dimethylaminopropyl)-*N'*-ethylcarbodiimide hydrochloride (383.4 mg, 2.0 mmol, 2.0 equiv), and 1-hydroxybenzotriazole (135.1 mg, 1.0 mmol, 1.0 equiv) were added, followed by *N,N*-dimethylformamide (5 mL). 4-Methylmorpholine (303.3 mg, 3.0 mmol, 3.0 equiv) was added at rt, and the reaction mixture was allowed to stir at rt overnight. The mixture was diluted with ethyl acetate, washed with water followed by brine, and dried over Na_2SO_4. The solvent was removed under reduced pressure to provide the crude product. The crude product was purified by flash column chromatography (EtOAc/MeOH/triethylamine = 9:1:0.1) to provide the title compound as beige solid in 65% yield (221.1 mg).

^{1}H NMR (500 MHz, DMSO-d_6) δ 13.50 – 13.22 (m, 1H), 8.90 – 6.88 (m, 6H), 5.27 – 4.92 (m, 1H), 4.00 – 3.52 (m, 2H), 2.57 – 2.53 (m, 2H), 2.44 – 2.28 (m, 2H), 1.94 – 1.69 (m, 3H);

^{13}C NMR (126 MHz, DMSO-d_6) δ 167.3, 152.4, 148.2, 147.0, 143.1, 142.2, 130.9, 129.2, 128.2, 127.7, 124.9, 112.9, 60.41, 50.71, 34.82, 24.94, 12.25;

HRMS (ESI) calcd. for $C_{10}H_{15}N_2$ $[M+H]^+$ *m/z* 341.1164, found 341.1158;

m.p. 104.0 – 105.7 °C;

$[\alpha]_D^{25}$ = –102.4 (c = 0.92, $CHCl_3$); 88% *ee*;

HPLC analysis CHIRALCEL® OJ-H column, 30% ^{i}PrOH in hexane, 0.8 mL/min, 254 nm UV detector, t_R (major) = 8.4 min, t_R (minor) = 12.4 min.

8. Conditions Optimization

Table S1: Asymmetric Migratory Hydroarylation to Access Enantioenriched *α*-(Hetero)Aryl-Substituted Amines

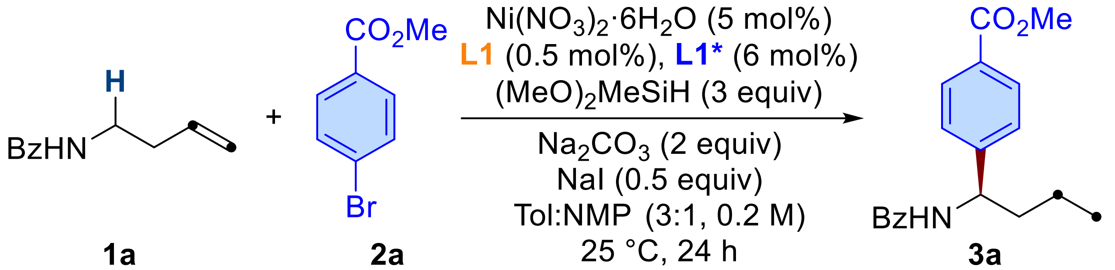

entry	deviation from standard conditions	Yield (%)	rr	*ee* (%)
1	none	73 (72)	97:3	92
2	*w/o* **L1**	0	-	-
3	*w/o* **L1***	0	-	-
4	**L1** reduced to 0.3 mol%	69	92:8	96
5	**L2** instead of **L1**	12	>99:1	89
6	**L2*** instead of **L1***	13	>99:1	-36
7	**L3*** instead of **L1***	72	93:7	-94
8	$NiBr_2$•dme instead of $Ni(NO_2)_3$•$6H_2O$	69	>99:1	86
9	NiI_2 instead of $Ni(NO_2)_3$•$6H_2O$	70	96:4	87
10	DEMS instead of DMMS	69	97:3	92
11	$(MeO)_3SiH$ instead of DMMS	41	99:1	86
12	K_2CO_3 instead of Na_2CO_3	70	>99:1	60
13	NaF instead of Na_2CO_3	0	-	-
14	*w/o* NaI	14	60:40	95
15	NMP only	19	>99:1	87
16	Tol only	0	-	-
17	ArI instead of ArBr	59	94:6	92

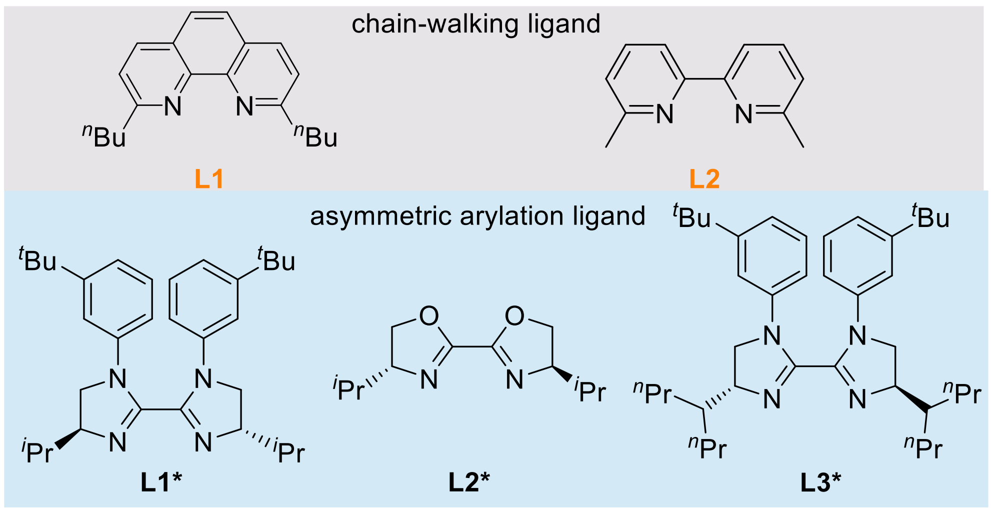

Scheme S2. The solvent-controlled experiments

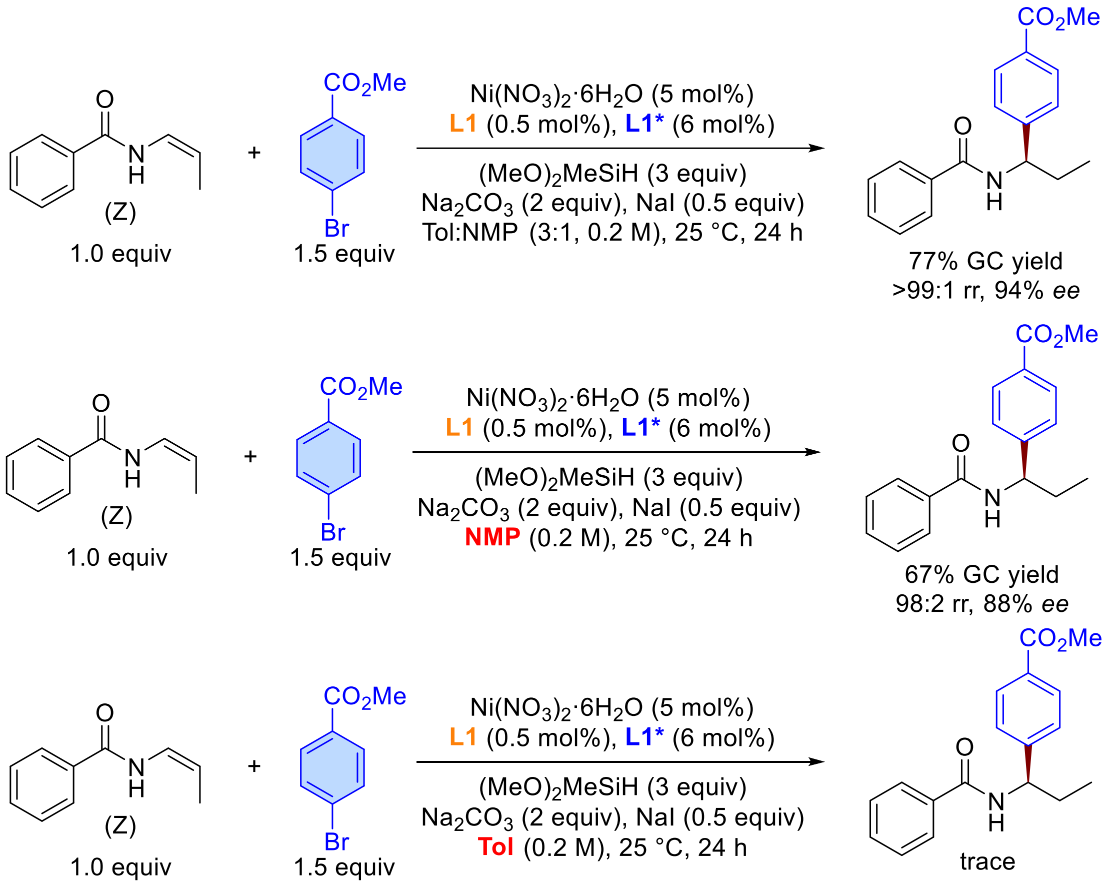

Table S2: Asymmetric Migratory Hydroarylation to Access Enantioenriched α-(Hetero)Aryl-Substituted *N*-heterocycles

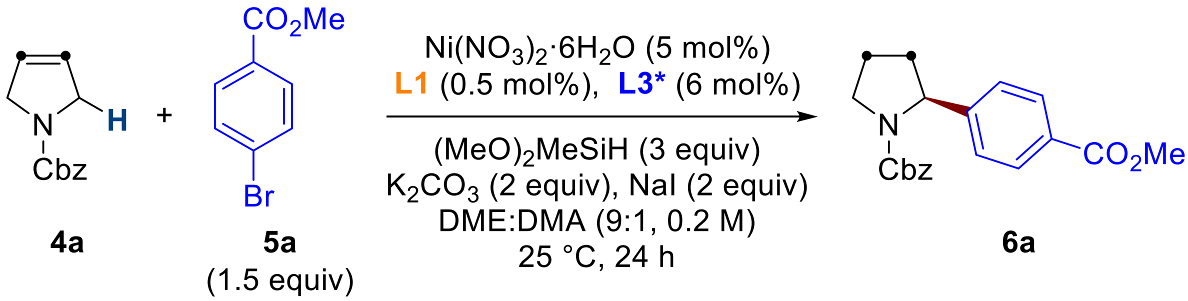

entry	deviation from standard conditions	Yield (%)	rr	*ee* (%)
1	none	78 (67)	>99:1	90
2	*w/o* **L1**	12	>99:1	86
3	*w/o* **L3***, 6 mol% **L1** used	51	>99:1	0
4	**L1*** instead of **L3***	29	>99:1	-75
5	Na_2CO_3 instead of K_2CO_3	32	>99:1	86
6	*w/o* NaI	23	>99:1	80

9. References

[1] X. Shu, D. Zhong, Y. Lin, X. Qin, H. Huo, *J. Am. Chem. Soc.* **2022**, *144*, 8797–8806.

[2] C. Guo, D.-W. Sun, S. Yang, S.-J. Mao, X.-H. Xu, S.-F. Zhu, Q.-L. Zhou, *J. Am. Chem. Soc.* 2015, 137, 1, 90–93.

[3] S. Dharuman, A. K. Palanivel, Y. D. Vankar, *Org. Bio. Chem.* **2014**, *12*, 4983–4998.

[4] P. Czodrowski, A. Mallinger, D. Wienke, C. Esdar, O. Pöschke, M. Busch, F. Rohdich, S. A. Eccles, M.-J. Ortiz-Ruiz, R. Schneider, F. I. Raynaud, P. A. Clarke, D. Musil, D. Schwarz, T. Dale, K. Urbahns, J. Blagg, K. Schiemann, *J. Med. Chem.* **2016**, *59*, 9337–9349.

10. Spectroscopic Data (NMR Spectrum)

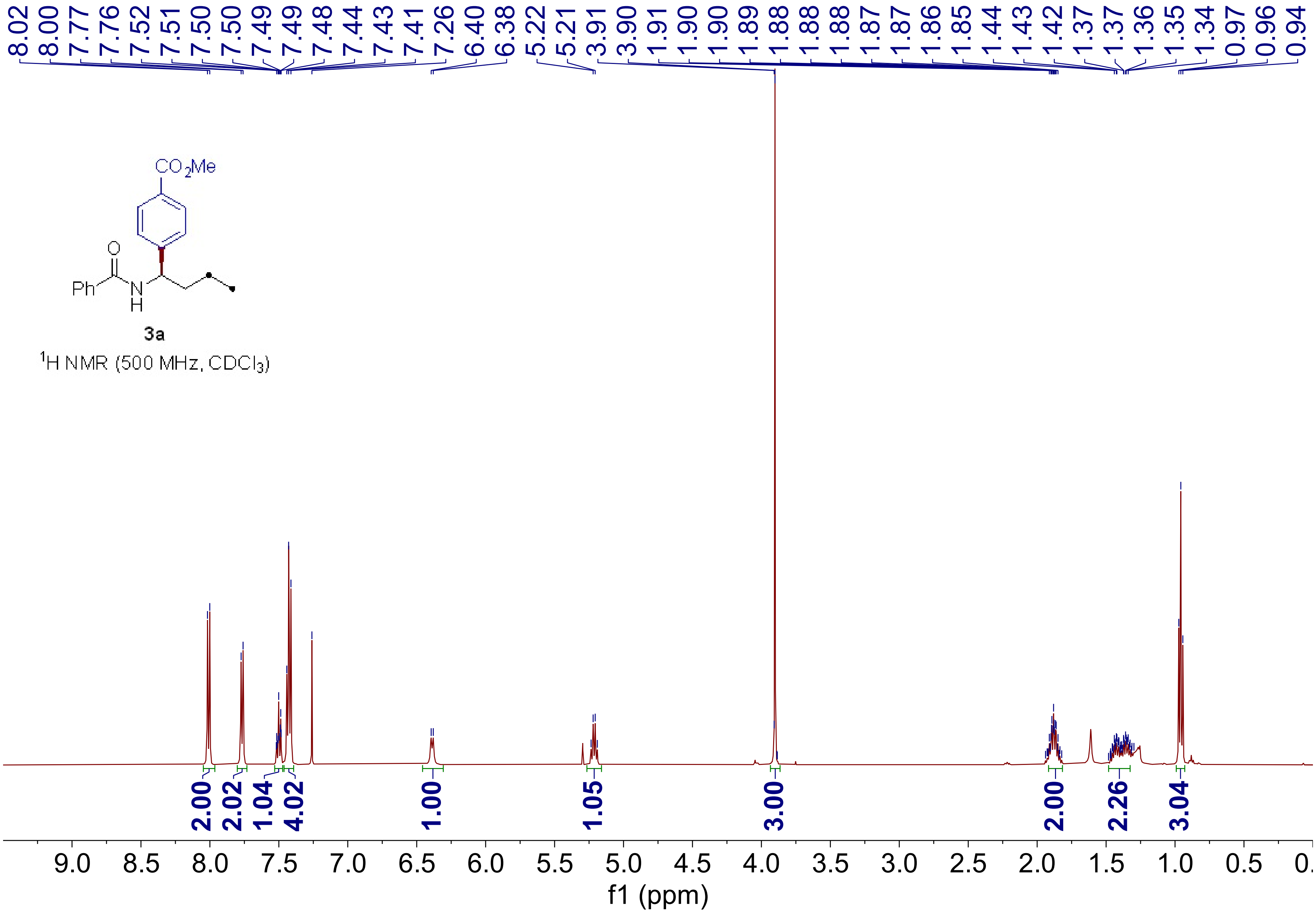

8.02 8.00 7.77 7.76 7.52 7.51 7.50 7.50 7.49 7.49 7.48 7.44 7.43 7.41 7.26 6.40 6.38 5.22 5.21 3.91 3.90 1.91 1.90 1.90 1.89 1.88 1.88 1.88 1.87 1.87 1.86 1.85 1.44 1.43 1.42 1.37 1.37 1.36 1.35 1.34 0.97 0.96 0.94
CO2Me
O
Ph
N
H
3a
1H NMR (500 MHz, CDCl3)
2.00 2.02 1.04 4.02 1.00 1.05 3.00 2.00 2.26 3.04
9.0 8.5 8.0 7.5 7.0 6.5 6.0 5.5 5.0 4.5 4.0 3.5 3.0 2.5 2.0 1.5 1.0 0.5 0.
f1 (ppm)

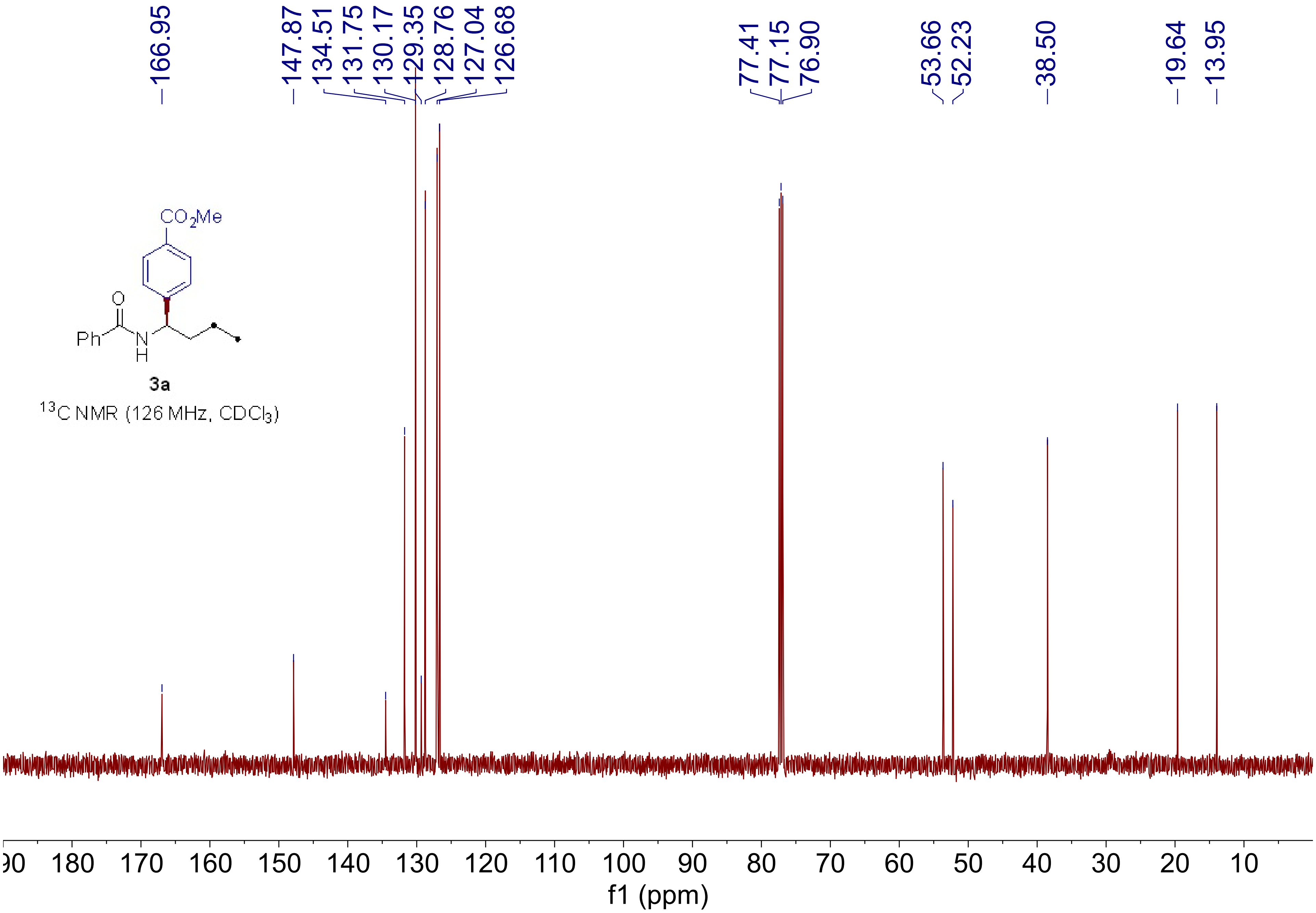
166.95
147.87
134.51
131.75
130.17
129.35
128.76
127.04
126.68
77.41
77.15
76.90
53.66
52.23
38.50
19.64
13.95
CO2Me
O
Ph
N
H
3a
13C NMR (126 MHz, CDCl3)
90 180 170 160 150 140 130 120 110 100 90 80 70 60 50 40 30 20 10
f1 (ppm)

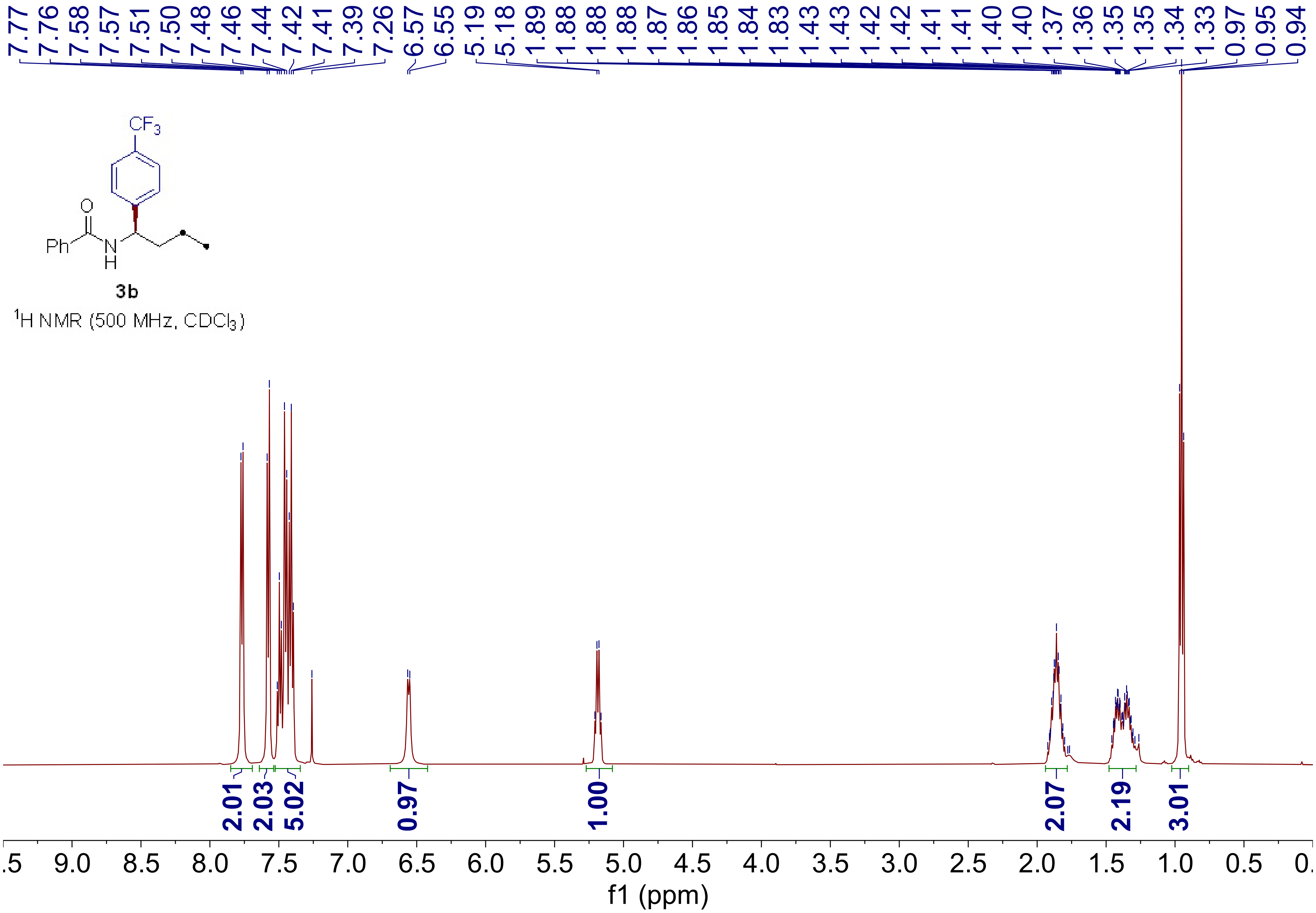
7.77 7.76 7.58 7.57 7.51 7.50 7.48 7.46 7.44 7.42 7.41 7.39 7.26 6.57 6.55 5.19 5.18 1.89 1.88 1.88 1.88 1.87 1.86 1.85 1.84 1.83 1.43 1.43 1.42 1.42 1.41 1.41 1.40 1.40 1.37 1.36 1.35 1.35 1.34 1.33 0.97 0.95 0.94
CF3
O
Ph
N
H
3b
1H NMR (500 MHz, CDCl3)
2.01 2.03 5.02 0.97 1.00 2.07 2.19 3.01
.5 9.0 8.5 8.0 7.5 7.0 6.5 6.0 5.5 5.0 4.5 4.0 3.5 3.0 2.5 2.0 1.5 1.0 0.5 0.
f1 (ppm)

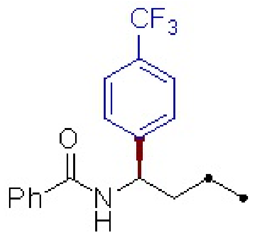

3b

^{13}C NMR (126 MHz, $CDCl_3$)

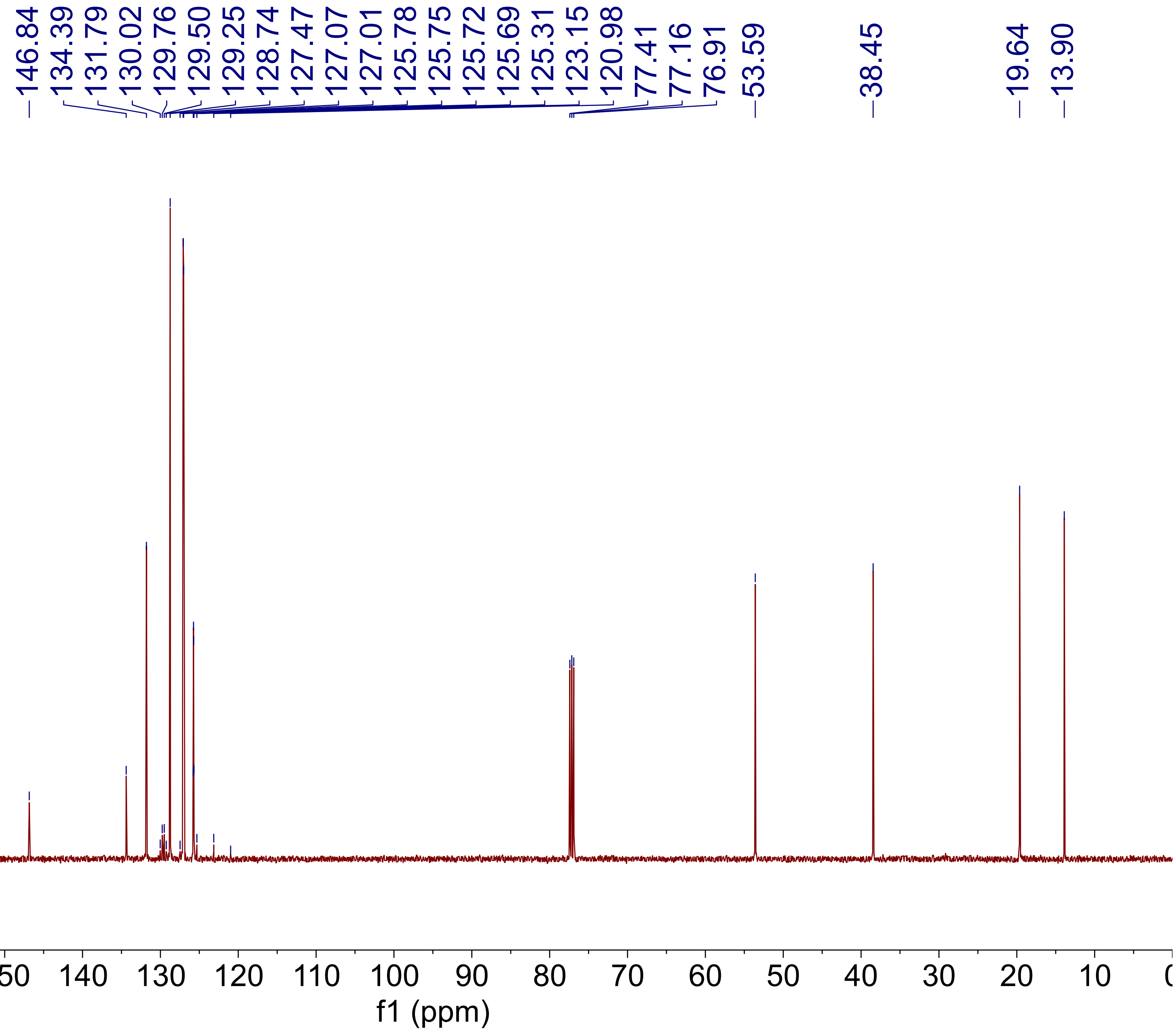

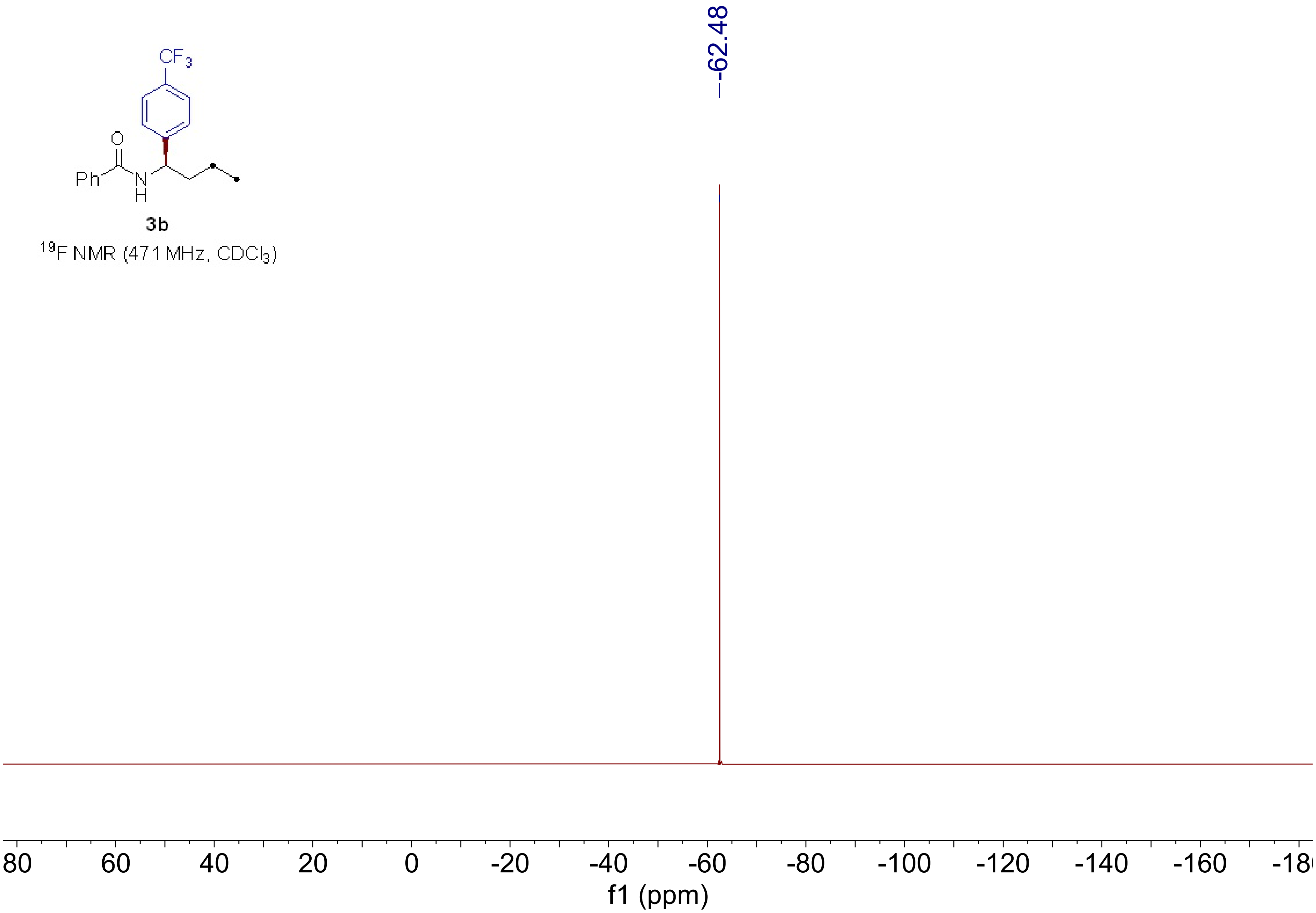
CF3
O
Ph
N
H
3b
19F NMR (471 MHz, CDCl3)
-62.48
80
60
40
20
0
-20
-40
-60
-80
-100
-120
-140
-160
f1 (ppm)

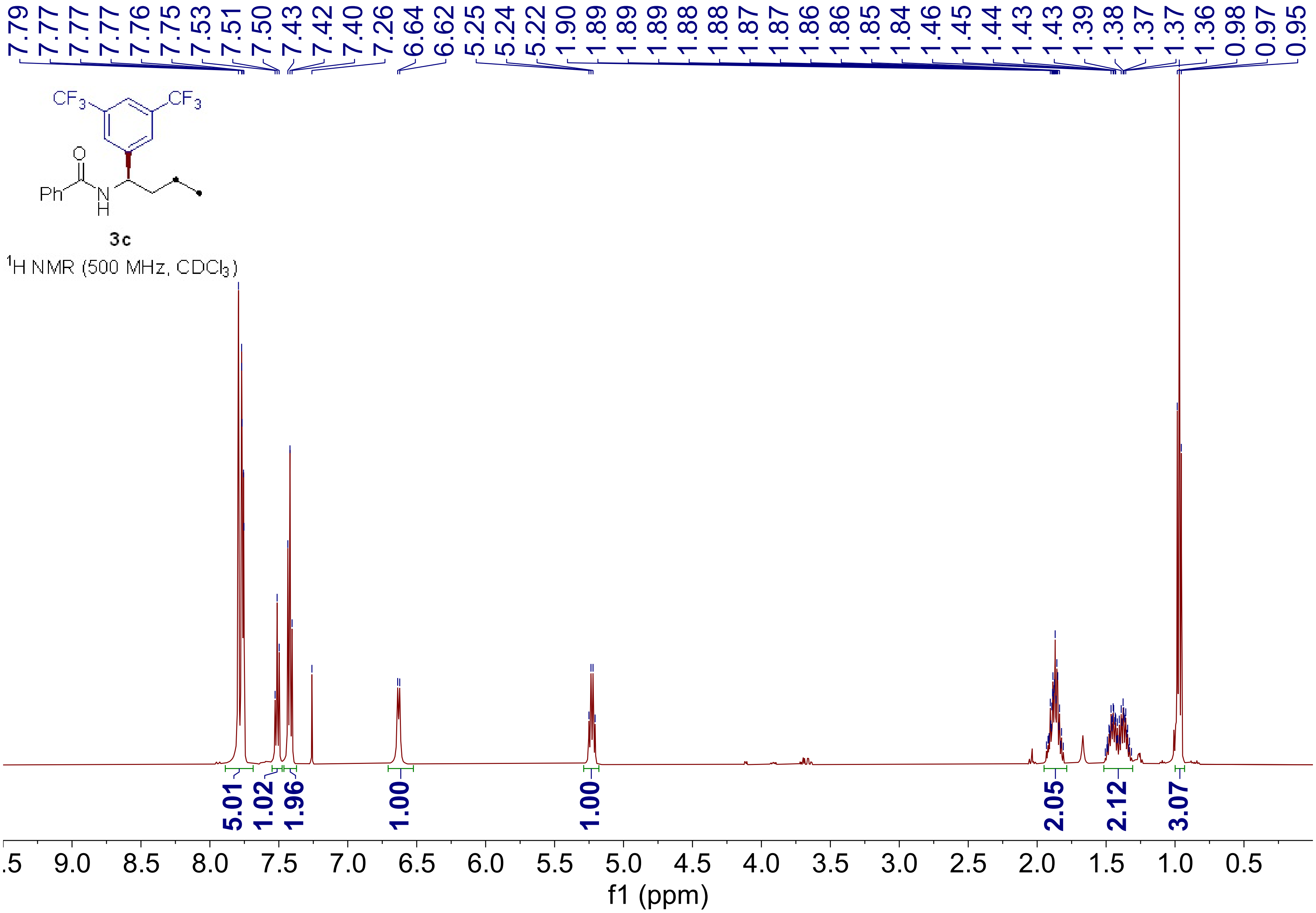

CF3
CF3
O
Ph
N
H
3c
1H NMR (500 MHz, CDCl3)
7.79 7.77 7.77 7.77 7.76 7.75 7.53 7.51 7.50 7.43 7.42 7.40 7.26 6.64 6.62 5.25 5.24 5.22 1.90 1.89 1.89 1.89 1.88 1.88 1.87 1.87 1.86 1.86 1.85 1.84 1.46 1.45 1.44 1.43 1.43 1.39 1.38 1.37 1.37 1.36 0.98 0.97 0.95
5.01 1.02 1.96 1.00 1.00 2.05 2.12 3.07
.5 9.0 8.5 8.0 7.5 7.0 6.5 6.0 5.5 5.0 4.5 4.0 3.5 3.0 2.5 2.0 1.5 1.0 0.5
f1 (ppm)

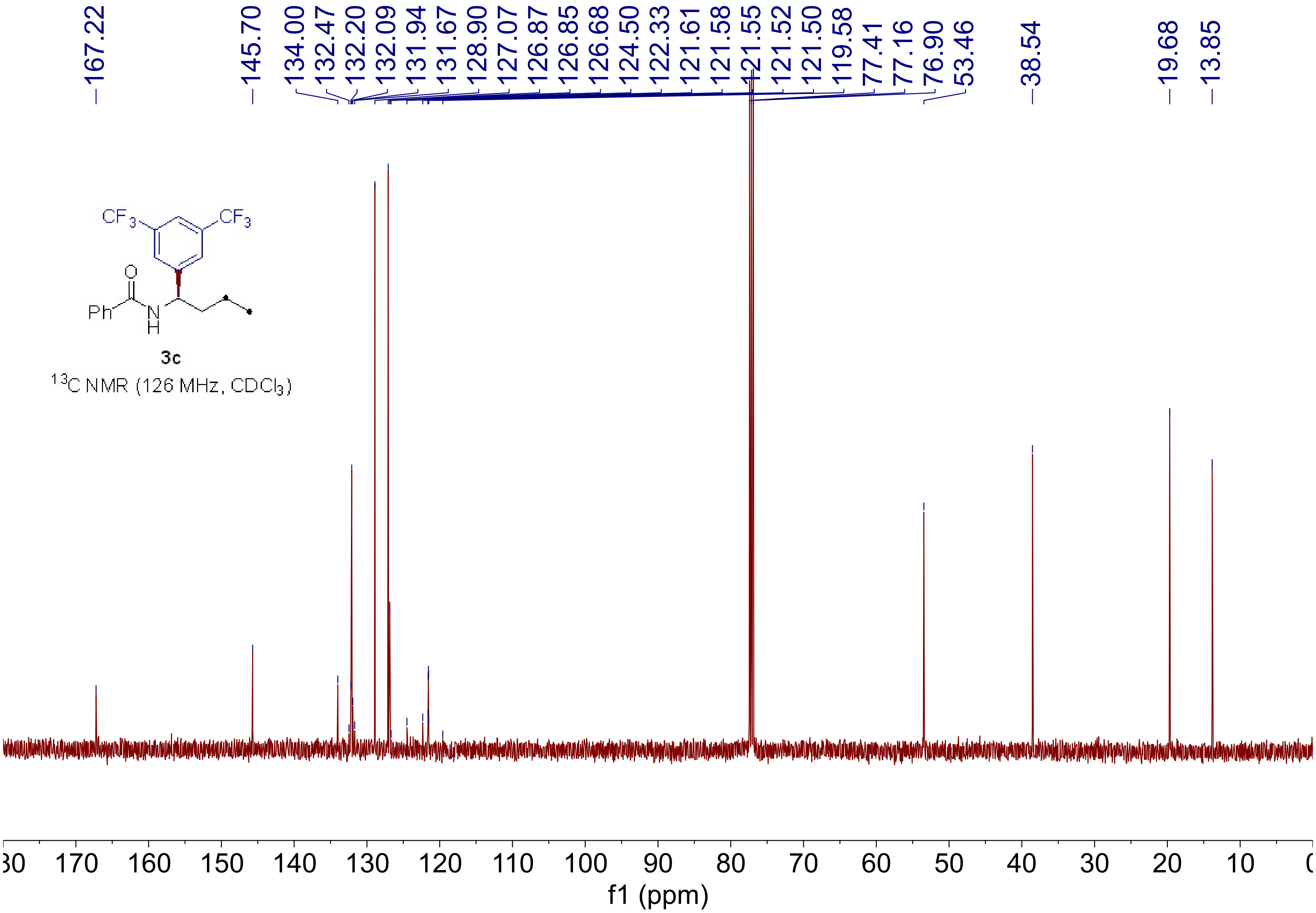
167.22
145.70
134.00
132.47
132.20
132.09
131.94
131.67
128.90
127.07
126.87
126.85
126.68
124.50
122.33
121.61
121.58
121.55
121.52
121.50
119.58
77.41
77.16
76.90
53.46
38.54
19.68
13.85
CF3
CF3
O
Ph
N
H
3c
13C NMR (126 MHz, CDCl3)
170 160 150 140 130 120 110 100 90 80 70 60 50 40 30 20 10
f1 (ppm)

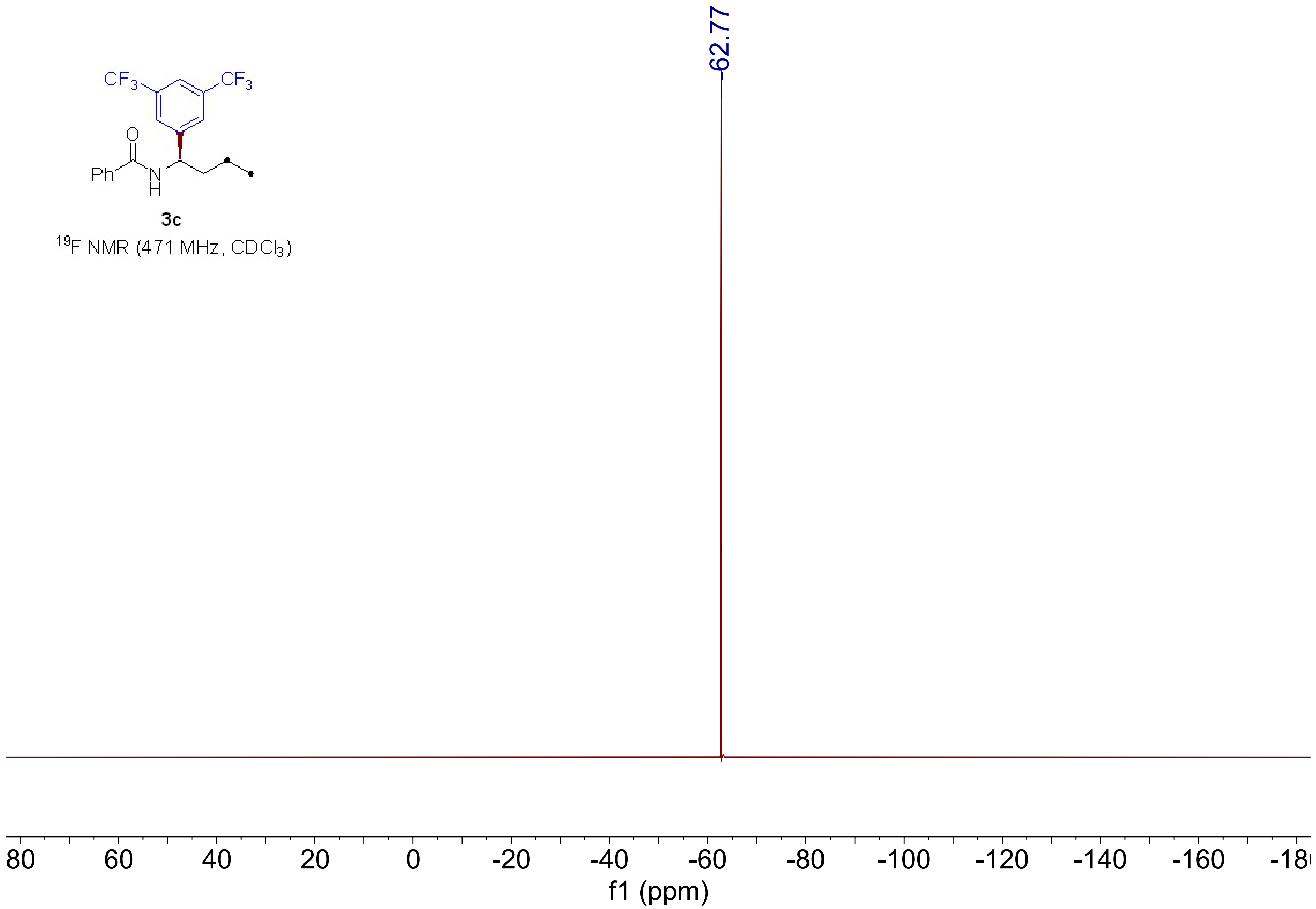
-62.77
CF3
CF3
O
Ph
N
H
3c
19F NMR (471 MHz, CDCl3)
80 60 40 20 0 -20 -40 -60 -80 -100 -120 -140 -160 -18
f1 (ppm)

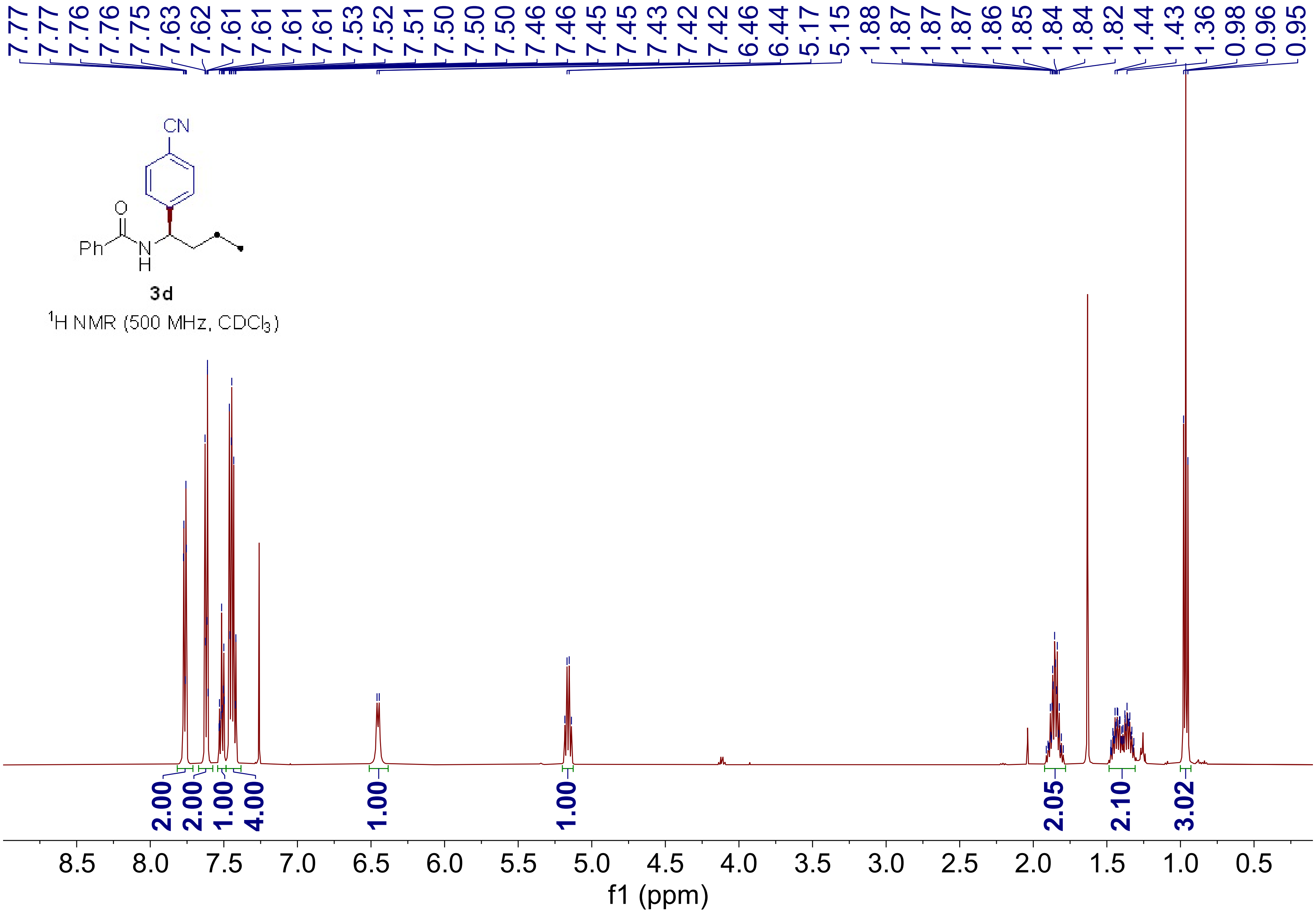

7.77 7.77 7.76 7.76 7.75 7.63 7.62 7.61 7.61 7.61 7.61 7.53 7.52 7.51 7.50 7.50 7.50 7.46 7.46 7.45 7.45 7.43 7.42 7.42 6.46 6.44 5.17 5.15 1.88 1.87 1.87 1.87 1.86 1.85 1.84 1.84 1.82 1.44 1.43 1.36 0.98 0.96 0.95
CN
O
Ph
N
H
3d
¹H NMR (500 MHz, CDCl3)
2.00 2.00 1.00 4.00 1.00 1.00 2.05 2.10 3.02
8.5 8.0 7.5 7.0 6.5 6.0 5.5 5.0 4.5 4.0 3.5 3.0 2.5 2.0 1.5 1.0 0.5
f1 (ppm)

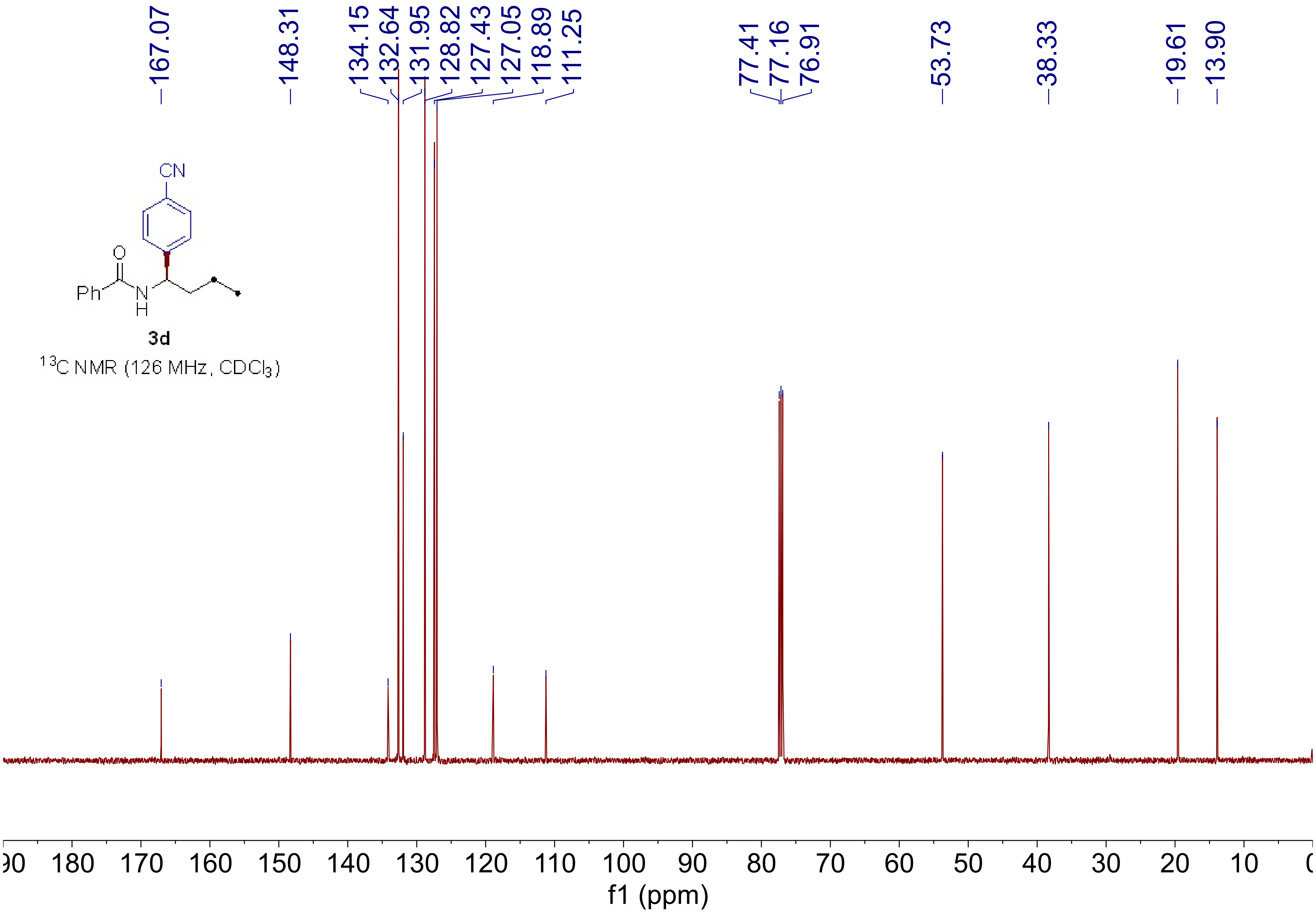
167.07
148.31
134.15
132.64
131.95
128.82
127.43
127.05
118.89
111.25
77.41
77.16
76.91
53.73
38.33
19.61
13.90
CN
O
Ph
N
H
3d
^{13}C NMR (126 MHz, CDCl$_3$)
f1 (ppm)

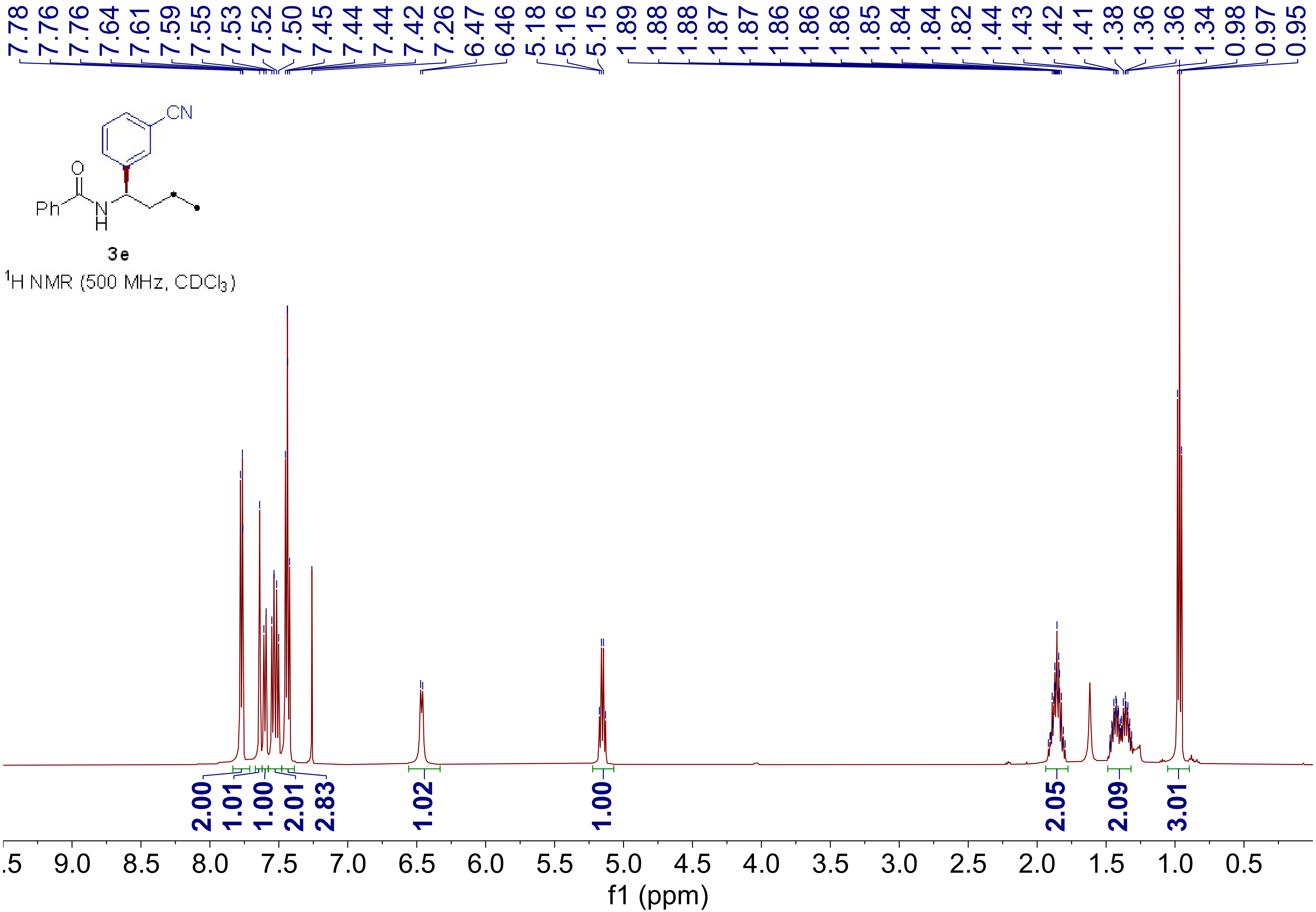

3e

^{1}H NMR (500 MHz, $CDCl_3$)

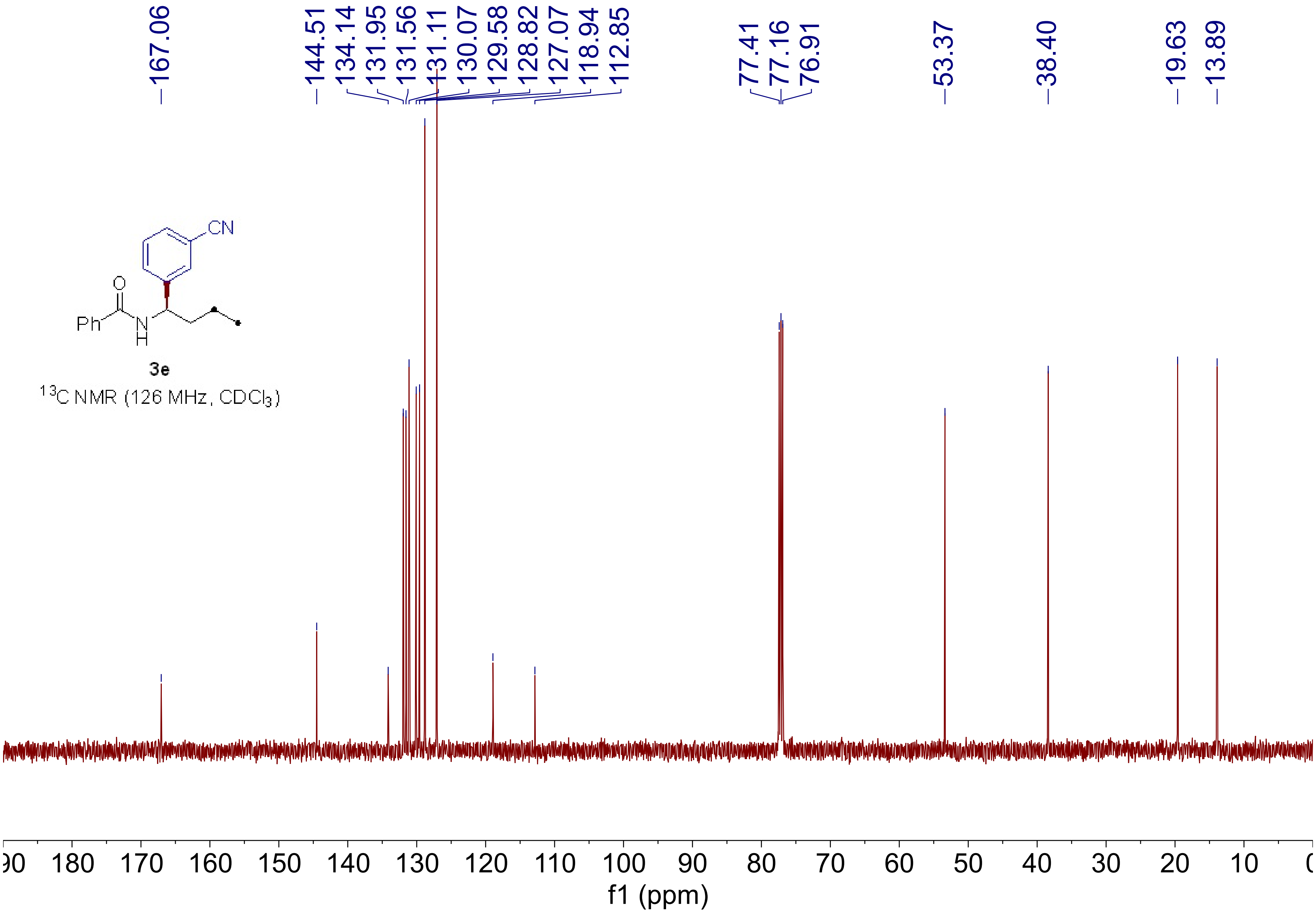
167.06
144.51
134.14
131.95
131.56
131.11
130.07
129.58
128.82
127.07
118.94
112.85
77.41
77.16
76.91
53.37
38.40
19.63
13.89
CN
O
Ph
N
H
3e
13C NMR (126 MHz, CDCl3)
180
170
160
150
140
130
120
110
100
90
80
70
60
50
40
30
20
10
f1 (ppm)

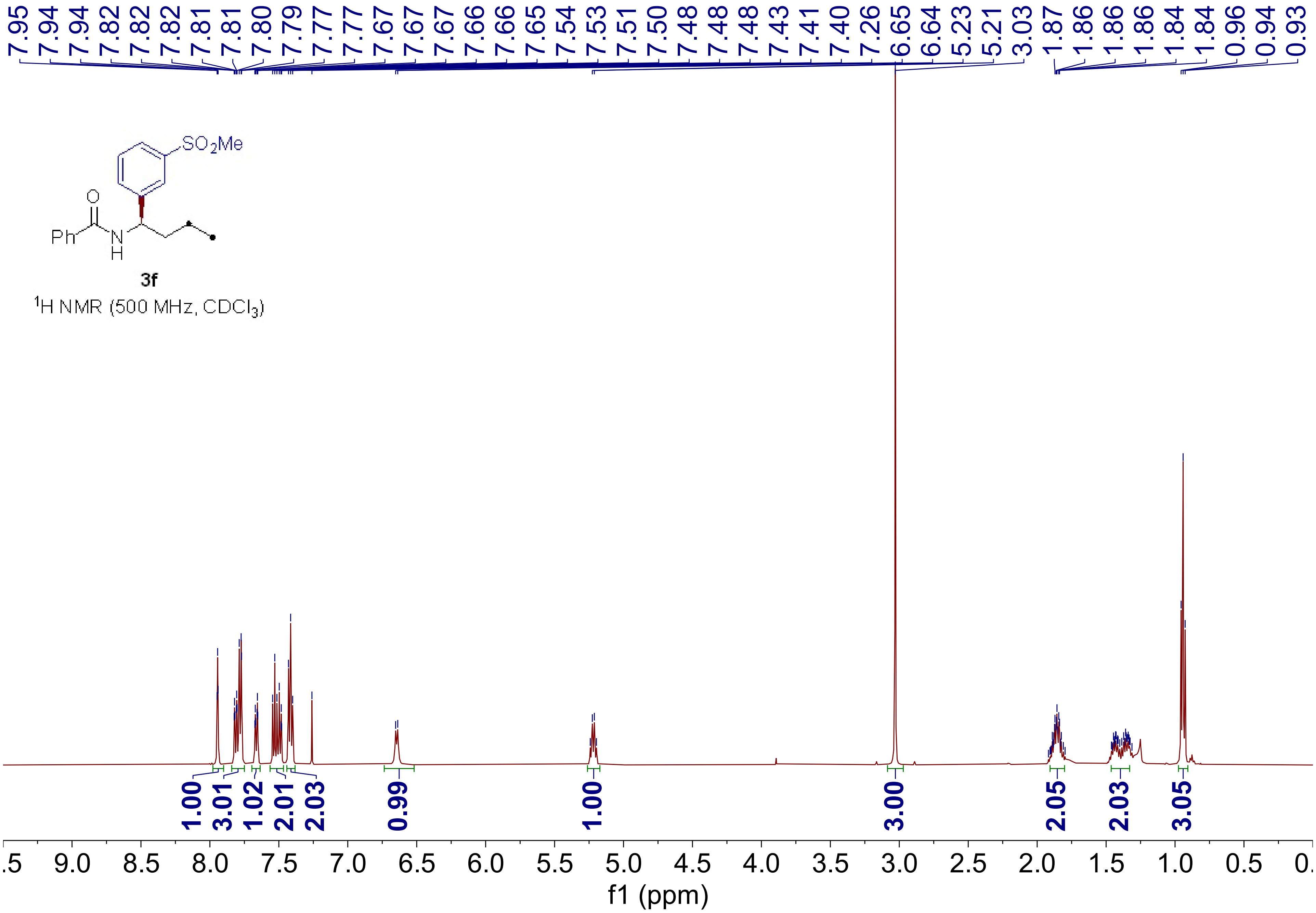
SO2Me
Ph
O
N
H
3f
1H NMR (500 MHz, CDCl3)
7.95 7.94 7.94 7.82 7.82 7.81 7.81 7.80 7.79 7.77 7.77 7.67 7.67 7.67 7.66 7.66 7.65 7.54 7.53 7.51 7.50 7.48 7.48 7.48 7.43 7.41 7.40 7.26 6.65 6.64 5.23 5.21 3.03 1.87 1.86 1.86 1.86 1.84 1.84 0.96 0.94 0.93
1.00 3.01 1.02 2.01 2.03 0.99 1.00 3.00 2.05 2.03 3.05
f1 (ppm)

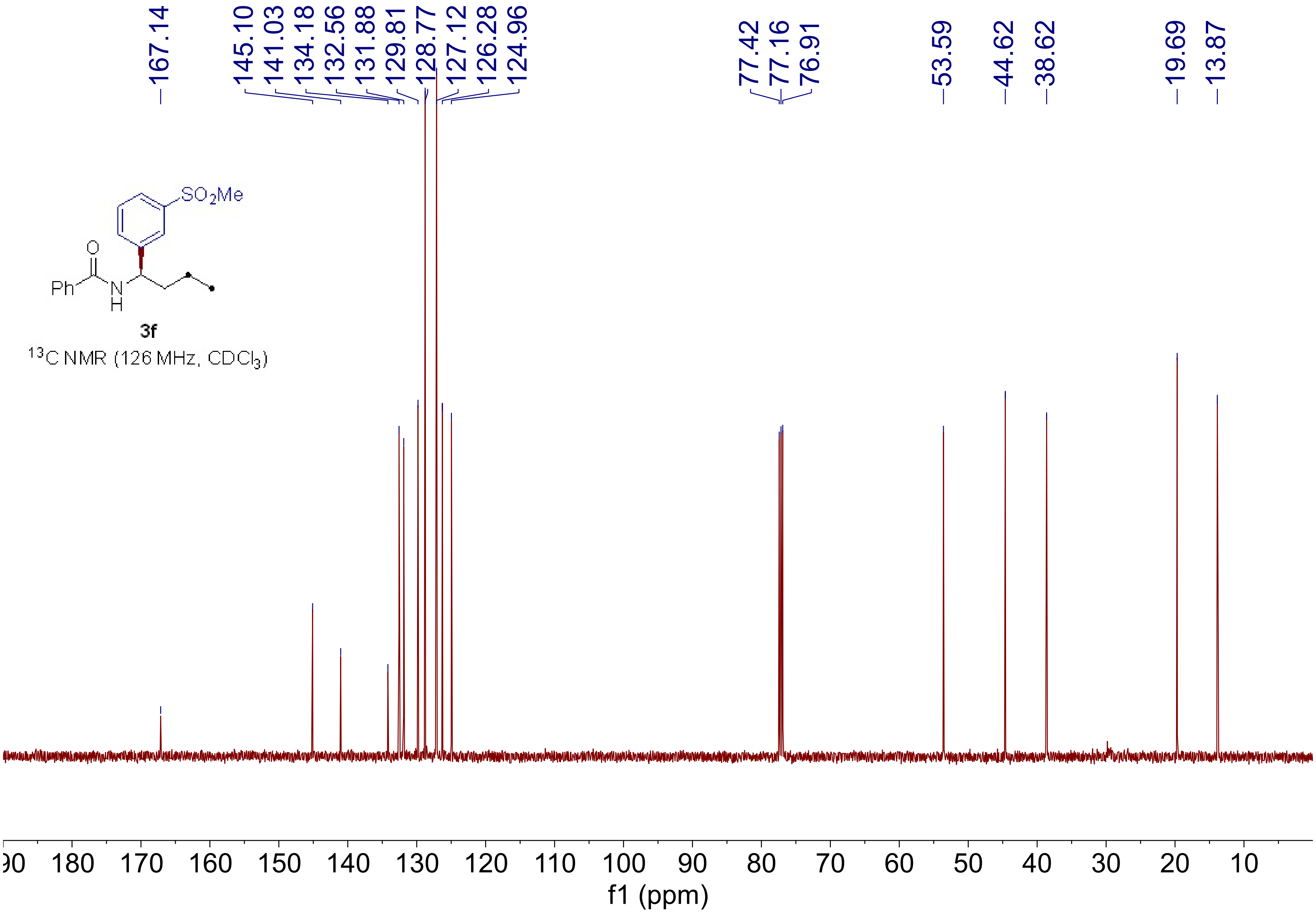
167.14
145.10
141.03
134.18
132.56
131.88
129.81
128.77
127.12
126.28
124.96
77.42
77.16
76.91
53.59
44.62
38.62
19.69
13.87
SO2Me
O
Ph
N
H
3f
13C NMR (126 MHz, CDCl3)
180 170 160 150 140 130 120 110 100 90 80 70 60 50 40 30 20 10
f1 (ppm)

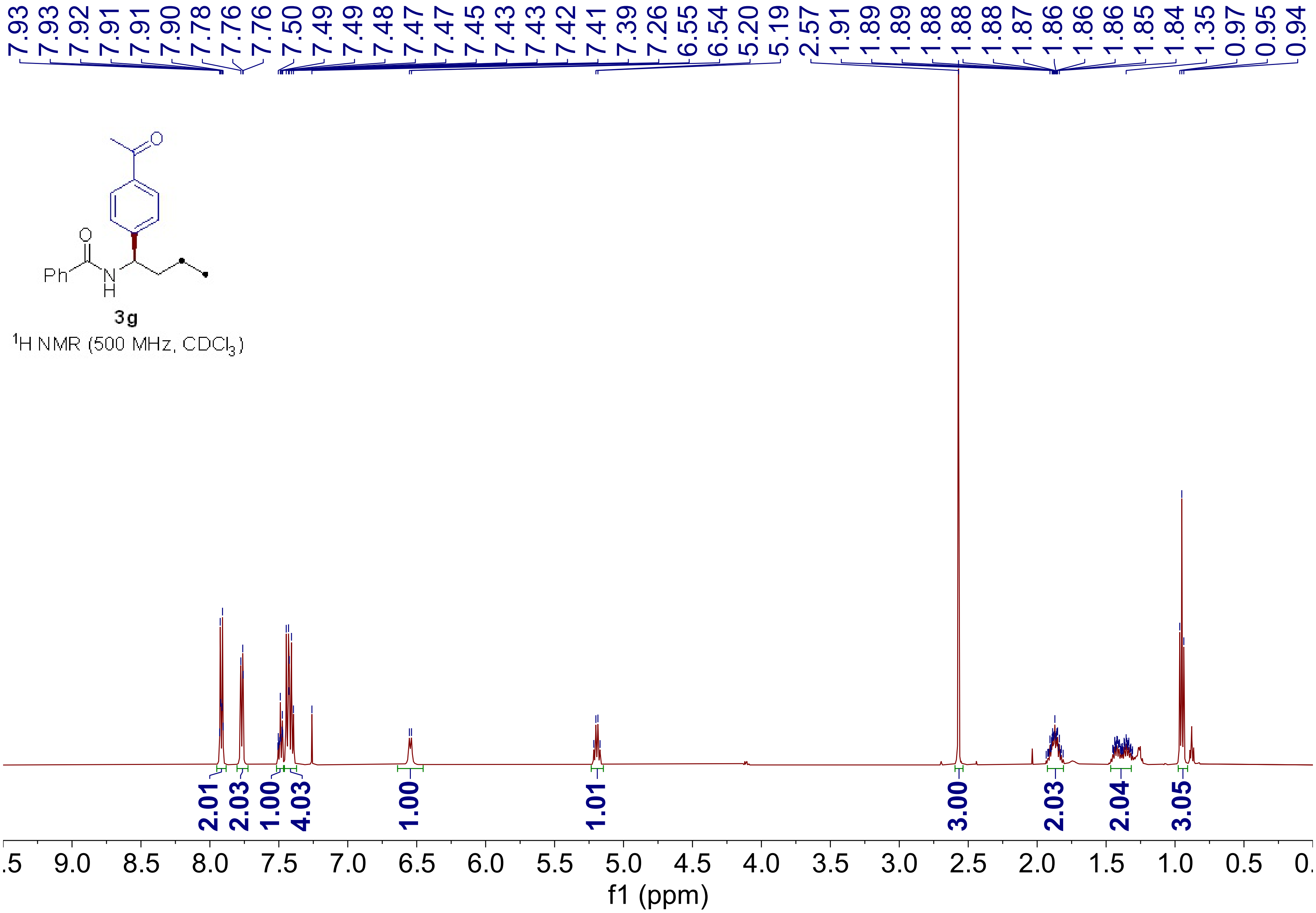

3g

^{1}H NMR (500 MHz, $CDCl_3$)

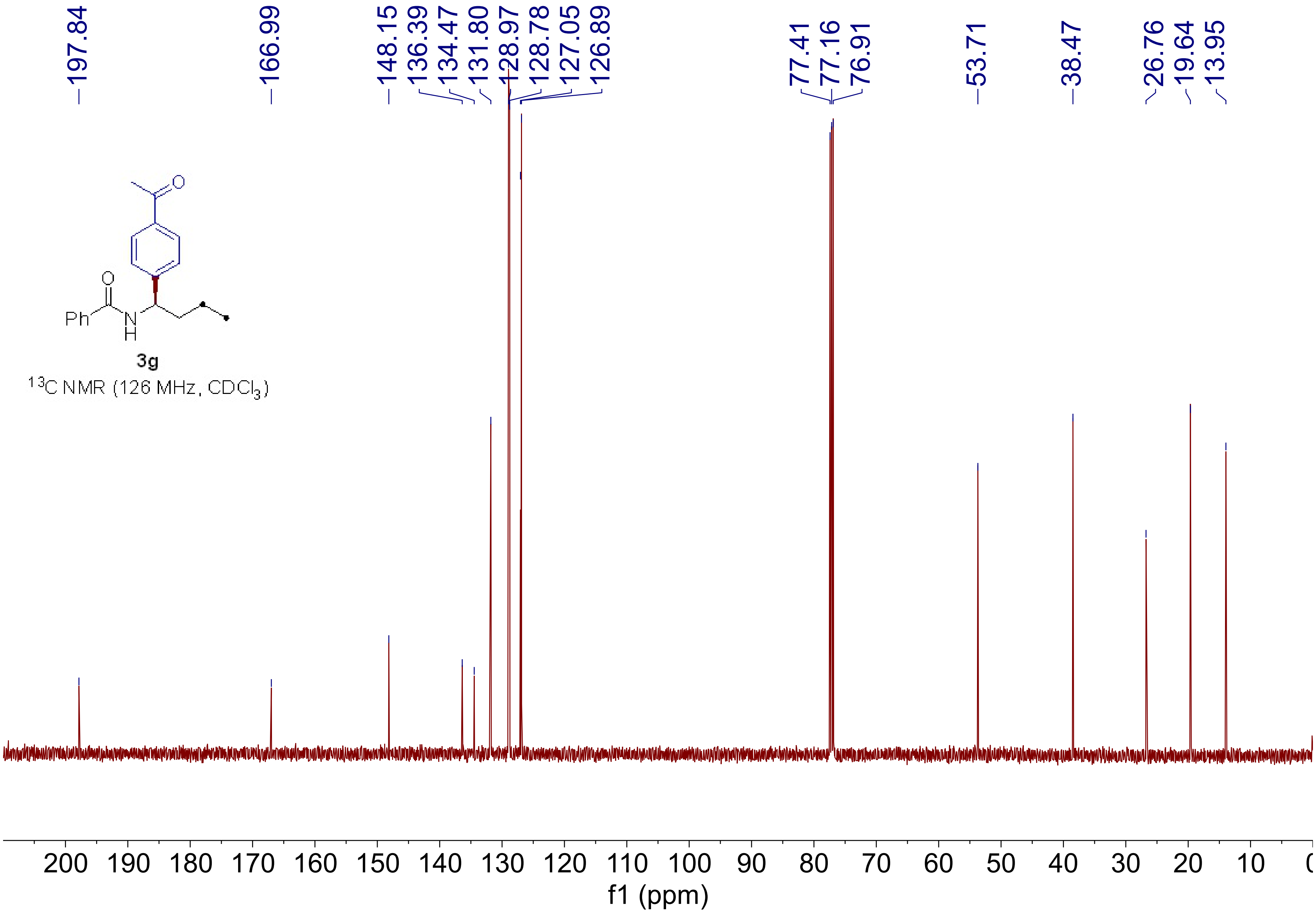
197.84
166.99
148.15
136.39
134.47
131.80
128.97
128.78
127.05
126.89
77.41
77.16
76.91
53.71
38.47
26.76
19.64
13.95
Ph
O
N
H
O
3g
^{13}C NMR (126 MHz, CDCl$_3$)
200 190 180 170 160 150 140 130 120 110 100 90 80 70 60 50 40 30 20 10
f1 (ppm)

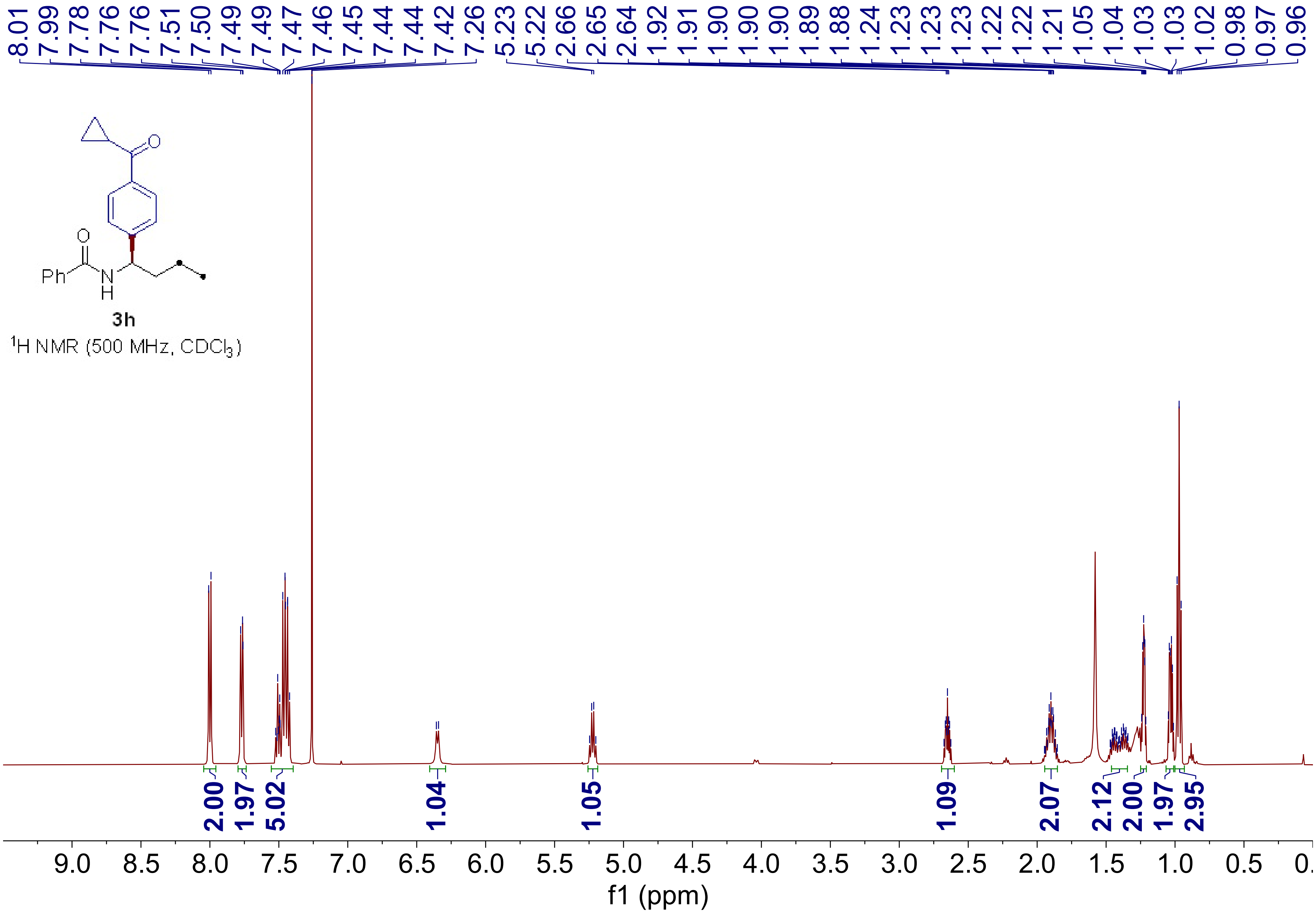

8.01 7.99 7.78 7.76 7.76 7.51 7.50 7.49 7.49 7.47 7.46 7.45 7.44 7.44 7.42 7.26 5.23 5.22 2.66 2.65 2.64 1.92 1.91 1.90 1.90 1.89 1.88 1.24 1.23 1.23 1.23 1.22 1.22 1.21 1.05 1.04 1.03 1.03 1.02 0.98 0.97 0.96
O
Ph
N
H
O
3h
^{1}H NMR (500 MHz, CDCl$_3$)
2.00 1.97 5.02 1.04 1.05 1.09 2.07 2.12 2.00 1.97 2.95
9.0 8.5 8.0 7.5 7.0 6.5 6.0 5.5 5.0 4.5 4.0 3.5 3.0 2.5 2.0 1.5 1.0 0.5 0.
f1 (ppm)

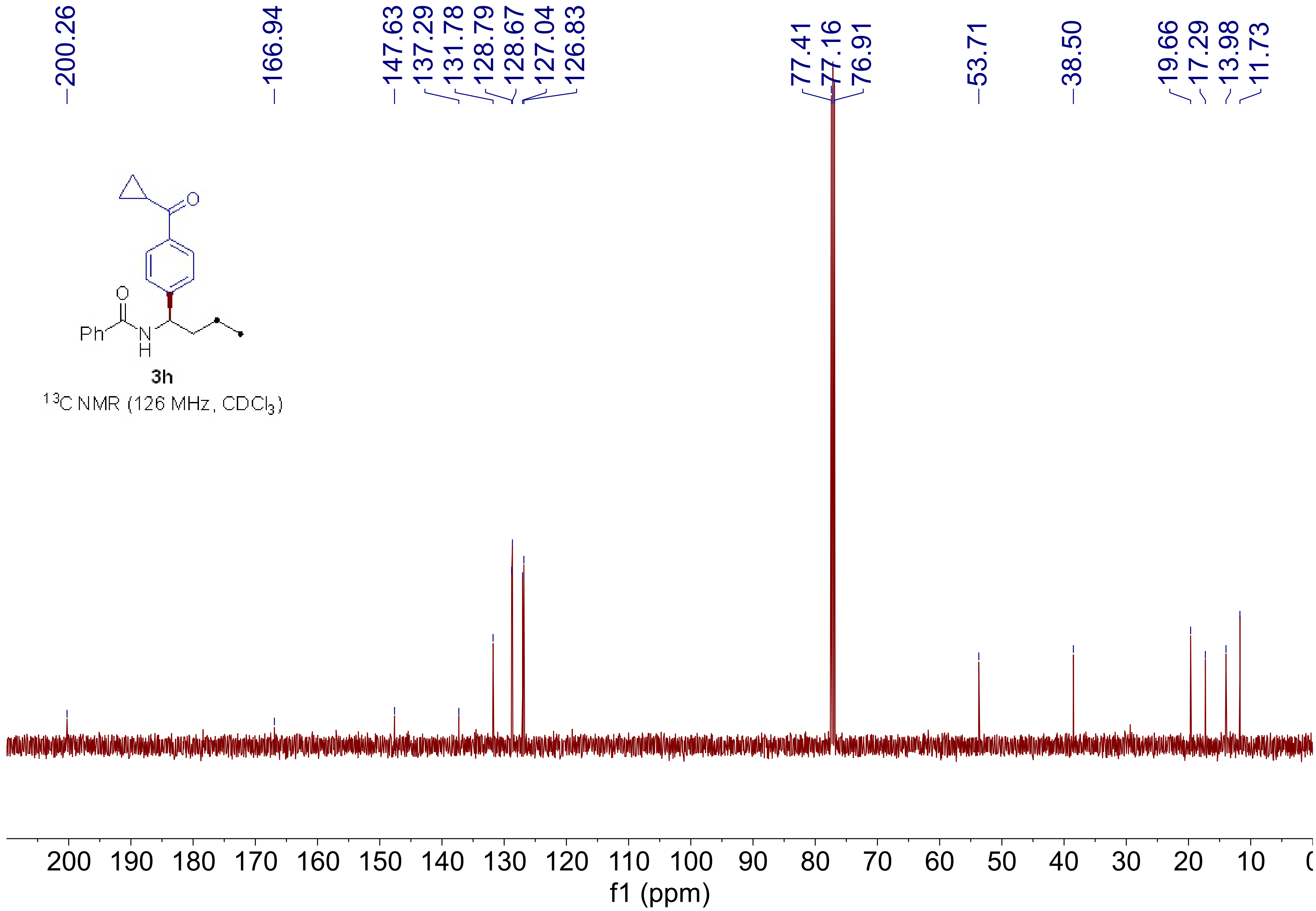
200.26
166.94
147.63
137.29
131.78
128.79
128.67
127.04
126.83
77.41
77.16
76.91
53.71
38.50
19.66
17.29
13.98
11.73
O
O
Ph
N
H
3h
^{13}C NMR (126 MHz, $CDCl_3$)
200 190 180 170 160 150 140 130 120 110 100 90 80 70 60 50 40 30 20 10
f1 (ppm)

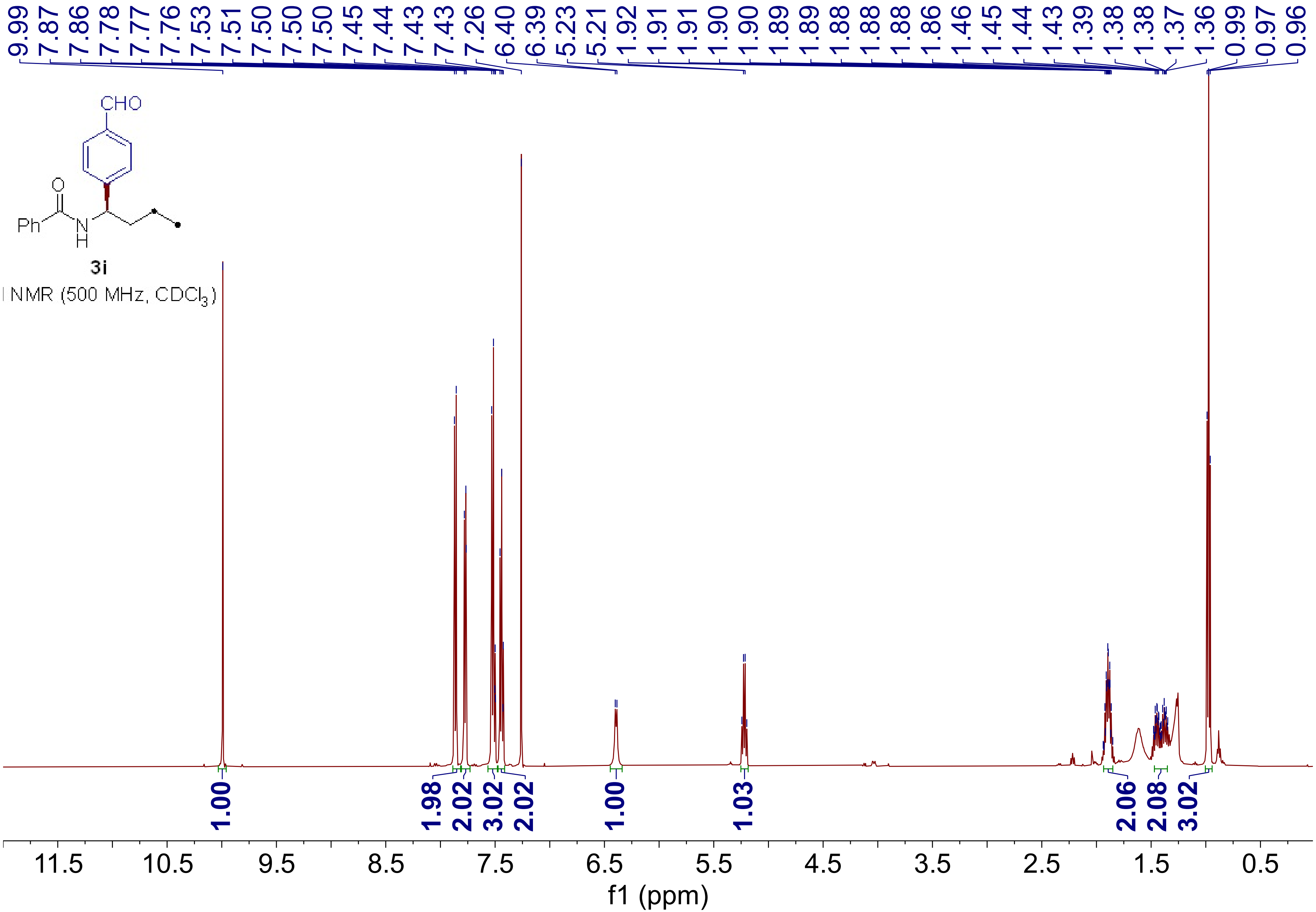
9.99 7.87 7.86 7.78 7.77 7.76 7.53 7.51 7.50 7.50 7.50 7.45 7.44 7.43 7.43 7.26 6.40 6.39 5.23 5.21 1.92 1.91 1.91 1.90 1.90 1.89 1.89 1.88 1.88 1.88 1.86 1.46 1.45 1.44 1.43 1.39 1.38 1.38 1.37 1.36 0.99 0.97 0.96
CHO
O
Ph
N
H
3i
NMR (500 MHz, CDCl3)
1.00
1.98
2.02
3.02
2.02
1.00
1.03
2.06
2.08
3.02
11.5
10.5
9.5
8.5
7.5
6.5
5.5
4.5
3.5
2.5
1.5
0.5
f1 (ppm)

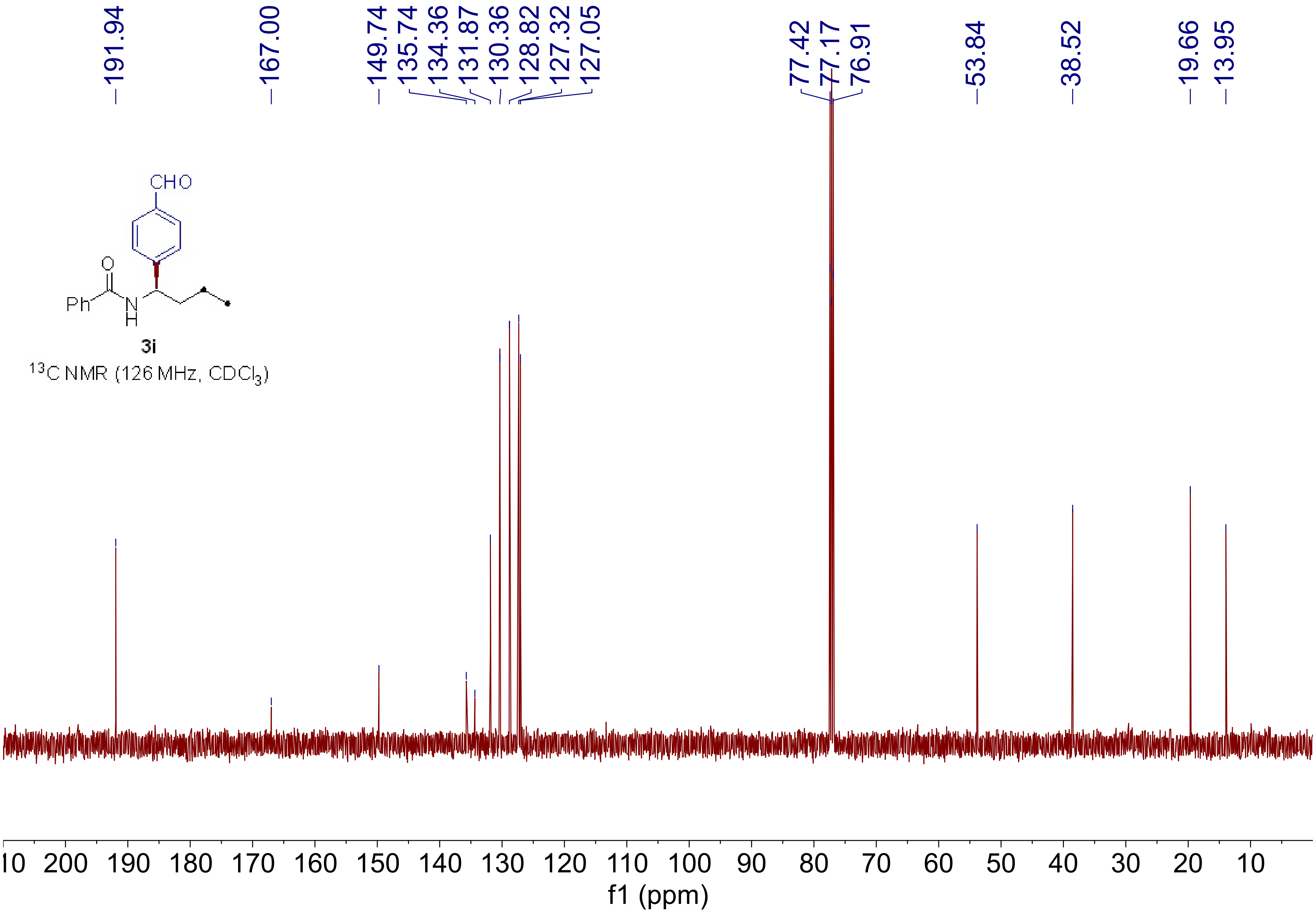
191.94
167.00
149.74
135.74
134.36
131.87
130.36
128.82
127.32
127.05
77.42
77.17
76.91
53.84
38.52
19.66
13.95
CHO
O
Ph
N
H
3i
^{13}C NMR (126 MHz, $CDCl_3$)
10 200 190 180 170 160 150 140 130 120 110 100 90 80 70 60 50 40 30 20 10
f1 (ppm)

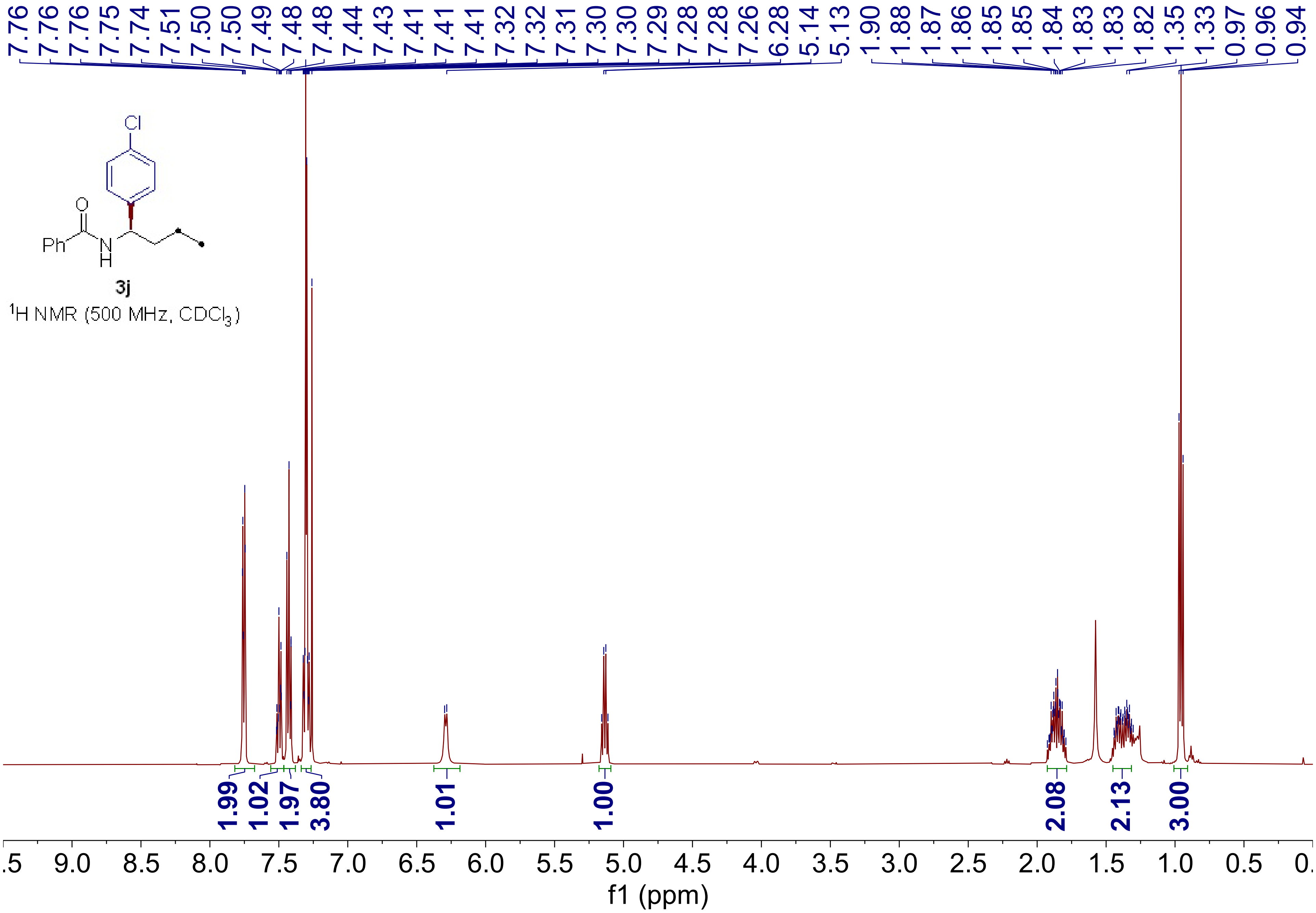
7.76 7.76 7.76 7.75 7.74 7.51 7.50 7.50 7.49 7.48 7.48 7.44 7.43 7.41 7.41 7.41 7.32 7.32 7.31 7.30 7.30 7.29 7.28 7.28 7.26 6.28 5.14 5.13 1.90 1.88 1.87 1.86 1.85 1.85 1.84 1.83 1.83 1.82 1.35 1.33 0.97 0.96 0.94
Cl
O
Ph
N
H
3j
1H NMR (500 MHz, CDCl3)
1.99
1.02
1.97
3.80
1.01
1.00
2.08
2.13
3.00
f1 (ppm)

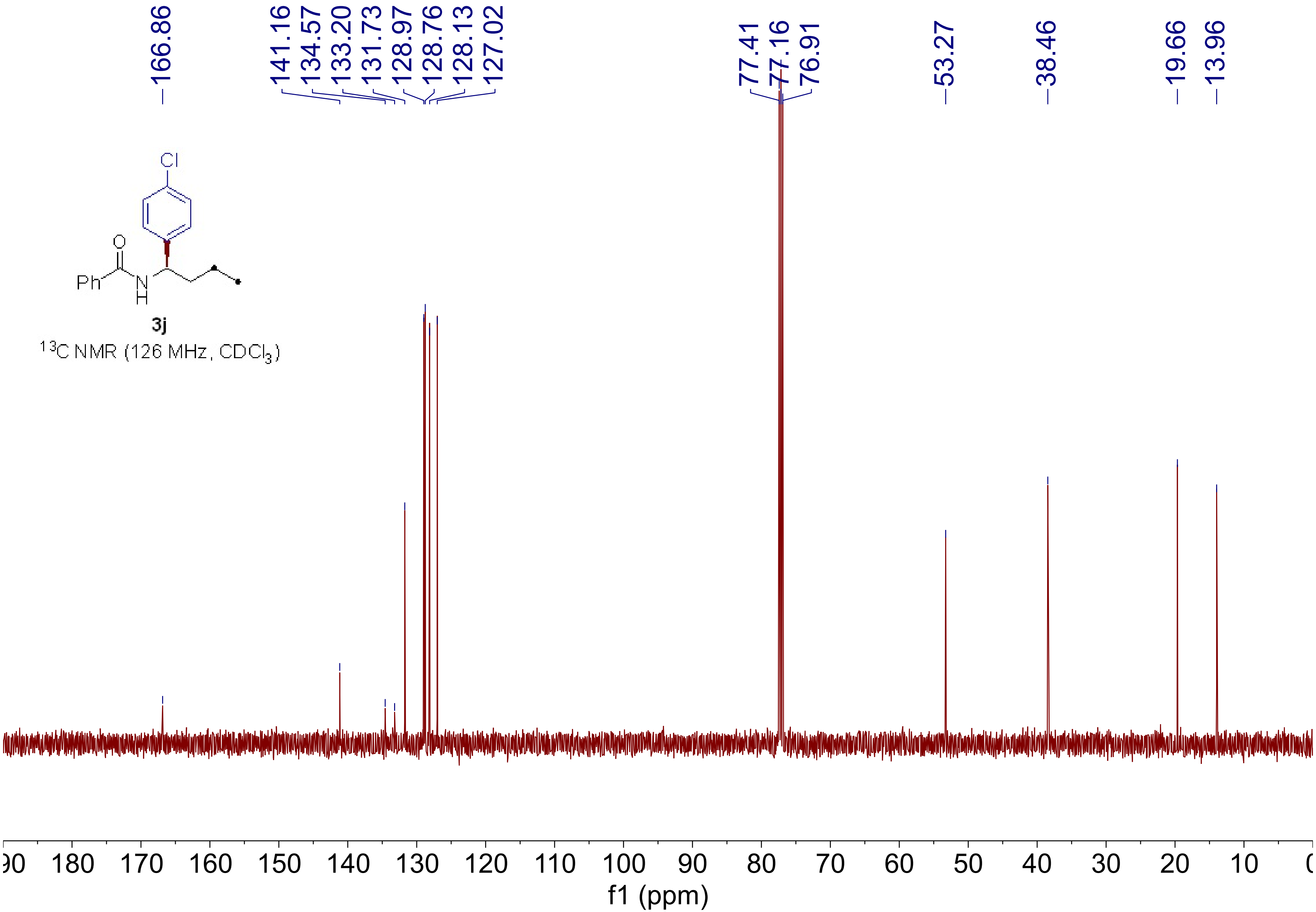
166.86
141.16
134.57
133.20
131.73
128.97
128.76
128.13
127.02
77.41
77.16
76.91
53.27
38.46
19.66
13.96
Cl
O
Ph
N
H
3j
^{13}C NMR (126 MHz, CDCl$_3$)
180 170 160 150 140 130 120 110 100 90 80 70 60 50 40 30 20 10
f1 (ppm)

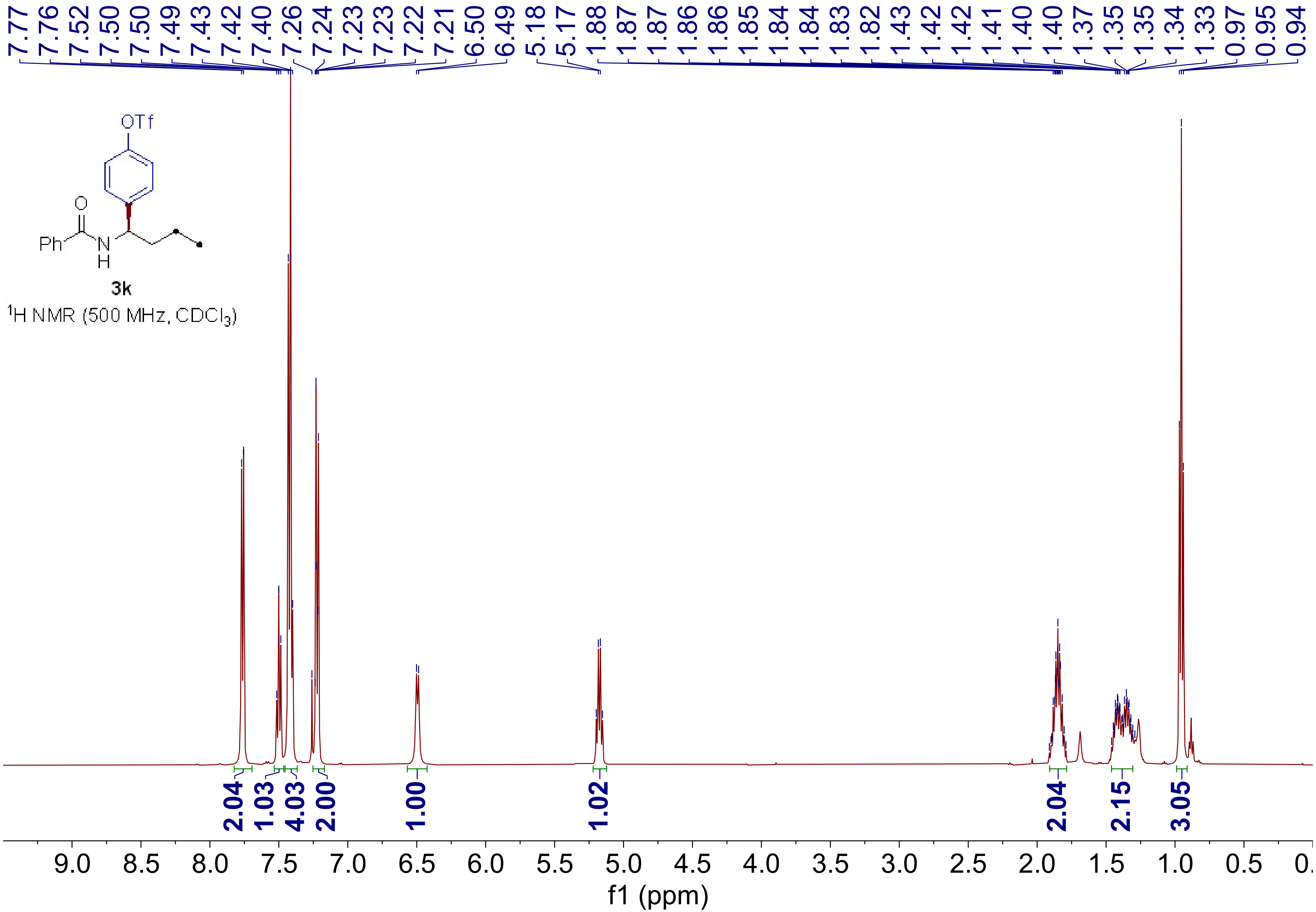
7.77 7.76 7.52 7.50 7.50 7.49 7.43 7.42 7.40 7.26 7.24 7.23 7.23 7.22 7.21 6.50 6.49 5.18 5.17 1.88 1.87 1.87 1.86 1.86 1.85 1.84 1.84 1.83 1.82 1.43 1.42 1.42 1.41 1.40 1.40 1.37 1.35 1.35 1.34 1.33 0.97 0.95 0.94
OTf
O
Ph
N
H
3k
1H NMR (500 MHz, CDCl3)
2.04 1.03 4.03 2.00 1.00 1.02 2.04 2.15 3.05
9.0 8.5 8.0 7.5 7.0 6.5 6.0 5.5 5.0 4.5 4.0 3.5 3.0 2.5 2.0 1.5 1.0 0.5 0.
f1 (ppm)

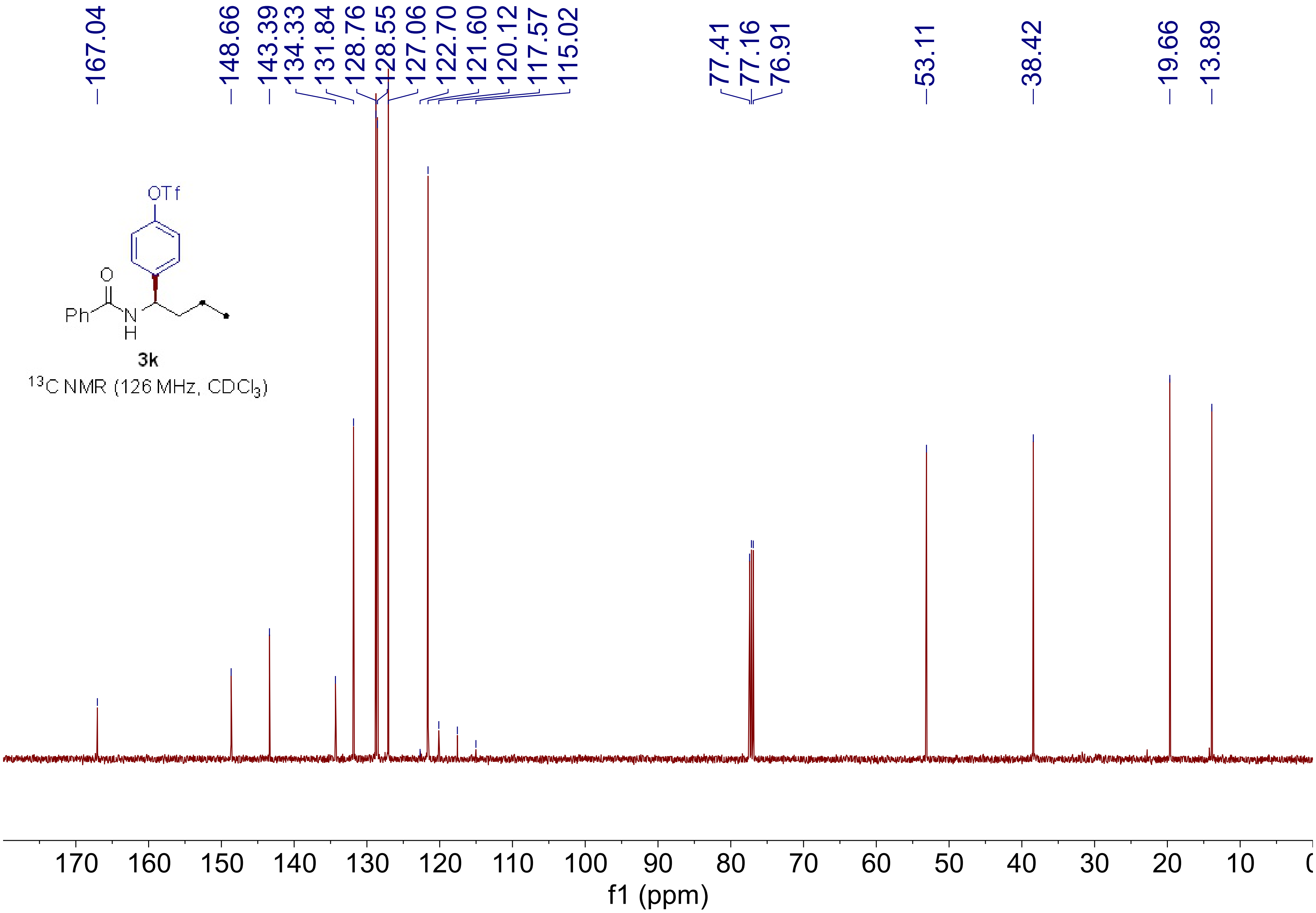
167.04
148.66
143.39
134.33
131.84
128.76
128.55
127.06
122.70
121.60
120.12
117.57
115.02
77.41
77.16
76.91
53.11
38.42
19.66
13.89
OTf
O
Ph
N
H
3k
^{13}C NMR (126 MHz, CDCl$_3$)
170 160 150 140 130 120 110 100 90 80 70 60 50 40 30 20 10
f1 (ppm)

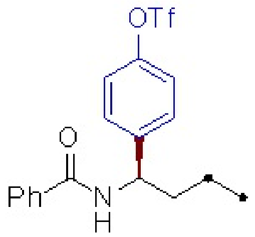

3k

^{19}F NMR (471 MHz, $CDCl_3$)

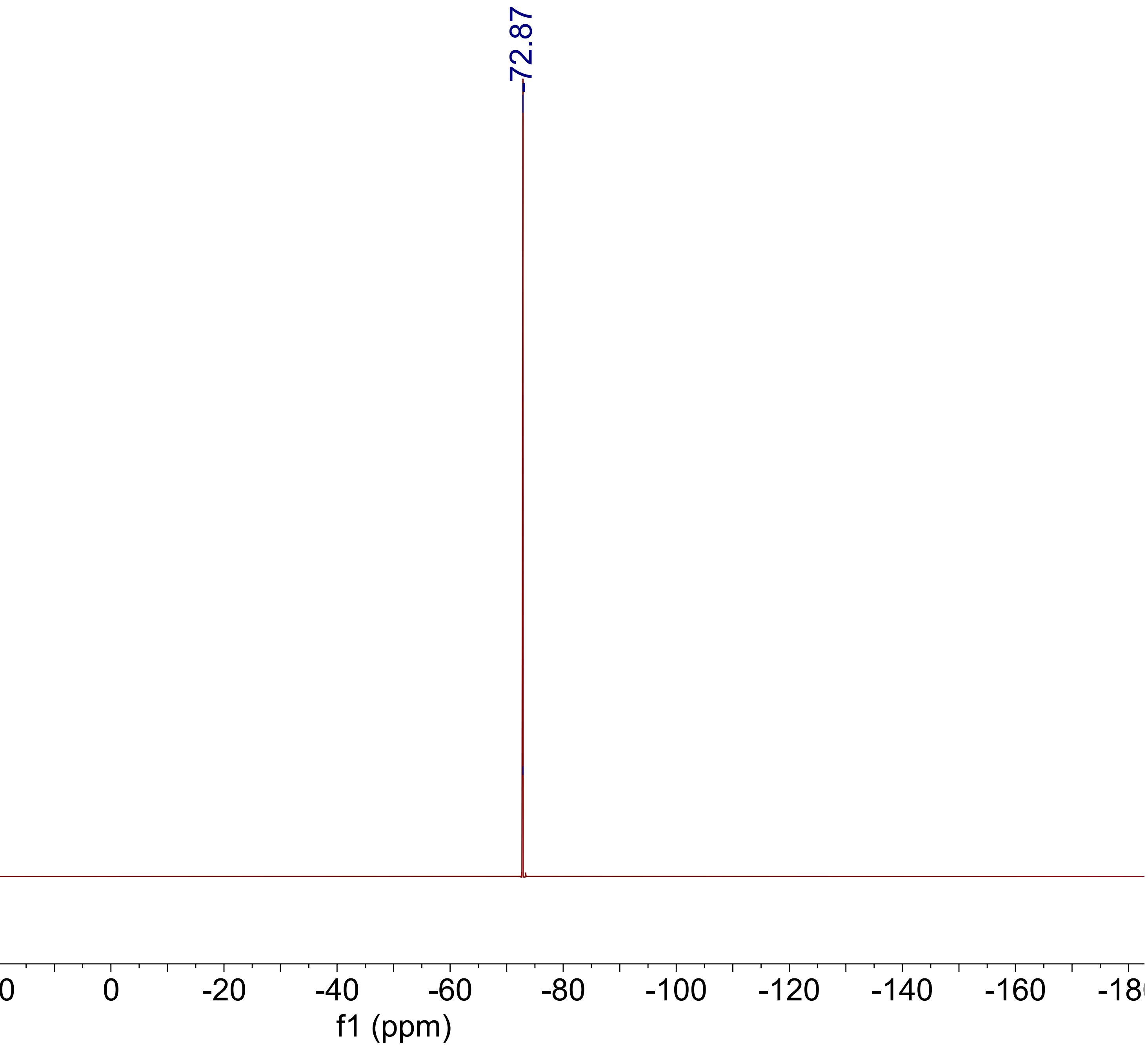

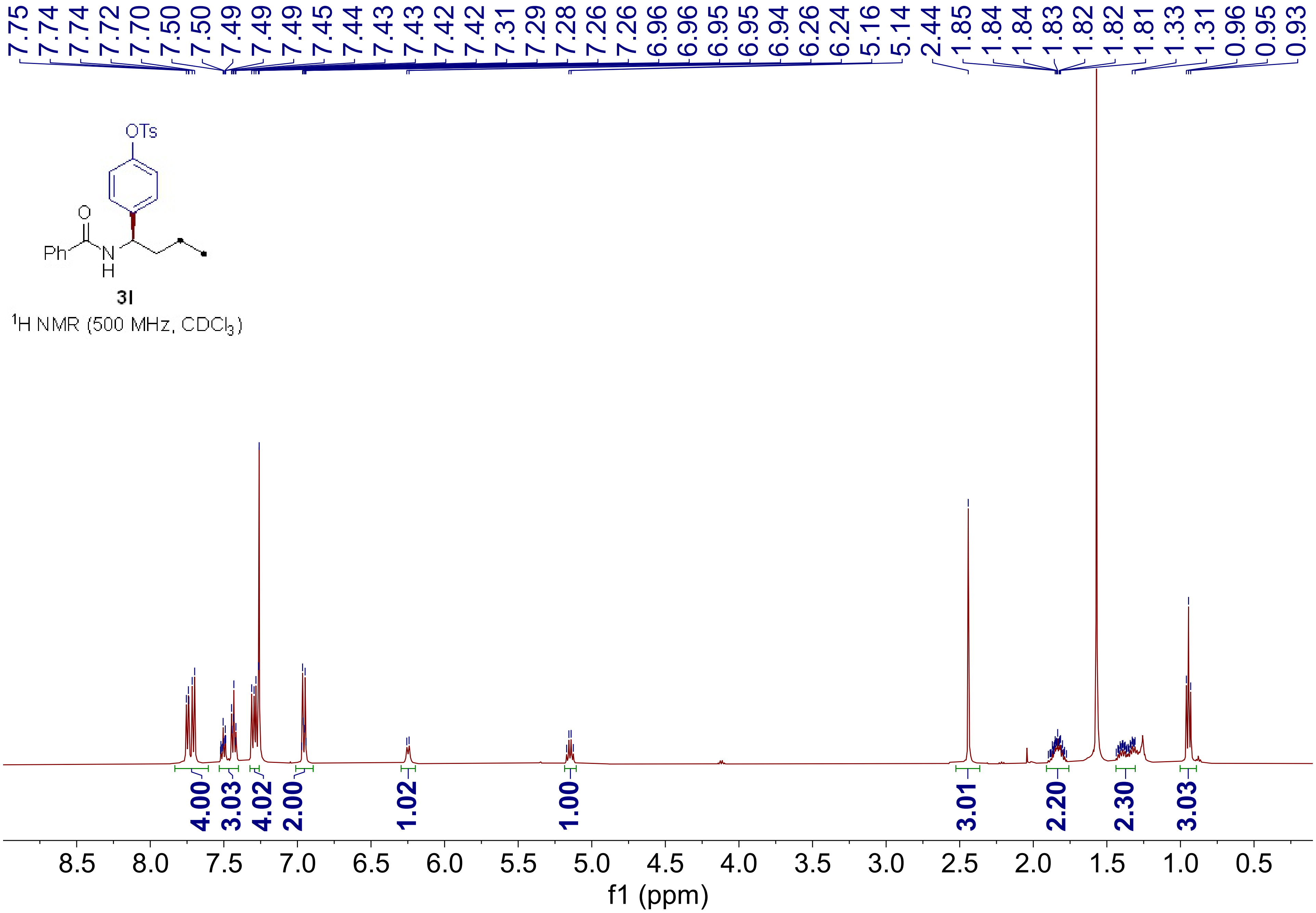

7.75 7.74 7.74 7.72 7.70 7.50 7.50 7.49 7.49 7.49 7.45 7.44 7.43 7.43 7.42 7.42 7.31 7.29 7.28 7.26 7.26 6.96 6.96 6.95 6.95 6.94 6.26 6.24 5.16 5.14 2.44 1.85 1.84 1.84 1.83 1.82 1.82 1.81 1.33 1.31 0.96 0.95 0.93
OTs
O
Ph
N
H
3l
^{1}H NMR (500 MHz, CDCl$_3$)
4.00 3.03 4.02 2.00 1.02 1.00 3.01 2.20 2.30 3.03
8.5 8.0 7.5 7.0 6.5 6.0 5.5 5.0 4.5 4.0 3.5 3.0 2.5 2.0 1.5 1.0 0.5
f1 (ppm)

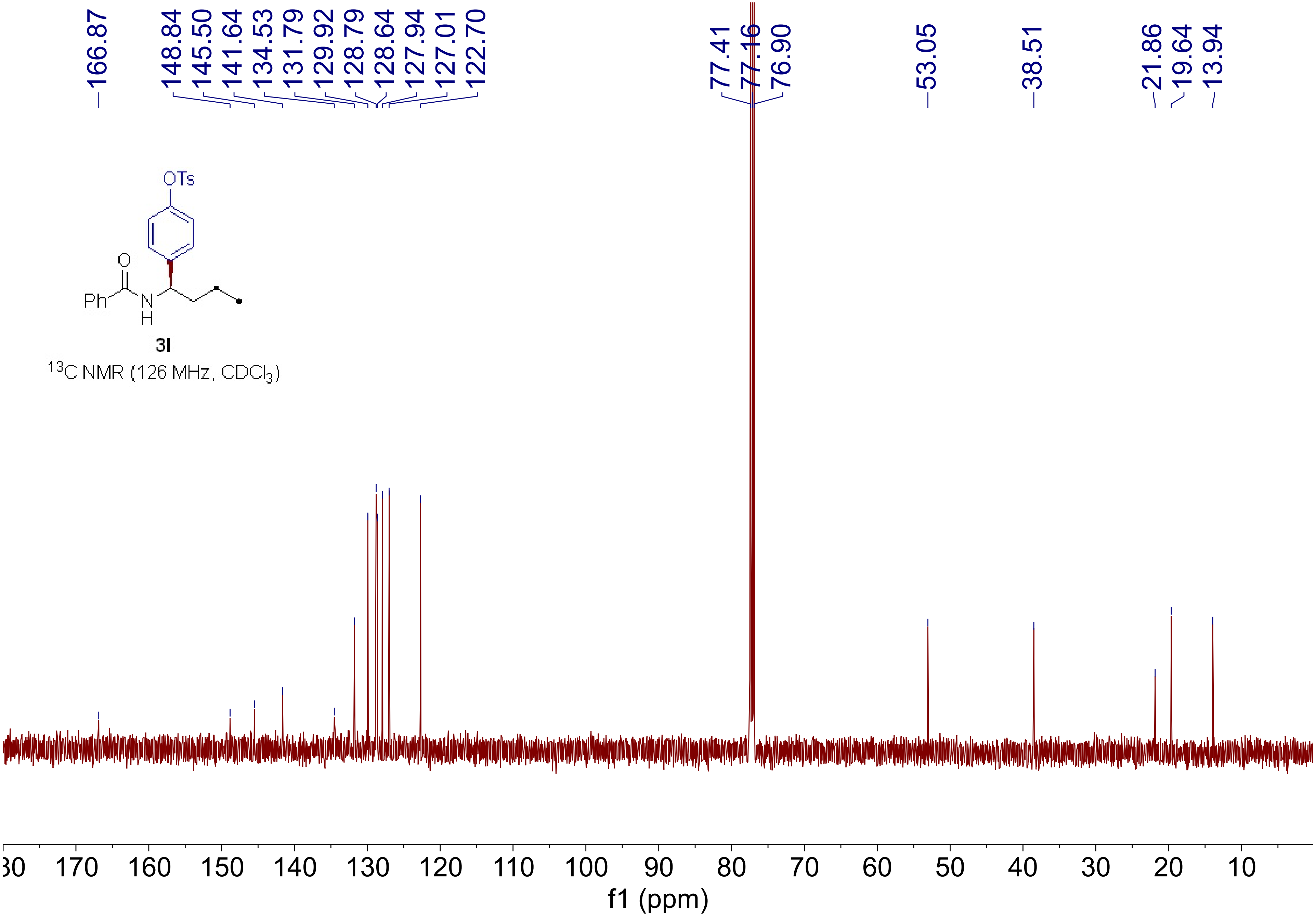
166.87
148.84
145.50
141.64
134.53
131.79
129.92
128.79
128.64
127.94
127.01
122.70
77.41
77.16
76.90
53.05
38.51
21.86
19.64
13.94
OTs
O
Ph
N
H
3l
^{13}C NMR (126 MHz, CDCl$_3$)
170
160
150
140
130
120
110
100
90
80
70
60
50
40
30
20
10
f1 (ppm)

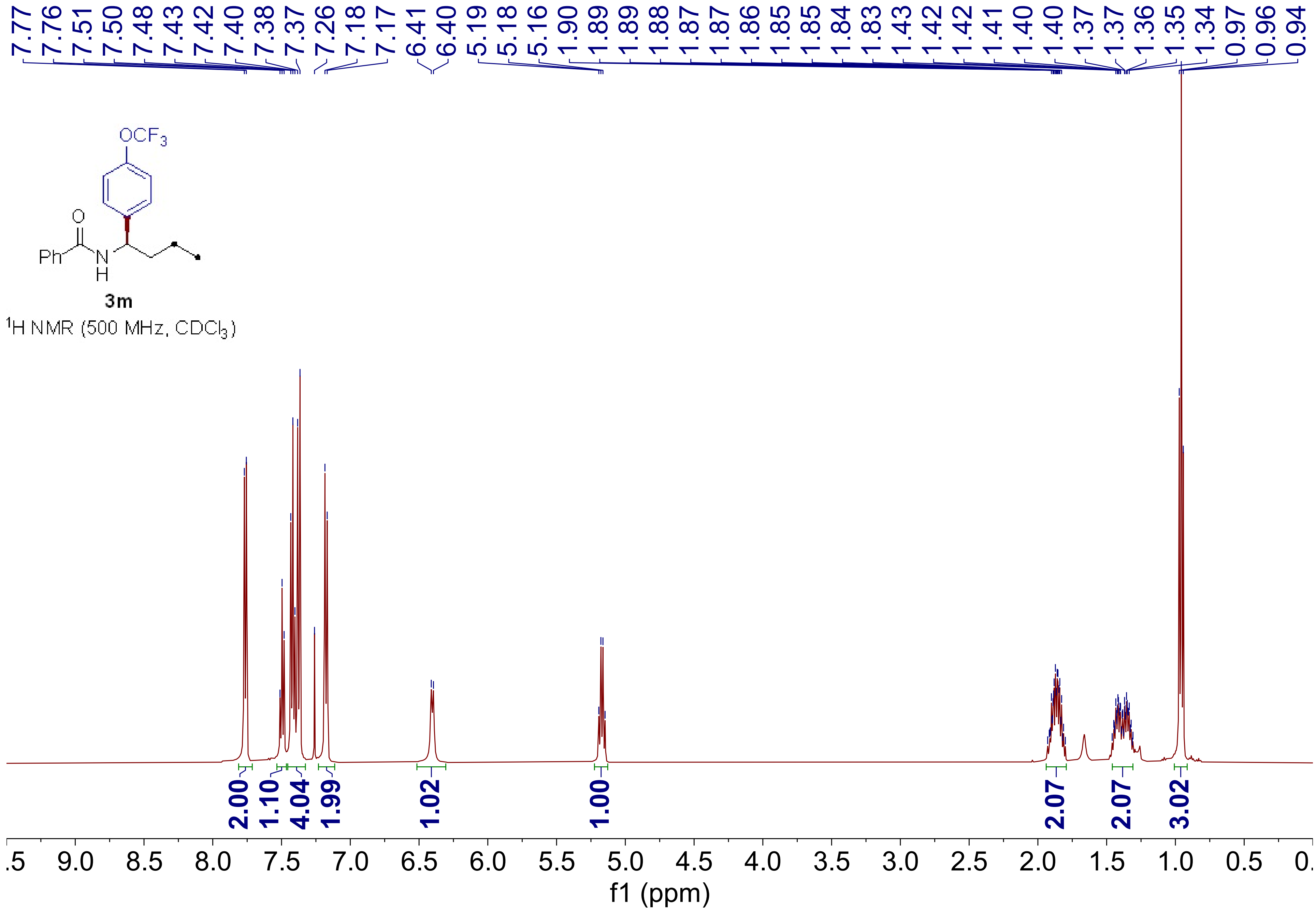
7.77 7.76 7.51 7.50 7.48 7.43 7.42 7.40 7.38 7.37 7.26 7.18 7.17 6.41 6.40 5.19 5.18 5.16 1.90 1.89 1.89 1.88 1.87 1.87 1.86 1.85 1.85 1.84 1.83 1.43 1.42 1.42 1.41 1.40 1.40 1.37 1.37 1.36 1.35 1.34 0.97 0.96 0.94
OCF3
O
Ph
N
H
3m
1H NMR (500 MHz, CDCl3)
2.00 1.10 4.04 1.99 1.02 1.00 2.07 2.07 3.02
.5 9.0 8.5 8.0 7.5 7.0 6.5 6.0 5.5 5.0 4.5 4.0 3.5 3.0 2.5 2.0 1.5 1.0 0.5 0.
f1 (ppm)

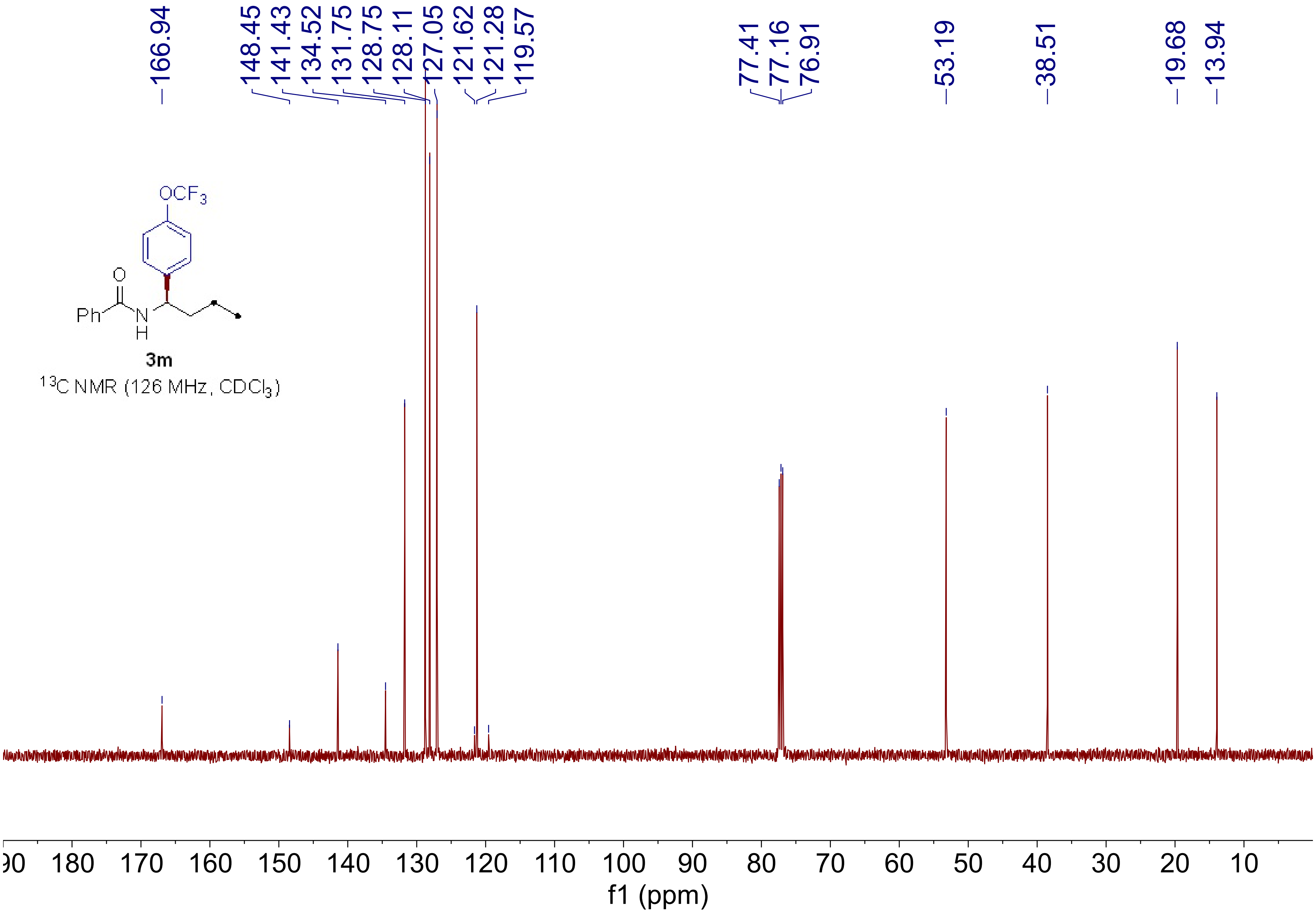
166.94
148.45
141.43
134.52
131.75
128.75
128.11
127.05
121.62
121.28
119.57
77.41
77.16
76.91
53.19
38.51
19.68
13.94
OCF3
O
Ph
N
H
3m
13C NMR (126 MHz, CDCl3)
f1 (ppm)

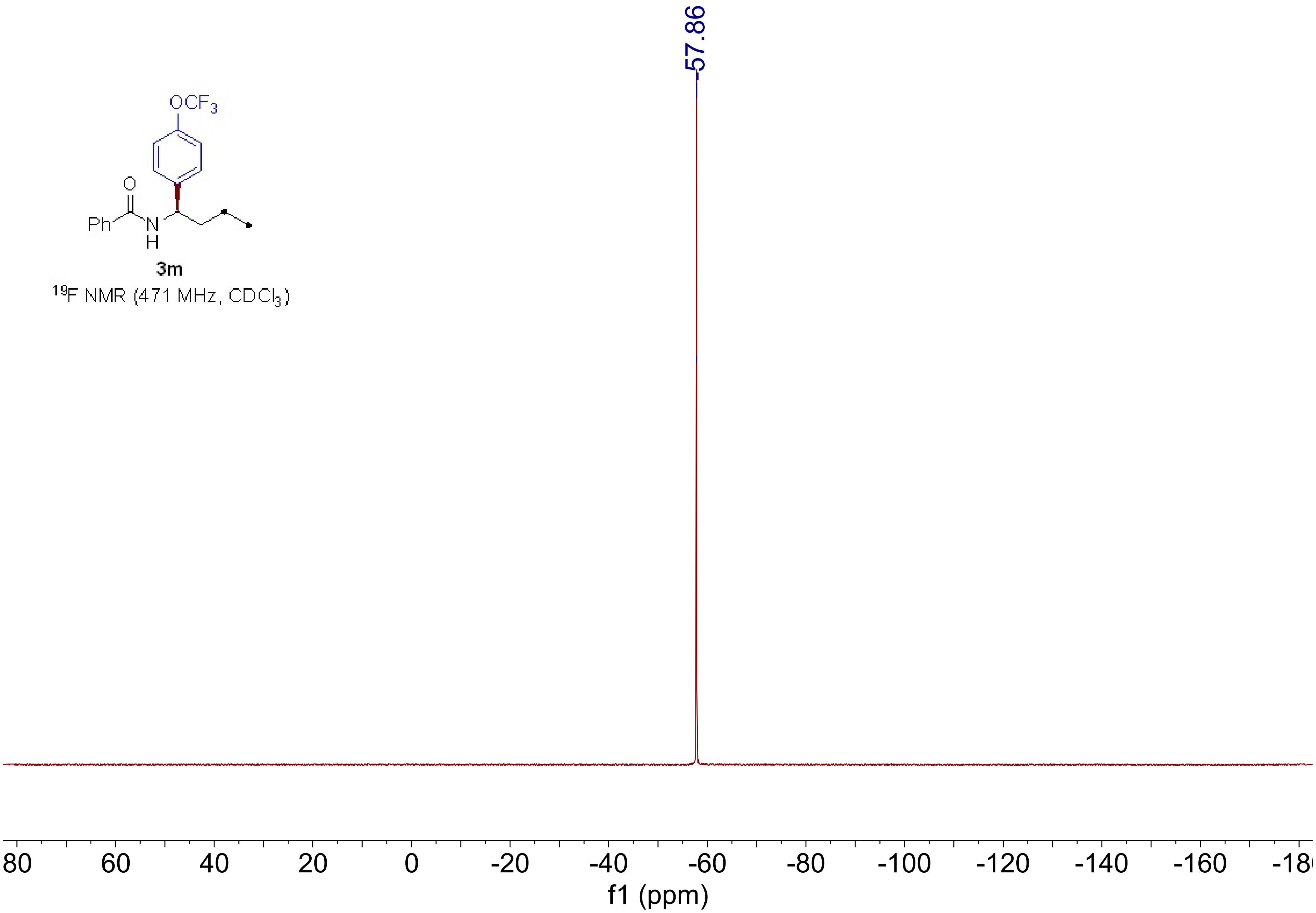
-57.86
OCF3
O
Ph
N
H
3m
19F NMR (471 MHz, CDCl3)
80
60
40
20
0
-20
-40
-60
-80
-100
-120
-140
-160
-18
f1 (ppm)

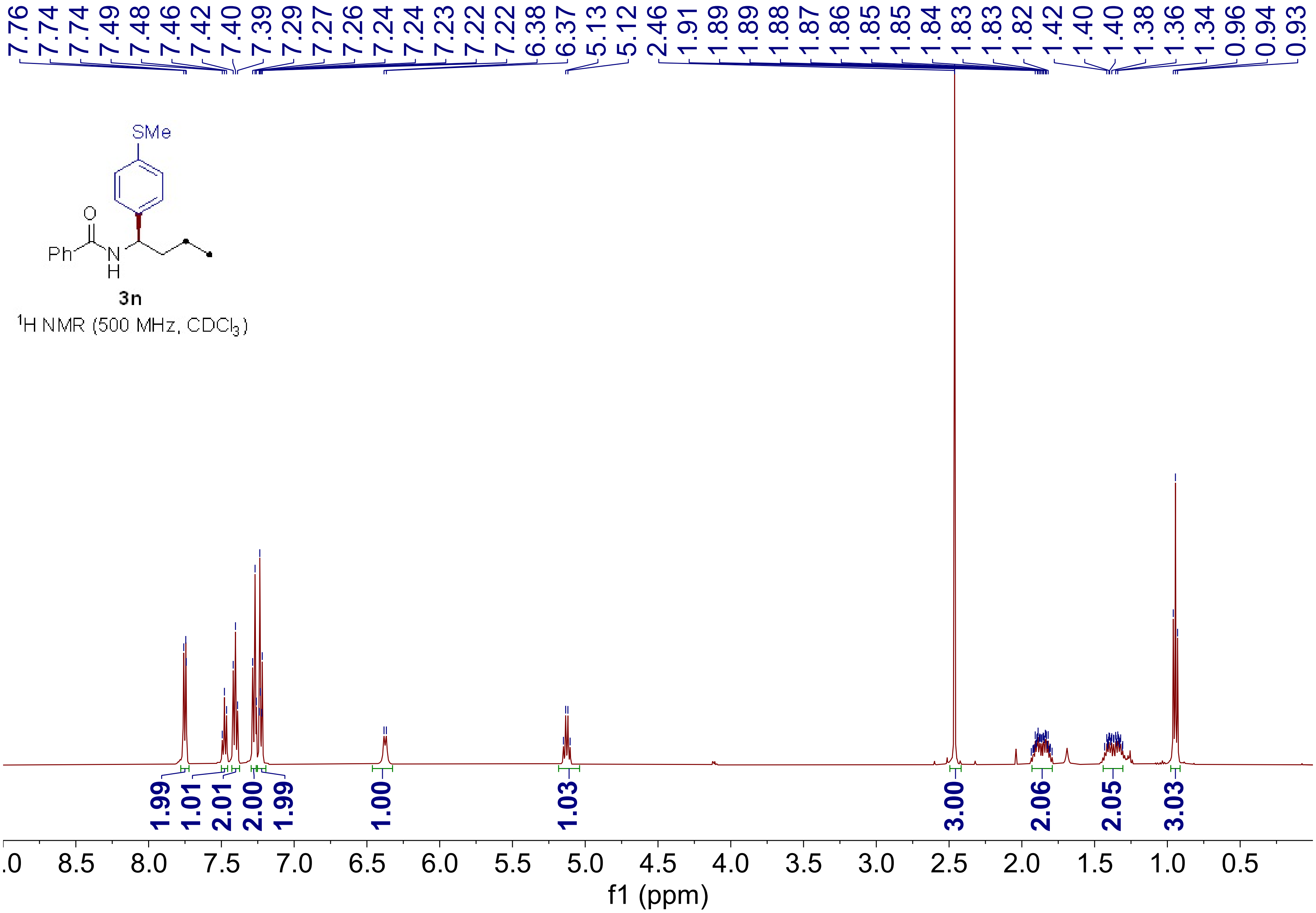
7.76 7.74 7.74 7.49 7.48 7.46 7.42 7.40 7.39 7.29 7.27 7.26 7.24 7.24 7.23 7.22 7.22 6.38 6.37 5.13 5.12 2.46 1.91 1.89 1.89 1.88 1.87 1.86 1.85 1.85 1.84 1.83 1.83 1.82 1.42 1.40 1.40 1.38 1.36 1.34 0.96 0.94 0.93
SMe
O
Ph
N
H
3n
^{1}H NMR (500 MHz, CDCl$_3$)
1.99 1.01 2.01 2.00 1.99 1.00 1.03 3.00 2.06 2.05 3.03
.0 8.5 8.0 7.5 7.0 6.5 6.0 5.5 5.0 4.5 4.0 3.5 3.0 2.5 2.0 1.5 1.0 0.5
f1 (ppm)

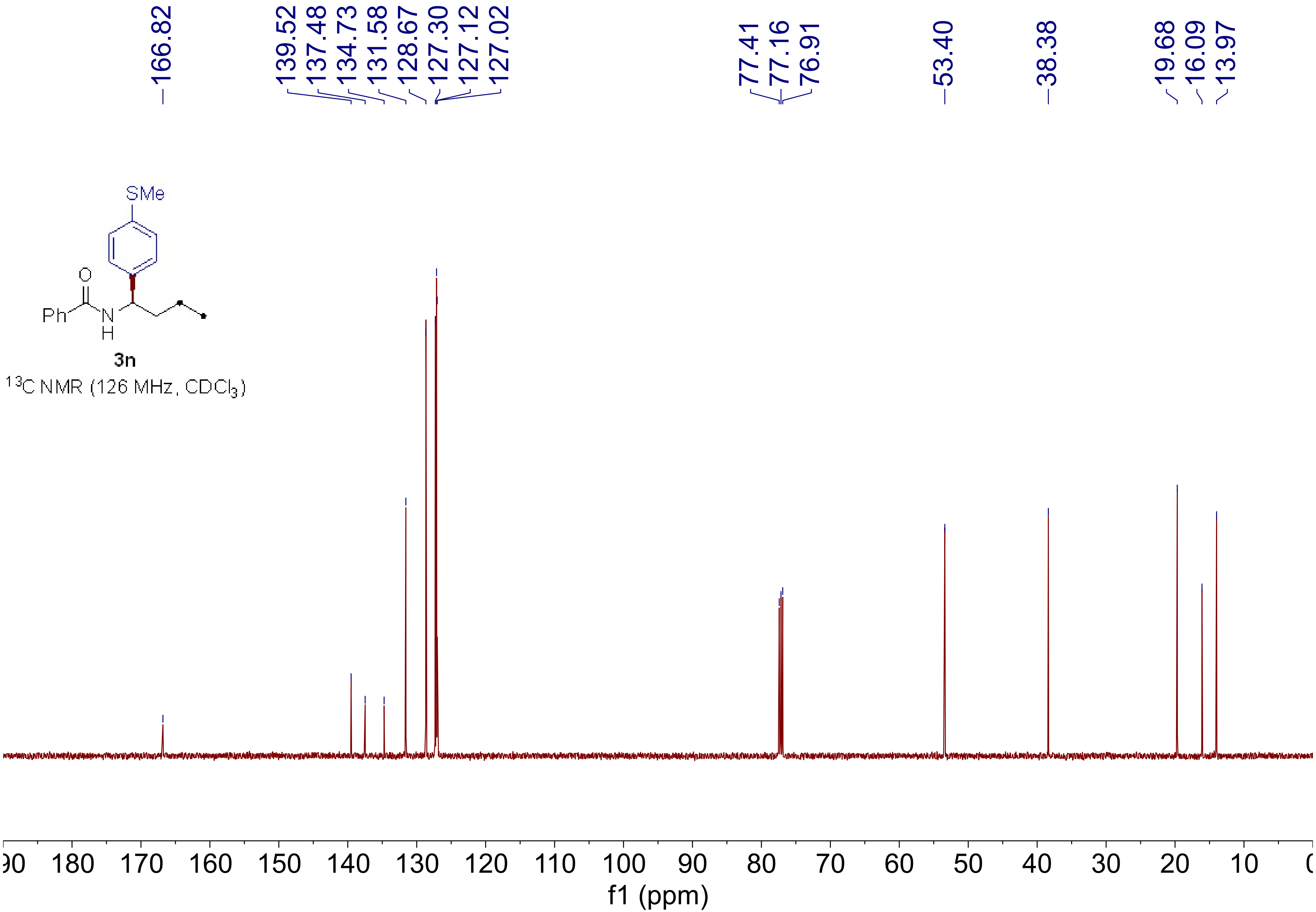
166.82
139.52
137.48
134.73
131.58
128.67
127.30
127.12
127.02
77.41
77.16
76.91
53.40
38.38
19.68
16.09
13.97
SMe
O
Ph
N
H
3n
^{13}C NMR (126 MHz, CDCl$_3$)
180
170
160
150
140
130
120
110
100
90
80
70
60
50
40
30
20
10
f1 (ppm)

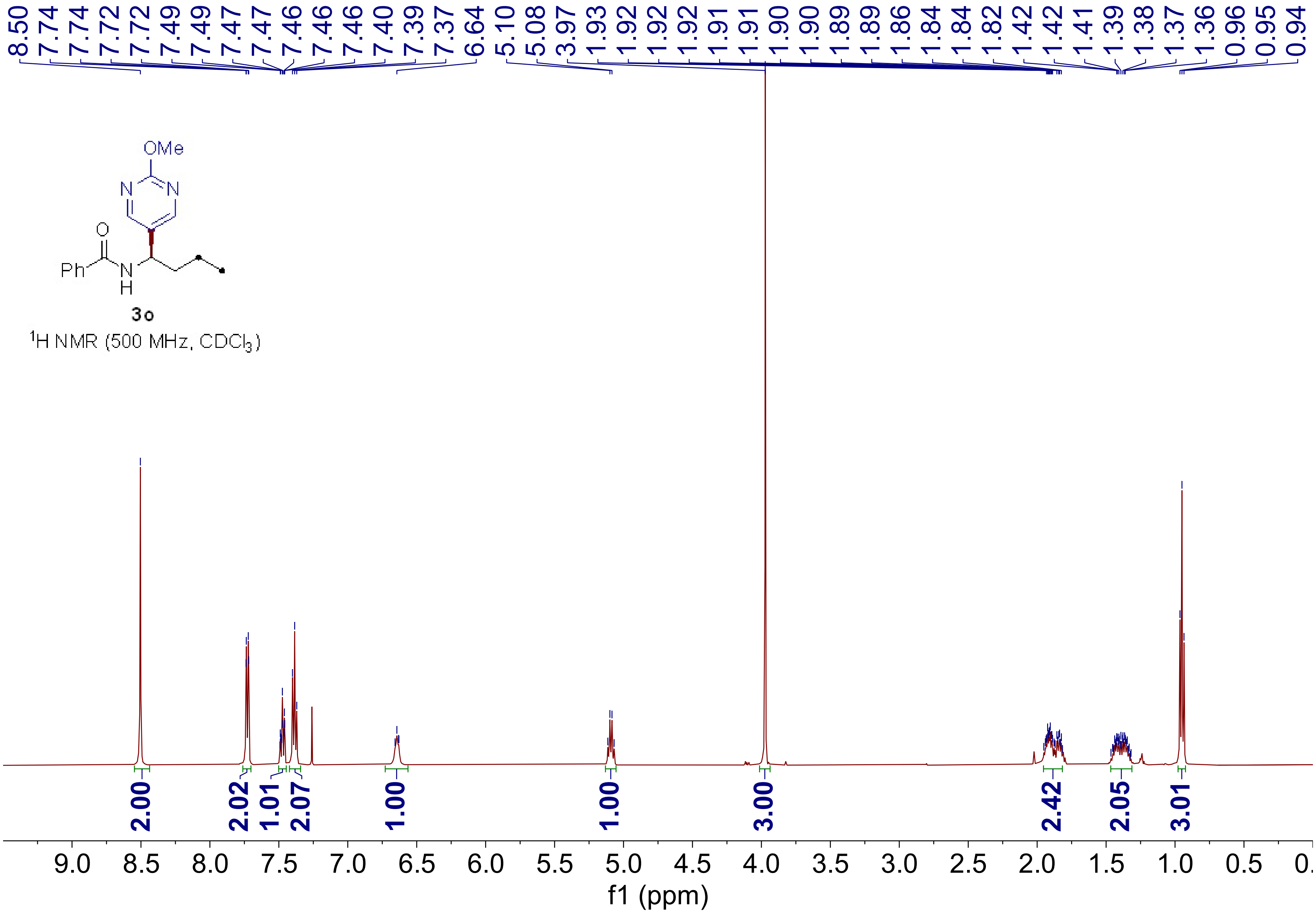
8.50 7.74 7.74 7.72 7.72 7.49 7.49 7.47 7.47 7.46 7.46 7.46 7.40 7.39 7.37 6.64 5.10 5.08 3.97 1.93 1.92 1.92 1.92 1.91 1.91 1.90 1.90 1.89 1.89 1.86 1.84 1.84 1.82 1.42 1.42 1.41 1.39 1.38 1.37 1.36 0.96 0.95 0.94
OMe
N
N
O
Ph
N
H
3o
1H NMR (500 MHz, CDCl3)
2.00 2.02 1.01 2.07 1.00 1.00 3.00 2.42 2.05 3.01
9.0 8.5 8.0 7.5 7.0 6.5 6.0 5.5 5.0 4.5 4.0 3.5 3.0 2.5 2.0 1.5 1.0 0.5
f1 (ppm)

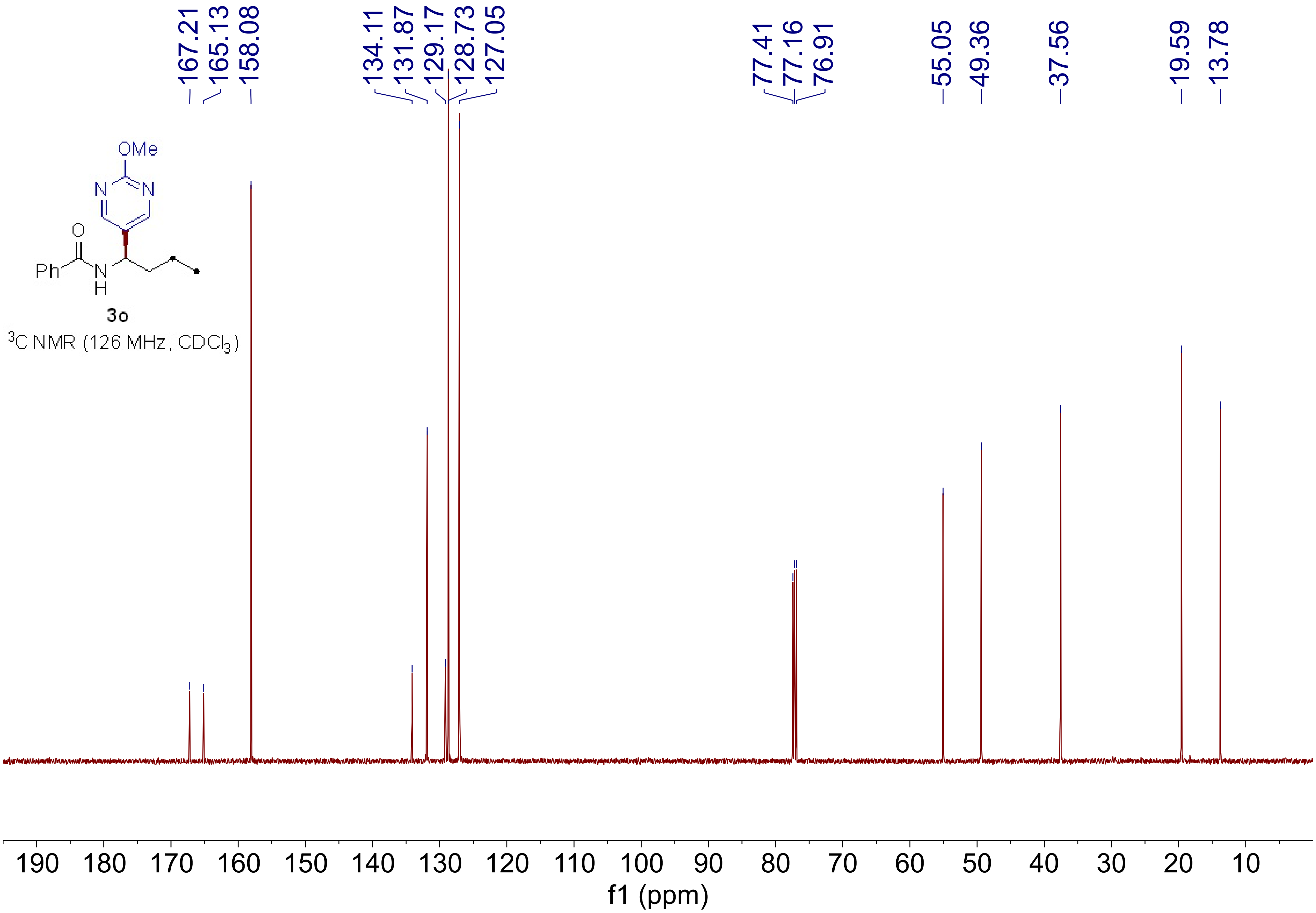

167.21
165.13
158.08
134.11
131.87
129.17
128.73
127.05
77.41
77.16
76.91
55.05
49.36
37.56
19.59
13.78
OMe
N
N
O
Ph
N
H
3o
3C NMR (126 MHz, CDCl3)
190 180 170 160 150 140 130 120 110 100 90 80 70 60 50 40 30 20 10
f1 (ppm)

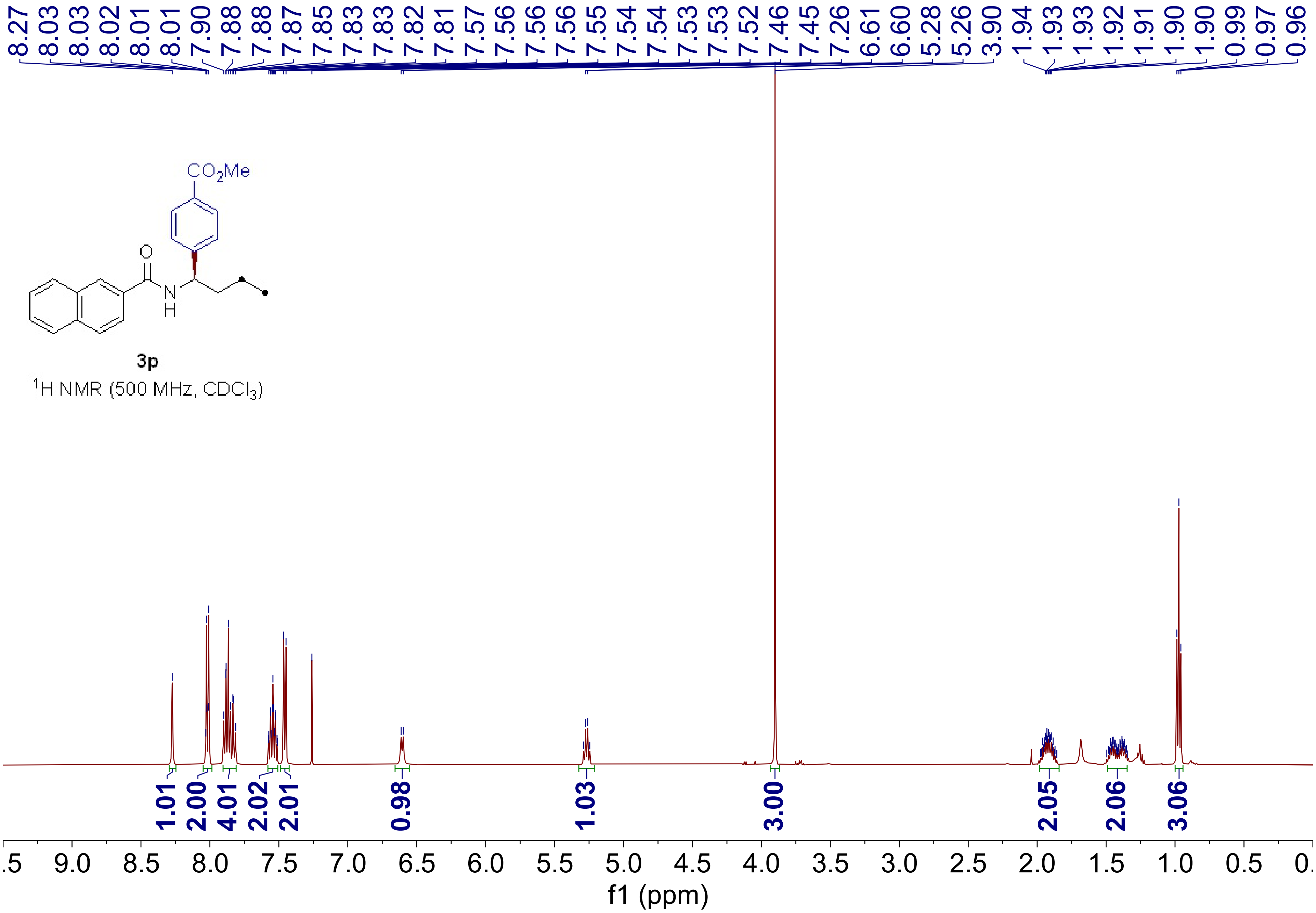
8.27 8.03 8.03 8.02 8.01 8.01 7.90 7.88 7.88 7.87 7.85 7.83 7.83 7.82 7.81 7.57 7.56 7.56 7.56 7.55 7.54 7.54 7.53 7.53 7.52 7.46 7.45 7.26 6.61 6.60 5.28 5.26 3.90 1.94 1.93 1.93 1.92 1.91 1.90 1.90 0.99 0.97 0.96
CO_2Me
O
N
H
3p
^1H NMR (500 MHz, $CDCl_3$)
1.01 2.00 4.01 2.02 2.01 0.98 1.03 3.00 2.05 2.06 3.06
.5 9.0 8.5 8.0 7.5 7.0 6.5 6.0 5.5 5.0 4.5 4.0 3.5 3.0 2.5 2.0 1.5 1.0 0.5 0.
f1 (ppm)

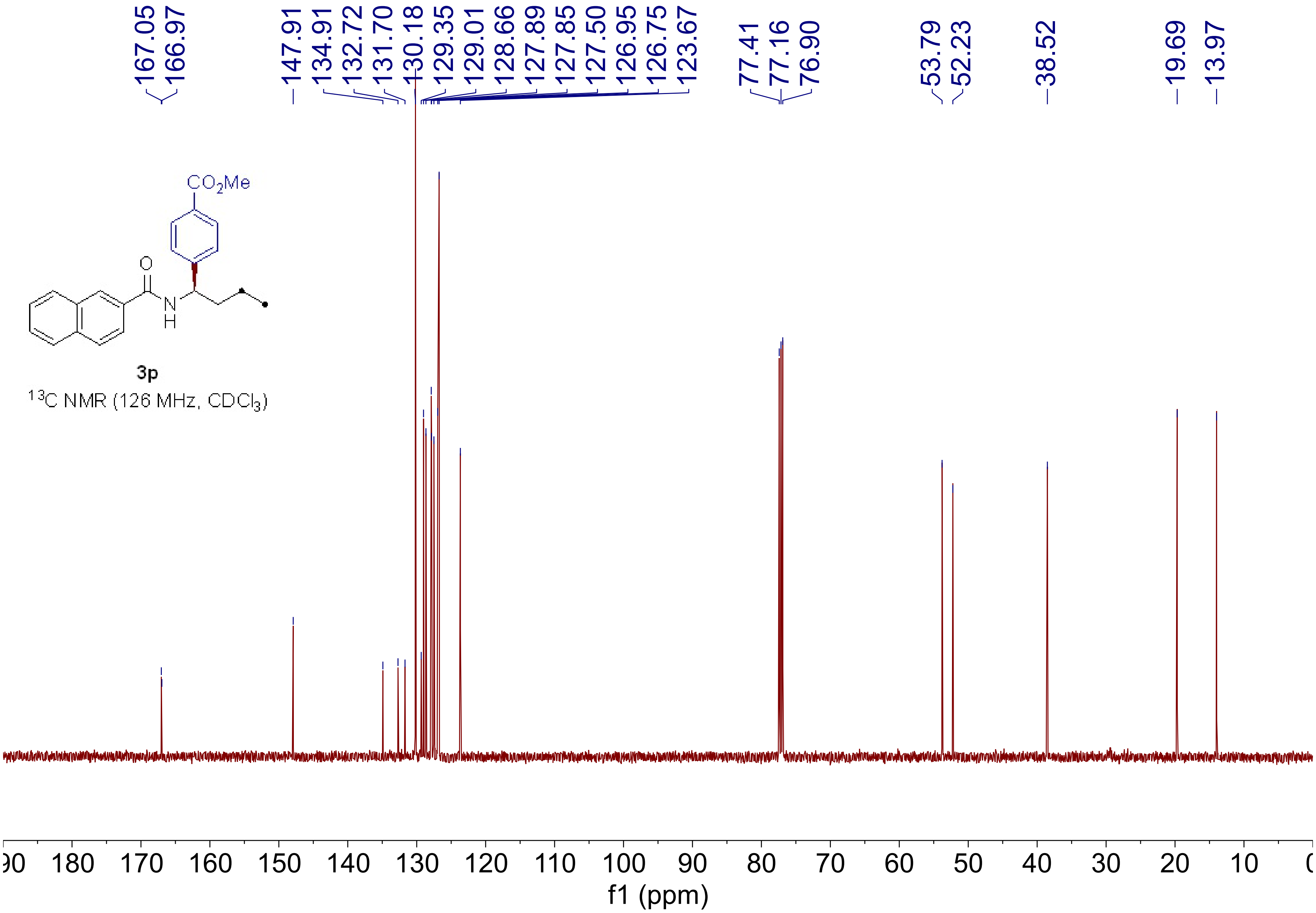

3p

^{13}C NMR (126 MHz, $CDCl_3$)

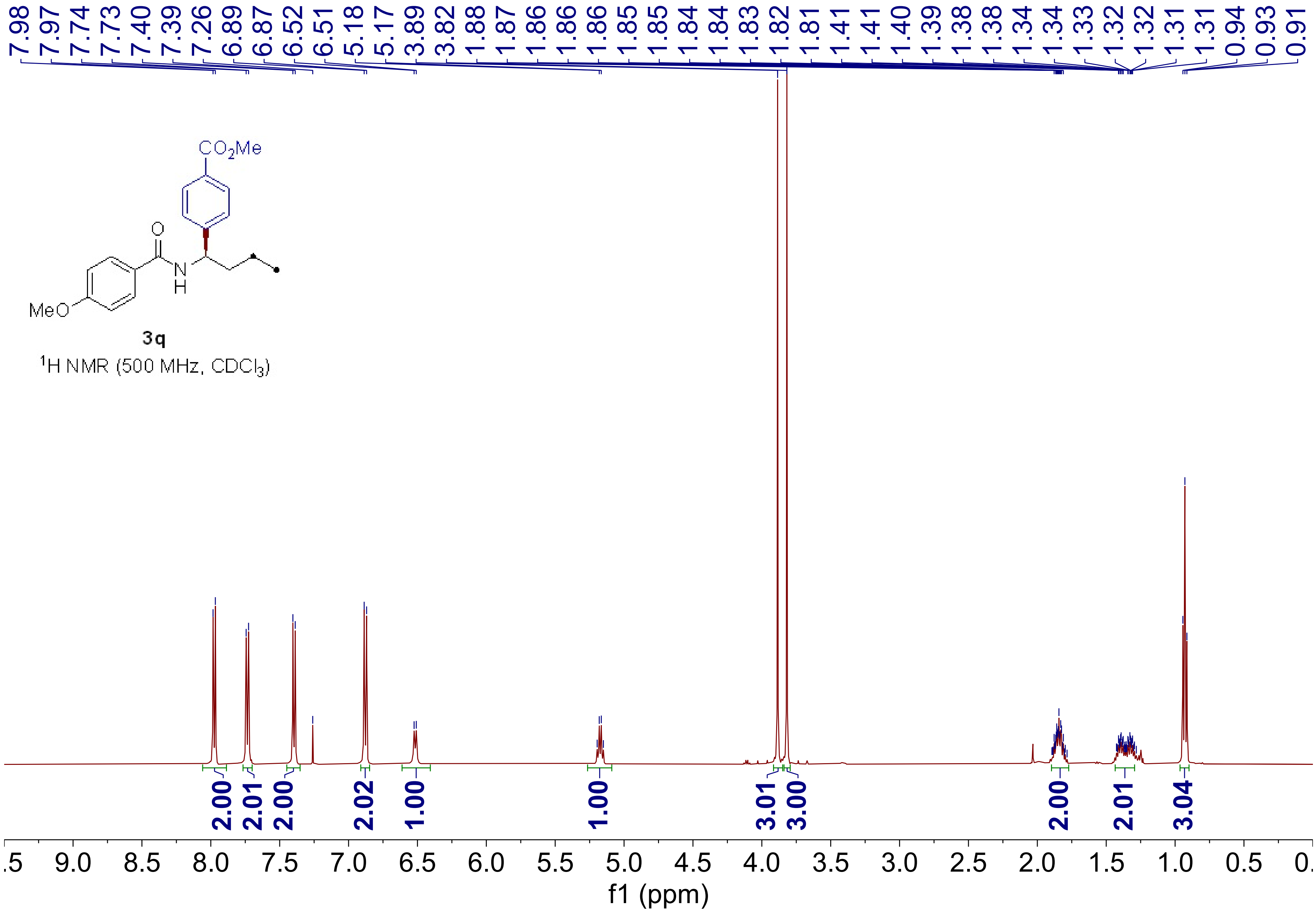
7.98 7.97 7.74 7.73 7.40 7.39 7.26 6.89 6.87 6.52 6.51 5.18 5.17 3.89 3.82 1.88 1.87 1.86 1.86 1.86 1.85 1.85 1.84 1.84 1.83 1.82 1.81 1.41 1.41 1.40 1.39 1.38 1.38 1.34 1.34 1.33 1.32 1.32 1.31 1.31 0.94 0.93 0.91
CO2Me
O
N
H
MeO
3q
1H NMR (500 MHz, CDCl3)
2.00 2.01 2.00 2.02 1.00 1.00 3.01 3.00 2.00 2.01 3.04
.5 9.0 8.5 8.0 7.5 7.0 6.5 6.0 5.5 5.0 4.5 4.0 3.5 3.0 2.5 2.0 1.5 1.0 0.5 0.
f1 (ppm)

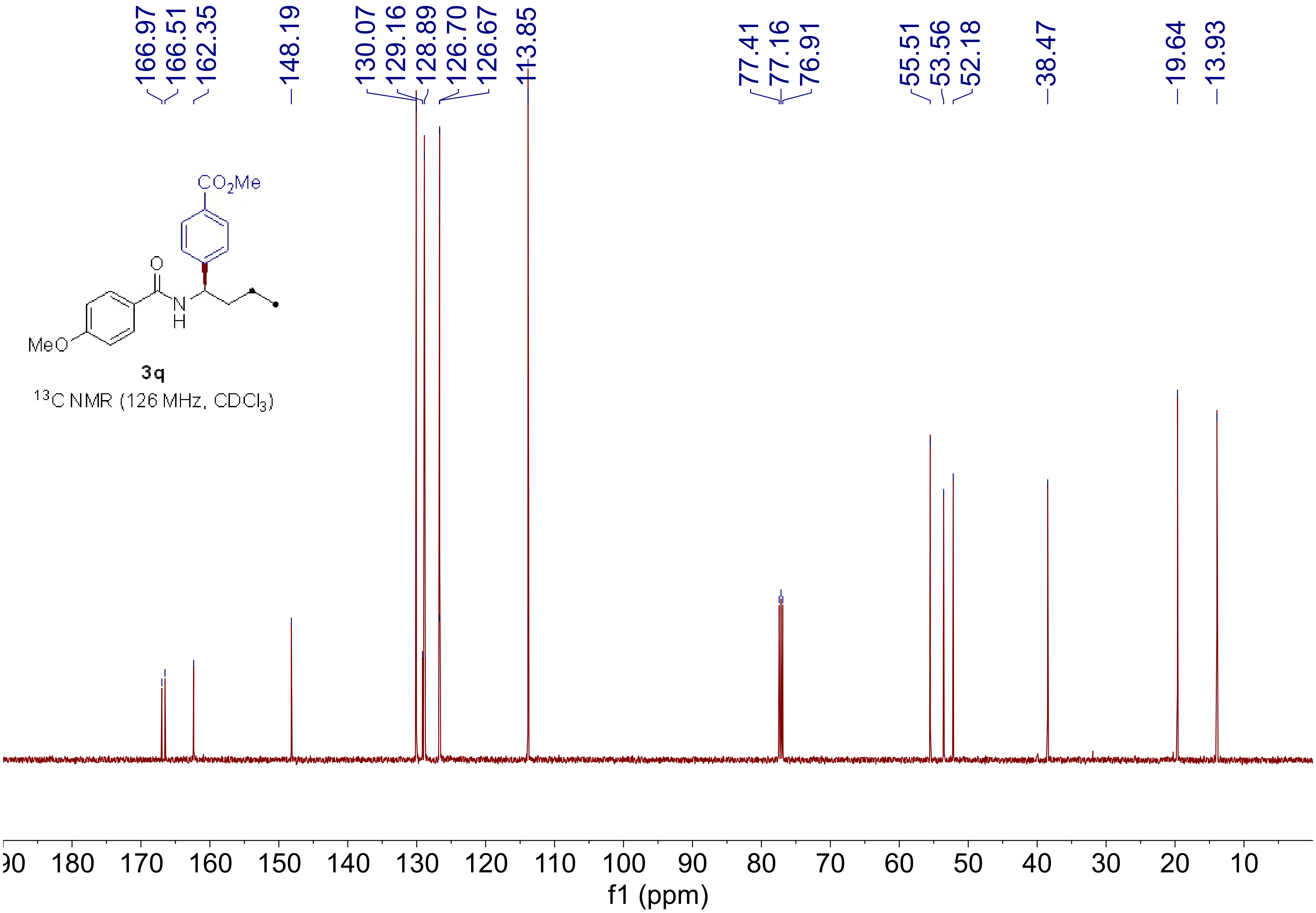
166.97
166.51
162.35
148.19
130.07
129.16
128.89
126.70
126.67
113.85
77.41
77.16
76.91
55.51
53.56
52.18
38.47
19.64
13.93
CO2Me
O
N
H
MeO
3q
13C NMR (126 MHz, CDCl3)
180
170
160
150
140
130
120
110
100
90
80
70
60
50
40
30
20
10
f1 (ppm)

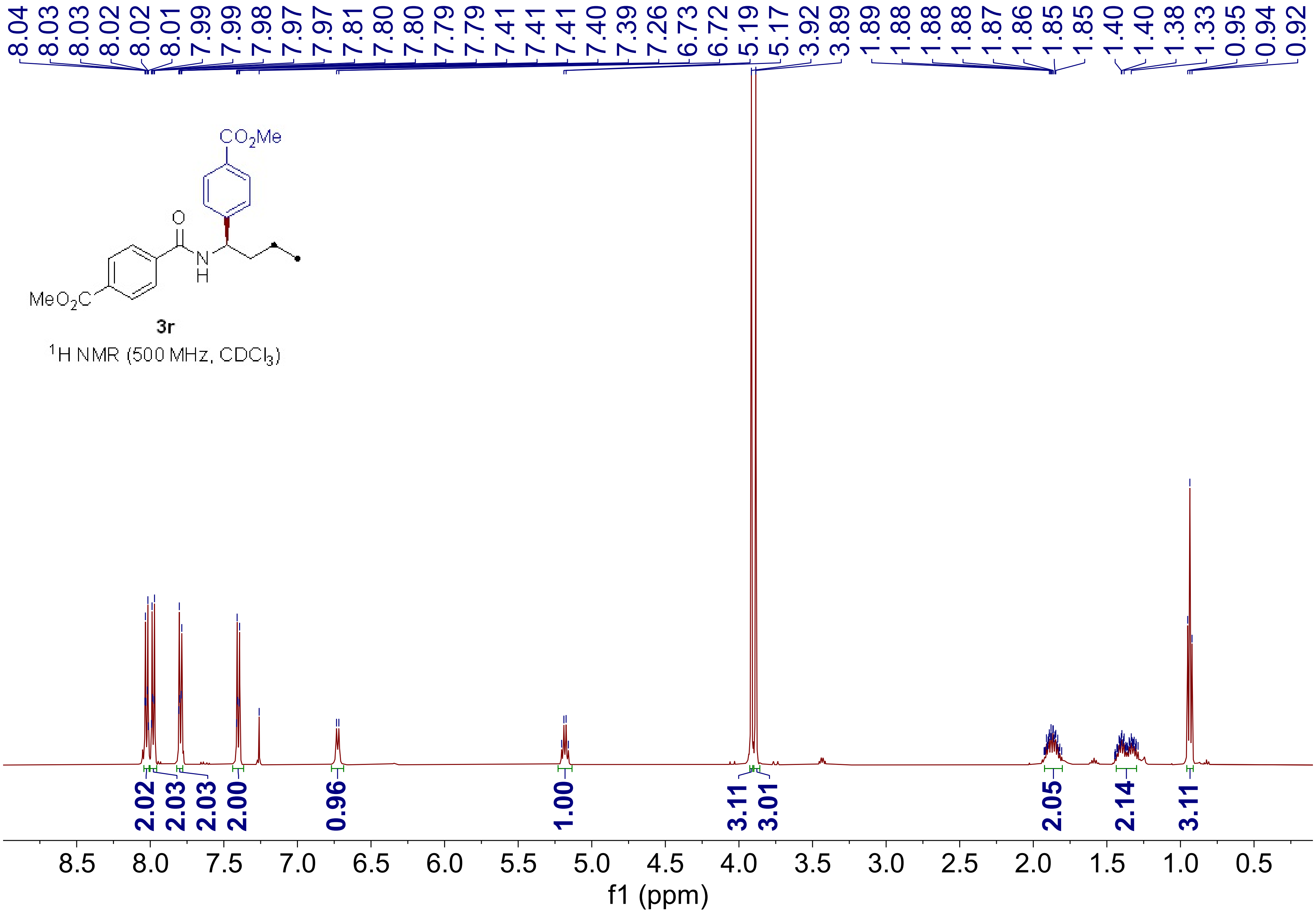
8.04 8.03 8.03 8.02 8.02 8.01 7.99 7.99 7.98 7.97 7.97 7.81 7.80 7.80 7.79 7.79 7.41 7.41 7.41 7.40 7.39 7.26 6.73 6.72 5.19 5.17 3.92 3.89 1.89 1.88 1.88 1.88 1.87 1.86 1.85 1.85 1.40 1.40 1.38 1.33 0.95 0.94 0.92
CO2Me
O
N
H
MeO2C
3r
1H NMR (500 MHz, CDCl3)
2.02 2.03 2.03 2.00 0.96 1.00 3.11 3.01 2.05 2.14 3.11
8.5 8.0 7.5 7.0 6.5 6.0 5.5 5.0 4.5 4.0 3.5 3.0 2.5 2.0 1.5 1.0 0.5
f1 (ppm)

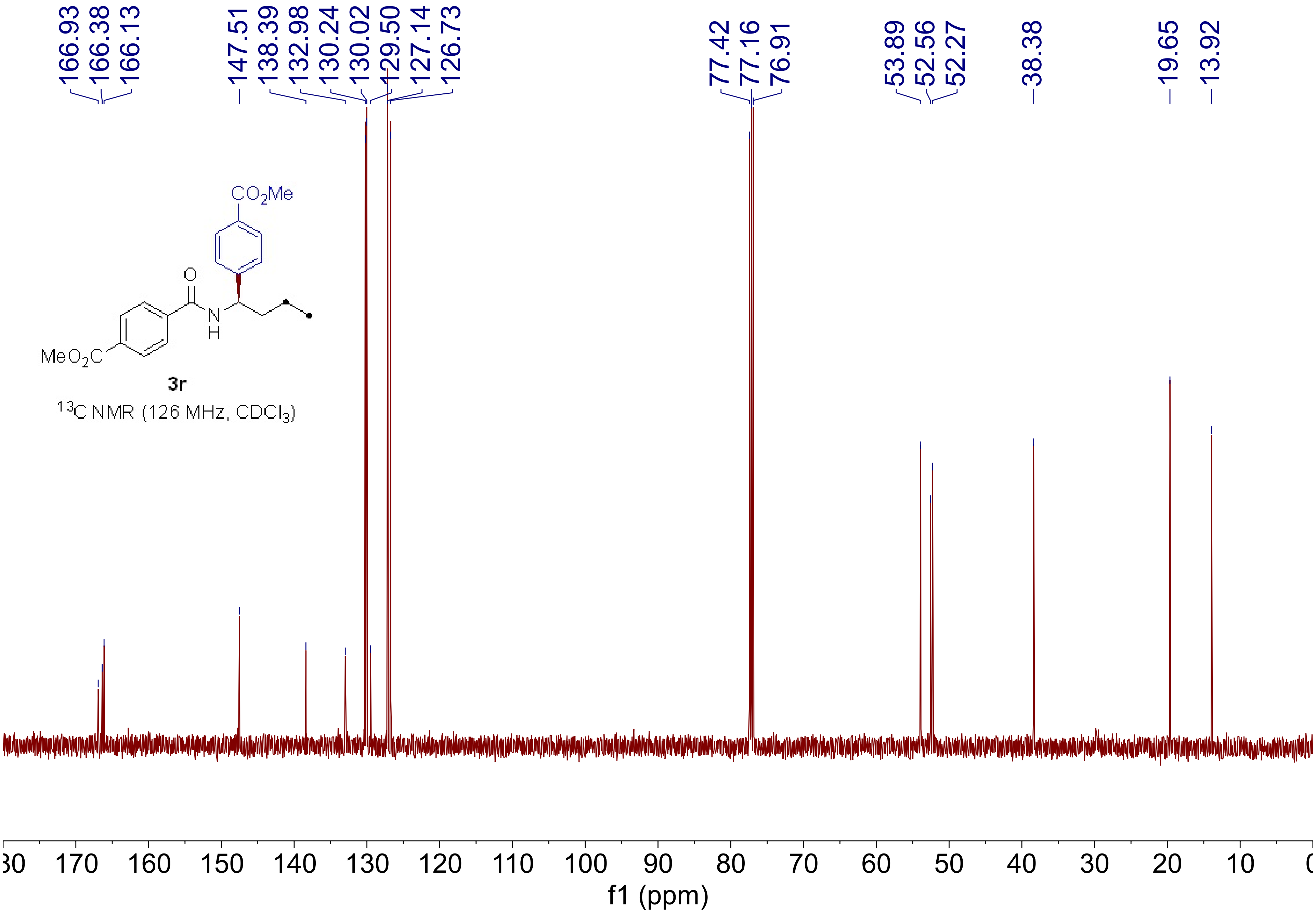

166.93
166.38
166.13
147.51
138.39
132.98
130.24
130.02
129.50
127.14
126.73
77.42
77.16
76.91
53.89
52.56
52.27
38.38
19.65
13.92
CO2Me
O
N
H
MeO2C
3r
13C NMR (126 MHz, CDCl3)
f1 (ppm)

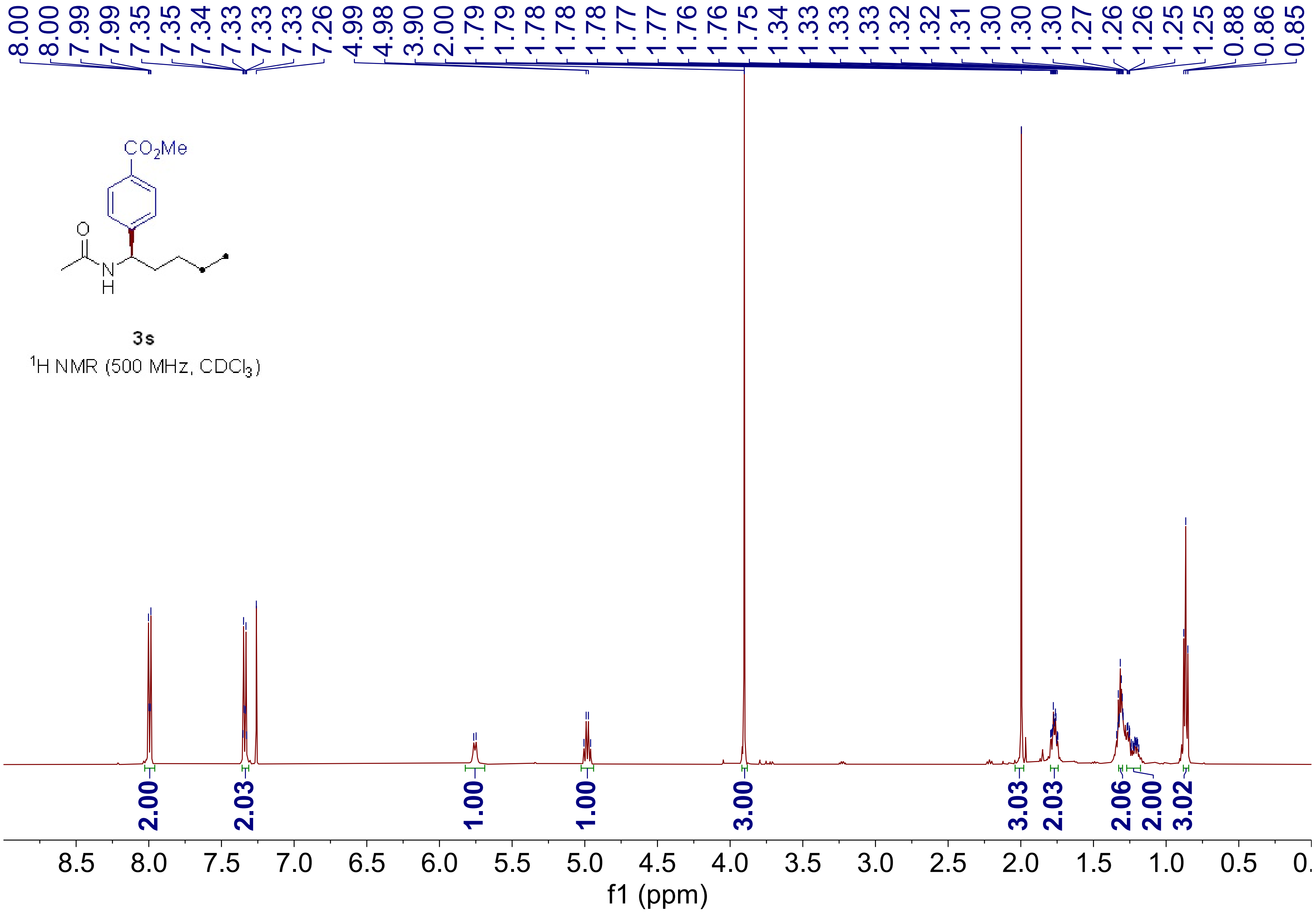
8.00 8.00 7.99 7.99 7.35 7.35 7.34 7.33 7.33 7.33 7.26 4.99 4.98 3.90 2.00 1.79 1.79 1.78 1.78 1.78 1.77 1.77 1.76 1.76 1.75 1.34 1.33 1.33 1.33 1.32 1.32 1.31 1.30 1.30 1.30 1.27 1.26 1.26 1.25 1.25 0.88 0.86 0.85
CO2Me
O
N
H
3s
1H NMR (500 MHz, CDCl3)
2.00 2.03 1.00 1.00 3.00 3.03 2.03 2.06 2.00 3.02
8.5 8.0 7.5 7.0 6.5 6.0 5.5 5.0 4.5 4.0 3.5 3.0 2.5 2.0 1.5 1.0 0.5
f1 (ppm)

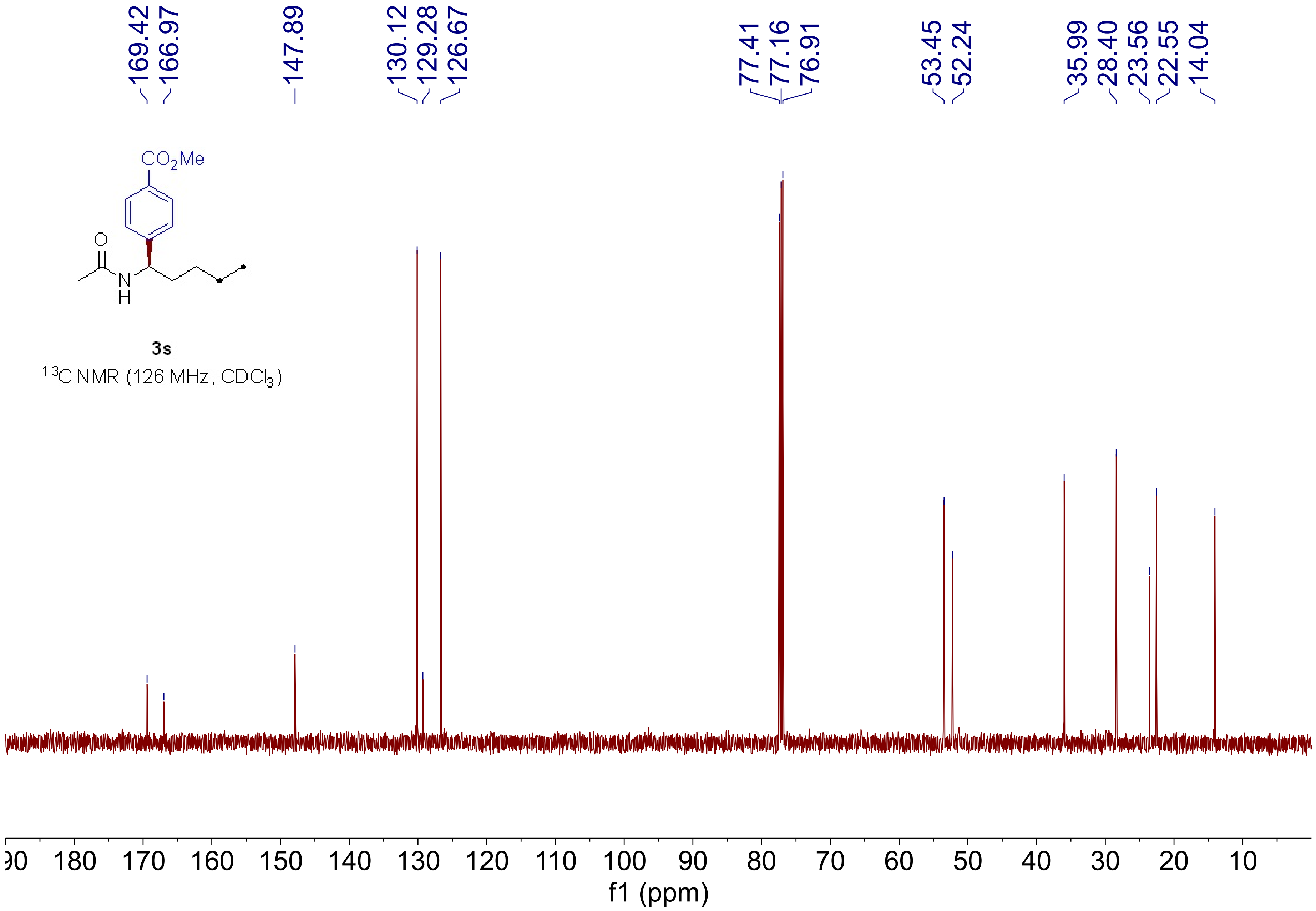
169.42
166.97
147.89
130.12
129.28
126.67
77.41
77.16
76.91
53.45
52.24
35.99
28.40
23.56
22.55
14.04
CO2Me
O
N
H
3s
13C NMR (126 MHz, CDCl3)
90 180 170 160 150 140 130 120 110 100 90 80 70 60 50 40 30 20 10
f1 (ppm)

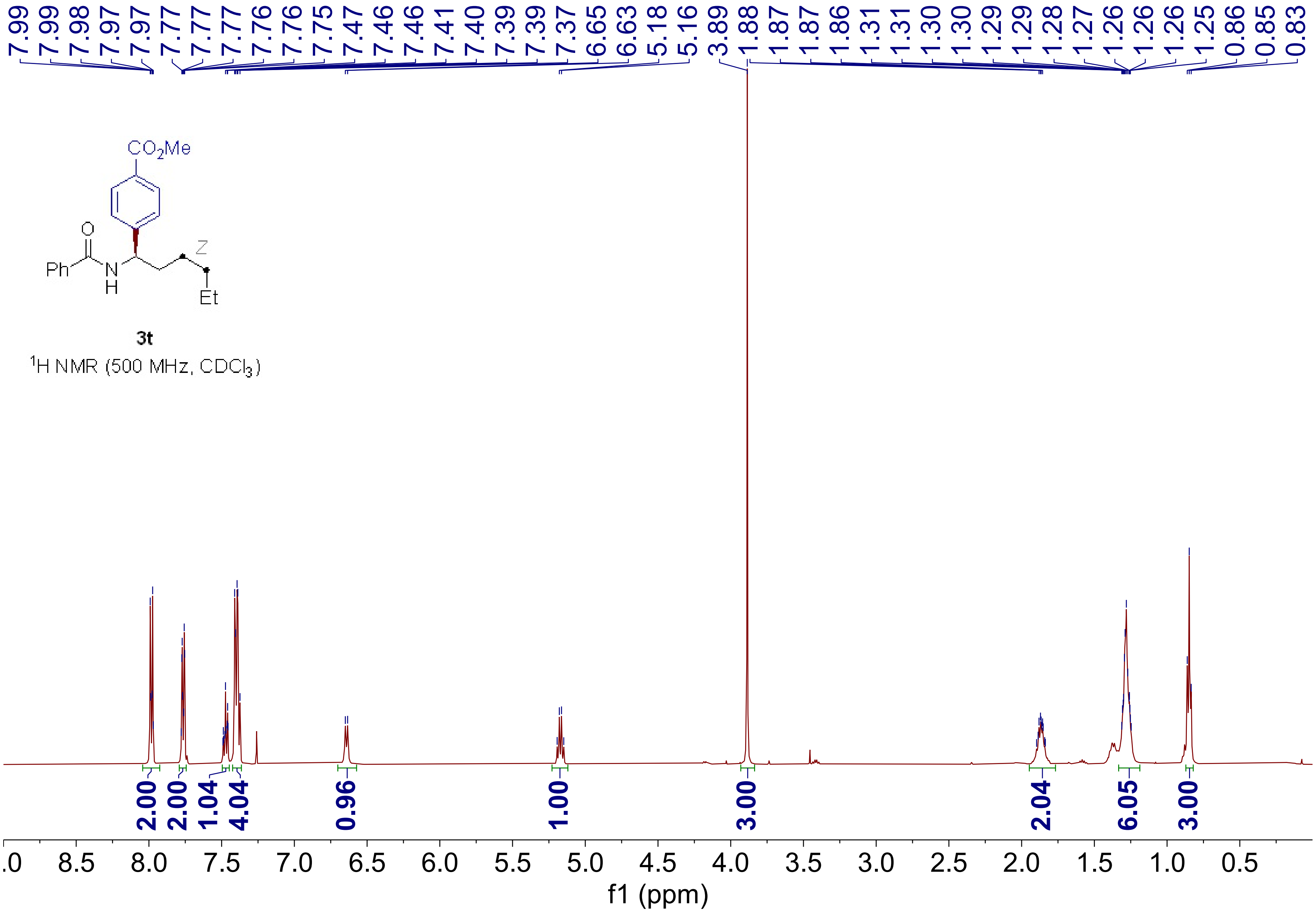

7.99 7.99 7.98 7.97 7.97 7.77 7.77 7.77 7.76 7.76 7.75 7.47 7.46 7.46 7.41 7.40 7.39 7.39 7.37 6.65 6.63 5.18 5.16 3.89 1.88 1.87 1.87 1.86 1.31 1.31 1.30 1.30 1.29 1.29 1.28 1.27 1.26 1.26 1.26 1.25 0.86 0.85 0.83
CO2Me
O
Ph
N
H
Z
Et
3t
1H NMR (500 MHz, CDCl3)
2.00 2.00 1.04 4.04 0.96 1.00 3.00 2.04 6.05 3.00
.0 8.5 8.0 7.5 7.0 6.5 6.0 5.5 5.0 4.5 4.0 3.5 3.0 2.5 2.0 1.5 1.0 0.5
f1 (ppm)

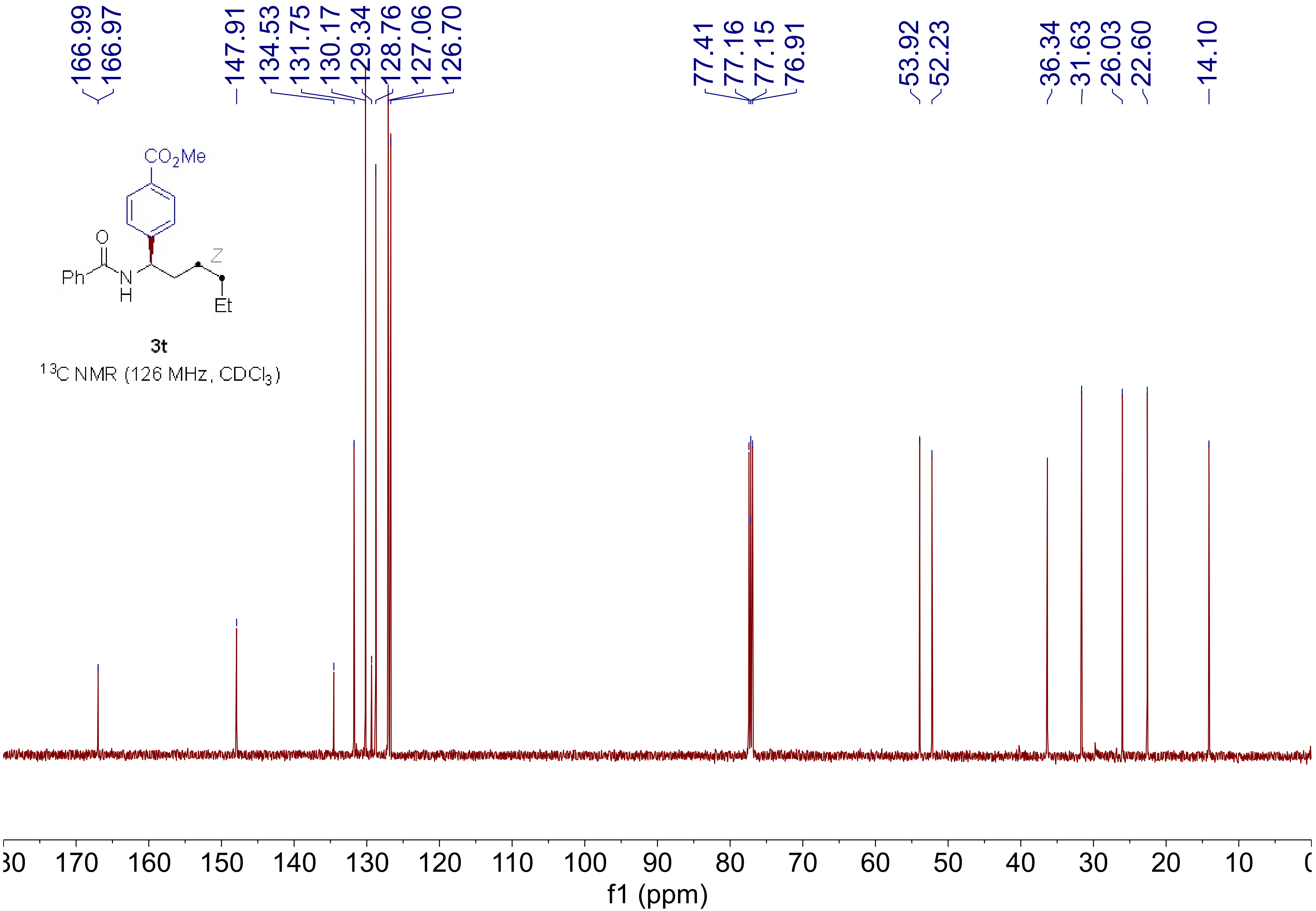

166.99
166.97
147.91
134.53
131.75
130.17
129.34
128.76
127.06
126.70
77.41
77.16
77.15
76.91
53.92
52.23
36.34
31.63
26.03
22.60
14.10
CO2Me
O
Ph
N
H
Z
Et
3t
13C NMR (126 MHz, CDCl3)
f1 (ppm)

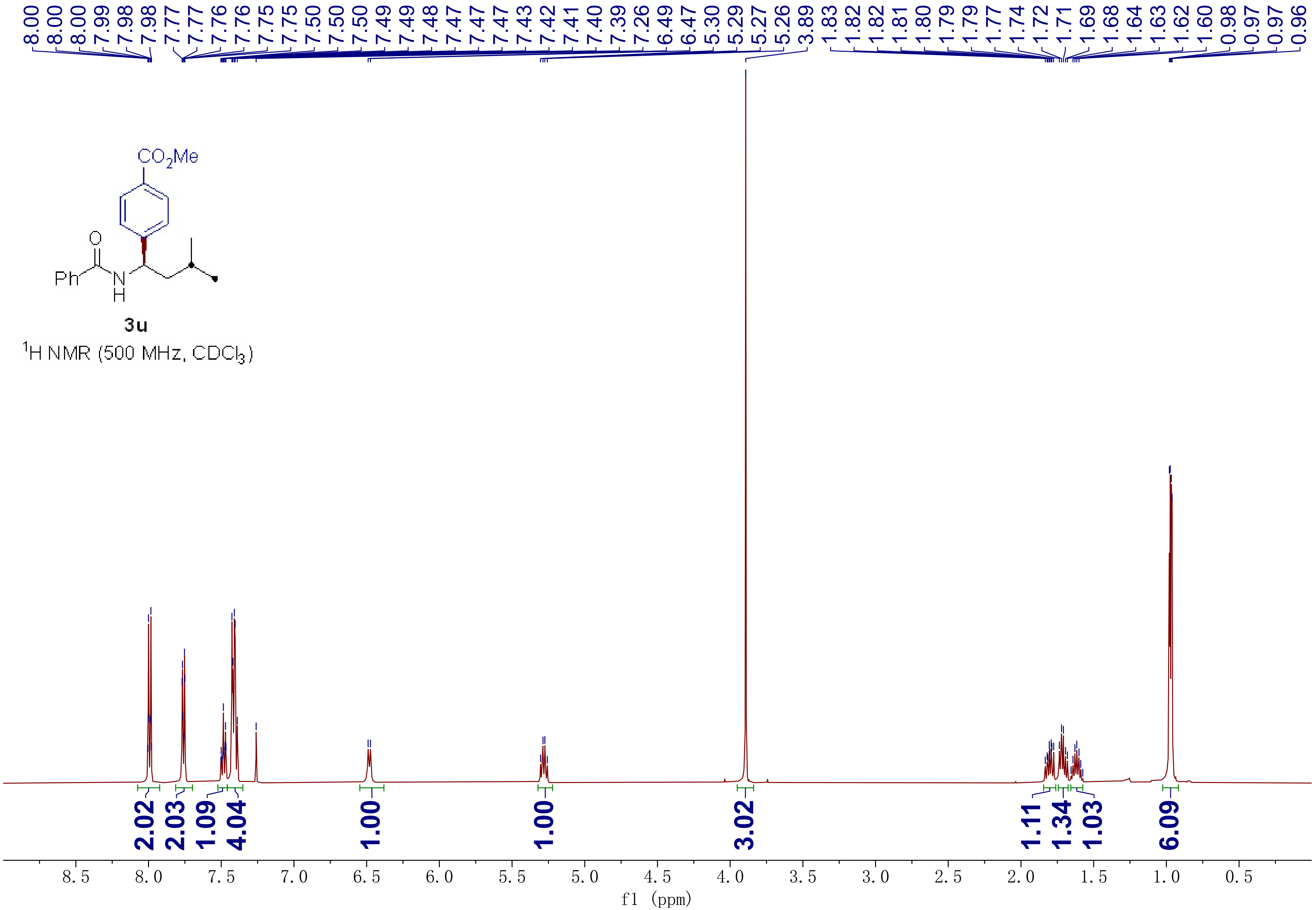
8.00 8.00 8.00 7.99 7.98 7.98 7.77 7.77 7.76 7.76 7.75 7.75 7.50 7.50 7.50 7.49 7.49 7.48 7.47 7.47 7.47 7.43 7.42 7.41 7.40 7.39 7.26 6.49 6.47 5.30 5.29 5.27 5.26 3.89 1.83 1.82 1.82 1.81 1.80 1.79 1.79 1.77 1.74 1.72 1.71 1.69 1.68 1.64 1.63 1.62 1.60 0.98 0.97 0.97 0.96
CO2Me
O
Ph
N
H
3u
1H NMR (500 MHz, CDCl3)
2.02 2.03 1.09 4.04 1.00 1.00 3.02 1.11 1.34 1.03 6.09
8.5 8.0 7.5 7.0 6.5 6.0 5.5 5.0 4.5 4.0 3.5 3.0 2.5 2.0 1.5 1.0 0.5
f1 (ppm)

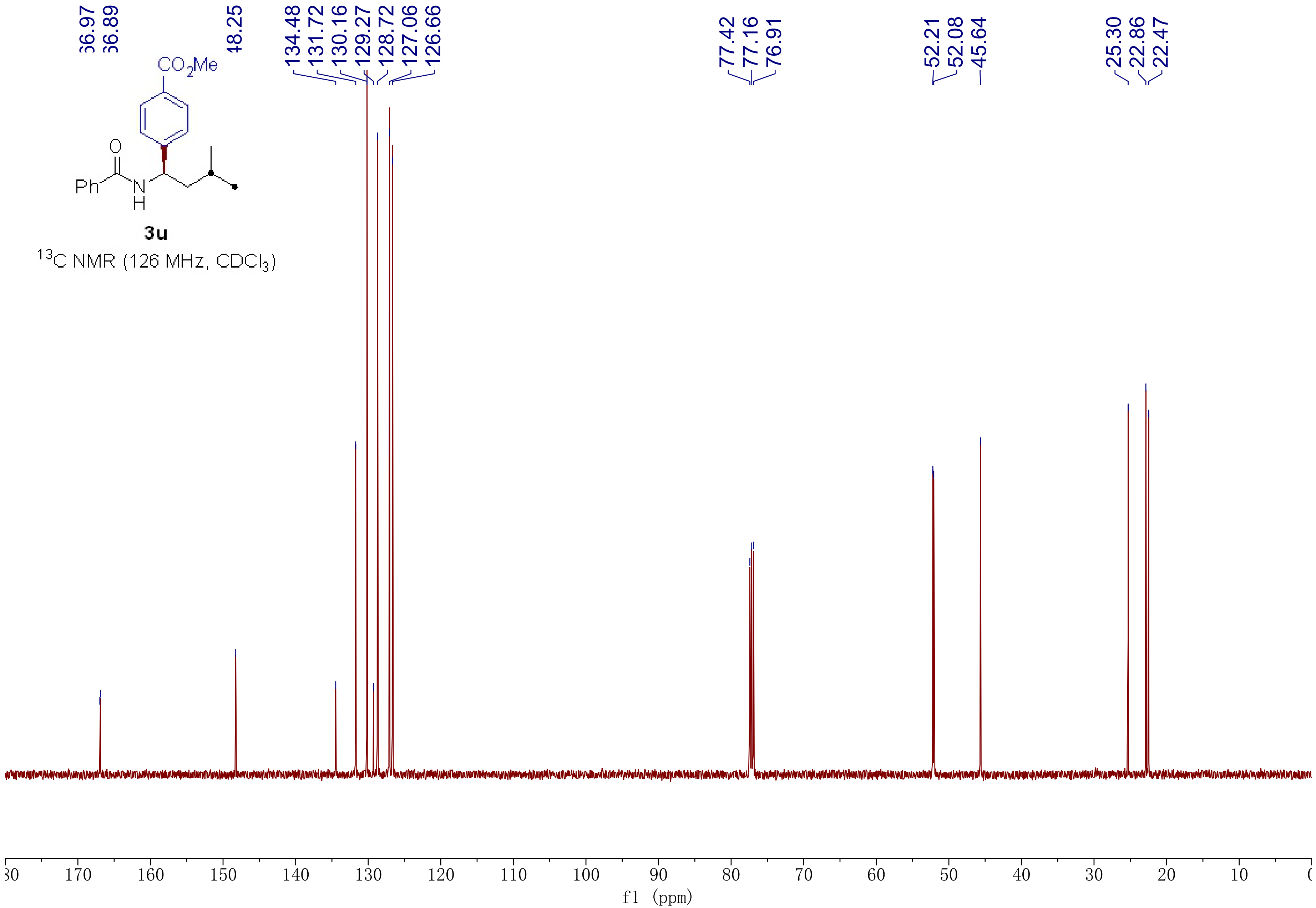

36.97
36.89
48.25
134.48
131.72
130.16
129.27
128.72
127.06
126.66
77.42
77.16
76.91
52.21
52.08
45.64
25.30
22.86
22.47
CO2Me
O
Ph
N
H
3u
13C NMR (126 MHz, CDCl3)
170
160
150
140
130
120
110
100
90
80
70
60
50
40
30
20
10
f1 (ppm)

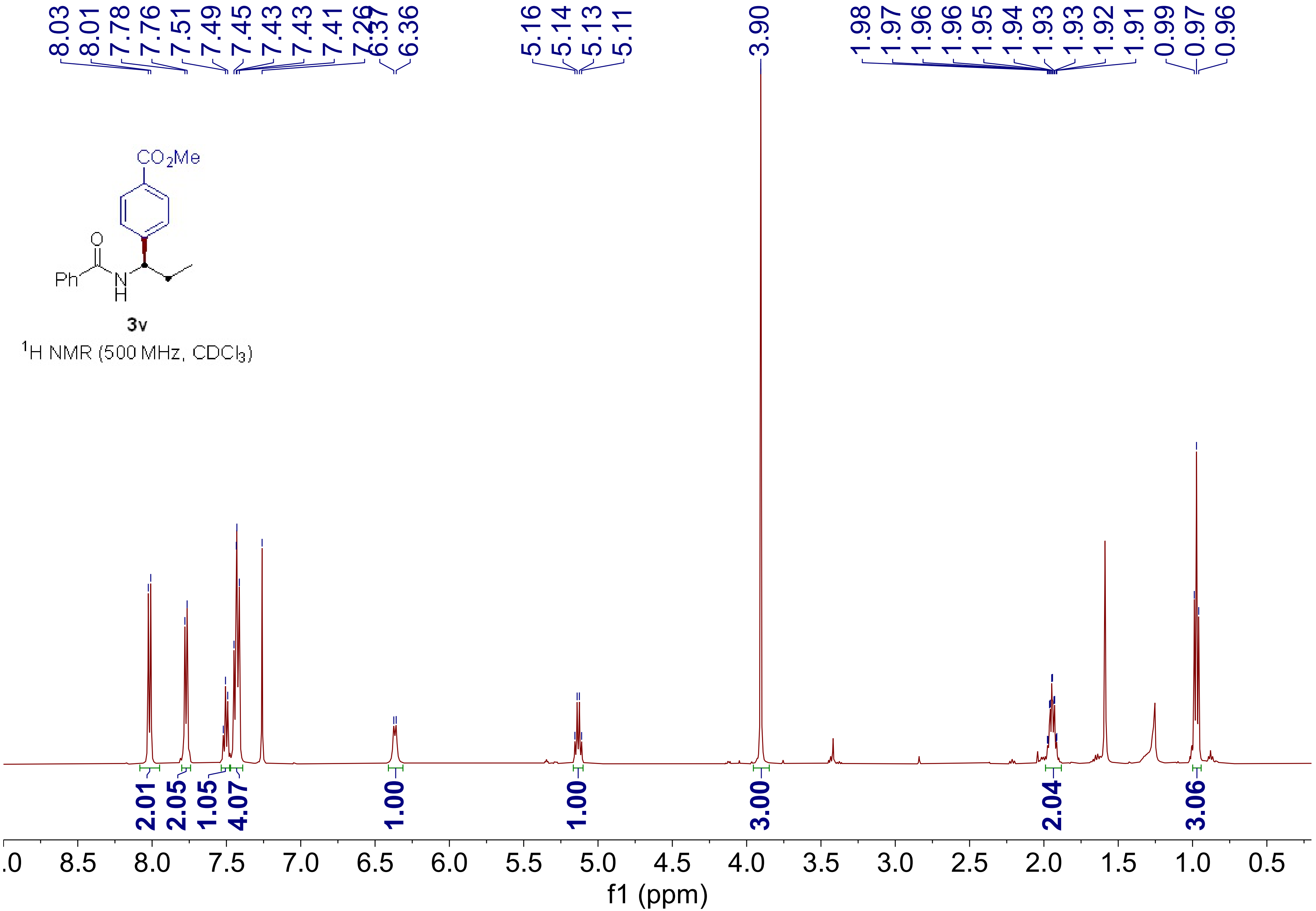
8.03
8.01
7.78
7.76
7.51
7.49
7.45
7.43
7.43
7.41
7.26
6.37
6.36
5.16
5.14
5.13
5.11
3.90
1.98
1.97
1.96
1.96
1.95
1.94
1.93
1.93
1.92
1.91
0.99
0.97
0.96
CO2Me
O
Ph
N
H
3v
1H NMR (500 MHz, CDCl3)
2.01
2.05
1.05
4.07
1.00
1.00
3.00
2.04
3.06
.0
8.5
8.0
7.5
7.0
6.5
6.0
5.5
5.0
4.5
4.0
3.5
3.0
2.5
2.0
1.5
1.0
0.5
f1 (ppm)

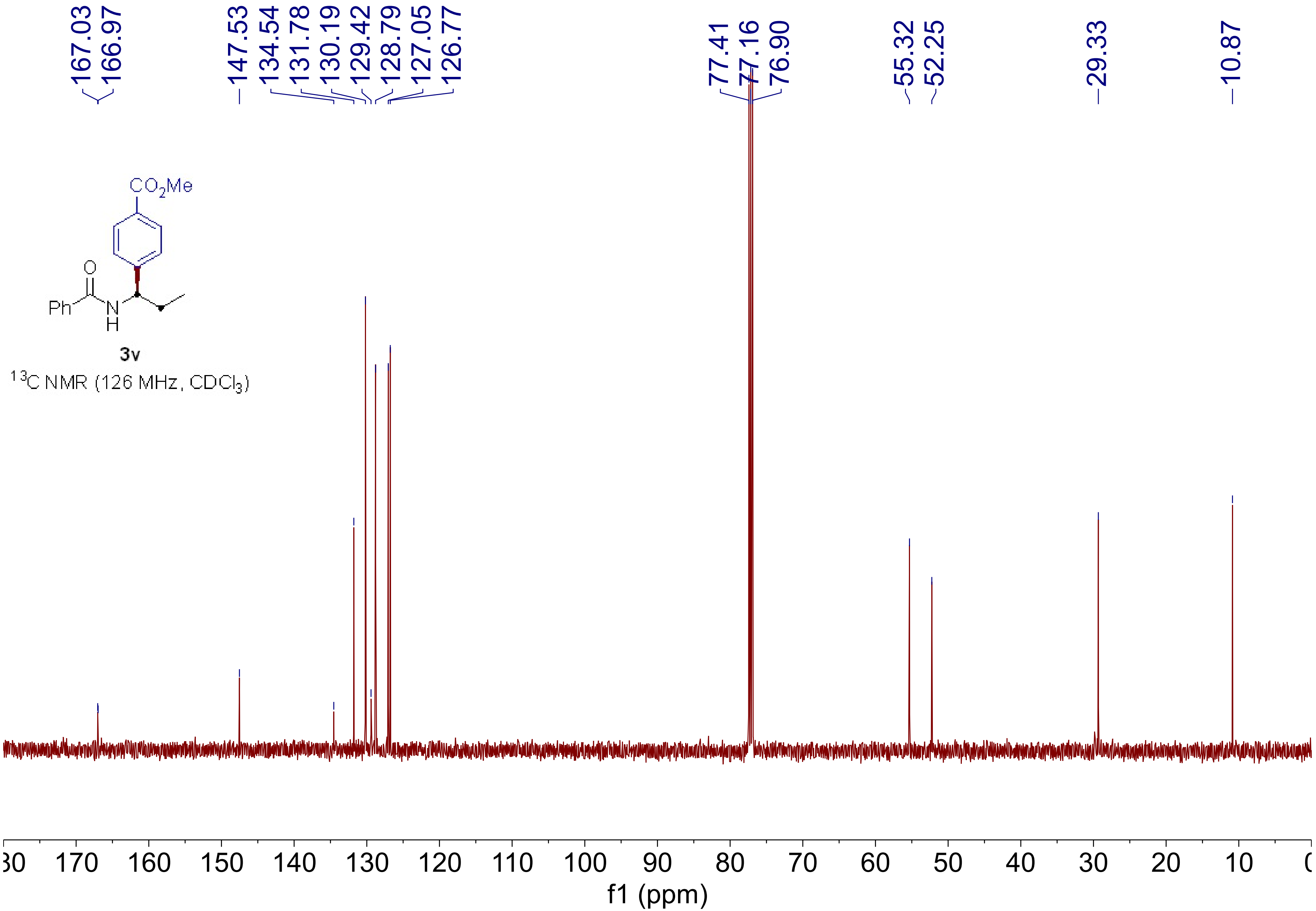
167.03
166.97
147.53
134.54
131.78
130.19
129.42
128.79
127.05
126.77
77.41
77.16
76.90
55.32
52.25
29.33
10.87
CO2Me
O
Ph
N
H
3v
13C NMR (126 MHz, CDCl3)
170
160
150
140
130
120
110
100
90
80
70
60
50
40
30
20
10
f1 (ppm)

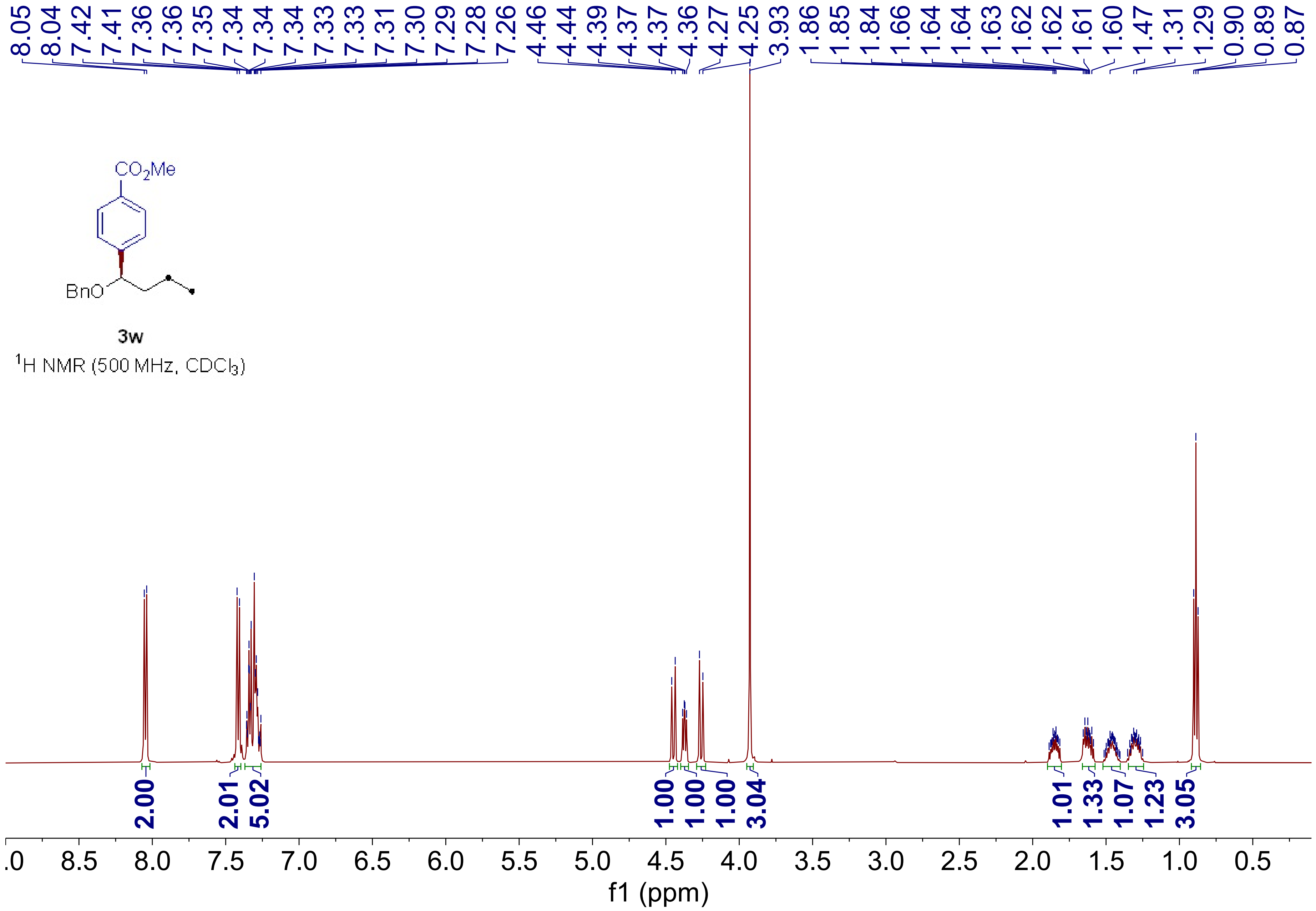
8.05 8.04 7.42 7.41 7.36 7.36 7.35 7.34 7.34 7.34 7.33 7.33 7.31 7.30 7.29 7.28 7.26 4.46 4.44 4.39 4.37 4.37 4.36 4.27 4.25 3.93 1.86 1.85 1.84 1.66 1.64 1.64 1.63 1.62 1.62 1.61 1.60 1.47 1.31 1.29 0.90 0.89 0.87
CO2Me
BnO
3w
1H NMR (500 MHz, CDCl3)
2.00 2.01 5.02 1.00 1.00 1.00 3.04 1.01 1.33 1.07 1.23 3.05
.0 8.5 8.0 7.5 7.0 6.5 6.0 5.5 5.0 4.5 4.0 3.5 3.0 2.5 2.0 1.5 1.0 0.5
f1 (ppm)

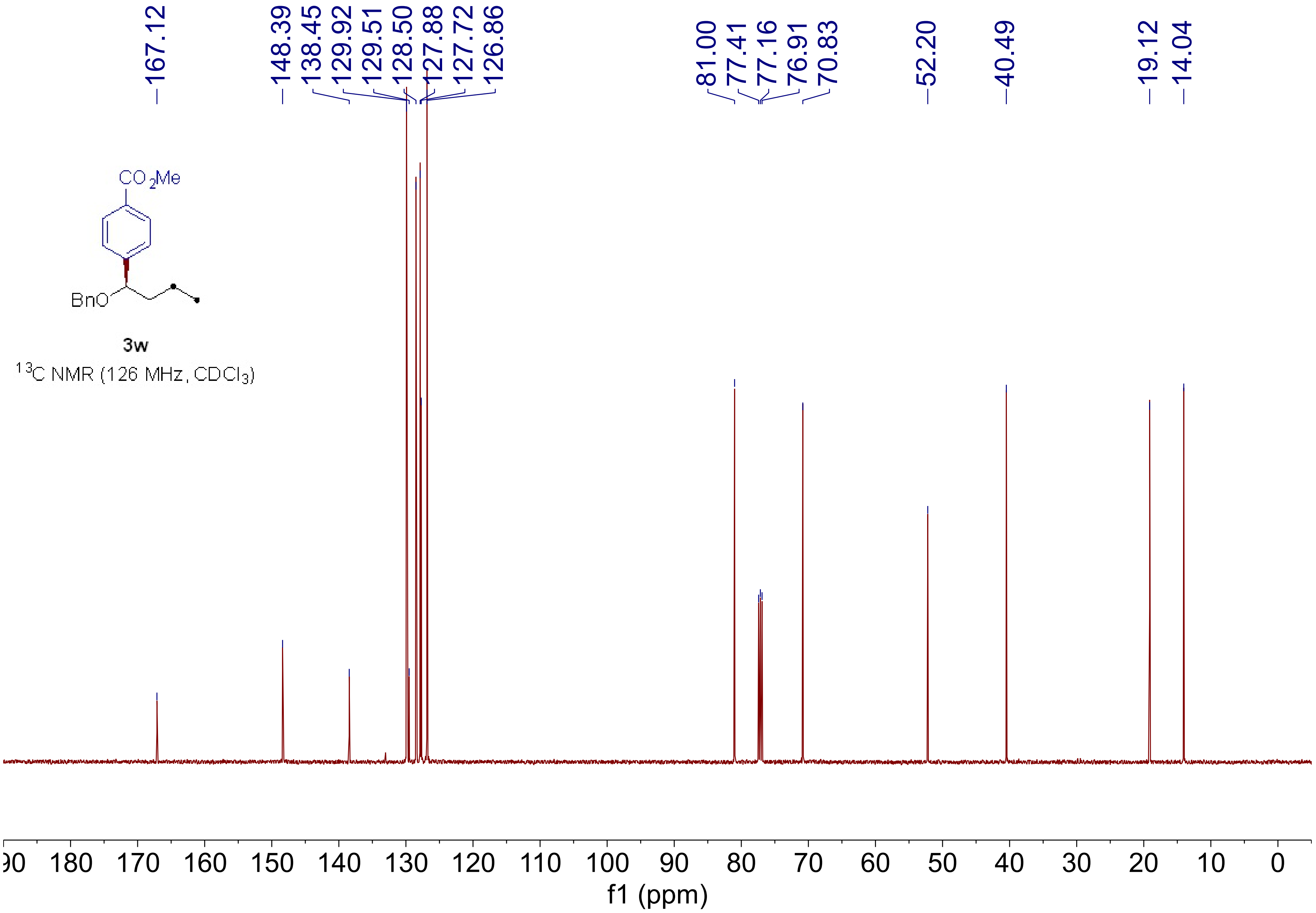
167.12
148.39
138.45
129.92
129.51
128.50
127.88
127.72
126.86
81.00
77.41
77.16
76.91
70.83
52.20
40.49
19.12
14.04
CO2Me
BnO
3w
13C NMR (126 MHz, CDCl3)
190 180 170 160 150 140 130 120 110 100 90 80 70 60 50 40 30 20 10 0
f1 (ppm)

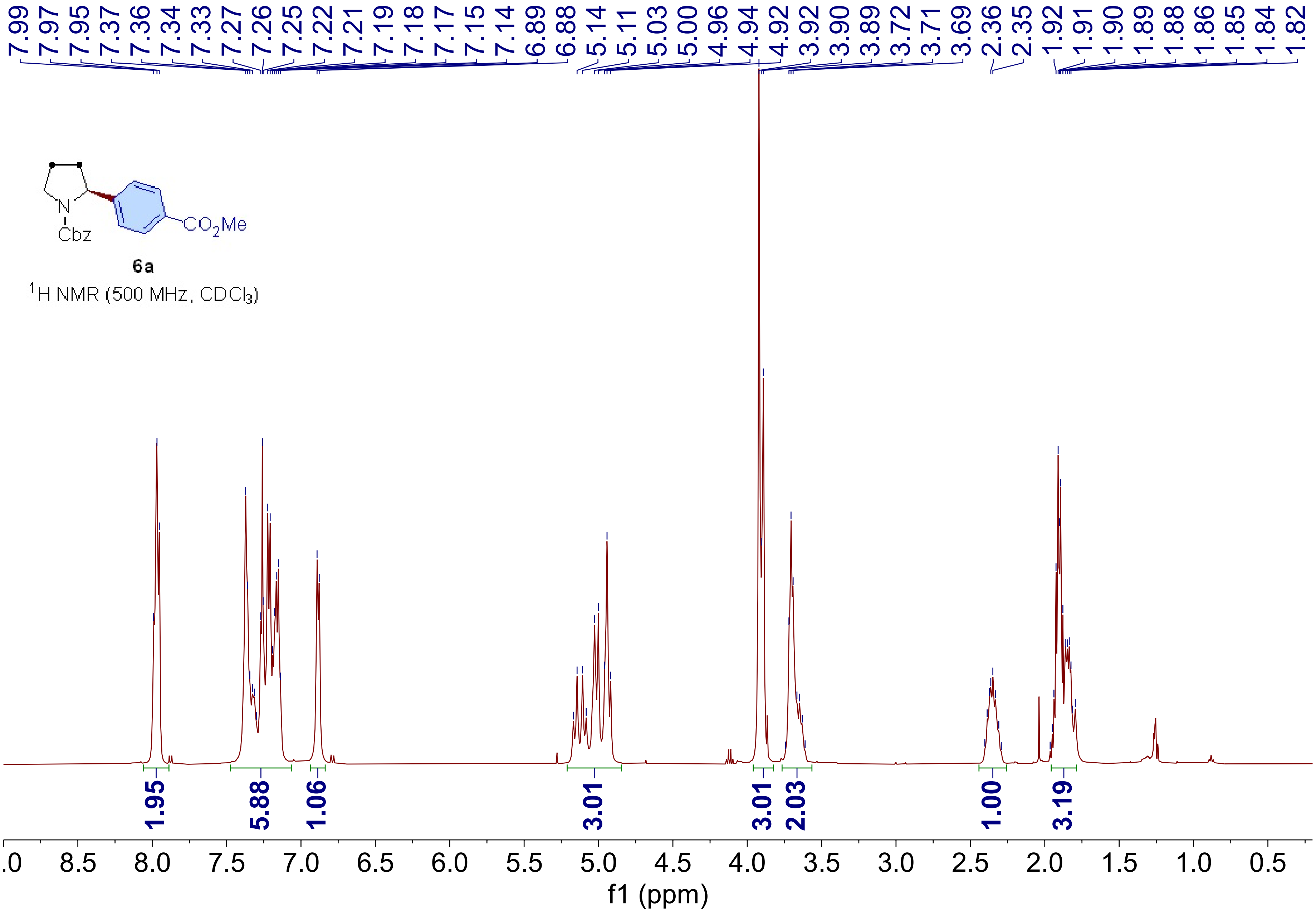

6a

^{1}H NMR (500 MHz, $CDCl_3$)

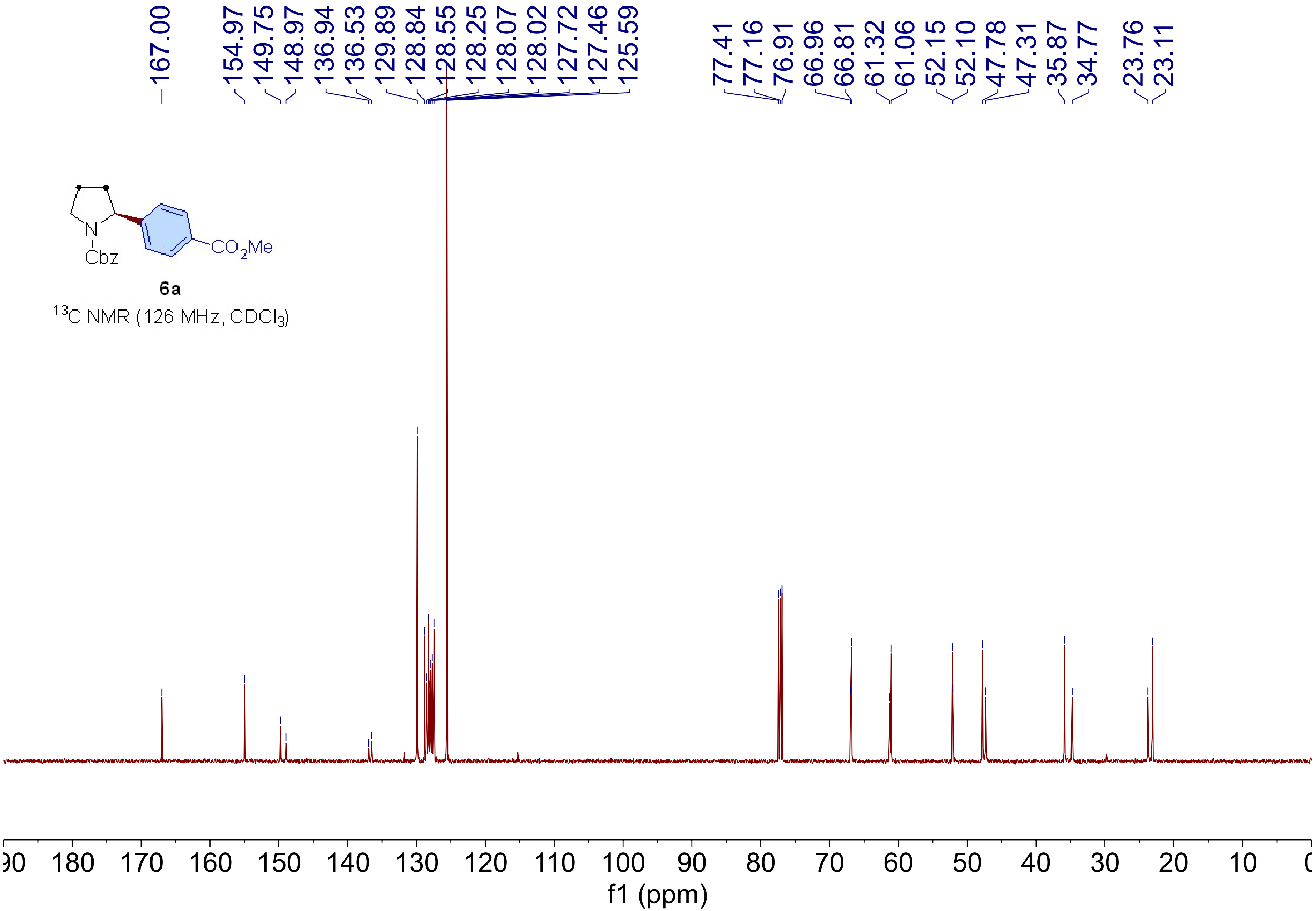
167.00
154.97
149.75
148.97
136.94
136.53
129.89
128.84
128.55
128.25
128.07
128.02
127.72
127.46
125.59
77.41
77.16
76.91
66.96
66.81
61.32
61.06
52.15
52.10
47.78
47.31
35.87
34.77
23.76
23.11
N
Cbz
CO2Me
6a
13C NMR (126 MHz, CDCl3)
f1 (ppm)

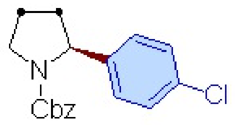

6b

^{1}H NMR (500 MHz, $CDCl_3$)

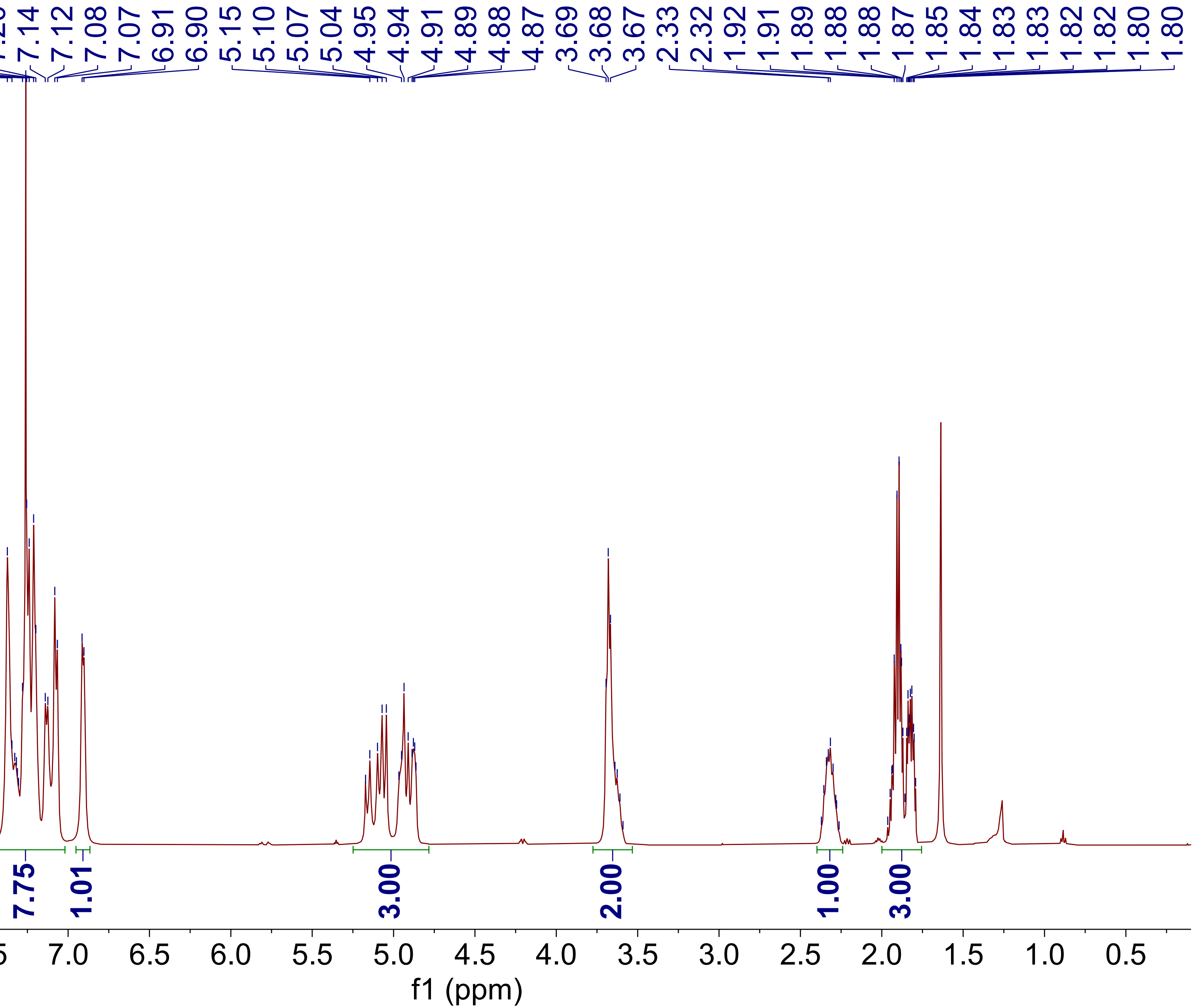

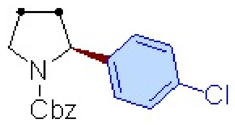

6b

^{13}C NMR (126 MHz, $CDCl_3$)

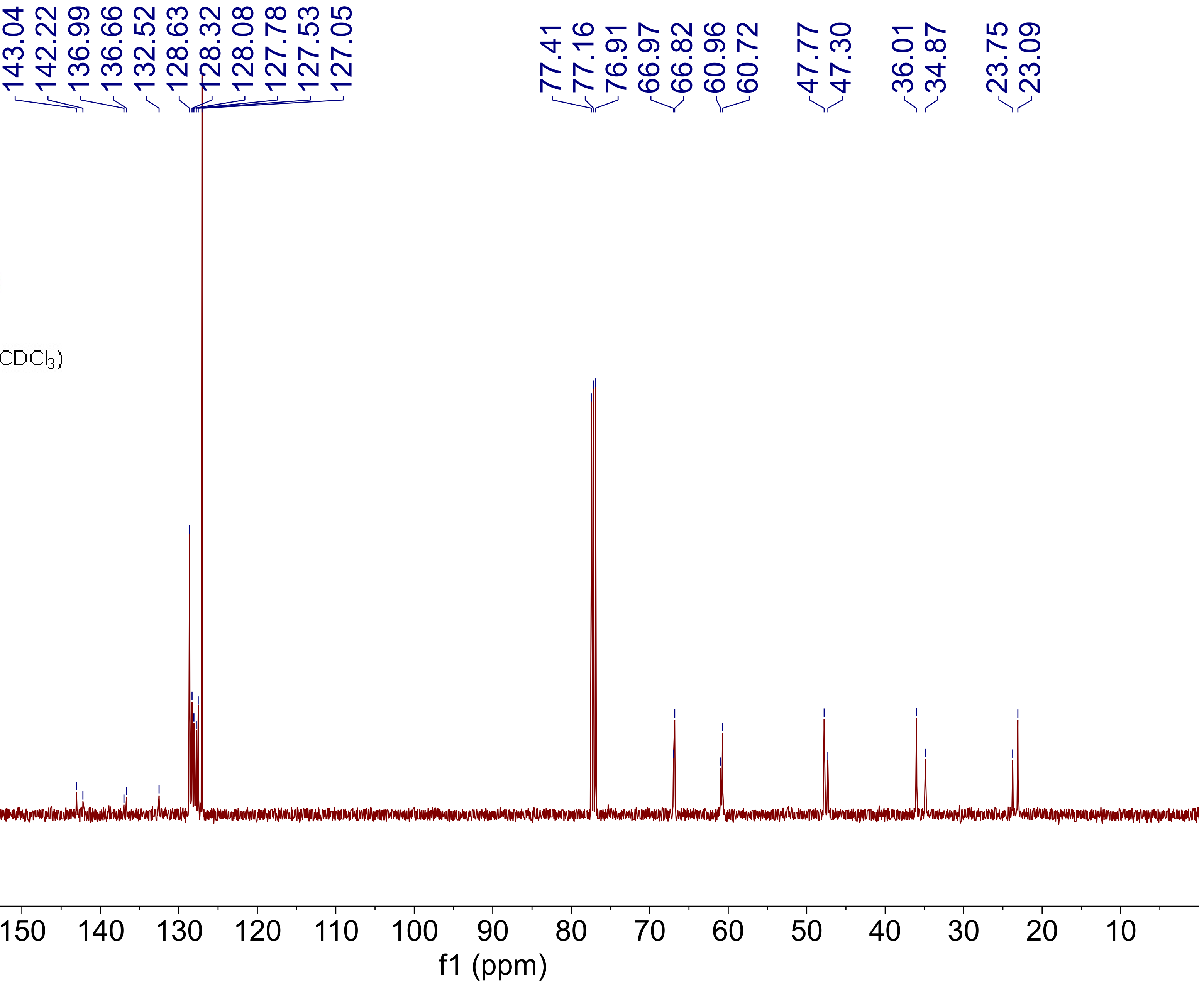

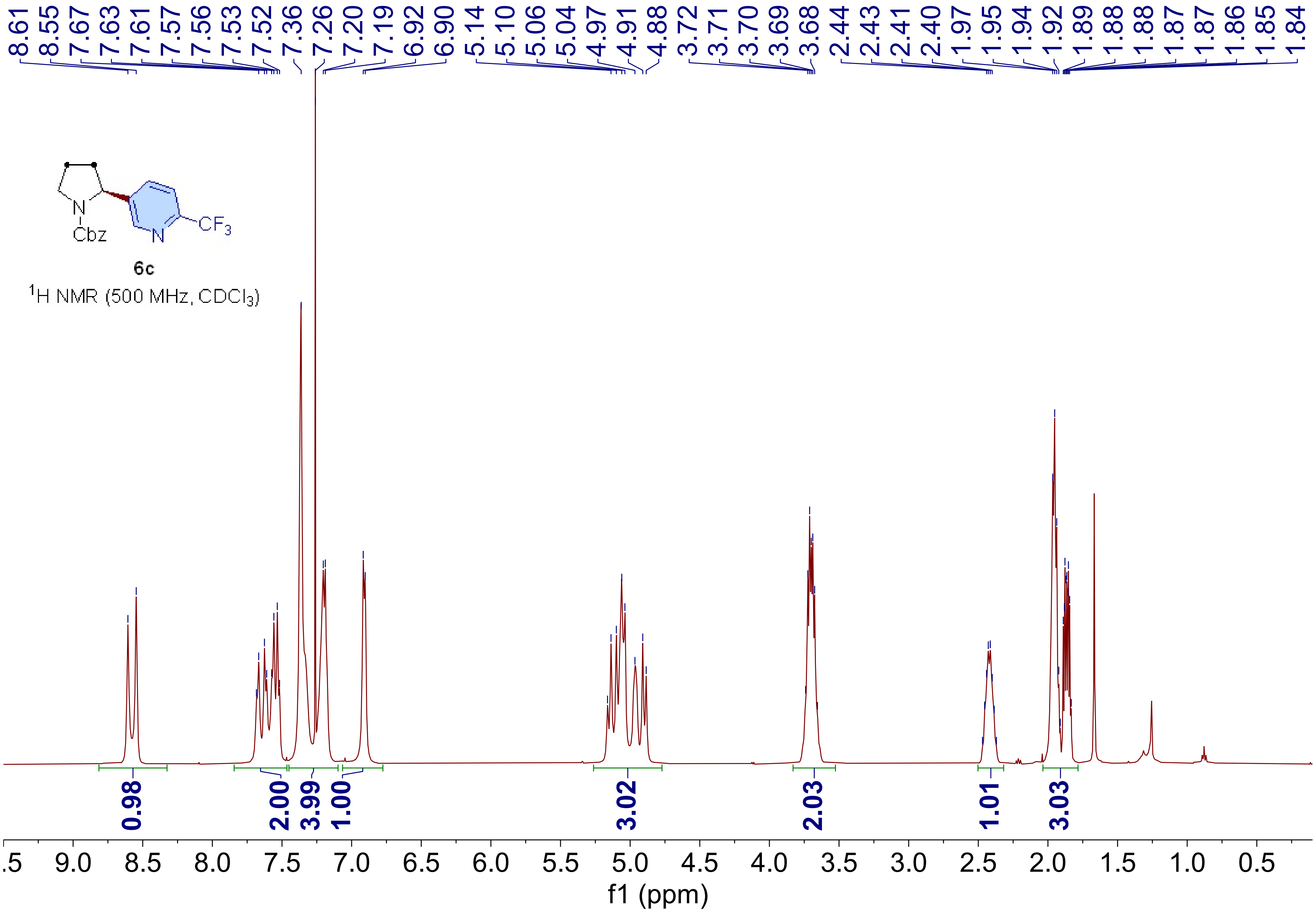
8.61 8.55 7.67 7.63 7.61 7.57 7.56 7.53 7.52 7.36 7.26 7.20 7.19 6.92 6.90 5.14 5.10 5.06 5.04 4.97 4.91 4.88 3.72 3.71 3.70 3.69 3.68 2.44 2.43 2.41 2.40 1.97 1.95 1.94 1.92 1.89 1.88 1.88 1.87 1.87 1.86 1.85 1.84
N
Cbz
N
CF3
6c
1H NMR (500 MHz, CDCl3)
0.98
2.00
3.99
1.00
3.02
2.03
1.01
3.03
.5 9.0 8.5 8.0 7.5 7.0 6.5 6.0 5.5 5.0 4.5 4.0 3.5 3.0 2.5 2.0 1.5 1.0 0.5
f1 (ppm)

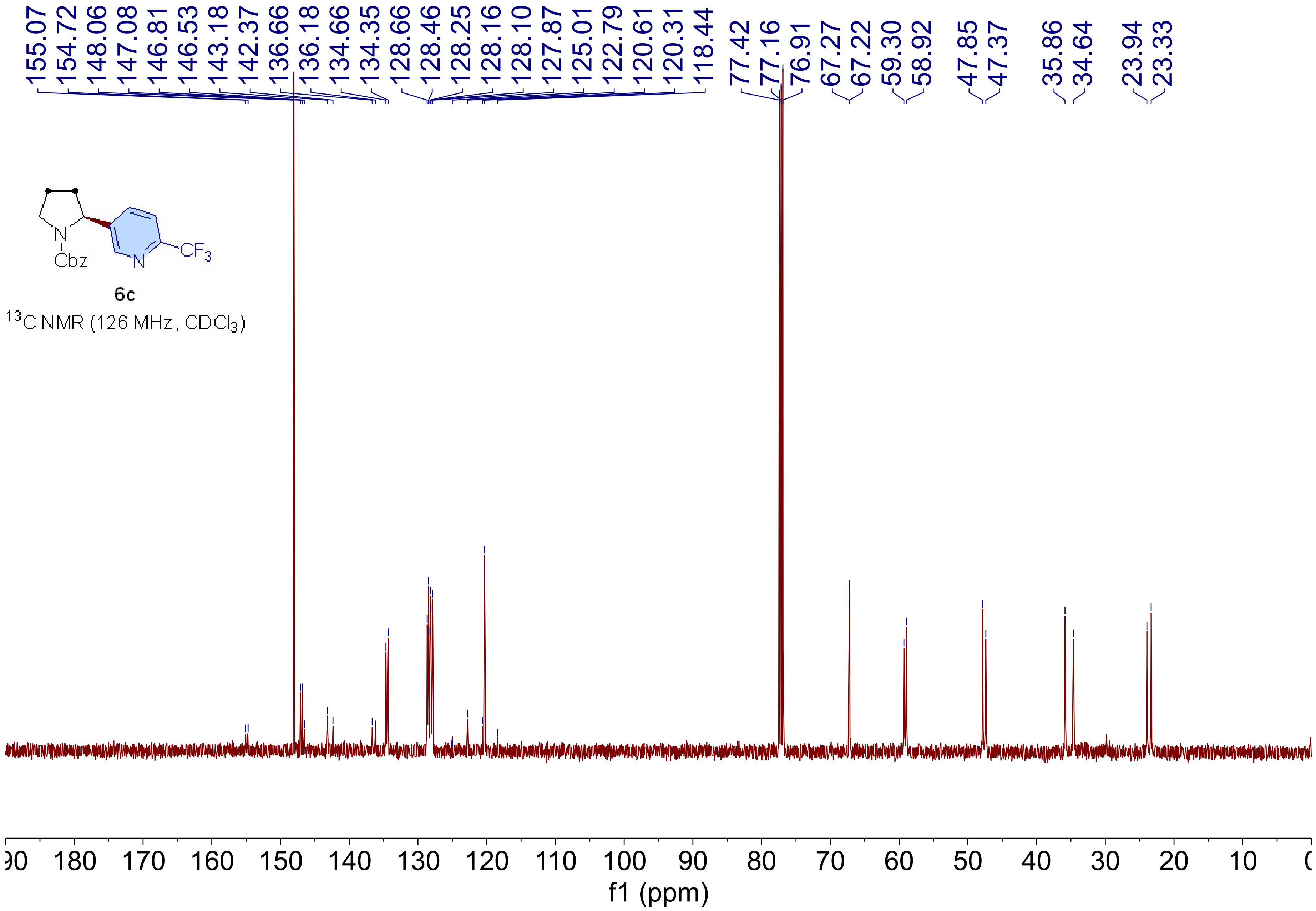
155.07
154.72
148.06
147.08
146.81
146.53
143.18
142.37
136.66
136.18
134.66
134.35
128.66
128.46
128.25
128.16
128.10
127.87
125.01
122.79
120.61
120.31
118.44
77.42
77.16
76.91
67.27
67.22
59.30
58.92
47.85
47.37
35.86
34.64
23.94
23.33
N
Cbz
N
CF3
6c
13C NMR (126 MHz, CDCl3)
f1 (ppm)

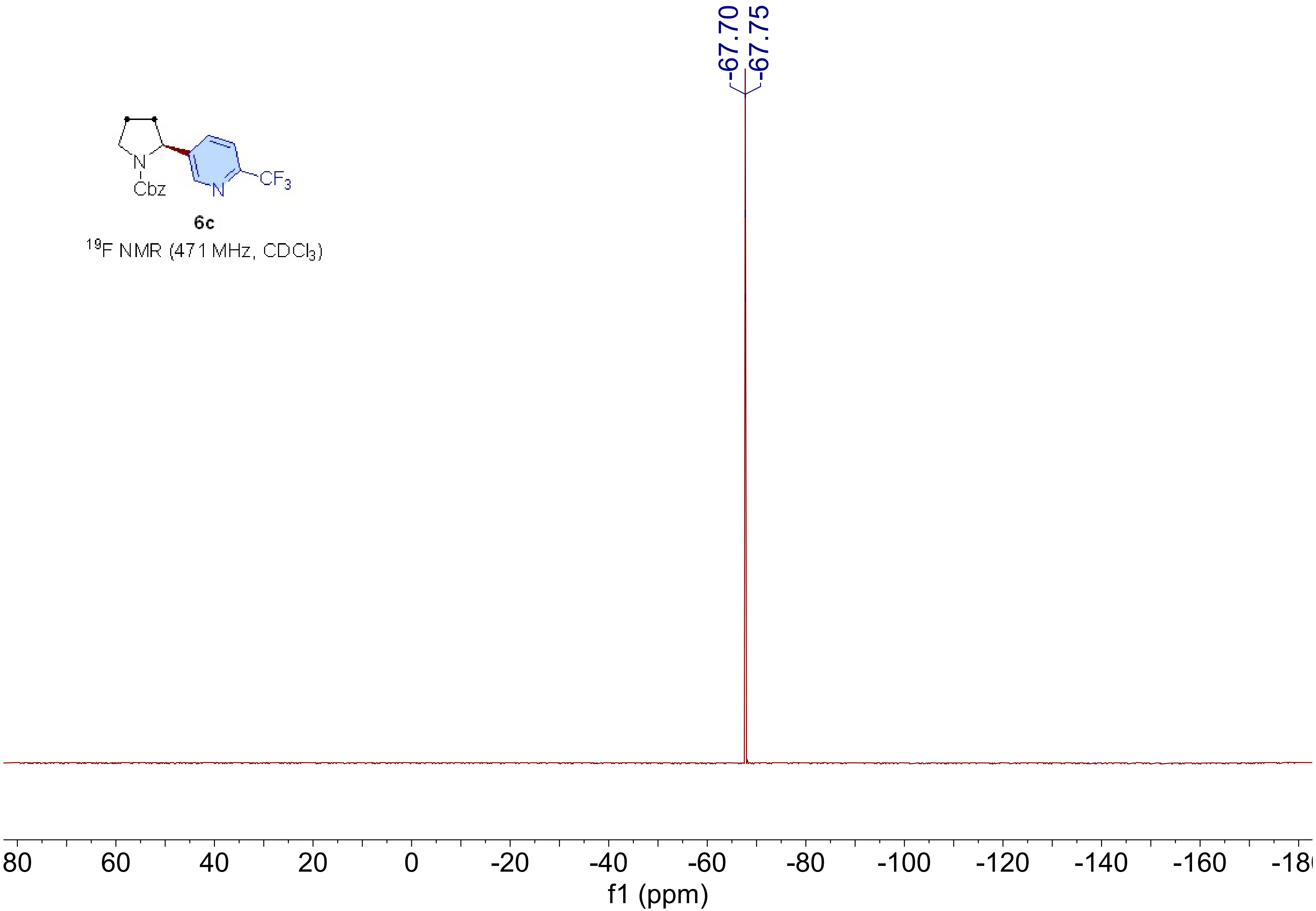

6c

^{19}F NMR (471 MHz, $CDCl_3$)

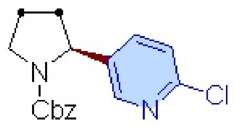

6d

H NMR (500 MHz, $CDCl_3$)

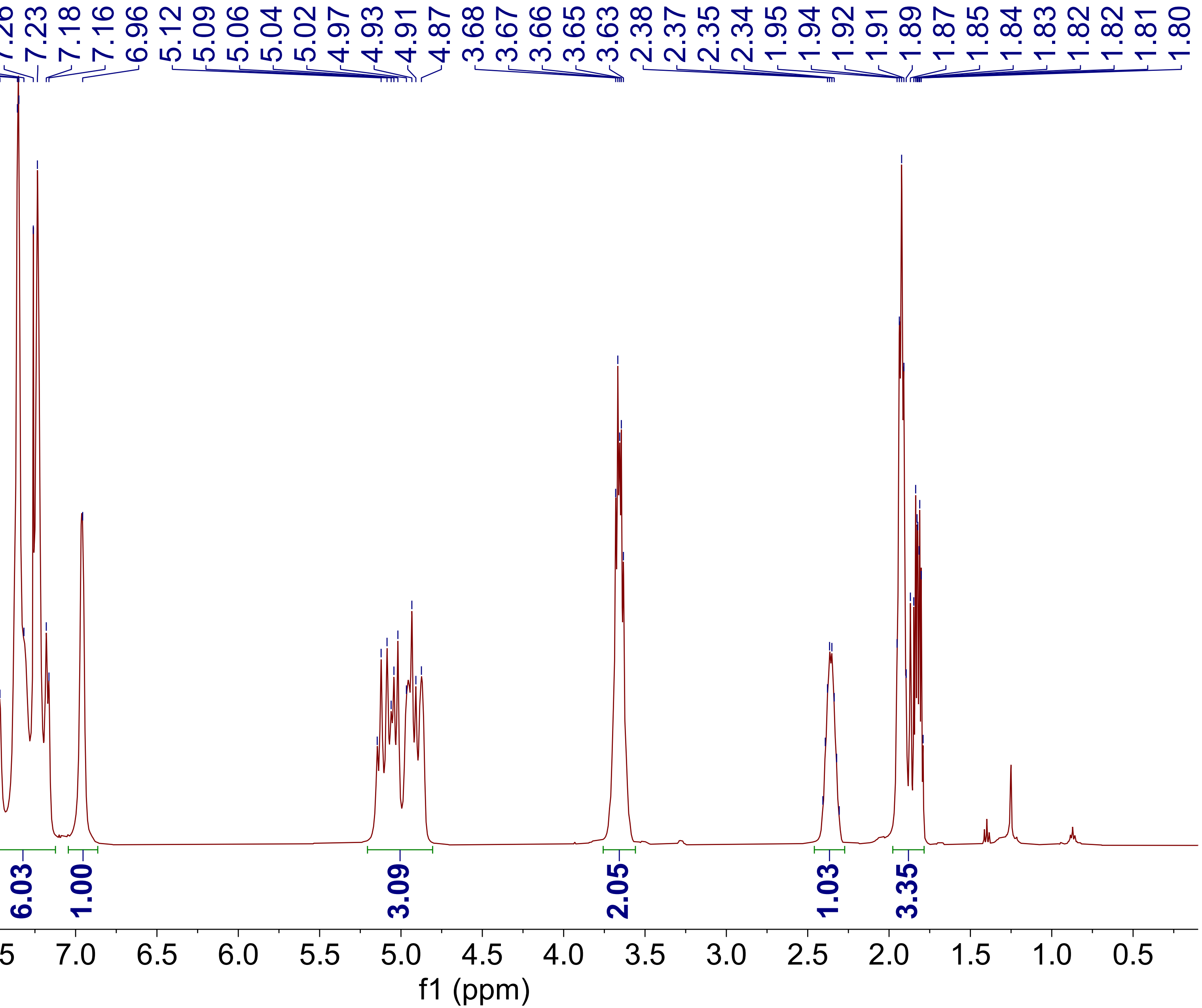

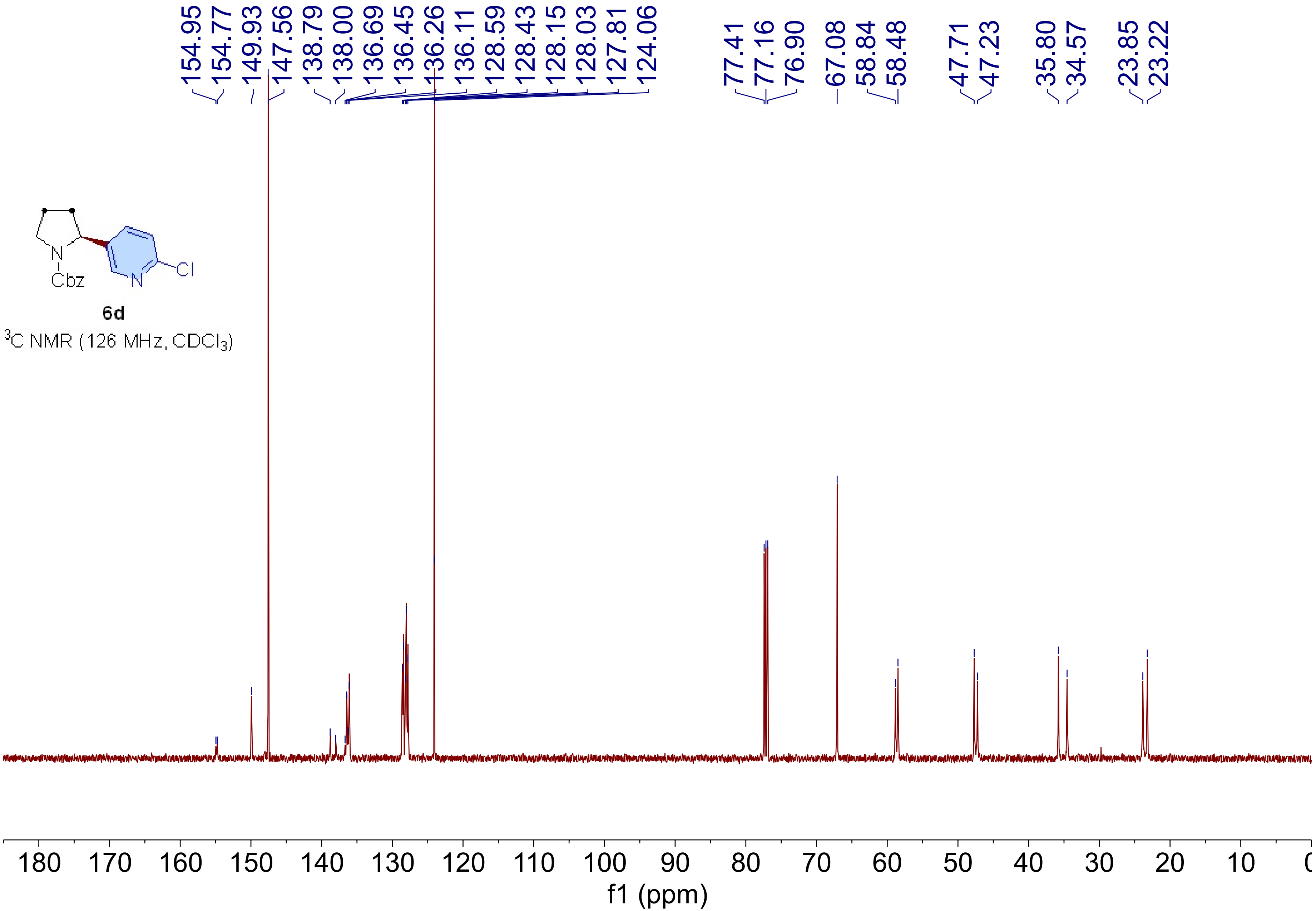
Cbz
N
Cl
6d
^{13}C NMR (126 MHz, $CDCl_3$)
154.95
154.77
149.93
147.56
138.79
138.00
136.69
136.45
136.26
136.11
128.59
128.43
128.15
128.03
127.81
124.06
77.41
77.16
76.90
67.08
58.84
58.48
47.71
47.23
35.80
34.57
23.85
23.22
180 170 160 150 140 130 120 110 100 90 80 70 60 50 40 30 20 10
f1 (ppm)

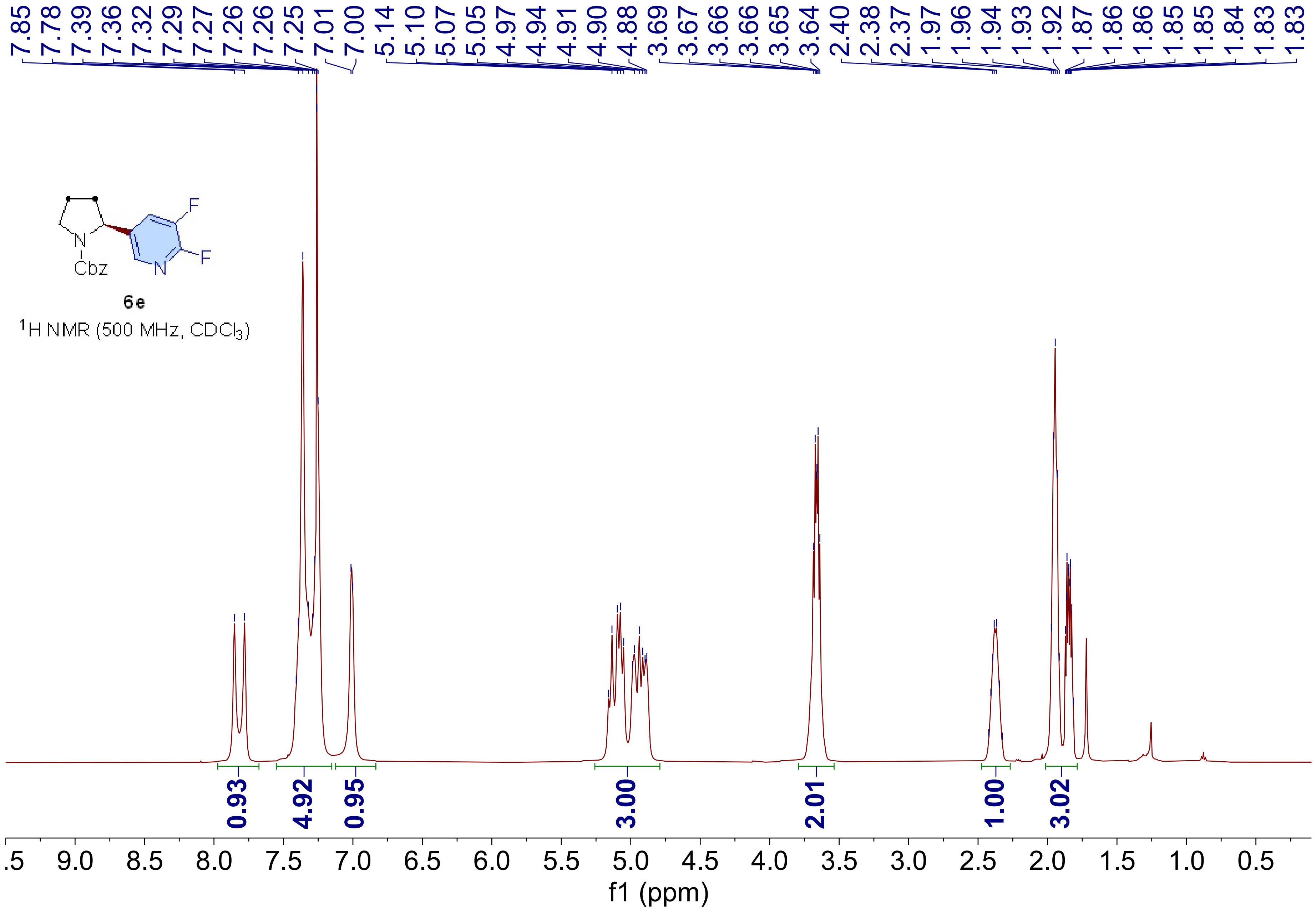

7.85 7.78 7.39 7.36 7.32 7.29 7.27 7.26 7.26 7.25 7.01 7.00 5.14 5.10 5.07 5.05 4.97 4.94 4.91 4.90 4.88 3.69 3.67 3.66 3.66 3.65 3.64 2.40 2.38 2.37 1.97 1.96 1.94 1.93 1.92 1.87 1.86 1.86 1.85 1.85 1.84 1.83 1.83
F
F
N
N
Cbz
6e
^{1}H NMR (500 MHz, $CDCl_3$)
0.93
4.92
0.95
3.00
2.01
1.00
3.02
f1 (ppm)

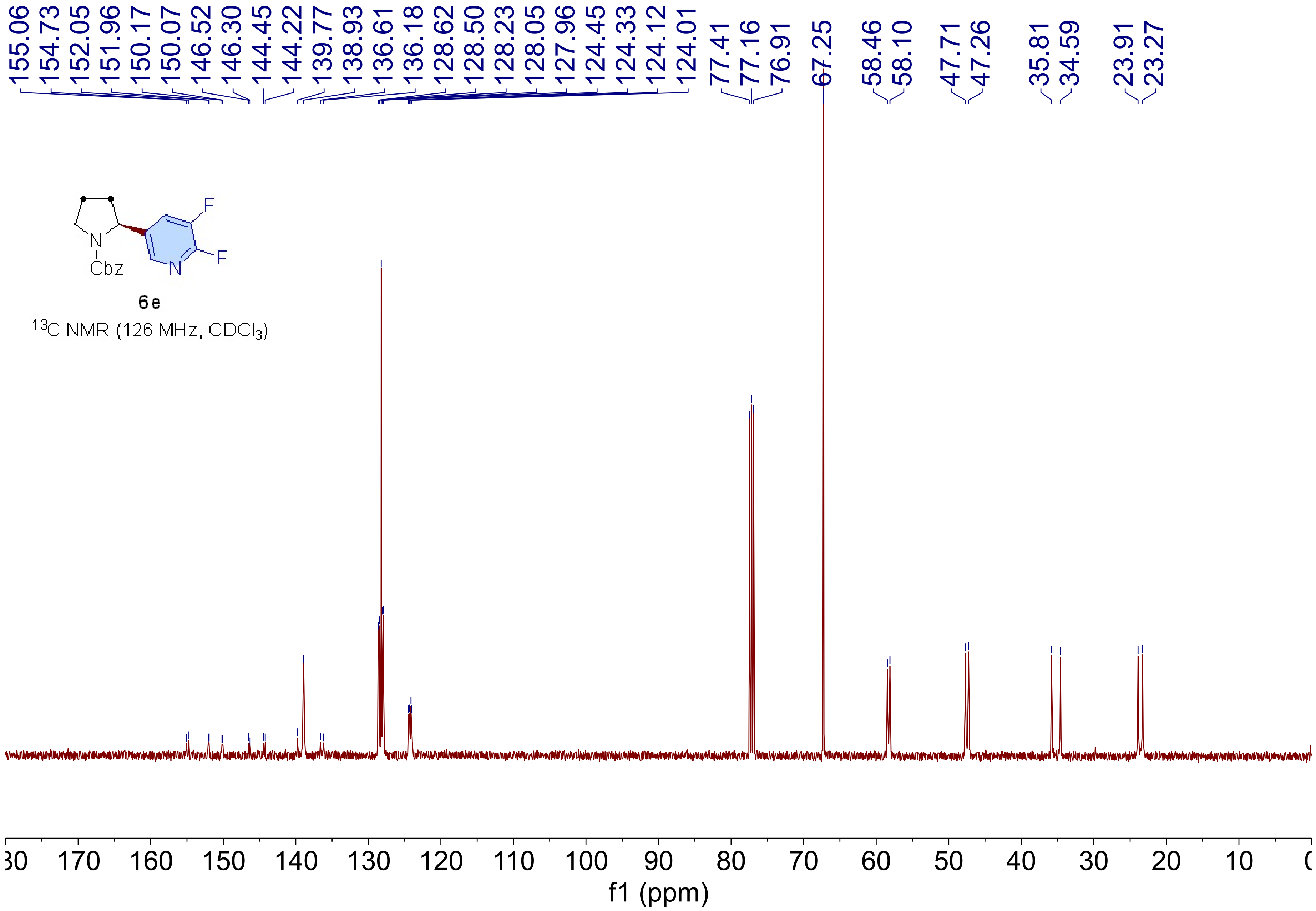
155.06
154.73
152.05
151.96
150.17
150.07
146.52
146.30
144.45
144.22
139.77
138.93
136.61
136.18
128.62
128.50
128.23
128.05
127.96
124.45
124.33
124.12
124.01
77.41
77.16
76.91
67.25
58.46
58.10
47.71
47.26
35.81
34.59
23.91
23.27
F
F
N
N
Cbz
6e
^{13}C NMR (126 MHz, CDCl$_3$)
f1 (ppm)

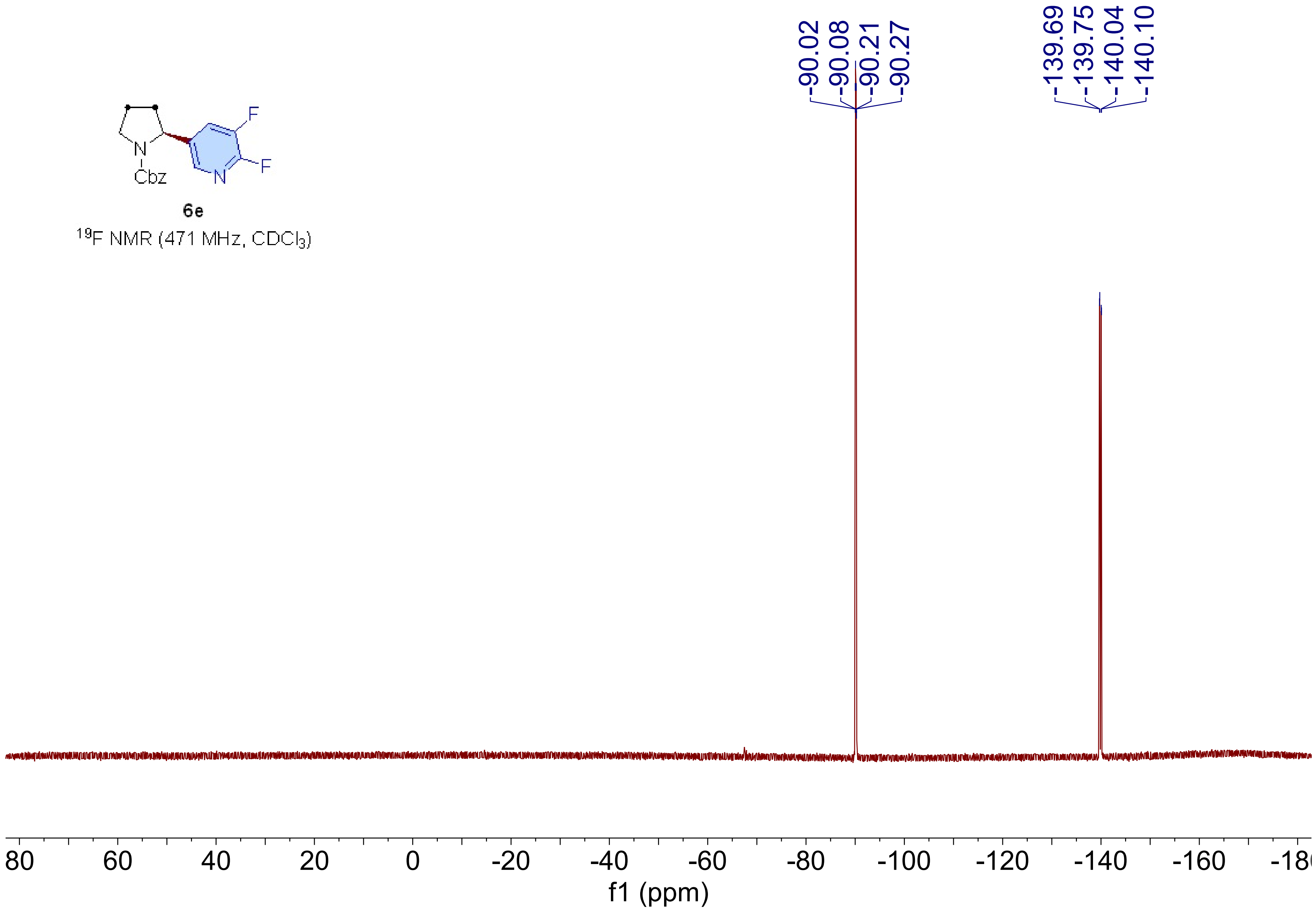
Cbz
6e
^{19}F NMR (471 MHz, CDCl$_3$)
-90.02
-90.08
-90.21
-90.27
-139.69
-139.75
-140.04
-140.10
80
60
40
20
0
-20
-40
-60
-80
-100
-120
-140
-160
f1 (ppm)

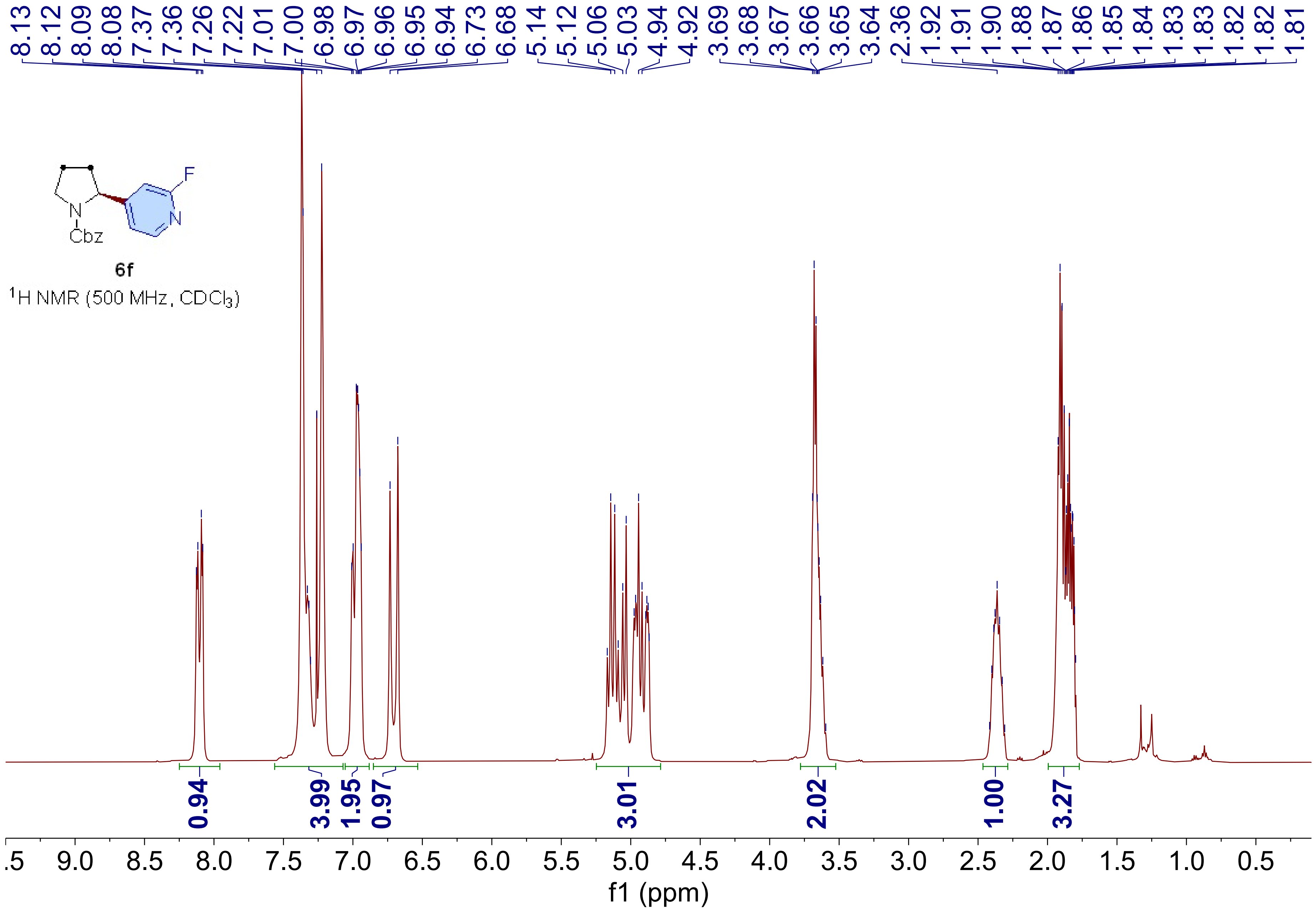
8.13 8.12 8.09 8.08 7.37 7.36 7.26 7.22 7.01 7.00 6.98 6.97 6.96 6.95 6.94 6.73 6.68 5.14 5.12 5.06 5.03 4.94 4.92 3.69 3.68 3.67 3.66 3.65 3.64 2.36 1.92 1.91 1.90 1.88 1.87 1.86 1.85 1.84 1.83 1.83 1.82 1.82 1.81
F
N
N
Cbz
6f
1H NMR (500 MHz, CDCl3)
0.94
3.99
1.95
0.97
3.01
2.02
1.00
3.27
f1 (ppm)

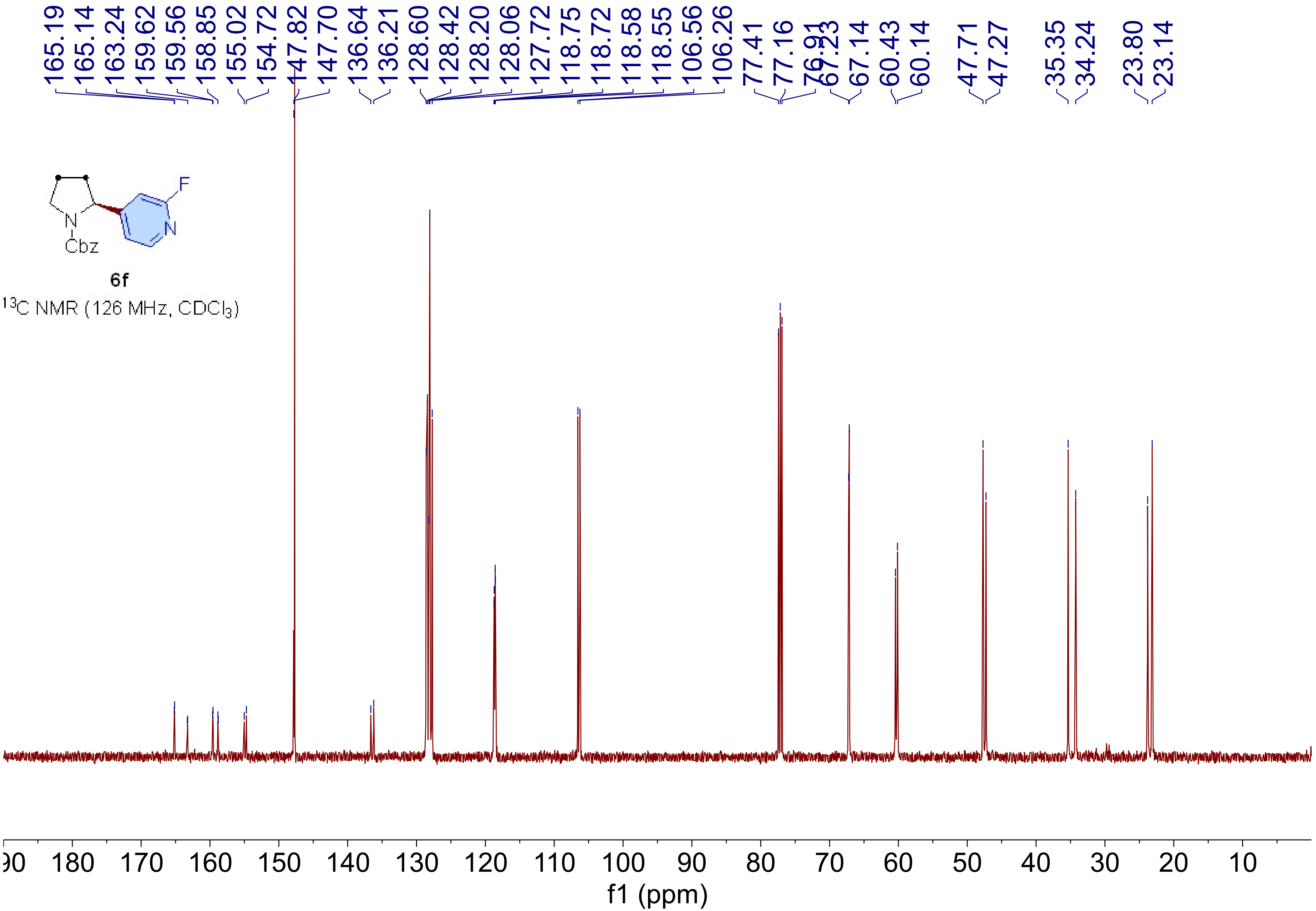

6f

^{13}C NMR (126 MHz, $CDCl_3$)

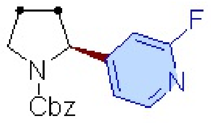

6f

^{19}F NMR (471 MHz, $CDCl_3$)

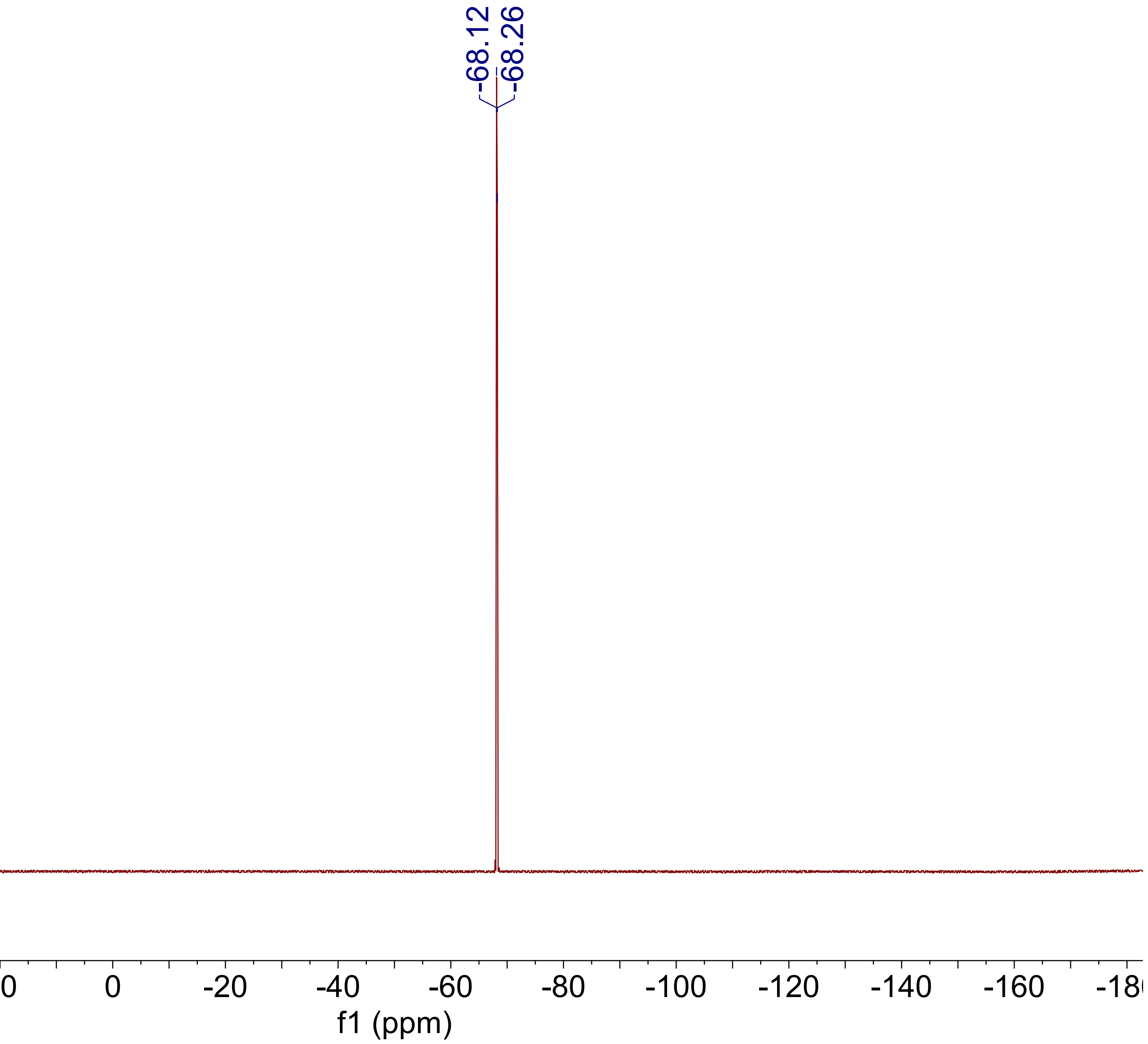

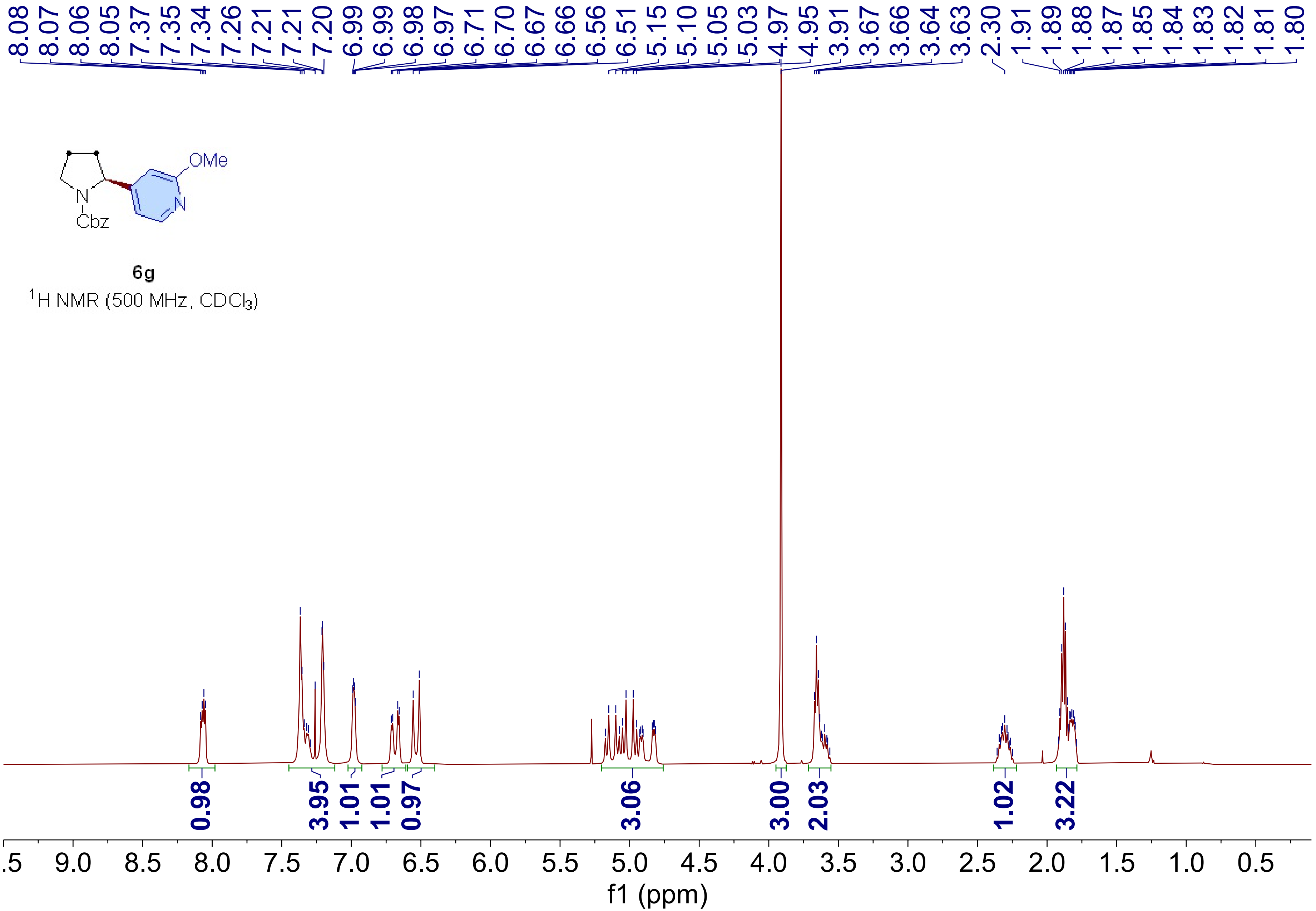
8.08 8.07 8.06 8.05 7.37 7.35 7.34 7.26 7.21 7.21 7.20 6.99 6.99 6.98 6.97 6.71 6.70 6.67 6.66 6.56 6.51 5.15 5.10 5.05 5.03 4.97 4.95 3.91 3.67 3.66 3.64 3.63 2.30 1.91 1.89 1.88 1.87 1.85 1.84 1.83 1.82 1.81 1.80
OMe
N
N
Cbz
6g
^{1}H NMR (500 MHz, CDCl$_3$)
0.98 3.95 1.01 1.01 0.97 3.06 3.00 2.03 1.02 3.22
.5 9.0 8.5 8.0 7.5 7.0 6.5 6.0 5.5 5.0 4.5 4.0 3.5 3.0 2.5 2.0 1.5 1.0 0.5
f1 (ppm)

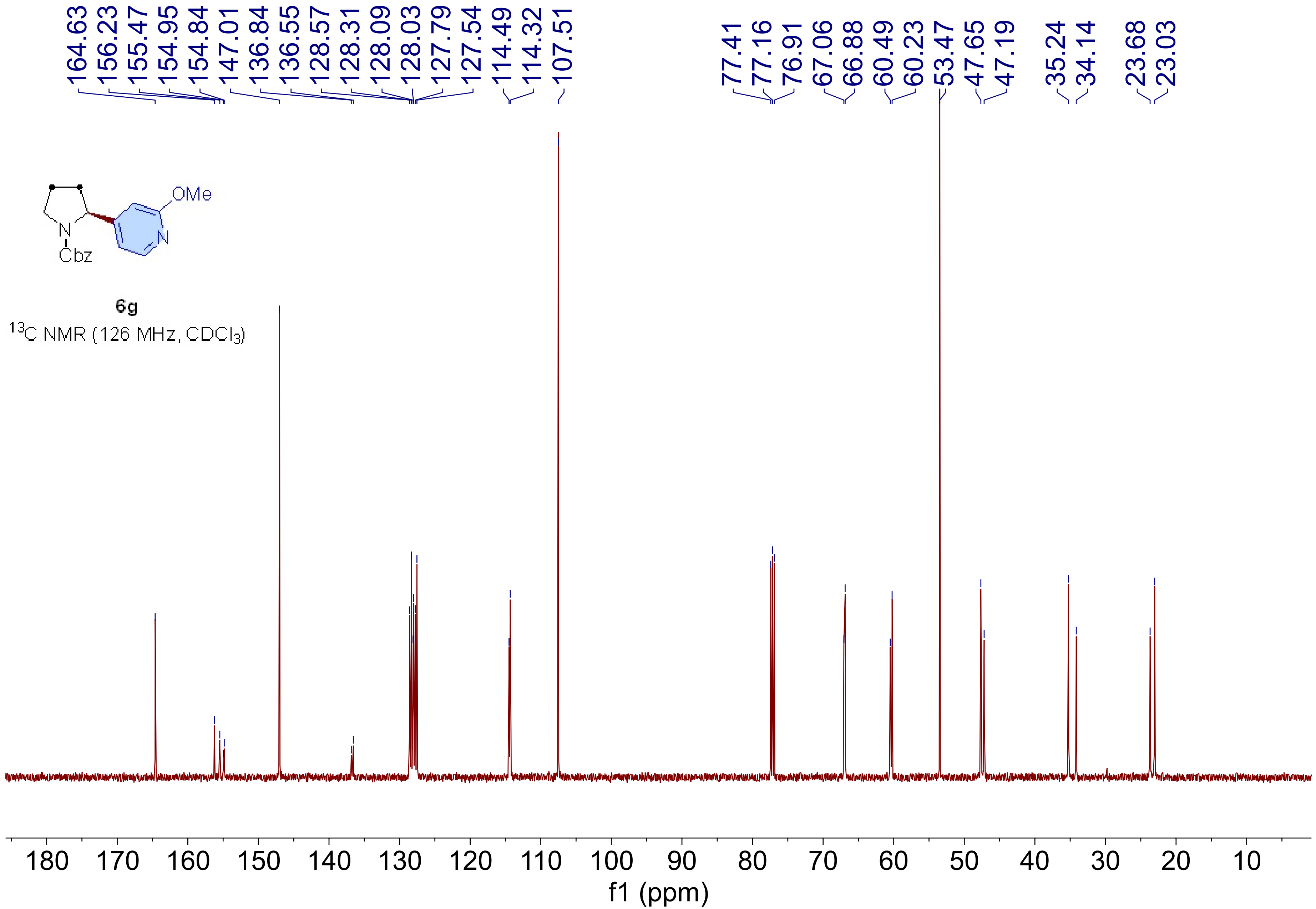

6g

^{13}C NMR (126 MHz, $CDCl_3$)

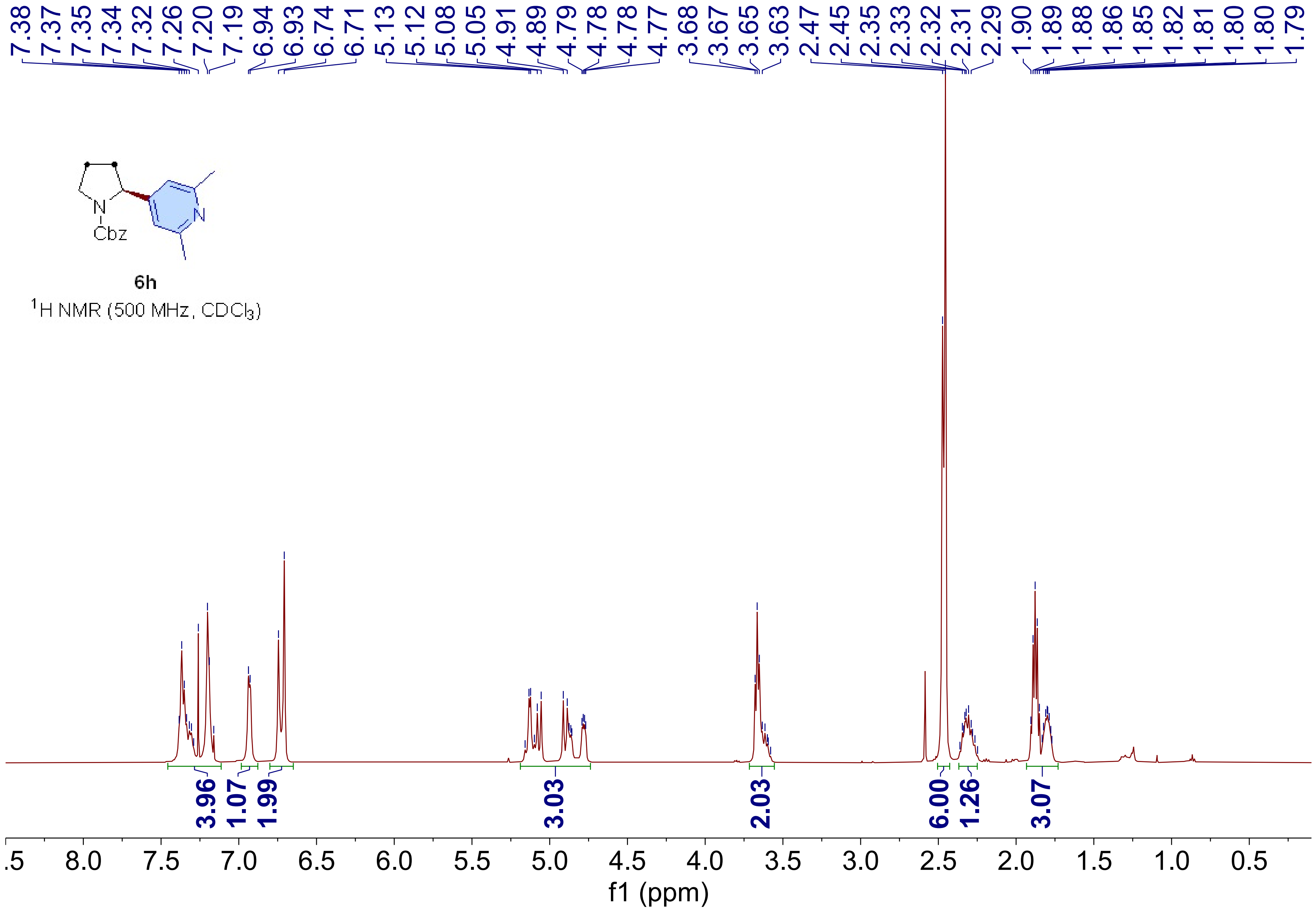
7.38 7.37 7.35 7.34 7.32 7.26 7.20 7.19 6.94 6.93 6.74 6.71 5.13 5.12 5.08 5.05 4.91 4.89 4.79 4.78 4.78 4.77 3.68 3.67 3.65 3.63 2.47 2.45 2.35 2.33 2.32 2.31 2.29 1.90 1.89 1.88 1.86 1.85 1.82 1.81 1.80 1.80 1.79
N
Cbz
N
6h
1H NMR (500 MHz, CDCl3)
3.96
1.07
1.99
3.03
2.03
6.00
1.26
3.07
.5 8.0 7.5 7.0 6.5 6.0 5.5 5.0 4.5 4.0 3.5 3.0 2.5 2.0 1.5 1.0 0.5
f1 (ppm)

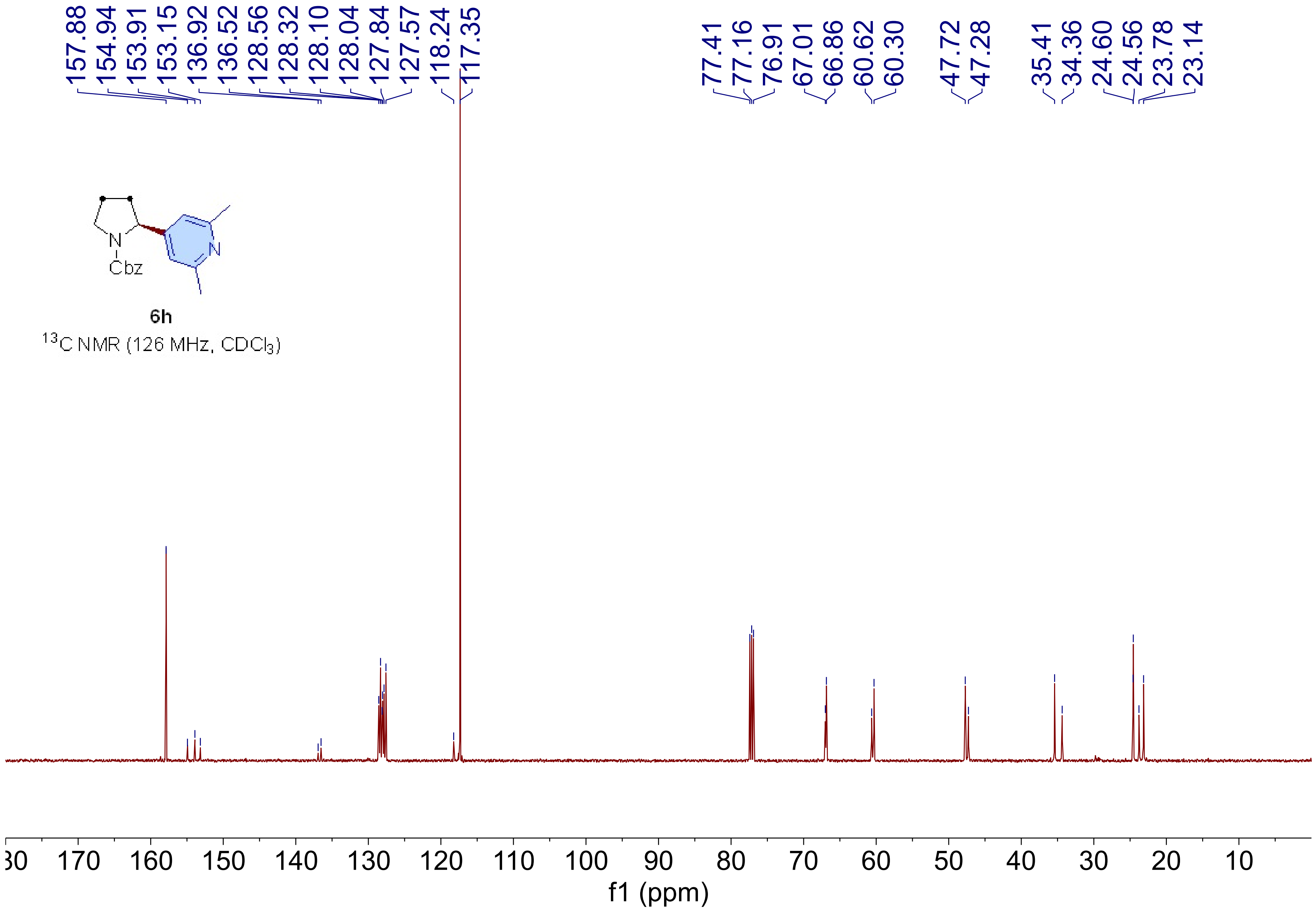
157.88
154.94
153.91
153.15
136.92
136.52
128.56
128.32
128.10
128.04
127.84
127.57
118.24
117.35
77.41
77.16
76.91
67.01
66.86
60.62
60.30
47.72
47.28
35.41
34.36
24.60
24.56
23.78
23.14
N
Cbz
N
6h
13C NMR (126 MHz, CDCl3)
f1 (ppm)

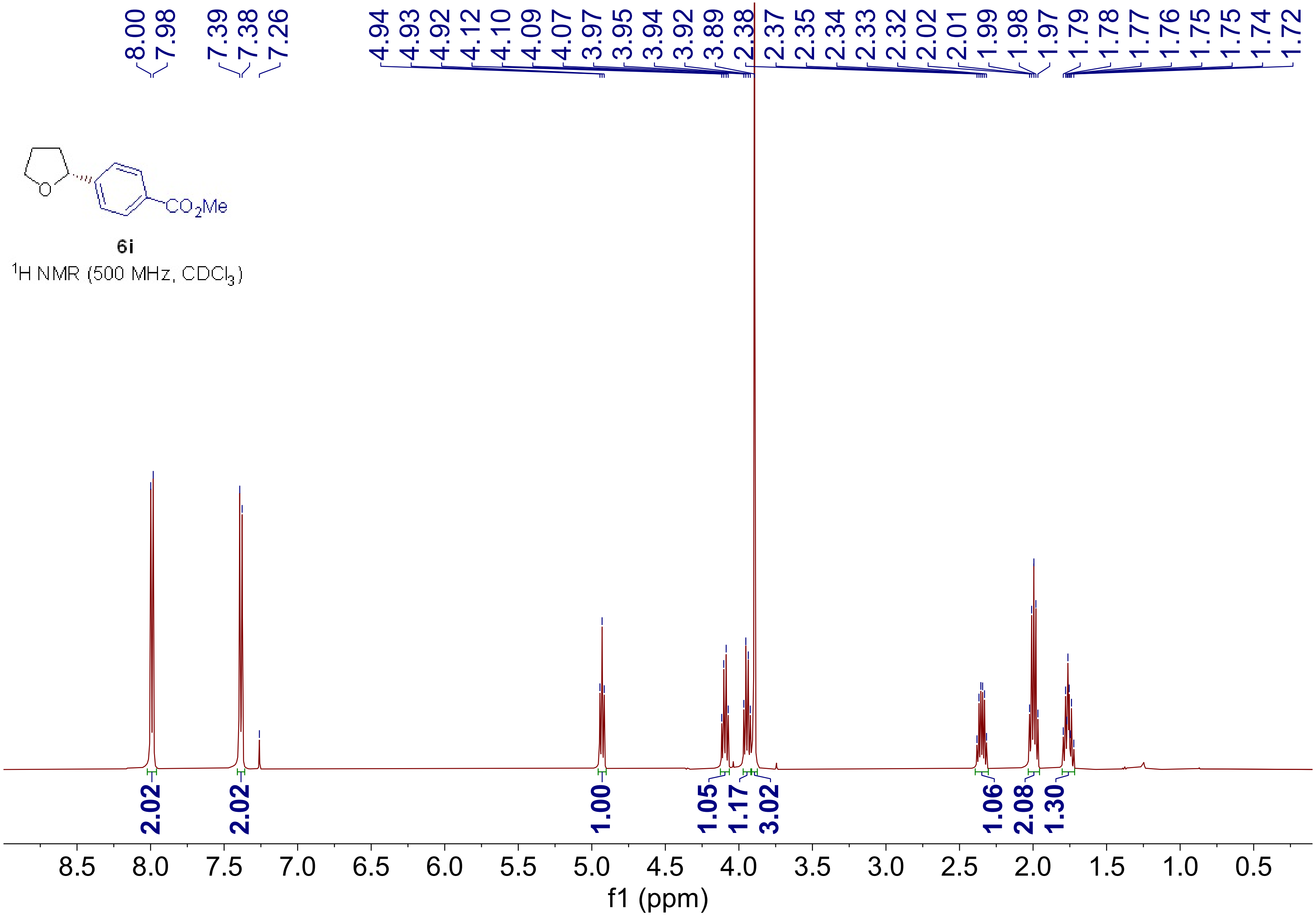
8.00
7.98
7.39
7.38
7.26
4.94
4.93
4.92
4.12
4.10
4.09
4.07
3.97
3.95
3.94
3.92
3.89
2.38
2.37
2.35
2.34
2.33
2.32
2.02
2.01
1.99
1.98
1.97
1.79
1.78
1.77
1.76
1.75
1.75
1.74
1.72
CO2Me
O
6i
1H NMR (500 MHz, CDCl3)
2.02
2.02
1.00
1.05
1.17
3.02
1.06
2.08
1.30
8.5
8.0
7.5
7.0
6.5
6.0
5.5
5.0
4.5
4.0
3.5
3.0
2.5
2.0
1.5
1.0
0.5
f1 (ppm)

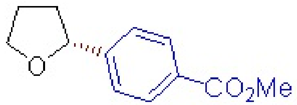

6i

^{13}C NMR (126 MHz, $CDCl_3$)

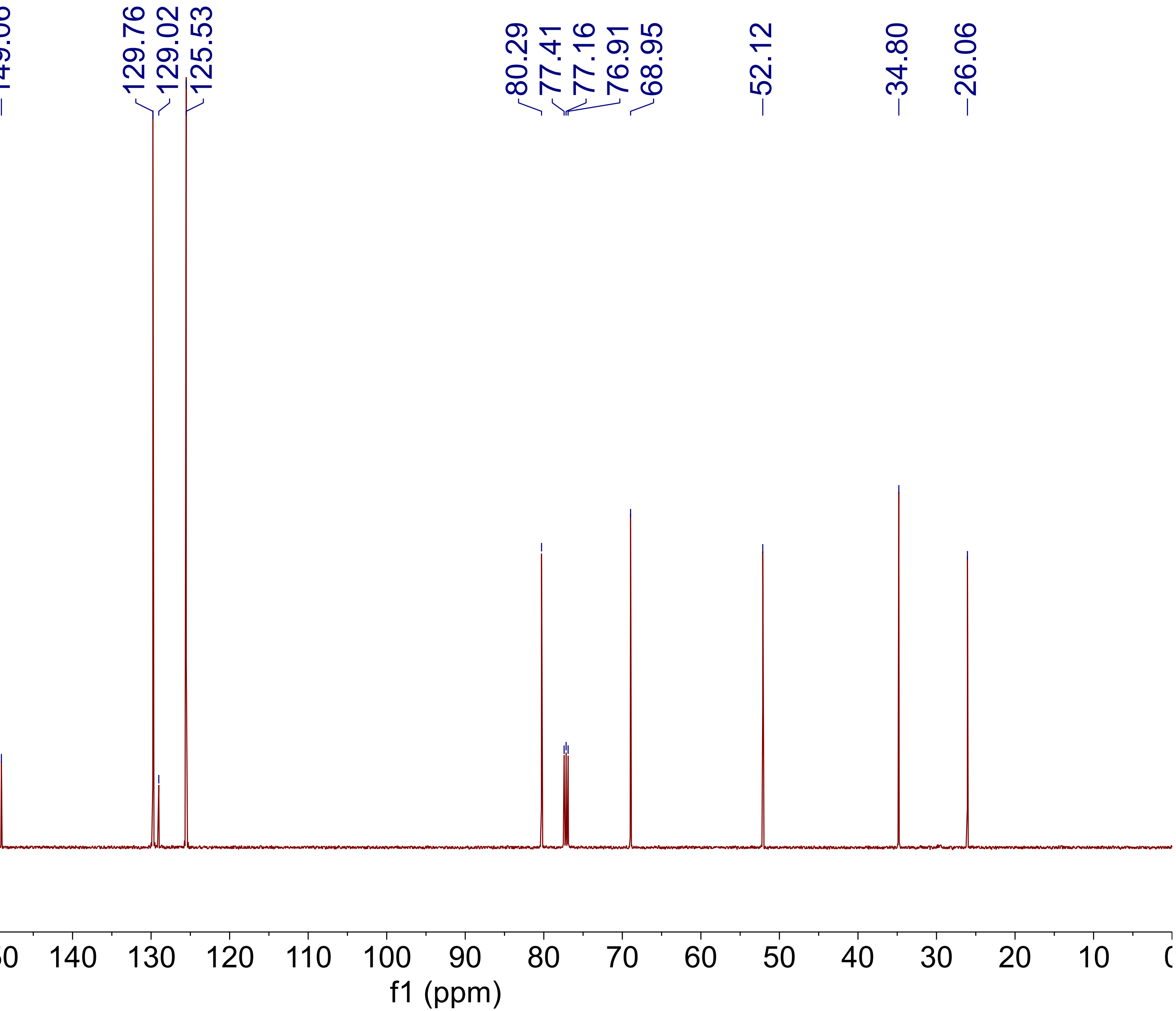

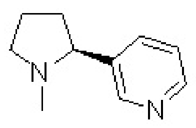

7

^{1}H NMR (500 MHz, $CDCl_3$)

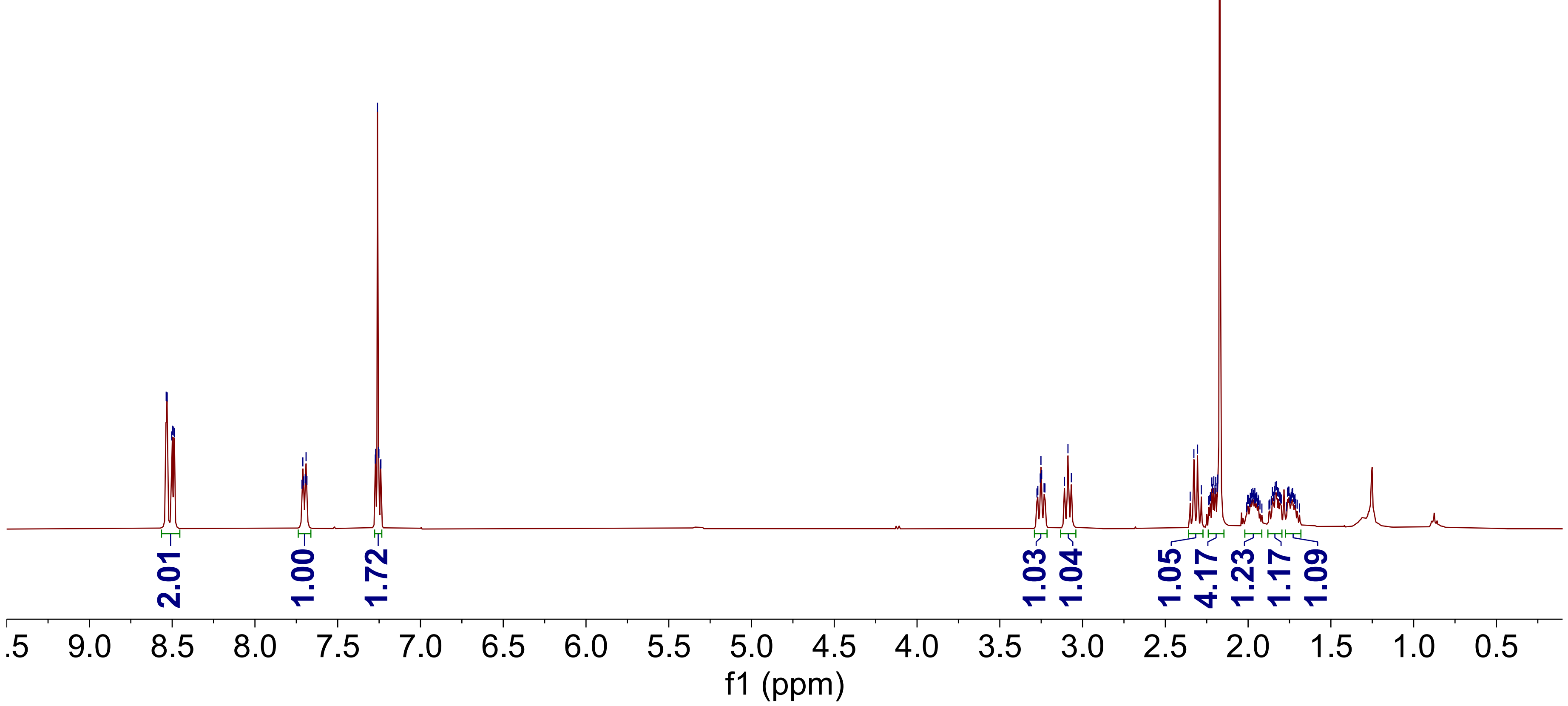

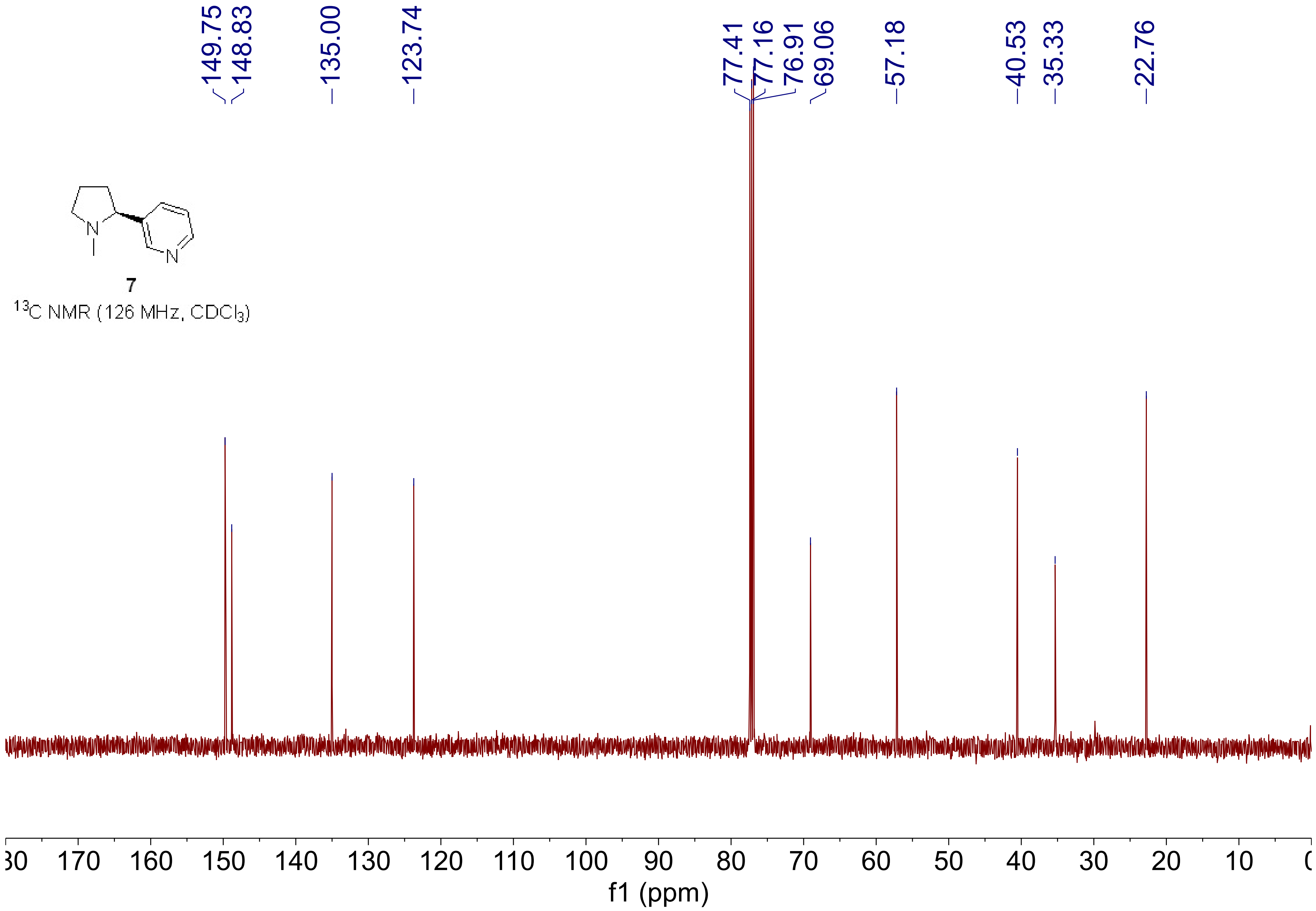

7

^{13}C NMR (126 MHz, $CDCl_3$)

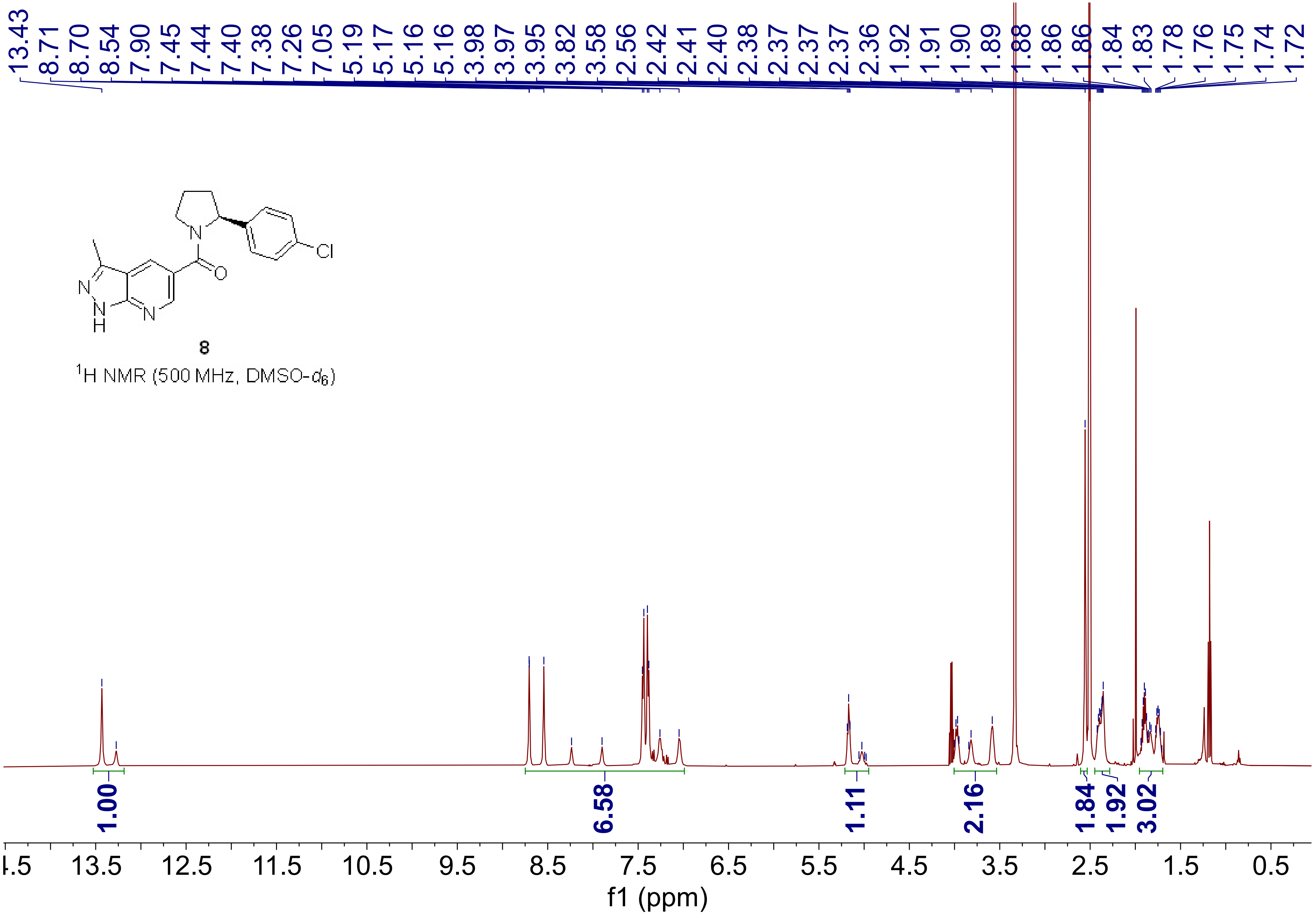

8

^{1}H NMR (500 MHz, DMSO-d_6)

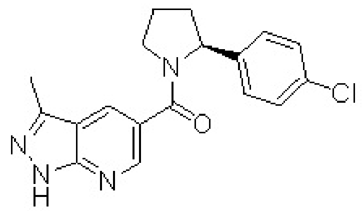

8

^{13}C NMR (126 MHz, DMSO-d_6)

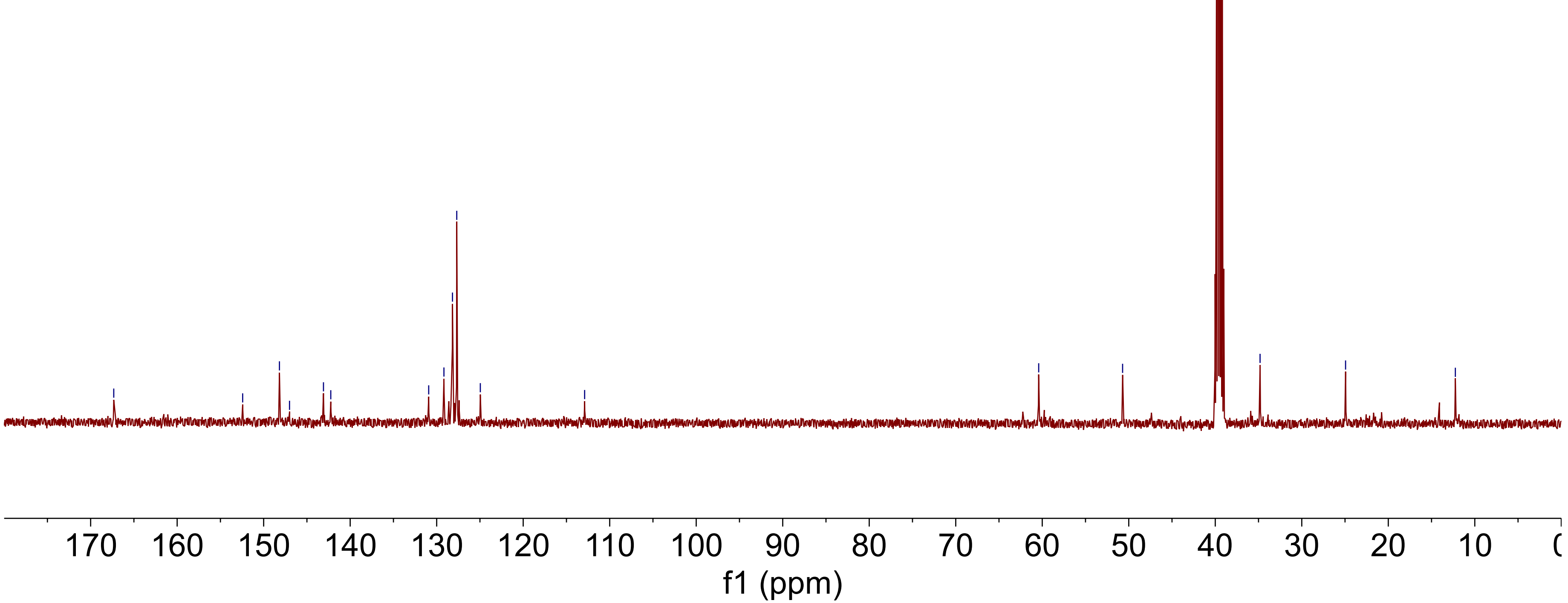

11. Spectroscopic Data (HPLC Spectrum)

```
Data File E:\DATA\20221017\LC 2022-10-30 16-37-11\OnlineEdited--015.D
Sample Name: ZJQ-02-26-1

    =====================================================================
    Acq. Operator   : SYSTEM                          Seq. Line :  15
    Acq. Instrument : HPLC1260                         Location :   P2-F1
    Injection Date  : 10/30/2022 10:45:54 PM                Inj :   1
                                                     Inj Volume : 3.000 µl
    Different Inj Volume from Sample Entry!  Actual Inj Volume : 1.000 µl
    Acq. Method     : E:\DATA\20221017\LC 2022-10-30 16-37-11\20IPA-20-0.8-1-ZJQ.M
    Last changed    : 10/30/2022 6:40:03 PM by SYSTEM
    Analysis Method : E:\DATA\20221017\LC 2022-10-30 16-37-11\20IPA-20-0.8-1-ZJQ.M (Sequence
                      Method)
    Last changed    : 10/31/2022 8:34:03 AM by SYSTEM
                      (modified after loading)
    Additional Info : Peak(s) manually integrated
```

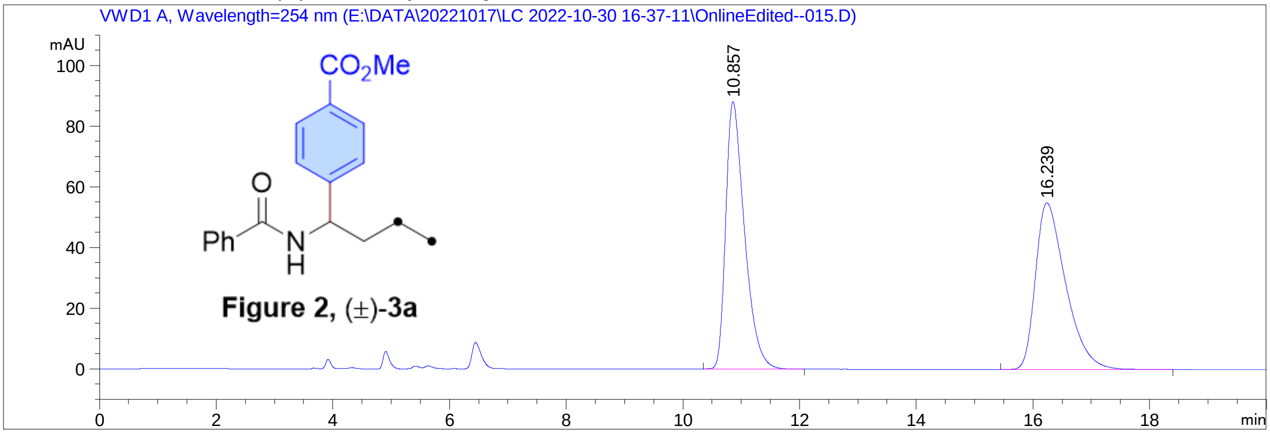

Figure 2, (±)-3a

```
    =====================================================================
                             Area Percent Report
    =====================================================================

    Sorted By             :      Signal
    Multiplier            :      1.0000
    Dilution              :      1.0000
    Do not use Multiplier & Dilution Factor with ISTDs


    Signal 1: VWD1 A, Wavelength=254 nm

    Peak RetTime Type  Width     Area      Height     Area
      #   [min]        [min]   [mAU*s]     [mAU]        %
    ----|-------|----|-------|----------|----------|--------|
       1  10.857 BB    0.3367 1967.49341   88.04835  49.9519
       2  16.239 BB    0.5418 1971.28052   54.90851  50.0481

    Totals :                  3938.77393  142.95686


    =====================================================================
                              *** End of Report ***
```

Data File E:\DATA\20220901\LC 2022-09-23 21-30-36\OnlineEdited--002.D
Sample Name: ZJQ-01-191-1

===

Acq. Operator : SYSTEM Seq. Line : 2
Acq. Instrument : HPLC1260 Location : P2-F1
Injection Date : 9/23/2022 9:53:08 PM Inj : 1
Inj Volume : 3.000 µl
Different Inj Volume from Sample Entry! Actual Inj Volume : 1.000 µl
Acq. Method : E:\DATA\20220901\LC 2022-09-23 21-30-36\20IPA-20-0.8-1-ZJQ.M
Last changed : 9/23/2022 9:30:36 PM by SYSTEM
Analysis Method : E:\DATA\20220901\LC 2022-09-23 21-30-36\20IPA-20-0.8-1-ZJQ.M (Sequence Method)
Last changed : 10/31/2022 11:00:38 AM by SYSTEM
(modified after loading)
Additional Info : Peak(s) manually integrated

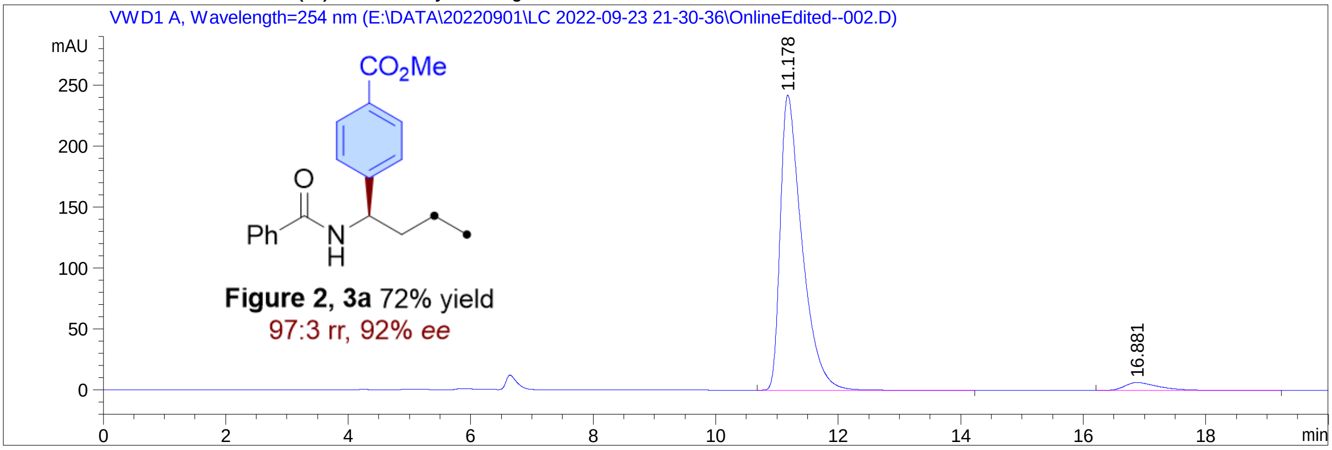

===

Area Percent Report

===

Sorted By : Signal
Multiplier : 1.0000
Dilution : 1.0000
Do not use Multiplier & Dilution Factor with ISTDs

Signal 1: VWD1 A, Wavelength=254 nm

Peak #	RetTime [min]	Type	Width [min]	Area [mAU*s]	Height [mAU]	Area %
1	11.178	BB	0.3613	5967.20313	242.30453	95.9658
2	16.881	BB	0.5587	250.84984	6.50734	4.0342

Totals : 6218.05293 248.81188

===

*** End of Report ***

Data File E:\DATA\20220901\LC 2022-09-26 13-52-37\OnlineEdited--013.D
Sample Name: ZJQ-01-61-8-RAC

===

Acq. Operator : SYSTEM Seq. Line : 13
Acq. Instrument : HPLC1260 Location : P2-A5
Injection Date : 9/26/2022 6:27:19 PM Inj : 1
Inj Volume : 3.000 µl
Different Inj Volume from Sample Entry! Actual Inj Volume : 1.000 µl
Acq. Method : E:\DATA\20220901\LC 2022-09-26 13-52-37\20IPA-20-0.8-1-ZJQ.M
Last changed : 9/26/2022 5:46:47 PM by SYSTEM
Analysis Method : E:\DATA\20220901\LC 2022-09-26 13-52-37\20IPA-20-0.8-1-ZJQ.M (Sequence Method)
Last changed : 11/1/2022 9:12:04 PM by SYSTEM
(modified after loading)
Additional Info : Peak(s) manually integrated

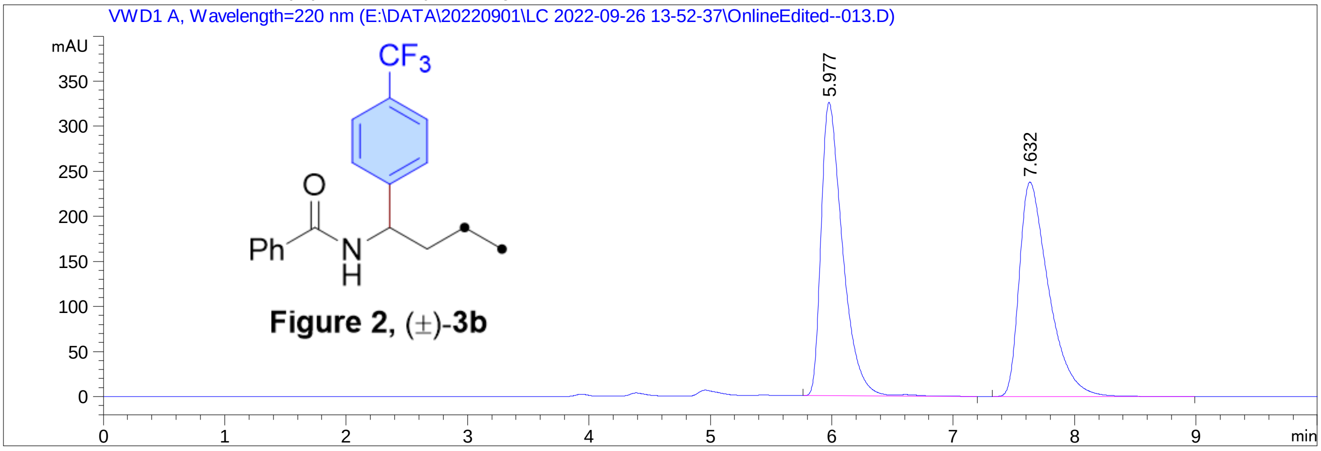

===

Area Percent Report

===

Sorted By : Signal
Multiplier : 1.0000
Dilution : 1.0000
Do not use Multiplier & Dilution Factor with ISTDs

Signal 1: VWD1 A, Wavelength=220 nm

Peak #	RetTime [min]	Type	Width [min]	Area [mAU*s]	Height [mAU]	Area %
1	5.977	BV R	0.1847	4012.39355	325.64807	50.0576
2	7.632	BB	0.2523	4003.16162	237.89633	49.9424

Totals : 8015.55518 563.54440

===

*** End of Report ***

Data File E:\DATA\20220901\LC 2022-09-26 13-52-37\OnlineEdited--014.D
Sample Name: ZJQ-01-194-1

===

Acq. Operator : SYSTEM Seq. Line : 14
Acq. Instrument : HPLC1260 Location : P2-A6
Injection Date : 9/26/2022 6:48:03 PM Inj : 1
Inj Volume : 3.000 µl
Different Inj Volume from Sample Entry! Actual Inj Volume : 1.000 µl
Acq. Method : E:\DATA\20220901\LC 2022-09-26 13-52-37\20IPA-20-0.8-1-ZJQ.M
Last changed : 9/26/2022 5:46:47 PM by SYSTEM
Analysis Method : E:\DATA\20220901\LC 2022-09-26 13-52-37\20IPA-20-0.8-1-ZJQ.M (Sequence Method)
Last changed : 11/1/2022 9:13:57 PM by SYSTEM
(modified after loading)
Additional Info : Peak(s) manually integrated

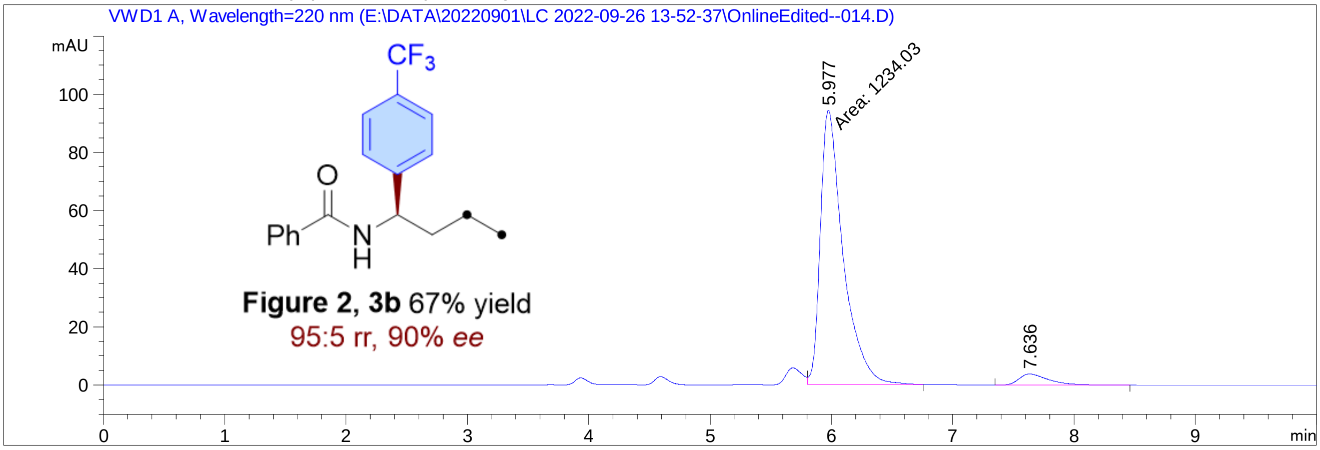

===

Area Percent Report

===

Sorted By : Signal
Multiplier : 1.0000
Dilution : 1.0000
Do not use Multiplier & Dilution Factor with ISTDs

Signal 1: VWD1 A, Wavelength=220 nm

Peak #	RetTime [min]	Type	Width [min]	Area [mAU*s]	Height [mAU]	Area %
1	5.977	FM	0.2180	1234.02856	94.33205	95.1555
2	7.636	BB	0.2492	62.82555	3.77481	4.8445

Totals : 1296.85411 98.10685

===

*** End of Report ***

Data File E:\DATA\20221104\LC 2022-11-17 16-41-34\OnlineEdited--045.D
Sample Name: ZJQ-02-37-2-RAC

===

Acq. Operator : SYSTEM Seq. Line : 45
Acq. Instrument : HPLC1260 Location : P2-F8
Injection Date : 11/18/2022 1:45:31 PM Inj : 1
Inj Volume : 3.000 µl
Different Inj Volume from Sample Entry! Actual Inj Volume : 1.000 µl
Acq. Method : E:\DATA\20221104\LC 2022-11-17 16-41-34\5IPA-25-0.8-4-220-ZJQ.M
Last changed : 11/18/2022 8:16:35 AM by SYSTEM
Analysis Method : E:\DATA\20221104\LC 2022-11-17 16-41-34\5IPA-25-0.8-4-220-ZJQ.M (Sequence Method)
Last changed : 11/18/2022 2:40:32 PM by SYSTEM
(modified after loading)
Additional Info : Peak(s) manually integrated

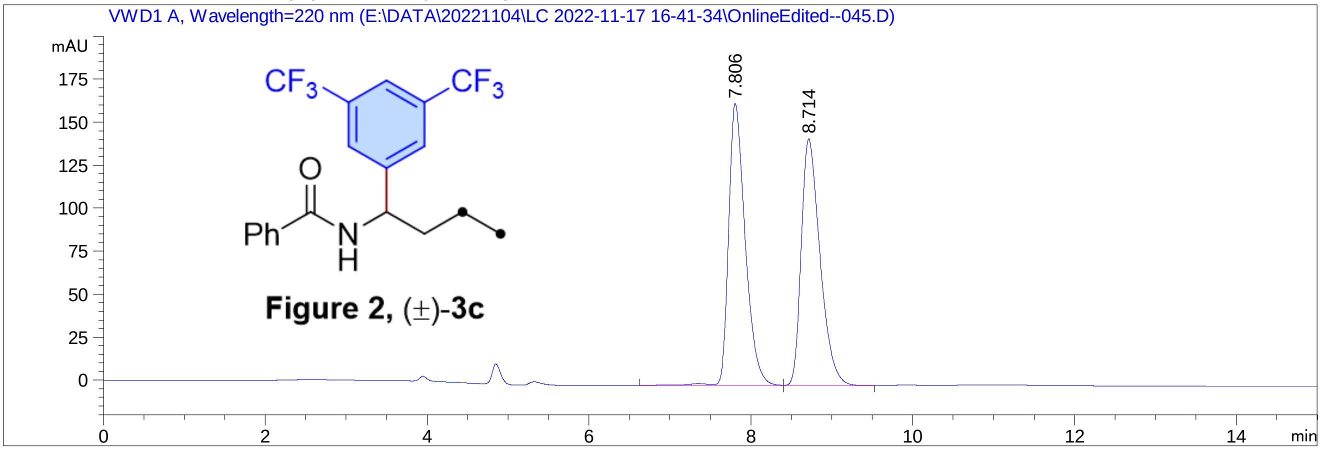

===

Area Percent Report

===

Sorted By : Signal
Multiplier : 1.0000
Dilution : 1.0000
Do not use Multiplier & Dilution Factor with ISTDs

Signal 1: VWD1 A, Wavelength=220 nm

Peak #	RetTime [min]	Type	Width [min]	Area [mAU*s]	Height [mAU]	Area %
1	7.806	VB R	0.2155	2338.08765	163.82365	50.2434
2	8.714	BB	0.2453	2315.43018	143.45453	49.7566
Totals :				4653.51782	307.27818	

===

*** End of Report ***

Data File E:\DATA\20221104\LC 2022-11-17 16-41-34\OnlineEdited--046.D
Sample Name: ZJQ-02-36-2

===

Acq. Operator : SYSTEM Seq. Line : 46
Acq. Instrument : HPLC1260 Location : P2-F9
Injection Date : 11/18/2022 2:11:17 PM Inj : 1
Inj Volume : 3.000 µl
Different Inj Volume from Sample Entry! Actual Inj Volume : 0.600 µl
Acq. Method : E:\DATA\20221104\LC 2022-11-17 16-41-34\5IPA-25-0.8-4-220-ZJQ.M
Last changed : 11/18/2022 2:12:51 PM by SYSTEM
(modified after loading)
Analysis Method : E:\DATA\20221104\LC 2022-11-17 16-41-34\5IPA-25-0.8-4-220-ZJQ.M (Sequence Method)
Last changed : 11/18/2022 2:39:42 PM by SYSTEM
(modified after loading)
Additional Info : Peak(s) manually integrated

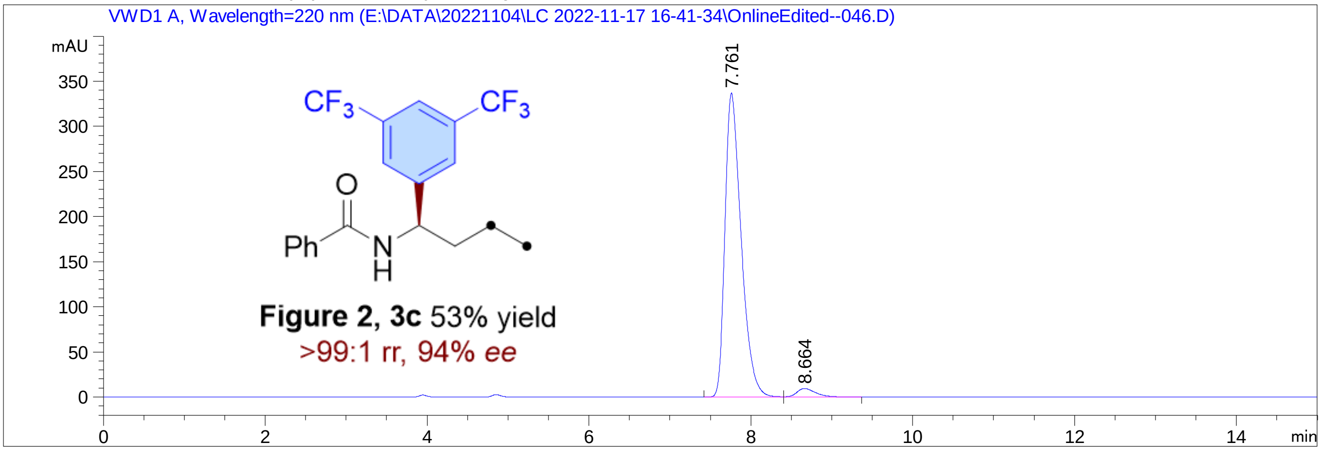

===

Area Percent Report

===

Sorted By : Signal
Multiplier : 1.0000
Dilution : 1.0000
Do not use Multiplier & Dilution Factor with ISTDs

Signal 1: VWD1 A, Wavelength=220 nm

Peak #	RetTime [min]	Type	Width [min]	Area [mAU*s]	Height [mAU]	Area %
1	7.761	BV	0.2112	4683.36719	336.92566	96.8750
2	8.664	VB	0.2426	151.07481	9.44736	3.1250

Totals : 4834.44200 346.37302

===

*** End of Report ***

Data File E:\DATA\20221017\LC 2022-10-30 16-37-11\OnlineEdited--016.D
Sample Name: ZJQ-02-26-8

===

Acq. Operator : SYSTEM   Seq. Line : 16
Acq. Instrument : HPLC1260   Location : P2-F3
Injection Date : 10/30/2022 11:06:40 PM   Inj : 1
Inj Volume : 3.000 µl
Different Inj Volume from Sample Entry! Actual Inj Volume : 1.000 µl
Acq. Method : E:\DATA\20221017\LC 2022-10-30 16-37-11\20IPA-20-0.8-1-ZJQ.M
Last changed : 10/30/2022 6:40:03 PM by SYSTEM
Analysis Method : E:\DATA\20221017\LC 2022-10-30 16-37-11\20IPA-20-0.8-1-ZJQ.M (Sequence Method)
Last changed : 10/31/2022 8:36:14 AM by SYSTEM
(modified after loading)
Additional Info : Peak(s) manually integrated

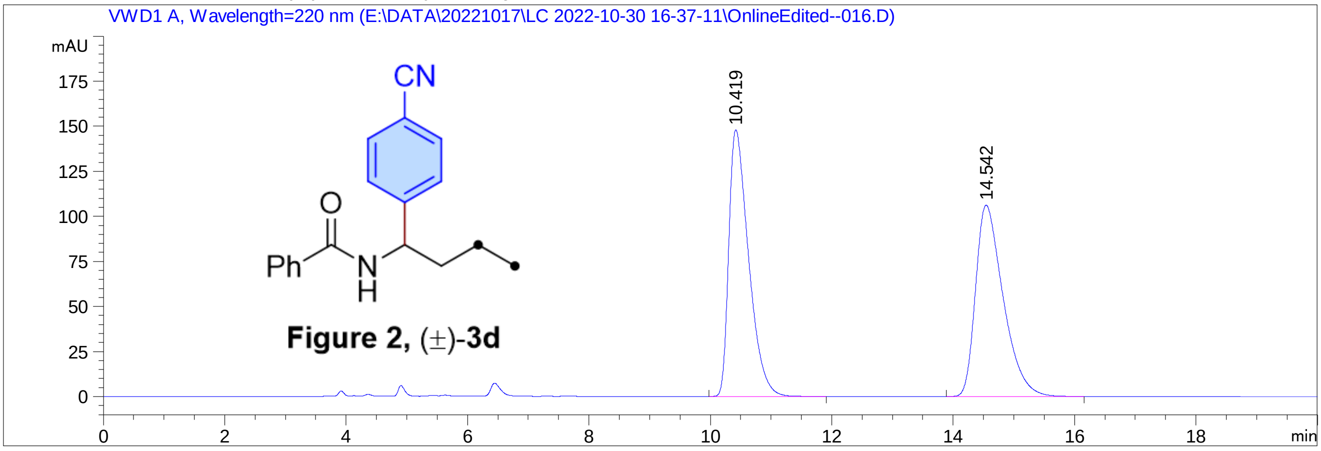

Figure 2, (±)-3d

===

Area Percent Report

===

Sorted By : Signal
Multiplier : 1.0000
Dilution : 1.0000
Do not use Multiplier & Dilution Factor with ISTDs

Signal 1: VWD1 A, Wavelength=220 nm

Peak #	RetTime [min]	Type	Width [min]	Area [mAU*s]	Height [mAU]	Area %
1	10.419	BB	0.3447	3344.90869	148.00951	49.9198
2	14.542	BB	0.4766	3355.65747	106.20735	50.0802

Totals : 6700.56616 254.21686

===

*** End of Report ***

Data File E:\DATA\20221104\LC 2022-11-22 21-33-41\2EK-0201.D
Sample Name: ZJQ-01-202-2

===

Acq. Operator : SYSTEM Seq. Line : 2
Acq. Instrument : HPLC1260 Location : P2-E11
Injection Date : 11/22/2022 9:56:13 PM Inj : 1
Inj Volume : 3.000 µl
Different Inj Volume from Sample Entry! Actual Inj Volume : 0.800 µl
Acq. Method : E:\DATA\20221104\LC 2022-11-22 21-33-41\20IPA-20-0.8-1-ZJQ.M
Last changed : 11/22/2022 9:33:41 PM by SYSTEM
Analysis Method : E:\DATA\20221104\LC 2022-11-22 21-33-41\20IPA-20-0.8-1-ZJQ.M (Sequence Method)
Last changed : 11/22/2022 10:36:30 PM by SYSTEM
(modified after loading)
Additional Info : Peak(s) manually integrated

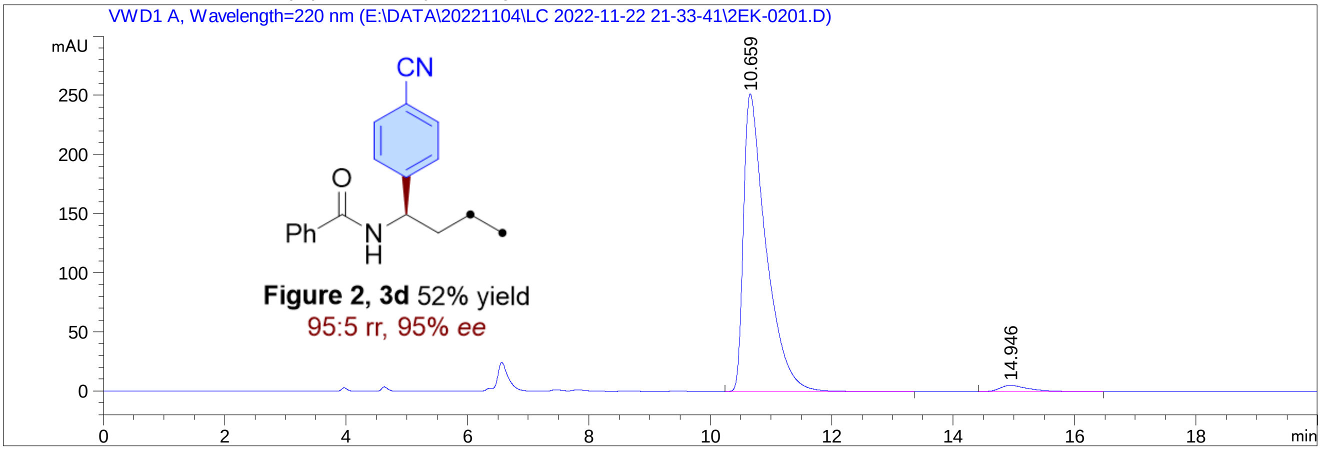

===

Area Percent Report

===

Sorted By : Signal
Multiplier : 1.0000
Dilution : 1.0000
Do not use Multiplier & Dilution Factor with ISTDs

Signal 1: VWD1 A, Wavelength=220 nm

Peak #	RetTime [min]	Type	Width [min]	Area [mAU*s]	Height [mAU]	Area %
1	10.659	BB	0.3727	6490.70801	251.82483	97.3791
2	14.946	BB	0.4767	174.69321	5.33904	2.6209

Totals : 6665.40121 257.16386

===

*** End of Report ***

Data File E:\DATA\20221017\LC 2022-10-27 14-42-40\OnlineEdited--105.D
Sample Name: ZJQ-m-CN

===

Acq. Operator : SYSTEM Seq. Line : 105
Acq. Instrument : HPLC1260 Location : P2-F4
Injection Date : 10/29/2022 7:29:19 AM Inj : 1
Inj Volume : 3.000 µl
Different Inj Volume from Sample Entry! Actual Inj Volume : 1.000 µl
Acq. Method : E:\DATA\20221017\LC 2022-10-27 14-42-40\20IPA-20-0.8-1-ZJQ.M
Last changed : 10/28/2022 11:34:48 PM by SYSTEM
Analysis Method : E:\DATA\20221017\LC 2022-10-27 14-42-40\20IPA-20-0.8-1-ZJQ.M (Sequence Method)
Last changed : 11/1/2022 9:58:53 PM by SYSTEM
(modified after loading)
Additional Info : Peak(s) manually integrated

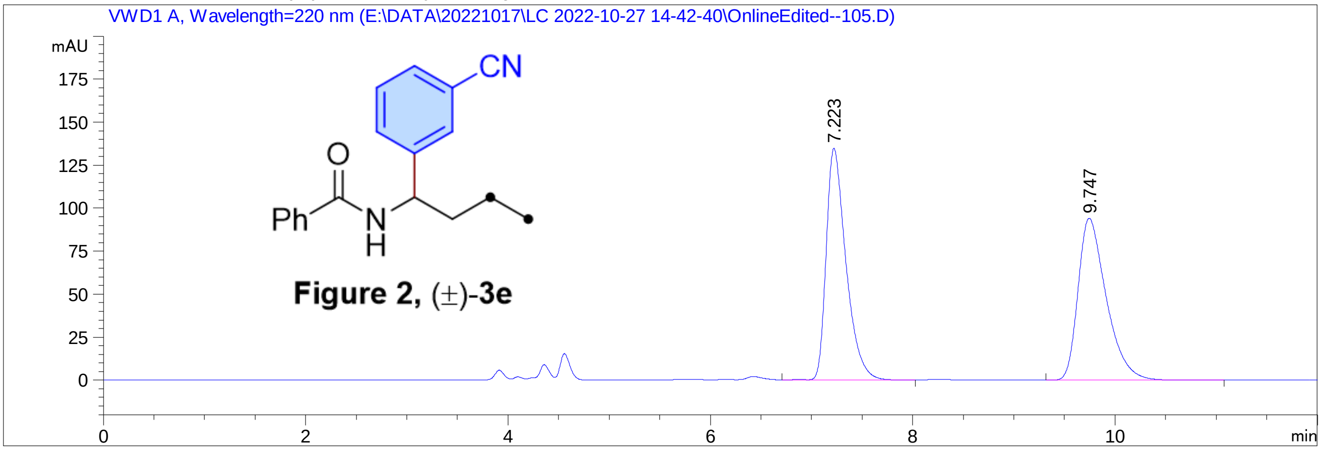

===

Area Percent Report

===

Sorted By : Signal
Multiplier : 1.0000
Dilution : 1.0000
Do not use Multiplier & Dilution Factor with ISTDs

Signal 1: VWD1 A, Wavelength=220 nm

Peak #	RetTime [min]	Type	Width [min]	Area [mAU*s]	Height [mAU]	Area %
1	7.223	VB R	0.2041	1825.46118	134.66850	50.0370
2	9.747	BB	0.2919	1822.76318	94.11681	49.9630

Totals : 3648.22437 228.78532

===

*** End of Report ***

Data File E:\DATA\20221104\LC 2022-11-22 22-53-25\OnlineEdited--003.D
Sample Name: ZJQ-01-198-1

===

Acq. Operator : SYSTEM Seq. Line : 3
Acq. Instrument : HPLC1260 Location : P2-E2
Injection Date : 11/22/2022 11:36:41 PM Inj : 1
Inj Volume : 3.000 µl
Different Inj Volume from Sample Entry! Actual Inj Volume : 0.800 µl
Acq. Method : E:\DATA\20221104\LC 2022-11-22 22-53-25\20IPA-20-0.8-1-ZJQ.M
Last changed : 11/22/2022 10:53:25 PM by SYSTEM
Analysis Method : E:\DATA\20221104\LC 2022-11-22 22-53-25\20IPA-20-0.8-1-ZJQ.M (Sequence Method)
Last changed : 11/23/2022 9:29:03 AM by SYSTEM
(modified after loading)
Additional Info : Peak(s) manually integrated

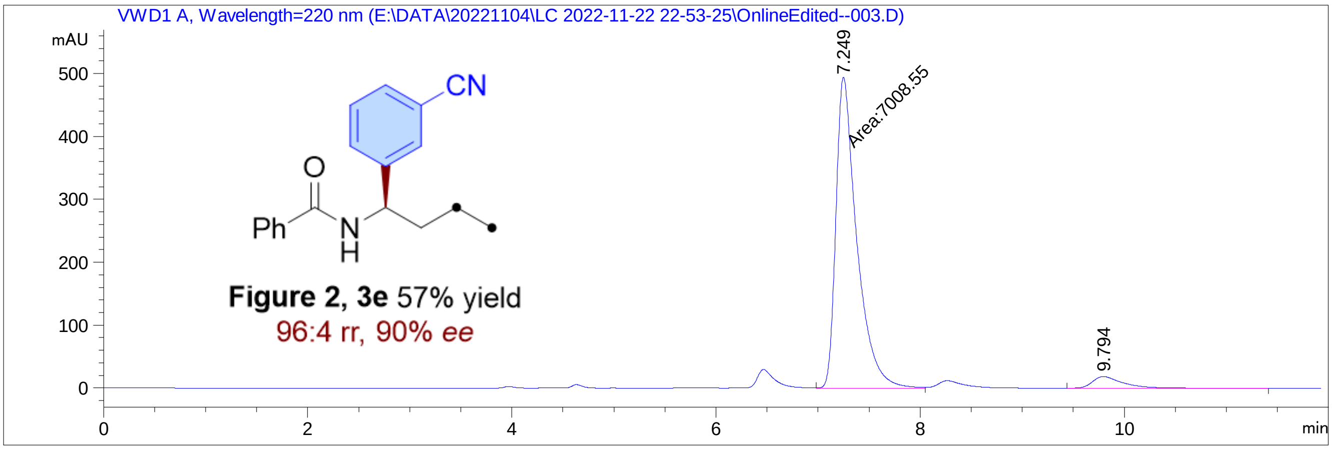

===

Area Percent Report

===

Sorted By : Signal
Multiplier : 1.0000
Dilution : 1.0000
Do not use Multiplier & Dilution Factor with ISTDs

Signal 1: VWD1 A, Wavelength=220 nm

Peak #	RetTime [min]	Type	Width [min]	Area [mAU*s]	Height [mAU]	Area %
1	7.249	MF	0.2363	7008.54834	494.37686	94.9306
2	9.794	BB	0.2940	374.26492	18.50569	5.0694

Totals : 7382.81326 512.88255

===

*** End of Report ***

Data File E:\DATA\20221216\LC 2022-12-19 10-41-20\OnlineEdited--038.D
Sample Name: ZJQ-02-28-5-RAC5

===

Acq. Operator : SYSTEM Seq. Line : 38
Acq. Instrument : HPLC1260 Location : P2-E9
Injection Date : 12/20/2022 12:59:05 AM Inj : 1
Inj Volume : 3.000 µl
Different Inj Volume from Sample Entry! Actual Inj Volume : 5.000 µl
Acq. Method : E:\DATA\20221216\LC 2022-12-19 10-41-20\20IPA-20-0.8-1-254-ZJQ.M
Last changed : 12/19/2022 7:56:55 PM by SYSTEM
Analysis Method : E:\DATA\20221216\LC 2022-12-19 10-41-20\20IPA-20-0.8-1-254-ZJQ.M (Sequence Method)
Last changed : 12/20/2022 9:19:46 AM by SYSTEM
(modified after loading)

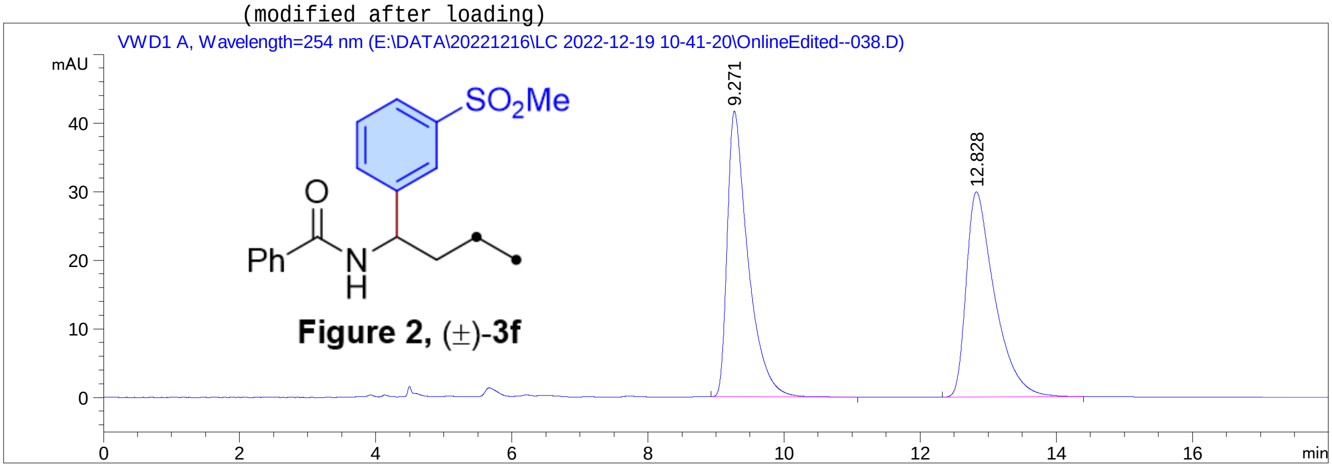

===

Area Percent Report

===

Sorted By : Signal
Multiplier : 1.0000
Dilution : 1.0000
Do not use Multiplier & Dilution Factor with ISTDs

Signal 1: VWD1 A, Wavelength=254 nm

Peak #	RetTime [min]	Type	Width [min]	Area [mAU*s]	Height [mAU]	Area %
1	9.271	BB	0.3084	882.65735	41.61573	50.3800
2	12.828	BB	0.4260	869.34131	29.92250	49.6200

Totals : 1751.99866 71.53823

===

*** End of Report ***

Data File E:\DATA\20221017\LC 2022-10-31 14-22-32\OnlineEdited--060.D
Sample Name: ZJQ-02-28-5

===

Acq. Operator : SYSTEM Seq. Line : 60
Acq. Instrument : HPLC1260 Location : P2-F3
Injection Date : 11/1/2022 10:08:04 AM Inj : 2
Inj Volume : 3.000 µl
Different Inj Volume from Sample Entry! Actual Inj Volume : 1.000 µl
Acq. Method : E:\DATA\20221017\LC 2022-10-31 14-22-32\20IPA-20-0.8-1-ZJQ.M
Last changed : 11/1/2022 10:10:01 AM by SYSTEM
(modified after loading)
Analysis Method : E:\DATA\20221017\LC 2022-10-31 14-22-32\20IPA-20-0.8-1-ZJQ.M (Sequence Method)
Last changed : 11/22/2022 10:43:22 PM by SYSTEM
(modified after loading)
Additional Info : Peak(s) manually integrated

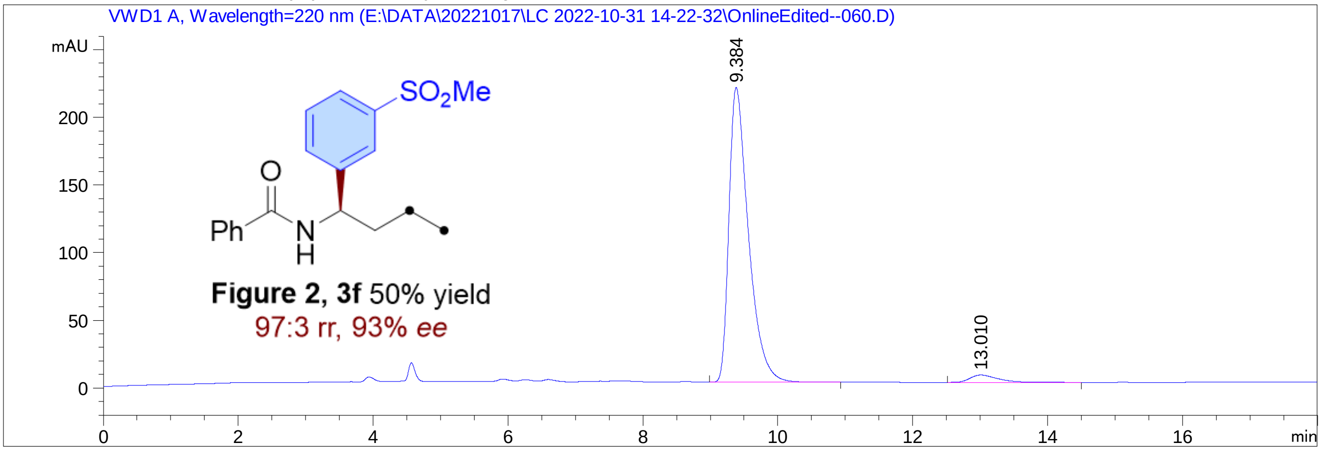

===

Area Percent Report

===

Sorted By : Signal
Multiplier : 1.0000
Dilution : 1.0000
Do not use Multiplier & Dilution Factor with ISTDs

Signal 1: VWD1 A, Wavelength=220 nm

Peak #	RetTime [min]	Type	Width [min]	Area [mAU*s]	Height [mAU]	Area %
1	9.384	BB	0.3003	4378.94775	218.03883	96.5983
2	13.010	BB	0.4285	154.20392	5.40987	3.4017

Totals : 4533.15167 223.44870

===

*** End of Report ***

Data File E:\DATA\20221017\LC 2022-11-02 13-33-52\OnlineEdited--011.D
Sample Name: ZJQ-02-26-4

===

Acq. Operator : SYSTEM Seq. Line : 11
Acq. Instrument : HPLC1260 Location : P2-A1
Injection Date : 11/2/2022 6:00:34 PM Inj : 1
Inj Volume : 3.000 µl
Different Inj Volume from Sample Entry! Actual Inj Volume : 1.000 µl
Acq. Method : E:\DATA\20221017\LC 2022-11-02 13-33-52\20IPA-25-0.8-1-ZJQ.M
Last changed : 11/2/2022 3:49:47 PM by SYSTEM
Analysis Method : E:\DATA\20221017\LC 2022-11-02 13-33-52\20IPA-25-0.8-1-ZJQ.M (Sequence Method)
Last changed : 11/21/2022 9:53:29 PM by SYSTEM
(modified after loading)
Additional Info : Peak(s) manually integrated

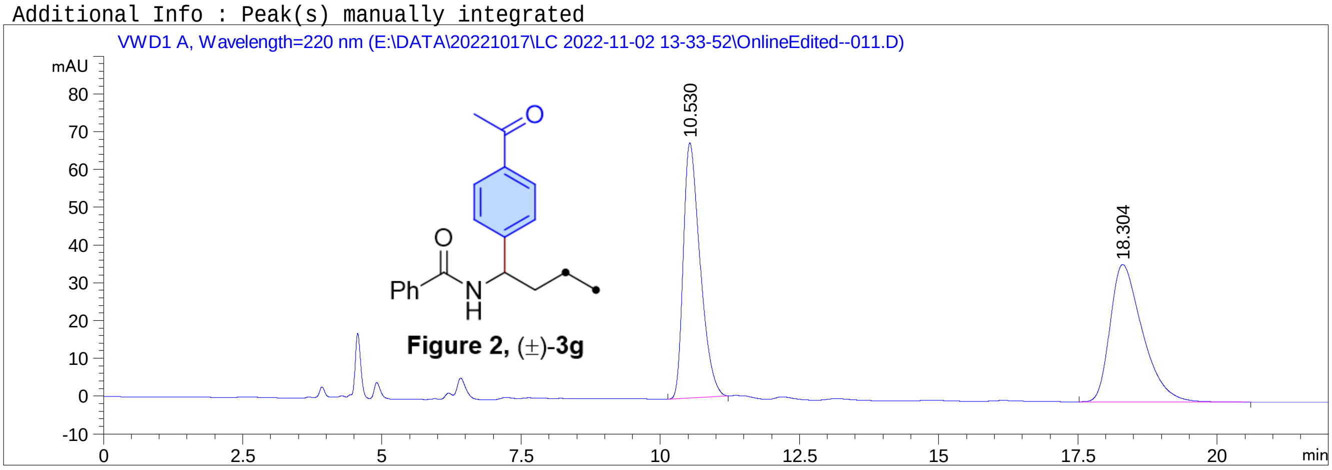

Figure 2, (±)-**3g**

===

Area Percent Report

===

Sorted By : Signal
Multiplier : 1.0000
Dilution : 1.0000
Do not use Multiplier & Dilution Factor with ISTDs

Signal 1: VWD1 A, Wavelength=220 nm

Peak #	RetTime [min]	Type	Width [min]	Area [mAU*s]	Height [mAU]	Area %
1	10.530	BB	0.3190	1424.62341	67.55287	49.2356
2	18.304	BB	0.6112	1468.85889	36.36516	50.7644

Totals : 2893.48230 103.91803

===

*** End of Report ***

Data File E:\DATA\20221017\LC 2022-10-19 09-38-25\OnlineEdited--015.D
Sample Name: ZJQ-01-198-4

===

Acq. Operator : SYSTEM Seq. Line : 15
Acq. Instrument : HPLC1260 Location : P2-D2
Injection Date : 10/19/2022 3:06:14 PM Inj : 1
Inj Volume : 3.000 µl
Different Inj Volume from Sample Entry! Actual Inj Volume : 1.000 µl
Acq. Method : E:\DATA\20221017\LC 2022-10-19 09-38-25\20IPA-25-0.8-1-ZJQ.M
Last changed : 10/19/2022 10:40:16 AM by SYSTEM
Analysis Method : E:\DATA\20221017\LC 2022-10-19 09-38-25\20IPA-25-0.8-1-ZJQ.M (Sequence Method)
Last changed : 11/1/2022 9:46:11 PM by SYSTEM
(modified after loading)
Additional Info : Peak(s) manually integrated

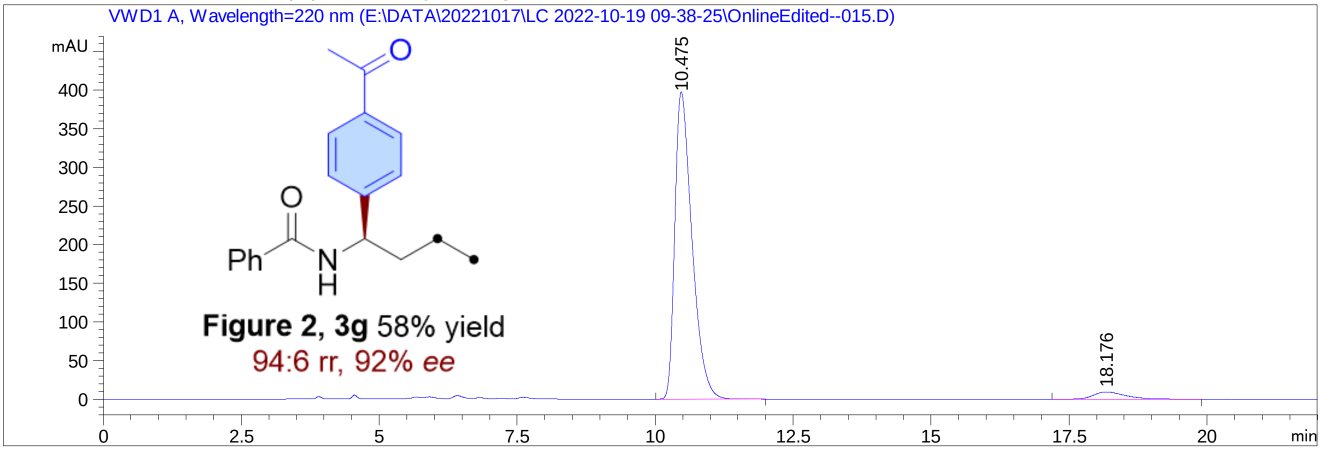

===

Area Percent Report

===

Sorted By : Signal
Multiplier : 1.0000
Dilution : 1.0000
Do not use Multiplier & Dilution Factor with ISTDs

Signal 1: VWD1 A, Wavelength=220 nm

Peak #	RetTime [min]	Type	Width [min]	Area [mAU*s]	Height [mAU]	Area %
1	10.475	BB	0.3278	8585.12305	397.84824	95.8353
2	18.176	BB	0.5942	373.08630	9.37589	4.1647

Totals : 8958.20935 407.22413

===

*** End of Report ***

Data File E:\DATA\20221216\LC 2022-12-19 10-41-20\OnlineEdited--036.D
Sample Name: ZJQ-01-194-3-RAC

===

Acq. Operator : SYSTEM Seq. Line : 36
Acq. Instrument : HPLC1260 Location : P2-E7
Injection Date : 12/19/2022 11:57:24 PM Inj : 1
Inj Volume : 3.000 µl
Different Inj Volume from Sample Entry! Actual Inj Volume : 5.000 µl
Acq. Method : E:\DATA\20221216\LC 2022-12-19 10-41-20\20IPA-30-0.8-1-254-ZJQ.M
Last changed : 12/19/2022 7:53:06 PM by SYSTEM
Analysis Method : E:\DATA\20221216\LC 2022-12-19 10-41-20\20IPA-30-0.8-1-254-ZJQ.M (Sequence Method)
Last changed : 12/20/2022 9:17:06 AM by SYSTEM
(modified after loading)

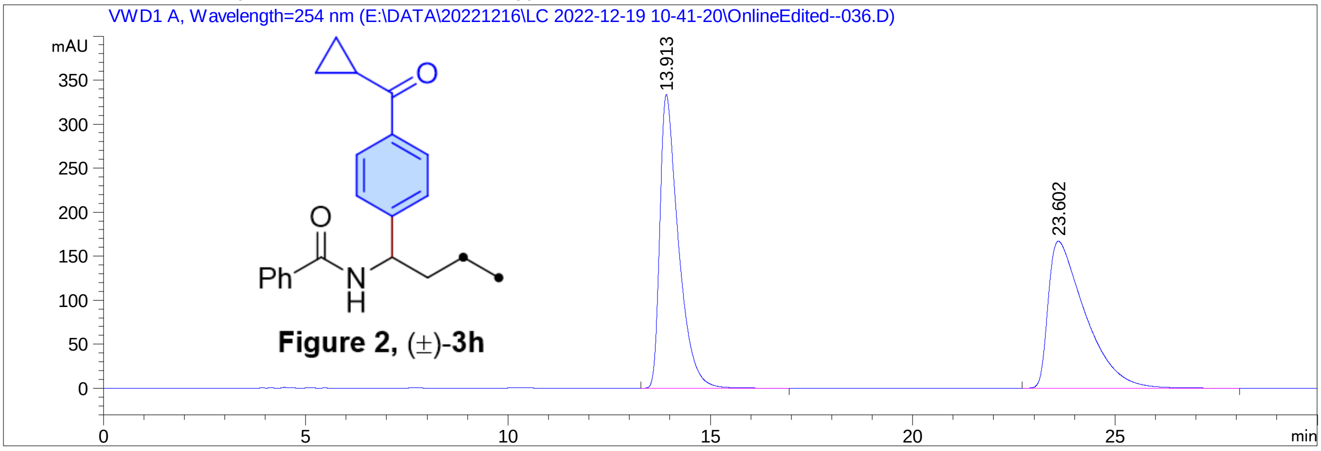

Figure 2, (±)-3h

===

Area Percent Report

===

Sorted By : Signal
Multiplier : 1.0000
Dilution : 1.0000
Do not use Multiplier & Dilution Factor with ISTDs

Signal 1: VWD1 A, Wavelength=254 nm

Peak #	RetTime [min]	Type	Width [min]	Area [mAU*s]	Height [mAU]	Area %
1	13.913	BB	0.4665	1.06296e4	333.79510	49.9669
2	23.602	BB	0.9378	1.06437e4	166.95276	50.0331

Totals : 2.12733e4 500.74786

===

*** End of Report ***

Data File E:\DATA\20221104\LC 2022-11-25 08-34-13\OnlineEdited--009.D
Sample Name: ZJQ-01-194-3

===

Acq. Operator : SYSTEM Seq. Line : 9
Acq. Instrument : HPLC1260 Location : P2-C11
Injection Date : 11/25/2022 11:31:15 AM Inj : 1
Inj Volume : 3.000 µl
Acq. Method : E:\DATA\20221104\LC 2022-11-25 08-34-13\20IPA-30-0.8-1-254-ZJQ.M
Last changed : 11/25/2022 11:25:21 AM by SYSTEM
Analysis Method : E:\DATA\20221104\LC 2022-11-25 08-34-13\20IPA-30-0.8-1-254-ZJQ.M (Sequence Method)
Last changed : 11/25/2022 2:44:58 PM by SYSTEM
(modified after loading)
Additional Info : Peak(s) manually integrated

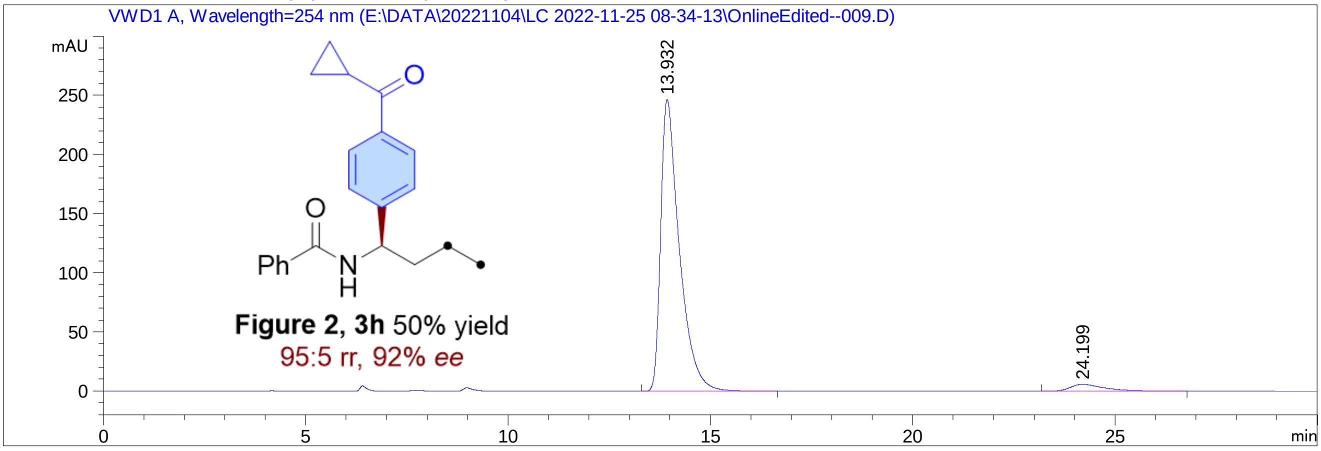

===

Area Percent Report

===

Sorted By : Signal
Multiplier : 1.0000
Dilution : 1.0000
Do not use Multiplier & Dilution Factor with ISTDs

Signal 1: VWD1 A, Wavelength=254 nm

Peak #	RetTime [min]	Type	Width [min]	Area [mAU*s]	Height [mAU]	Area %
1	13.932	BB	0.4649	7886.19092	246.71638	95.9123
2	24.199	BB	0.8270	336.10617	5.79249	4.0877

Totals : 8222.29709 252.50887

===

*** End of Report ***

Data File E:\DATA\20221017\LC 2022-11-02 13-33-52\OnlineEdited--012.D
Sample Name: ZJQ-02-26-5

===

Acq. Operator : SYSTEM Seq. Line : 12
Acq. Instrument : HPLC1260 Location : P2-A2
Injection Date : 11/2/2022 6:26:21 PM Inj : 1
Inj Volume : 3.000 µl
Different Inj Volume from Sample Entry! Actual Inj Volume : 1.000 µl
Acq. Method : E:\DATA\20221017\LC 2022-11-02 13-33-52\20IPA-20-0.8-1-ZJQ.M
Last changed : 11/2/2022 3:46:42 PM by SYSTEM
Analysis Method : E:\DATA\20221017\LC 2022-11-02 13-33-52\20IPA-20-0.8-1-ZJQ.M (Sequence Method)
Last changed : 11/21/2022 9:56:05 PM by SYSTEM
(modified after loading)
Additional Info : Peak(s) manually integrated

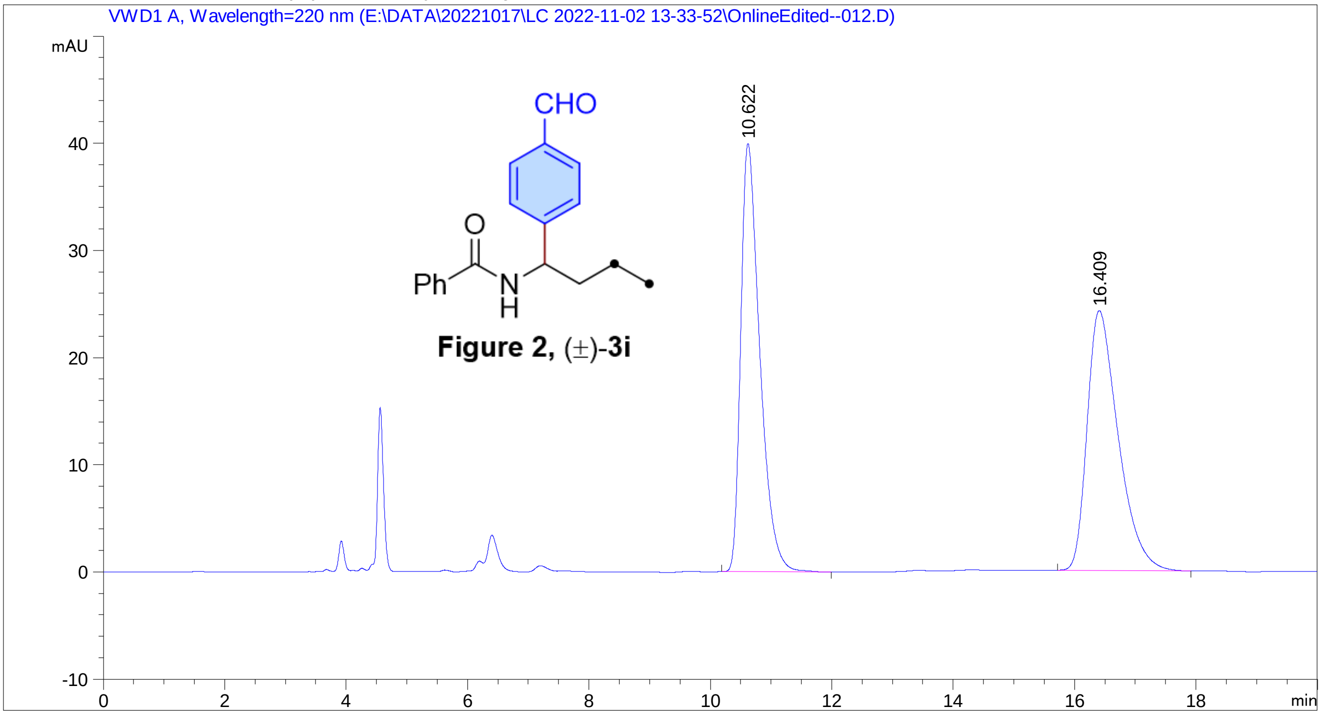

===

Area Percent Report

===

Sorted By : Signal
Multiplier : 1.0000
Dilution : 1.0000
Use Multiplier & Dilution Factor with ISTDs

Signal 1: VWD1 A, Wavelength=220 nm

Peak #	RetTime [min]	Type	Width [min]	Area [mAU*s]	Height [mAU]	Area %
1	10.622	BB	0.3284	871.31982	39.96159	50.2603
2	16.409	BB	0.5359	862.29382	24.24496	49.7397

Totals : 1733.61365 64.20655

Data File E:\DATA\20221017\LC 2022-10-19 09-38-25\OnlineEdited--009.D
Sample Name: ZJQ-01-201-1

===
Acq. Operator : SYSTEM Seq. Line : 9
Acq. Instrument : HPLC1260 Location : P2-D1
Injection Date : 10/19/2022 12:56:43 PM Inj : 1
Inj Volume : 3.000 µl
Different Inj Volume from Sample Entry! Actual Inj Volume : 1.000 µl
Acq. Method : E:\DATA\20221017\LC 2022-10-19 09-38-25\20IPA-20-0.8-1-ZJQ.M
Last changed : 10/19/2022 10:30:22 AM by SYSTEM
Analysis Method : E:\DATA\20221017\LC 2022-10-19 09-38-25\20IPA-20-0.8-1-ZJQ.M (Sequence Method)
Last changed : 11/1/2022 9:43:16 PM by SYSTEM
(modified after loading)
Additional Info : Peak(s) manually integrated

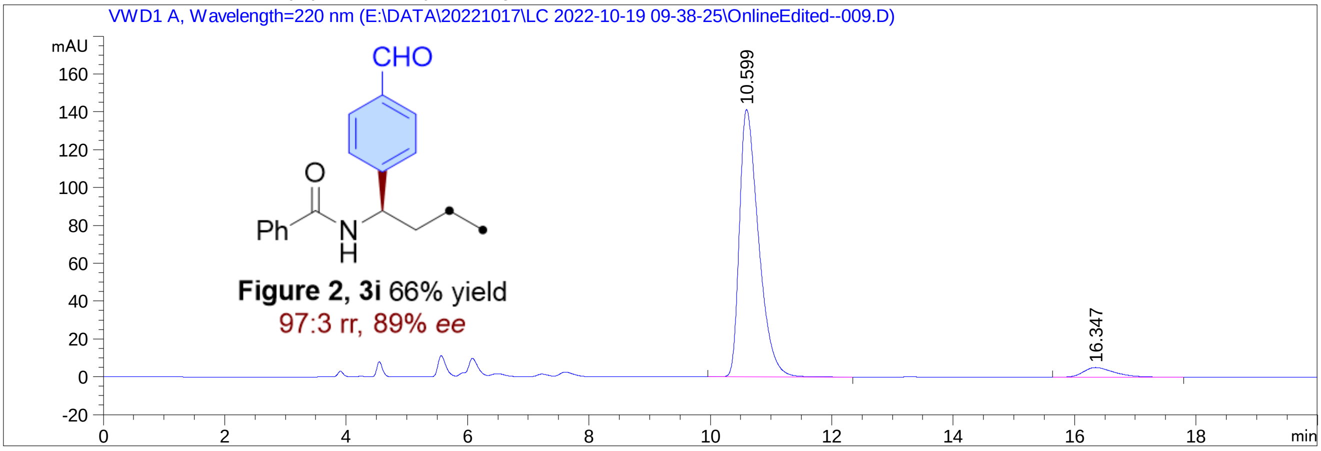

===
Area Percent Report
===

Sorted By : Signal
Multiplier : 1.0000
Dilution : 1.0000
Do not use Multiplier & Dilution Factor with ISTDs

Signal 1: VWD1 A, Wavelength=220 nm

Peak #	RetTime [min]	Type	Width [min]	Area [mAU*s]	Height [mAU]	Area %
1	10.599	BB	0.3324	3092.21436	141.27211	94.5995
2	16.347	BB	0.5178	176.52930	5.06280	5.4005

Totals : 3268.74365 146.33491

===
*** End of Report ***

Data File E:\DATA\20221017\LC 2022-10-27 14-42-40\OnlineEdited--103.D
Sample Name: ZJQ-CL

===
Acq. Operator : SYSTEM Seq. Line : 103
Acq. Instrument : HPLC1260 Location : P2-F2
Injection Date : 10/29/2022 6:47:47 AM Inj : 1
Inj Volume : 3.000 µl
Different Inj Volume from Sample Entry! Actual Inj Volume : 1.000 µl
Acq. Method : E:\DATA\20221017\LC 2022-10-27 14-42-40\20IPA-20-0.8-1-ZJQ.M
Last changed : 10/28/2022 11:34:48 PM by SYSTEM
Analysis Method : E:\DATA\20221017\LC 2022-10-27 14-42-40\20IPA-20-0.8-1-ZJQ.M (Sequence Method)
Last changed : 11/1/2022 9:58:02 PM by SYSTEM
(modified after loading)
Additional Info : Peak(s) manually integrated

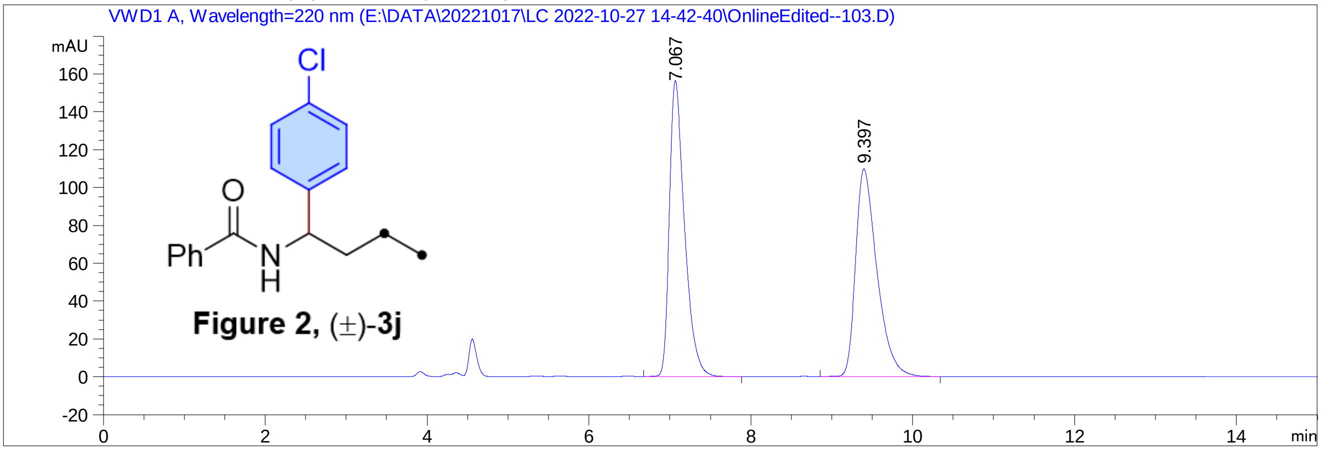

===
Area Percent Report
===

Sorted By : Signal
Multiplier : 1.0000
Dilution : 1.0000
Do not use Multiplier & Dilution Factor with ISTDs

Signal 1: VWD1 A, Wavelength=220 nm

Peak #	RetTime [min]	Type	Width [min]	Area [mAU*s]	Height [mAU]	Area %
1	7.067	BB	0.1941	2025.12427	156.38866	50.1545
2	9.397	BB	0.2758	2012.64856	109.81233	49.8455

Totals : 4037.77283 266.20099

===
*** End of Report ***

Data File E:\DATA\20221104\LC 2022-11-23 08-42-22\2EA-0201.D
Sample Name: ZJQ-02-28-3

===

Acq. Operator : SYSTEM Seq. Line : 2
Acq. Instrument : HPLC1260 Location : P2-E1
Injection Date : 11/23/2022 9:05:22 AM Inj : 1
Inj Volume : 3.000 µl
Different Inj Volume from Sample Entry! Actual Inj Volume : 1.000 µl
Acq. Method : E:\DATA\20221104\LC 2022-11-23 08-42-22\20IPA-20-0.8-1-ZJQ.M
Last changed : 11/23/2022 8:42:22 AM by SYSTEM
Analysis Method : E:\DATA\20221104\LC 2022-11-23 08-42-22\20IPA-20-0.8-1-ZJQ.M (Sequence Method)
Last changed : 11/23/2022 9:27:30 AM by SYSTEM
(modified after loading)
Additional Info : Peak(s) manually integrated

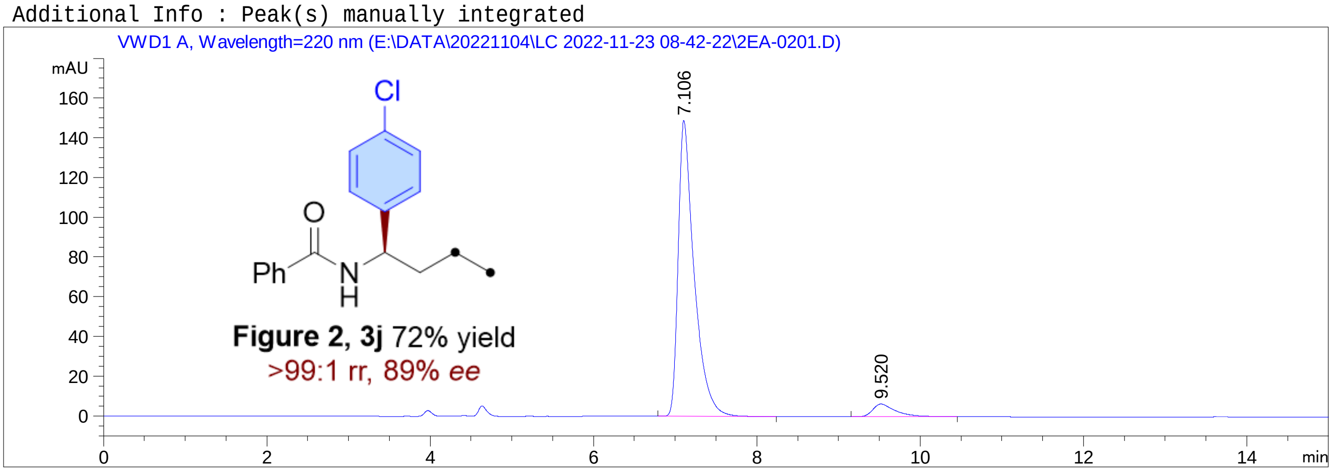

===

Area Percent Report

===

Sorted By : Signal
Multiplier : 1.0000
Dilution : 1.0000
Do not use Multiplier & Dilution Factor with ISTDs

Signal 1: VWD1 A, Wavelength=220 nm

Peak #	RetTime [min]	Type	Width [min]	Area [mAU*s]	Height [mAU]	Area %
1	7.106	BB	0.1977	2027.46448	149.00839	94.2882
2	9.520	BB	0.2795	122.81940	6.38148	5.7118

Totals : 2150.28387 155.38987

===

*** End of Report ***

Data File D:\Chem32\...-XYH 2023-05-27 08-42-35\2023-05-27ZJQ-01-201-2-RAC-OTF20IPA-15-0.D
Sample Name: ZJQ-01-201-2-RAC-OTF

===
Acq. Operator : SYSTEM Seq. Line : 10
Acq. Instrument : HPLC-1260 Location : P2-F-01
Injection Date : 5/27/2023 1:36:22 PM Inj : 1
Inj Volume : 3.000 µl
Different Inj Volume from Sample Entry! Actual Inj Volume : 8.000 µl
Acq. Method : D:\Chem32\1\Data\20230527\def_LC-XYH 2023-05-27 08-42-35\20IPA-15-0.8-1-254-ZJQ.M
Last changed : 5/27/2023 10:12:10 AM by SYSTEM
Analysis Method : D:\Chem32\1\Data\20230527\def_LC-XYH 2023-05-27 08-42-35\20IPA-15-0.8-1-254-ZJQ.M (Sequence Method)
Last changed : 5/29/2023 9:25:29 AM by SYSTEM
(modified after loading)
Additional Info : Peak(s) manually integrated

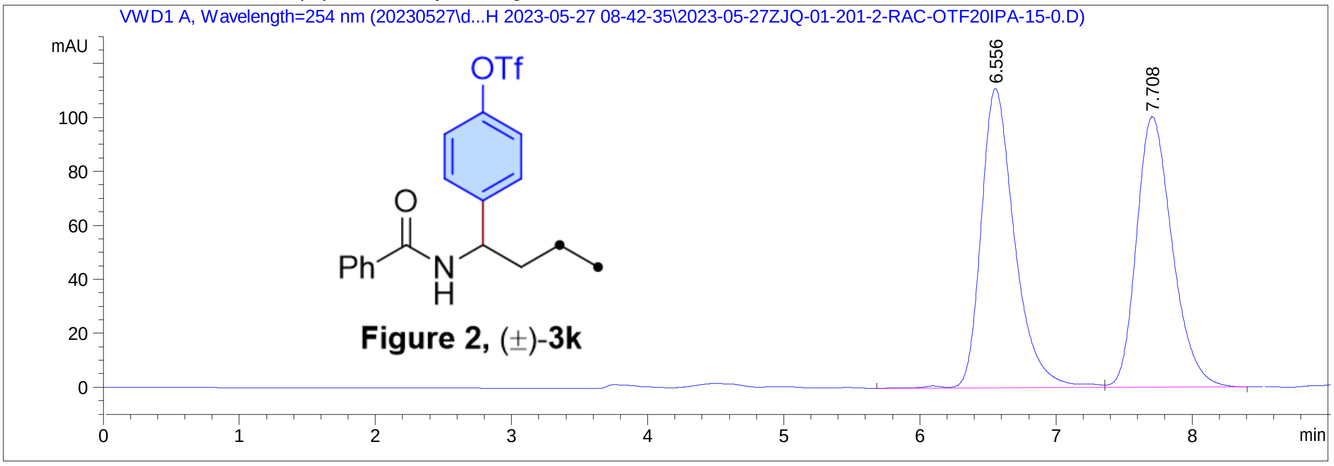

===
Area Percent Report
===

Sorted By : Signal
Multiplier : 1.0000
Dilution : 1.0000
Do not use Multiplier & Dilution Factor with ISTDs

Signal 1: VWD1 A, Wavelength=254 nm

Peak #	RetTime [min]	Type	Width [min]	Area [mAU*s]	Height [mAU]	Area %
1	6.556	BV	0.2594	1905.20496	110.92241	50.9667
2	7.708	VB	0.2812	1832.93176	100.28035	49.0333

Totals : 3738.13672 211.20276

===
*** End of Report ***

Data File D:\Chem32\...-XYH 2023-05-27 08-42-35\2023-05-27ZJQ-01-201-220IPA-15-0.8-1-254.D
Sample Name: ZJQ-01-201-2

===

```
Acq. Operator   : SYSTEM                        Seq. Line :  11
Acq. Instrument : HPLC-1260                      Location :   P2-F-02
Injection Date  : 5/27/2023 1:52:09 PM                Inj :   1
                                               Inj Volume : 3.000 µl
Different Inj Volume from Sample Entry!  Actual Inj Volume : 8.000 µl
Acq. Method     : D:\Chem32\1\Data\20230527\def_LC-XYH 2023-05-27 08-42-35\20IPA-15-0.8-1-254
                  -ZJQ.M
Last changed    : 5/27/2023 10:12:10 AM by SYSTEM
Analysis Method : D:\Chem32\1\Data\20230527\def_LC-XYH 2023-05-27 08-42-35\20IPA-15-0.8-1-254
                  -ZJQ.M (Sequence Method)
Last changed    : 5/29/2023 9:26:46 AM by SYSTEM
                  (modified after loading)
Additional Info : Peak(s) manually integrated
```

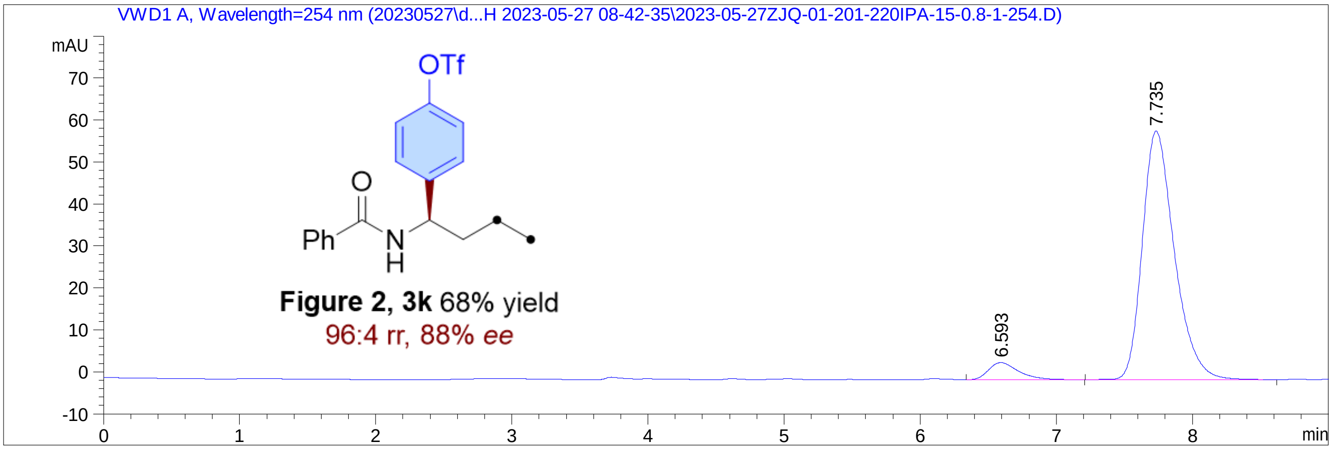

===

Area Percent Report

===

```
Sorted By             :      Signal
Multiplier            :      1.0000
Dilution              :      1.0000
Do not use Multiplier & Dilution Factor with ISTDs
```

Signal 1: VWD1 A, Wavelength=254 nm

Peak #	RetTime [min]	Type	Width [min]	Area [mAU*s]	Height [mAU]	Area %
1	6.593	BB	0.2312	62.27728	4.07813	6.0364
2	7.735	BB	0.2490	969.42419	59.21591	93.9636

Totals : 1031.70147 63.29405

===

*** End of Report ***

Data File D:\Chem32\...-XYH 2023-05-25 21-06-24\2023-05-25ZJQ-01-201-4-RAC30IPA-20-0.8-2.D
Sample Name: ZJQ-01-201-4-RAC

```
=====================================================================
Acq. Operator   : SYSTEM                        Seq. Line :    3
Acq. Instrument : HPLC-1260                      Location :    P2-F-02
Injection Date  : 5/25/2023 9:50:09 PM                Inj :    1
                                               Inj Volume : 3.000 µl
Different Inj Volume from Sample Entry!  Actual Inj Volume : 20.000 µl
Acq. Method     : D:\Chem32\1\Data\Demo\def_LC-XYH 2023-05-25 21-06-24\30IPA-20-0.8-2-254-ZJQ
                  .M
Last changed    : 5/25/2023 9:56:54 PM by SYSTEM
                  (modified after loading)
Analysis Method : D:\Chem32\1\Data\Demo\def_LC-XYH 2023-05-25 21-06-24\30IPA-20-0.8-2-254-ZJQ
                  .M (Sequence Method)
Last changed    : 5/26/2023 8:44:52 AM by SYSTEM
                  (modified after loading)
Additional Info : Peak(s) manually integrated
```

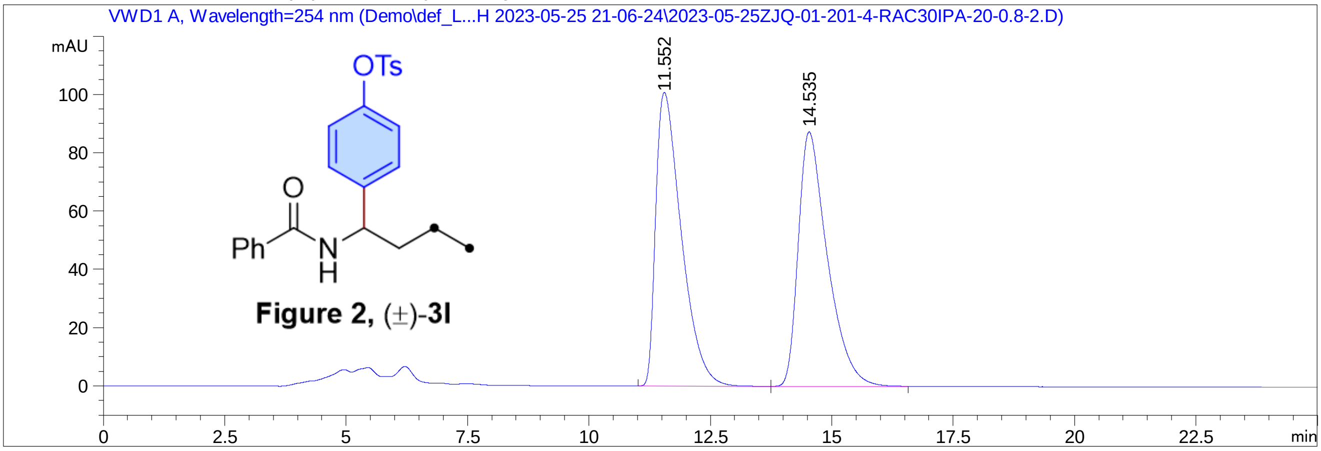


```
=====================================================================
                         Area Percent Report
=====================================================================

Sorted By             :      Signal
Multiplier            :      1.0000
Dilution              :      1.0000
Do not use Multiplier & Dilution Factor with ISTDs


Signal 1: VWD1 A, Wavelength=254 nm

Peak RetTime Type  Width     Area      Height     Area
  #   [min]        [min]   [mAU*s]     [mAU]        %
----|-------|----|-------|----------|----------|--------|
   1  11.552 BB    0.5563 3691.05151  100.77092   50.3581
   2  14.535 BB    0.6163 3638.55908   87.29358   49.6419

Totals :                  7329.61060  188.06450


=====================================================================
                          *** End of Report ***
```

Data File D:\Chem32\...-XYH 2023-05-25 21-06-24\2023-05-25ZJQ-01-201-430IPA-20-0.8-2-251.D
Sample Name: ZJQ-01-201-4

===

Acq. Operator : SYSTEM Seq. Line : 4
Acq. Instrument : HPLC-1260 Location : P2-F-01
Injection Date : 5/25/2023 10:16:01 PM Inj : 1
Inj Volume : 3.000 µl
Different Inj Volume from Sample Entry! Actual Inj Volume : 10.000 µl
Acq. Method : D:\Chem32\1\Data\Demo\def_LC-XYH 2023-05-25 21-06-24\30IPA-20-0.8-2-254-ZJQ.M
Last changed : 5/25/2023 9:56:54 PM by SYSTEM
Analysis Method : D:\Chem32\1\Data\Demo\def_LC-XYH 2023-05-25 21-06-24\30IPA-20-0.8-2-254-ZJQ.M (Sequence Method)
Last changed : 5/26/2023 8:46:44 AM by SYSTEM
(modified after loading)

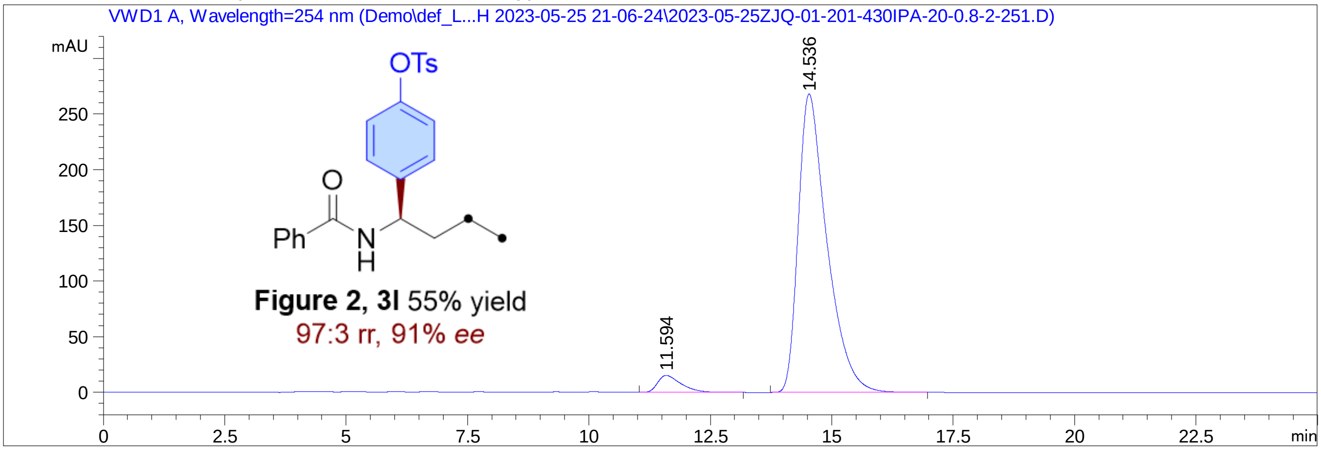

===

Area Percent Report

===

Sorted By : Signal
Multiplier : 1.0000
Dilution : 1.0000
Do not use Multiplier & Dilution Factor with ISTDs

Signal 1: VWD1 A, Wavelength=254 nm

Peak #	RetTime [min]	Type	Width [min]	Area [mAU*s]	Height [mAU]	Area %
1	11.594	BB	0.5013	513.95142	15.28469	4.4572
2	14.536	BB	0.6062	1.10169e4	268.20691	95.5428

Totals : 1.15308e4 283.49160

===

*** End of Report ***

Data File E:\DATA\20221017\LC 2022-10-29 15-18-16\OnlineEdited--030.D
Sample Name: ZJQ-02-26-3

===

Acq. Operator : SYSTEM Seq. Line : 30
Acq. Instrument : HPLC1260 Location : P2-F9
Injection Date : 10/30/2022 3:47:21 AM Inj : 1
Inj Volume : 3.000 µl
Different Inj Volume from Sample Entry! Actual Inj Volume : 1.000 µl
Acq. Method : E:\DATA\20221017\LC 2022-10-29 15-18-16\20IPA-20-0.8-1-ZJQ.M
Last changed : 10/29/2022 10:05:54 PM by SYSTEM
Analysis Method : E:\DATA\20221017\LC 2022-10-29 15-18-16\20IPA-20-0.8-1-ZJQ.M (Sequence Method)
Last changed : 10/30/2022 9:59:25 AM by SYSTEM
(modified after loading)
Additional Info : Peak(s) manually integrated

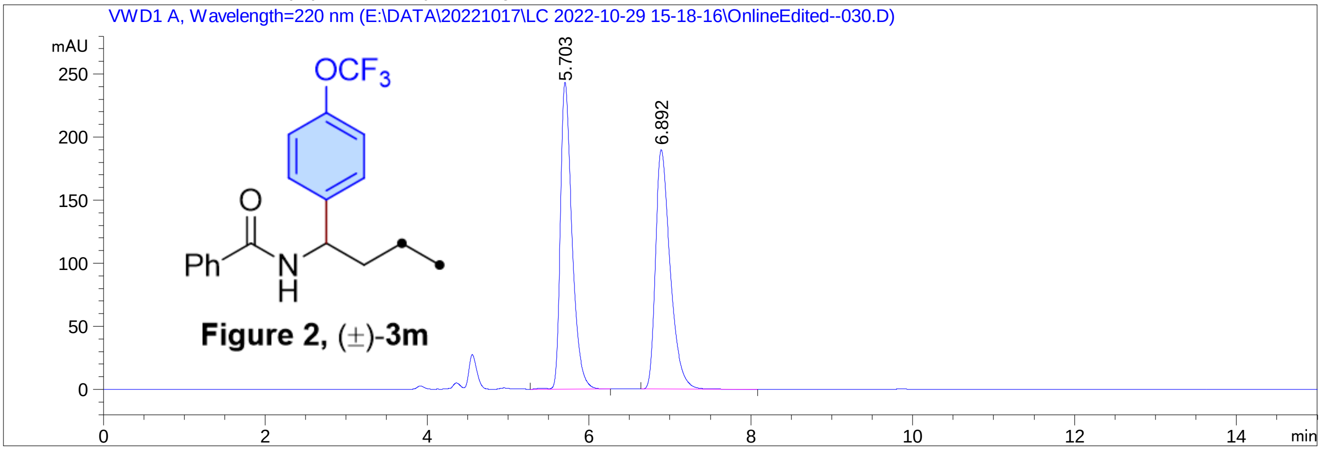

===

Area Percent Report

===

Sorted By : Signal
Multiplier : 1.0000
Dilution : 1.0000
Do not use Multiplier & Dilution Factor with ISTDs

Signal 1: VWD1 A, Wavelength=220 nm

Peak #	RetTime [min]	Type	Width [min]	Area [mAU*s]	Height [mAU]	Area %
1	5.703	VB R	0.1496	2413.22876	243.46538	50.2114
2	6.892	BB	0.1890	2392.90747	189.86459	49.7886

Totals : 4806.13623 433.32997

===

*** End of Report ***

Data File E:\DATA\20221017\LC 2022-10-17 17-57-01\2EA-0201.D
Sample Name: ZJQ-01-194-2

===

Acq. Operator : SYSTEM   Seq. Line : 2
Acq. Instrument : HPLC1260   Location : P2-E1
Injection Date : 10/17/2022 6:19:32 PM   Inj : 1
Inj Volume : 3.000 µl
Different Inj Volume from Sample Entry! Actual Inj Volume : 0.100 µl
Acq. Method : E:\DATA\20221017\LC 2022-10-17 17-57-01\20IPA_20_0.8_1-220-ZJQ.M
Last changed : 10/17/2022 5:57:01 PM by SYSTEM
Analysis Method : E:\DATA\20221017\LC 2022-10-17 17-57-01\20IPA_20_0.8_1-220-ZJQ.M (Sequence Method)
Last changed : 10/31/2022 11:08:07 AM by SYSTEM
(modified after loading)
Additional Info : Peak(s) manually integrated

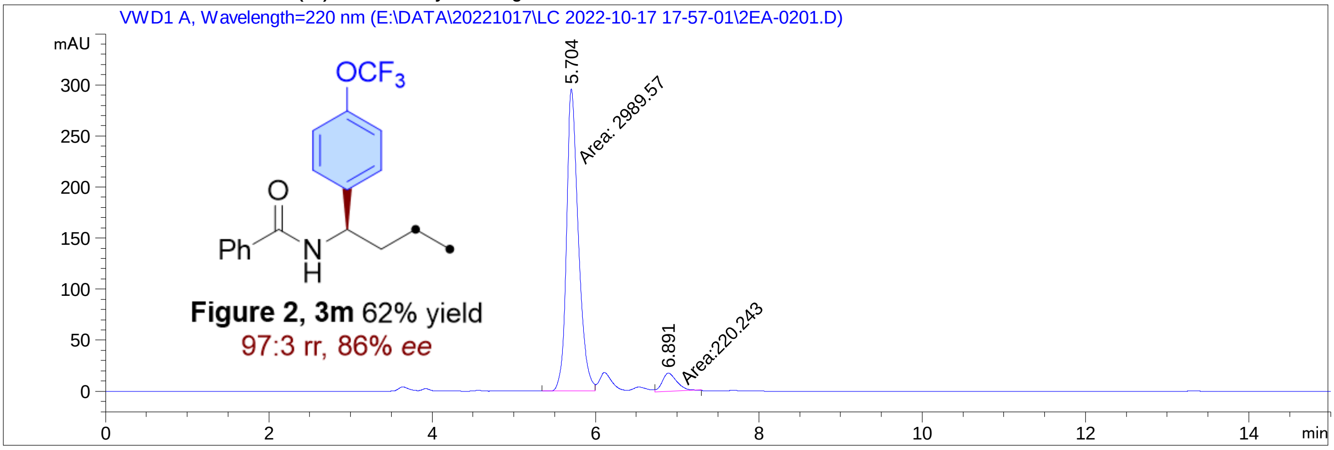

===

Area Percent Report

===

Sorted By : Signal
Multiplier : 1.0000
Dilution : 1.0000
Do not use Multiplier & Dilution Factor with ISTDs

Signal 1: VWD1 A, Wavelength=220 nm

Peak #	RetTime [min]	Type	Width [min]	Area [mAU*s]	Height [mAU]	Area %
1	5.704	MF	0.1685	2989.56567	295.71387	93.1384
2	6.891	FM	0.2055	220.24323	17.86212	6.8616

Totals : 3209.80890 313.57598

===

*** End of Report ***

Data File E:\DATA\20221104\LC 2022-12-01 15-16-07\OnlineEdited--008.D
Sample Name: ZJQ-02-66-13

===
Acq. Operator   : SYSTEM   Seq. Line :   8
Acq. Instrument : HPLC1260   Location :   P2-C10
Injection Date : 12/1/2022 6:11:09 PM   Inj :   1
                                                 Inj Volume : 3.000 µl
Different Inj Volume from Sample Entry! Actual Inj Volume : 5.000 µl
Acq. Method   : E:\DATA\20221104\LC 2022-12-01 15-16-07\20IPA-20-0.8-1-254-ZJQ.M
Last changed   : 12/1/2022 4:02:07 PM by SYSTEM
Analysis Method : E:\DATA\20221104\LC 2022-12-01 15-16-07\20IPA-20-0.8-1-254-ZJQ.M (Sequence Method)
Last changed   : 12/1/2022 7:38:07 PM by SYSTEM
(modified after loading)
Additional Info : Peak(s) manually integrated

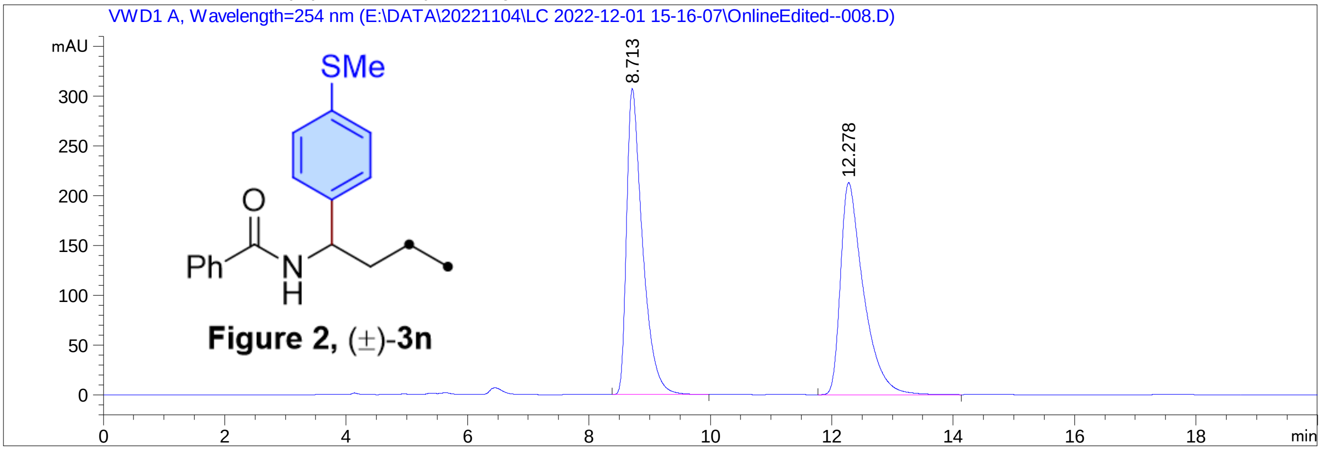

===
Area Percent Report
===

Sorted By : Signal
Multiplier : 1.0000
Dilution : 1.0000
Do not use Multiplier & Dilution Factor with ISTDs

Signal 1: VWD1 A, Wavelength=254 nm

Peak #	RetTime [min]	Type	Width [min]	Area [mAU*s]	Height [mAU]	Area %
1	8.713	BB	0.2678	5602.88330	307.13885	49.9369
2	12.278	BB	0.3849	5617.03711	212.85367	50.0631

Totals : 1.12199e4   519.99252

===
*** End of Report ***

Data File E:\DATA\20221104\LC 2022-11-29 15-10-00\OnlineEdited--058.D
Sample Name: ZJQ-02-66-12

===

Acq. Operator : SYSTEM Seq. Line : 58
Acq. Instrument : HPLC1260 Location : P2-C11
Injection Date : 11/30/2022 2:26:26 PM Inj : 1
Inj Volume : 3.000 µl
Different Inj Volume from Sample Entry! Actual Inj Volume : 1.000 µl
Acq. Method : E:\DATA\20221104\LC 2022-11-29 15-10-00\20IPA-20-0.8-1-254-ZJQ.M
Last changed : 11/30/2022 11:18:38 AM by SYSTEM
Analysis Method : E:\DATA\20221104\LC 2022-11-29 15-10-00\20IPA-20-0.8-1-254-ZJQ.M (Sequence Method)
Last changed : 12/1/2022 8:52:59 AM by SYSTEM
(modified after loading)
Additional Info : Peak(s) manually integrated

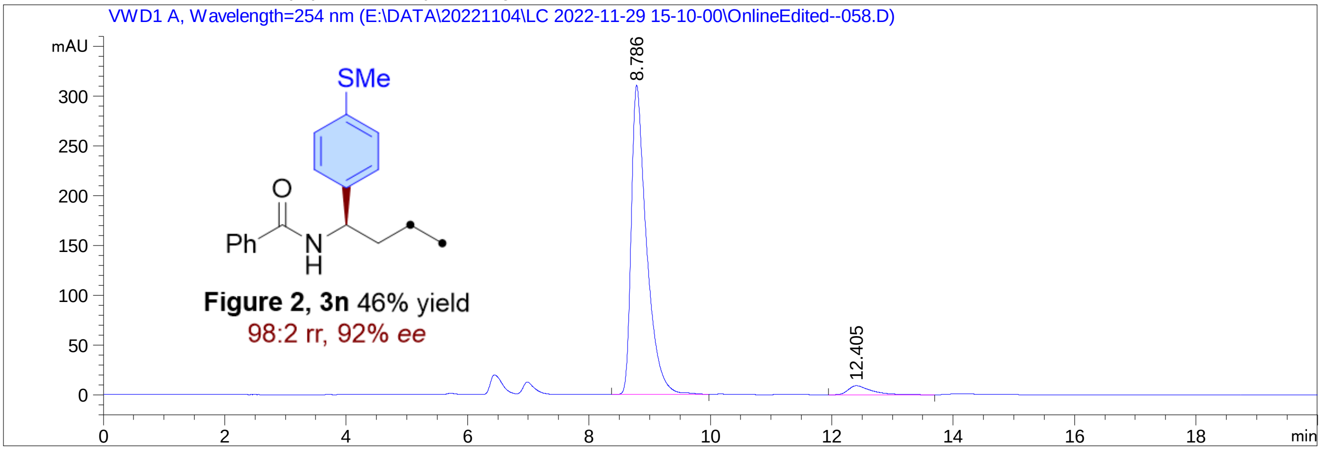

===

Area Percent Report

===

Sorted By : Signal
Multiplier : 1.0000
Dilution : 1.0000
Do not use Multiplier & Dilution Factor with ISTDs

Signal 1: VWD1 A, Wavelength=254 nm

Peak #	RetTime [min]	Type	Width [min]	Area [mAU*s]	Height [mAU]	Area %
1	8.786	BB	0.2537	5421.26758	310.50696	95.8650
2	12.405	BB	0.3745	233.84073	9.04996	4.1350

Totals : 5655.10831 319.55692

===

*** End of Report ***

Data File D:\Chem32\...-XYH 2023-06-15 21-25-27\2023-06-15ZJQ-03-47-3-1-OD20IPA-20-0.8-1.D
Sample Name: ZJQ-03-47-3-1-OD

===

Acq. Operator : SYSTEM Seq. Line : 2
Acq. Instrument : HPLC-1260 Location : P2-F-04
Injection Date : 6/15/2023 9:48:20 PM Inj : 1
Inj Volume : 3.000 µl
Different Inj Volume from Sample Entry! Actual Inj Volume : 5.000 µl
Acq. Method : D:\Chem32\1\Data\20230601\def_LC-XYH 2023-06-15 21-25-27\20IPA-20-0.8-1-254 -ZJQ.M
Last changed : 5/25/2023 10:22:57 PM by SYSTEM
Analysis Method : D:\Chem32\1\Data\20230601\def_LC-XYH 2023-06-15 21-25-27\20IPA-20-0.8-1-254 -ZJQ.M (Sequence Method)
Last changed : 6/22/2023 11:17:01 AM by SYSTEM
(modified after loading)
Additional Info : Peak(s) manually integrated

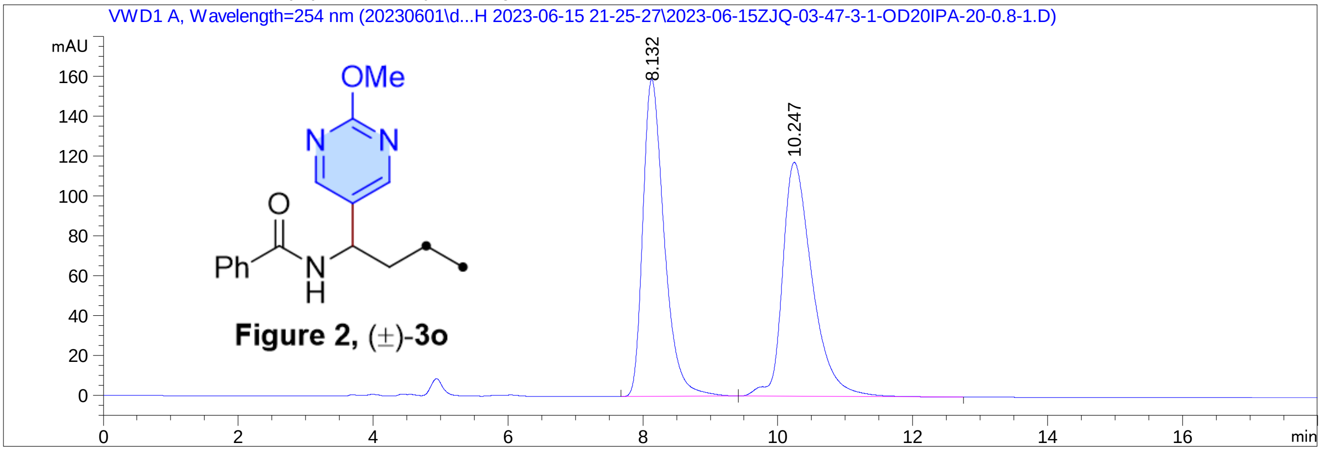

===

Area Percent Report

===

Sorted By : Signal
Multiplier : 1.0000
Dilution : 1.0000
Do not use Multiplier & Dilution Factor with ISTDs

Signal 1: VWD1 A, Wavelength=254 nm

Peak #	RetTime [min]	Type	Width [min]	Area [mAU*s]	Height [mAU]	Area %
1	8.132	BB	0.3350	3504.41968	159.08466	49.3666
2	10.247	VB R	0.4624	3594.35059	117.32225	50.6334

Totals : 7098.77026 276.40691

===

*** End of Report ***

Data File D:\Chem32\...-XYH 2023-06-14 17-21-14\2023-06-15ZJQ-03-46-3-OD20IPA-20-0.8-1-2.D
Sample Name: ZJQ-03-46-3-OD

===

Acq. Operator : SYSTEM Seq. Line : 17
Acq. Instrument : HPLC-1260 Location : P2-F-04
Injection Date : 6/15/2023 12:04:42 AM Inj : 1
Inj Volume : 3.000 µl
Acq. Method : D:\Chem32\1\Data\20230601\def_LC-XYH 2023-06-14 17-21-14\20IPA-20-0.8-1-254 -ZJQ.M
Last changed : 5/25/2023 10:22:57 PM by SYSTEM
Analysis Method : D:\Chem32\1\Data\20230601\def_LC-XYH 2023-06-14 17-21-14\20IPA-20-0.8-1-254 -ZJQ.M (Sequence Method)
Last changed : 6/22/2023 11:15:42 AM by SYSTEM
(modified after loading)
Additional Info : Peak(s) manually integrated

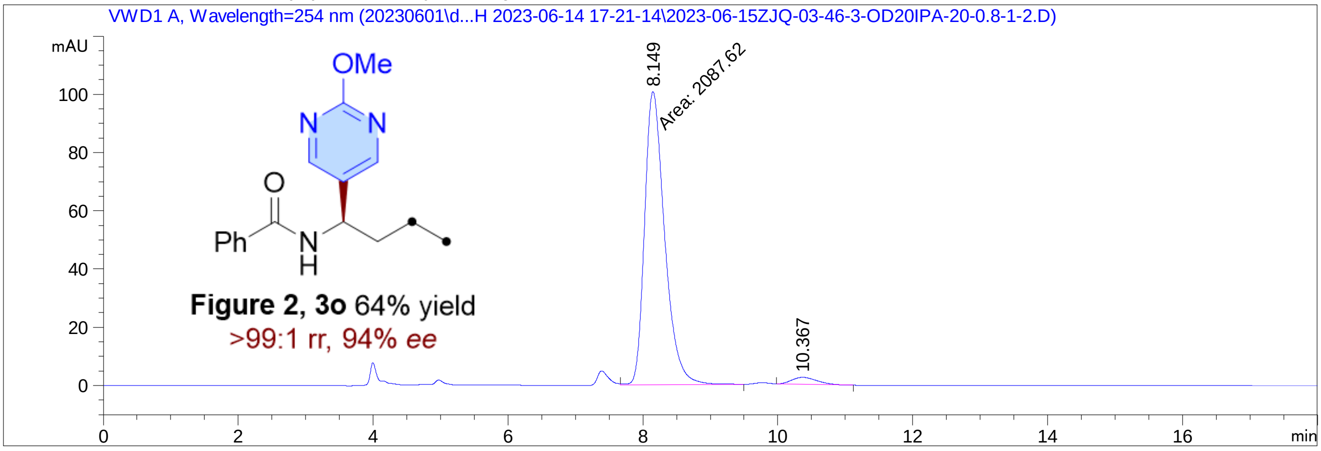

===

Area Percent Report

===

Sorted By : Signal
Multiplier : 1.0000
Dilution : 1.0000
Do not use Multiplier & Dilution Factor with ISTDs

Signal 1: VWD1 A, Wavelength=254 nm

Peak #	RetTime [min]	Type	Width [min]	Area [mAU*s]	Height [mAU]	Area %
1	8.149	FM	0.3452	2087.62427	100.79292	97.1049
2	10.367	BB	0.4030	62.24055	2.38857	2.8951

Totals : 2149.86482 103.18148

===

*** End of Report ***

Data File E:\DATA\20221104\LC 2022-12-09 10-39-59\OnlineEdited--057.D
Sample Name: ZJQ-02-72-3

===

Acq. Operator : SYSTEM Seq. Line : 57
Acq. Instrument : HPLC1260 Location : P2-E2
Injection Date : 12/10/2022 8:46:52 AM Inj : 1
Inj Volume : 3.000 µl
Different Inj Volume from Sample Entry! Actual Inj Volume : 8.000 µl
Acq. Method : E:\DATA\20221104\LC 2022-12-09 10-39-59\30IPA-20-0.8-4-220-ZJQ.M
Last changed : 12/9/2022 10:49:14 PM by SYSTEM
Analysis Method : E:\DATA\20221104\LC 2022-12-09 10-39-59\30IPA-20-0.8-4-220-ZJQ.M (Sequence Method)
Last changed : 12/19/2022 8:04:30 PM by SYSTEM
(modified after loading)
Additional Info : Peak(s) manually integrated

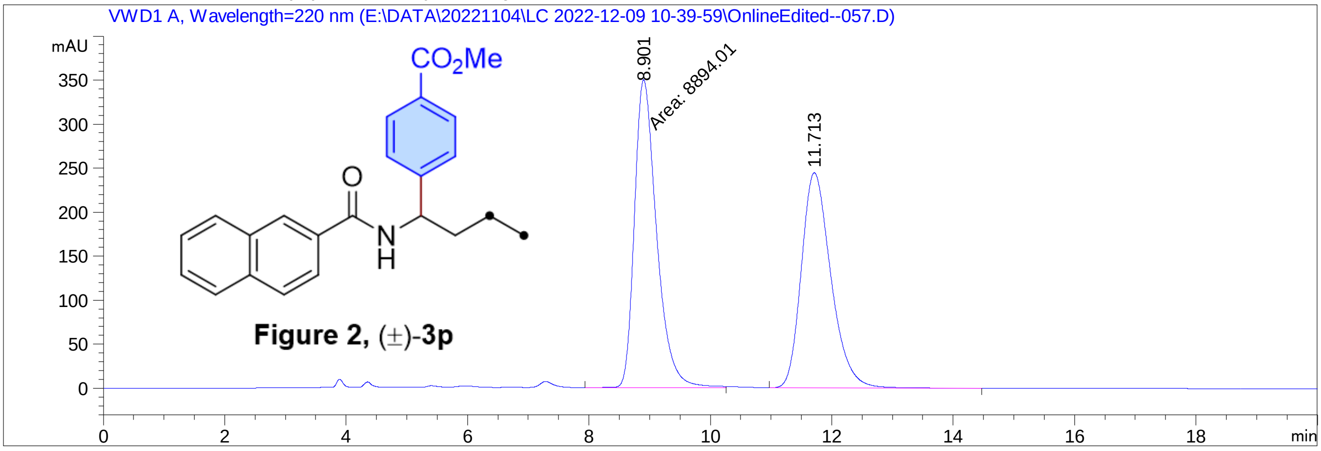

===

Area Percent Report

===

Sorted By : Signal
Multiplier : 1.0000
Dilution : 1.0000
Do not use Multiplier & Dilution Factor with ISTDs

Signal 1: VWD1 A, Wavelength=220 nm

Peak #	RetTime [min]	Type	Width [min]	Area [mAU*s]	Height [mAU]	Area %
1	8.901	MF	0.4236	8894.00684	349.91989	51.8291
2	11.713	BB	0.5185	8266.24414	244.46594	48.1709

Totals : 1.71603e4 594.38583

===

*** End of Report ***

Data File E:\DATA\20221216\LC 2022-12-19 10-41-20\OnlineEdited--034.D
Sample Name: ZJQ-02-34-1

===

Acq. Operator : SYSTEM Seq. Line : 34
Acq. Instrument : HPLC1260 Location : P2-E10
Injection Date : 12/19/2022 11:15:43 PM Inj : 1
Inj Volume : 3.000 µl
Different Inj Volume from Sample Entry! Actual Inj Volume : 1.000 µl
Acq. Method : E:\DATA\20221216\LC 2022-12-19 10-41-20\30IPA-20-0.8-2-254-ZJQ.M
Last changed : 12/19/2022 10:39:06 PM by SYSTEM
Analysis Method : E:\DATA\20221216\LC 2022-12-19 10-41-20\30IPA-20-0.8-2-254-ZJQ.M (Sequence Method)
Last changed : 12/20/2022 9:15:42 AM by SYSTEM
(modified after loading)
Additional Info : Peak(s) manually integrated

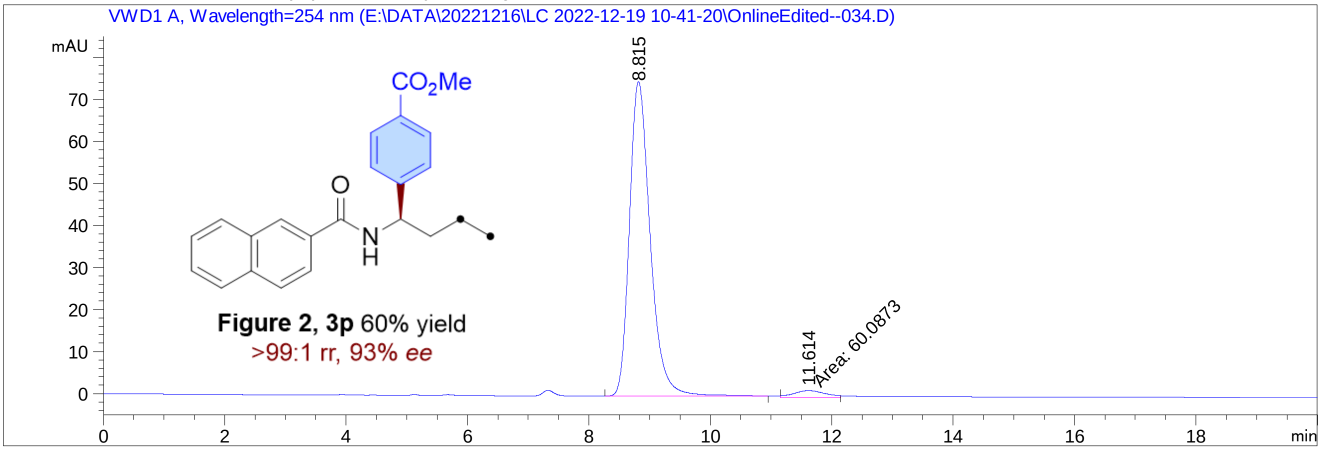

===

Area Percent Report

===

Sorted By : Signal
Multiplier : 1.0000
Dilution : 1.0000
Do not use Multiplier & Dilution Factor with ISTDs

Signal 1: VWD1 A, Wavelength=254 nm

Peak #	RetTime [min]	Type	Width [min]	Area [mAU*s]	Height [mAU]	Area %
1	8.815	BB	0.3627	1773.27295	74.76226	96.7226
2	11.614	MM	0.6087	60.08733	1.64525	3.2774

Totals : 1833.36028 76.40751

===

*** End of Report ***

Data File E:\DATA\20221104\LC 2022-11-29 11-31-33\2AJ-0201.D
Sample Name: ZJQ-02-64-2

===

Acq. Operator	: SYSTEM	Seq. Line :	2
Acq. Instrument	: HPLC1260	Location :	P2-A10
Injection Date	: 11/29/2022 11:54:23 AM	Inj :	1
		Inj Volume :	3.000 µl

Acq. Method : E:\DATA\20221104\LC 2022-11-29 11-31-33\30IPA-20-0.8-1-254-ZJQ.M
Last changed : 11/29/2022 11:31:34 AM by SYSTEM
Analysis Method : E:\DATA\20221104\LC 2022-11-29 11-31-33\30IPA-20-0.8-1-254-ZJQ.M (Sequence Method)
Last changed : 11/29/2022 3:03:36 PM by SYSTEM
(modified after loading)

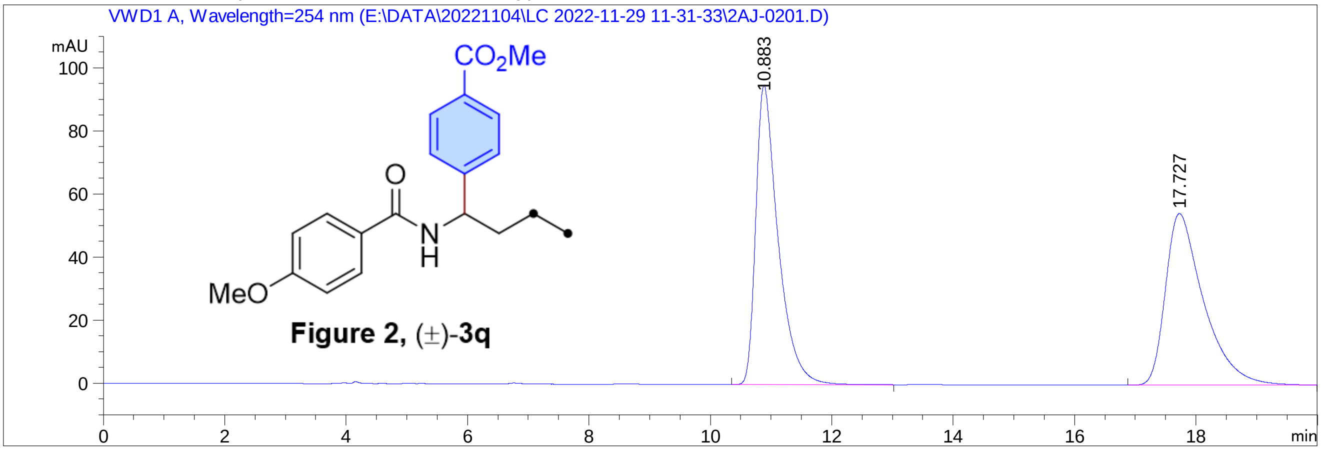

===

Area Percent Report

===

Sorted By : Signal
Multiplier : 1.0000
Dilution : 1.0000
Do not use Multiplier & Dilution Factor with ISTDs

Signal 1: VWD1 A, Wavelength=254 nm

Peak #	RetTime [min]	Type	Width [min]	Area [mAU*s]	Height [mAU]	Area %
1	10.883	BB	0.3745	2421.41675	94.95718	50.4622
2	17.727	BBA	0.6428	2377.05859	54.39446	49.5378

Totals : 4798.47534 149.35163

===

*** End of Report ***

Data File E:\DATA\20221104\LC 2022-11-25 08-34-13\OnlineEdited--047.D
Sample Name: ZJQ-02-9-2

===

Acq. Operator : SYSTEM   Seq. Line : 47
Acq. Instrument : HPLC1260   Location : P2-A1
Injection Date : 11/26/2022 3:46:52 AM   Inj : 1
Inj Volume : 3.000 µl
Different Inj Volume from Sample Entry! Actual Inj Volume : 1.000 µl
Acq. Method : E:\DATA\20221104\LC 2022-11-25 08-34-13\30IPA-30-0.8-1-254-ZJQ.M
Last changed : 11/25/2022 10:00:05 AM by SYSTEM
Analysis Method : E:\DATA\20221104\LC 2022-11-25 08-34-13\30IPA-30-0.8-1-254-ZJQ.M (Sequence Method)
Last changed : 11/26/2022 10:16:14 AM by SYSTEM
(modified after loading)
Additional Info : Peak(s) manually integrated

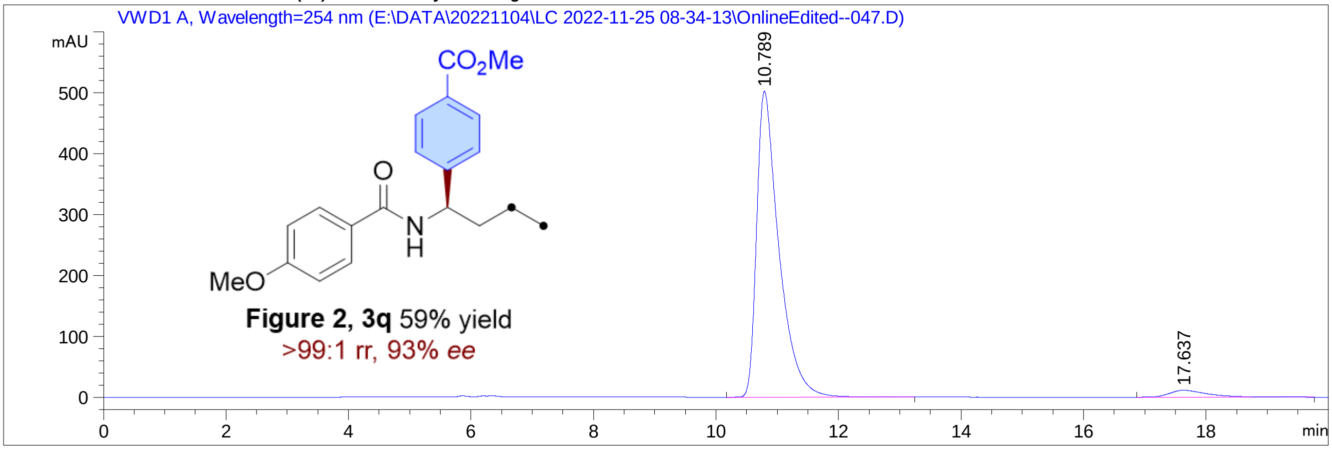

===

Area Percent Report

===

Sorted By : Signal
Multiplier : 1.0000
Dilution : 1.0000
Do not use Multiplier & Dilution Factor with ISTDs

Signal 1: VWD1 A, Wavelength=254 nm

Peak #	RetTime [min]	Type	Width [min]	Area [mAU*s]	Height [mAU]	Area %
1	10.789	BB	0.3686	1.26761e4	502.06302	96.3166
2	17.637	BB	0.6315	484.77106	11.27660	3.6834

Totals : 1.31609e4 513.33962

===

*** End of Report ***

Data File E:\DATA\20221104\LC 2022-11-27 11-08-17\2BA-0201.D
Sample Name: ZJQ-02-44-2

===

Acq. Operator : SYSTEM Seq. Line : 2
Acq. Instrument : HPLC1260 Location : P2-B1
Injection Date : 11/27/2022 11:30:51 AM Inj : 1
Inj Volume : 3.000 µl
Different Inj Volume from Sample Entry! Actual Inj Volume : 5.000 µl
Acq. Method : E:\DATA\20221104\LC 2022-11-27 11-08-17\20IPA-20-0.8-2-254-ZJQ.M
Last changed : 11/27/2022 11:08:17 AM by SYSTEM
Analysis Method : E:\DATA\20221104\LC 2022-11-27 11-08-17\20IPA-20-0.8-2-254-ZJQ.M (Sequence Method)
Last changed : 11/27/2022 12:17:32 PM by SYSTEM
(modified after loading)
Additional Info : Peak(s) manually integrated

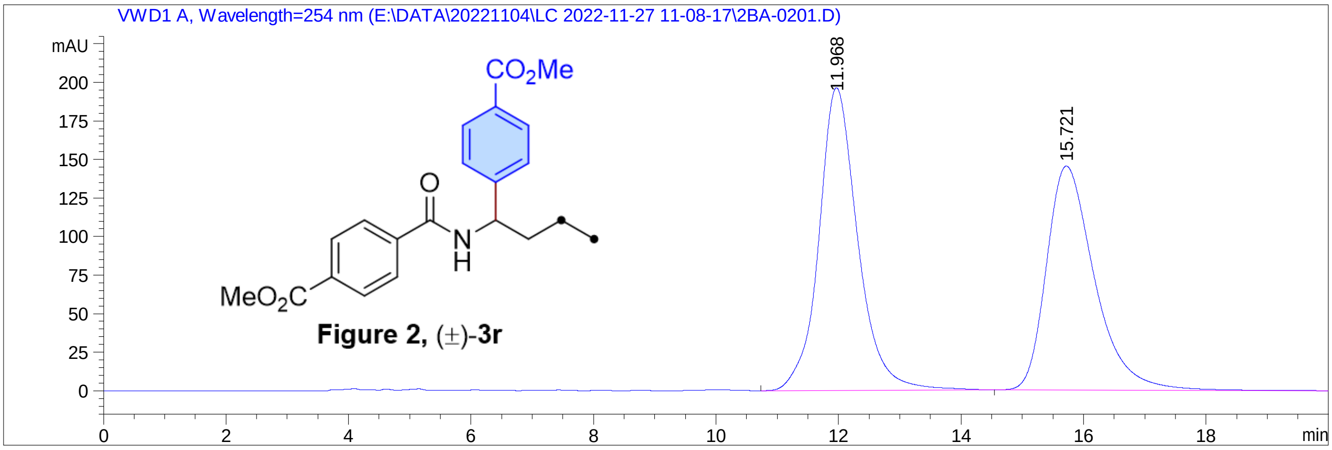

===

Area Percent Report

===

Sorted By : Signal
Multiplier : 1.0000
Dilution : 1.0000
Do not use Multiplier & Dilution Factor with ISTDs

Signal 1: VWD1 A, Wavelength=254 nm

Peak #	RetTime [min]	Type	Width [min]	Area [mAU*s]	Height [mAU]	Area %
1	11.968	BB	0.6627	8676.56836	196.30040	52.2755
2	15.721	BBA	0.8294	7921.19531	145.20605	47.7245

Totals : 1.65978e4 341.50645

===

*** End of Report ***

Data File E:\DATA\20221104\LC 2022-11-25 08-34-13\OnlineEdited--017.D
Sample Name: ZJQ-02-44-1

===
Acq. Operator : SYSTEM Seq. Line : 17
Acq. Instrument : HPLC1260 Location : P2-E6
Injection Date : 11/25/2022 2:35:11 PM Inj : 1
Inj Volume : 3.000 µl
Different Inj Volume from Sample Entry! Actual Inj Volume : 2.000 µl
Acq. Method : E:\DATA\20221104\LC 2022-11-25 08-34-13\20IPA-20-0.8-2-254-ZJQ.M
Last changed : 11/25/2022 10:40:10 AM by SYSTEM
Analysis Method : E:\DATA\20221104\LC 2022-11-25 08-34-13\20IPA-20-0.8-2-254-ZJQ.M (Sequence Method)
Last changed : 11/25/2022 3:00:15 PM by SYSTEM
(modified after loading)
Additional Info : Peak(s) manually integrated

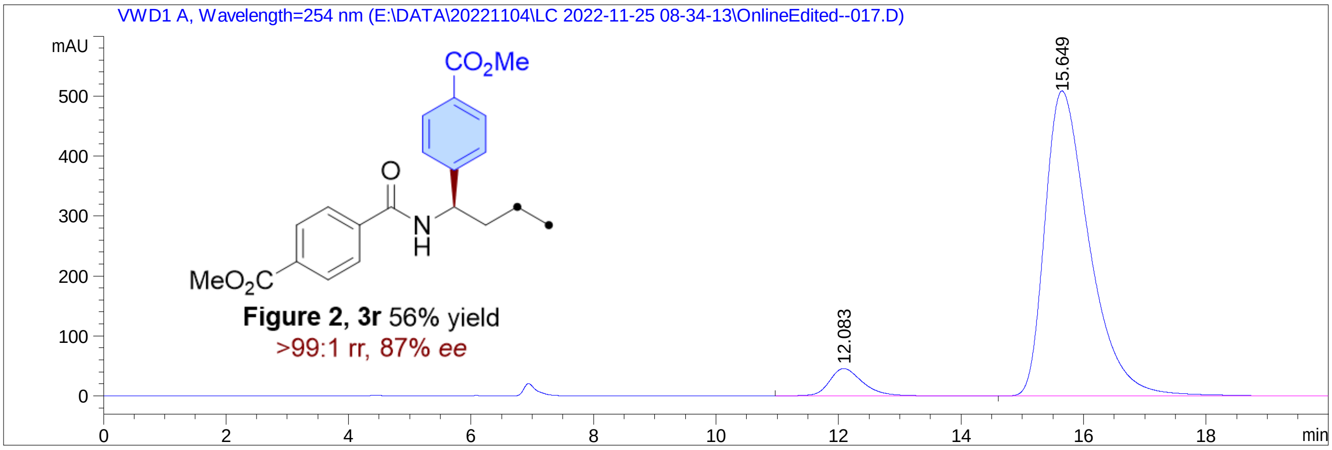

===
Area Percent Report
===

Sorted By : Signal
Multiplier : 1.0000
Dilution : 1.0000
Do not use Multiplier & Dilution Factor with ISTDs

Signal 1: VWD1 A, Wavelength=254 nm

Peak #	RetTime [min]	Type	Width [min]	Area [mAU*s]	Height [mAU]	Area %
1	12.083	MM	0.5776	1752.48926	45.88535	6.4124
2	15.649	MM	0.7656	2.55771e4	508.73096	93.5876

Totals : 2.73295e4 554.61631

===
*** End of Report ***

Data File E:\DATA\20221017\LC 2022-11-01 16-27-07\OnlineEdited--013.D
Sample Name: ZJQ-02-7-12

===
Acq. Operator   : SYSTEM   Seq. Line : 13
Acq. Instrument : HPLC1260   Location :   P2-A2
Injection Date : 11/1/2022 9:26:30 PM   Inj :   1
Inj Volume : 3.000 µl
Different Inj Volume from Sample Entry! Actual Inj Volume : 1.000 µl
Acq. Method   : E:\DATA\20221017\LC 2022-11-01 16-27-07\10IPA-20-0.8-1-220-ZJQ.M
Last changed   : 11/1/2022 9:06:52 PM by SYSTEM
Analysis Method : E:\DATA\20221017\LC 2022-11-01 16-27-07\10IPA-20-0.8-1-220-ZJQ.M (Sequence Method)
Last changed   : 11/24/2022 9:18:30 PM by SYSTEM
(modified after loading)
Additional Info : Peak(s) manually integrated

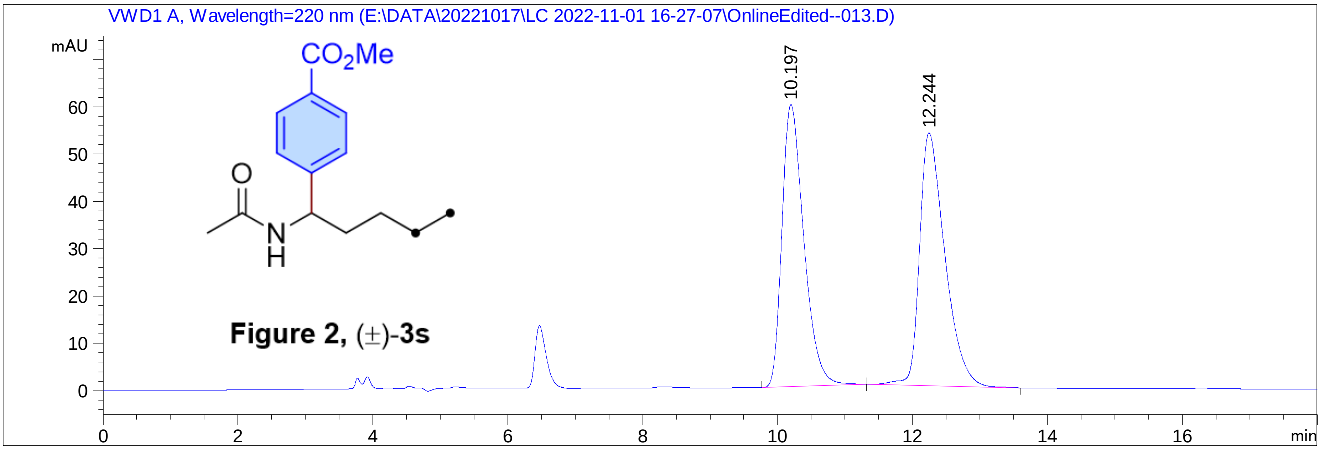

===
Area Percent Report
===

Sorted By : Signal
Multiplier : 1.0000
Dilution : 1.0000
Do not use Multiplier & Dilution Factor with ISTDs

Signal 1: VWD1 A, Wavelength=220 nm

Peak #	RetTime [min]	Type	Width [min]	Area [mAU*s]	Height [mAU]	Area %
1	10.197	BB	0.3466	1347.23120	59.63608	49.1409
2	12.244	BB	0.3935	1394.33728	53.42418	50.8591

Totals : 2741.56848 113.06026

===
*** End of Report ***

Data File E:\DATA\20221104\LC 2022-11-25 08-34-13\OnlineEdited--013.D
Sample Name: ZJQ-02-12-5

===

Acq. Operator : SYSTEM Seq. Line : 13
Acq. Instrument : HPLC1260 Location : P2-D10
Injection Date : 11/25/2022 1:15:02 PM Inj : 1
Inj Volume : 3.000 µl
Acq. Method : E:\DATA\20221104\LC 2022-11-25 08-34-13\10IPA-20-0.8-1-254-ZJQ.M
Last changed : 11/25/2022 10:37:34 AM by SYSTEM
Analysis Method : E:\DATA\20221104\LC 2022-11-25 08-34-13\10IPA-20-0.8-1-254-ZJQ.M (Sequence Method)
Last changed : 11/25/2022 2:48:28 PM by SYSTEM
(modified after loading)

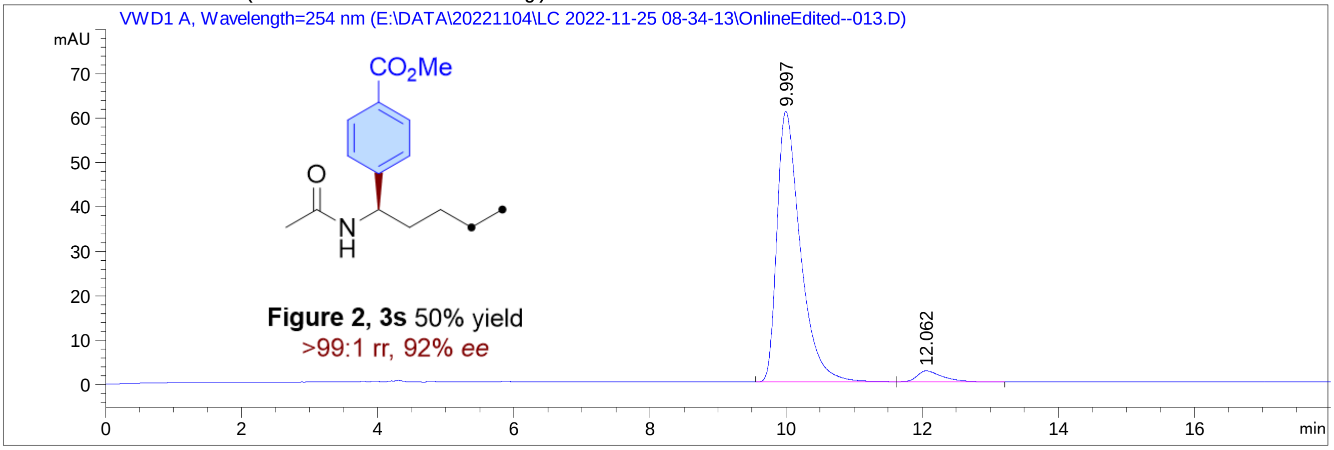

===

Area Percent Report

===

Sorted By : Signal
Multiplier : 1.0000
Dilution : 1.0000
Do not use Multiplier & Dilution Factor with ISTDs

Signal 1: VWD1 A, Wavelength=254 nm

Peak #	RetTime [min]	Type	Width [min]	Area [mAU*s]	Height [mAU]	Area %
1	9.997	BB	0.3588	1454.21777	60.85099	95.9193
2	12.062	BB	0.3655	61.86659	2.44284	4.0807

Totals : 1516.08437 63.29383

===

*** End of Report ***

Data File E:\DATA\20221104\LC 2022-11-20 17-43-23\OnlineEdited--004.D
Sample Name: ZJQ-02-51-4

===

Acq. Operator : SYSTEM Seq. Line : 4
Acq. Instrument : HPLC1260 Location : P2-E10
Injection Date : 11/20/2022 6:47:48 PM Inj : 1
Inj Volume : 3.000 µl
Different Inj Volume from Sample Entry! Actual Inj Volume : 1.000 µl
Acq. Method : E:\DATA\20221104\LC 2022-11-20 17-43-23\20IPA-20-0.8-1-ZJQ.M
Last changed : 11/20/2022 5:43:23 PM by SYSTEM
Analysis Method : E:\DATA\20221104\LC 2022-11-20 17-43-23\20IPA-20-0.8-1-ZJQ.M (Sequence Method)
Last changed : 11/21/2022 9:03:43 PM by SYSTEM
(modified after loading)
Additional Info : Peak(s) manually integrated

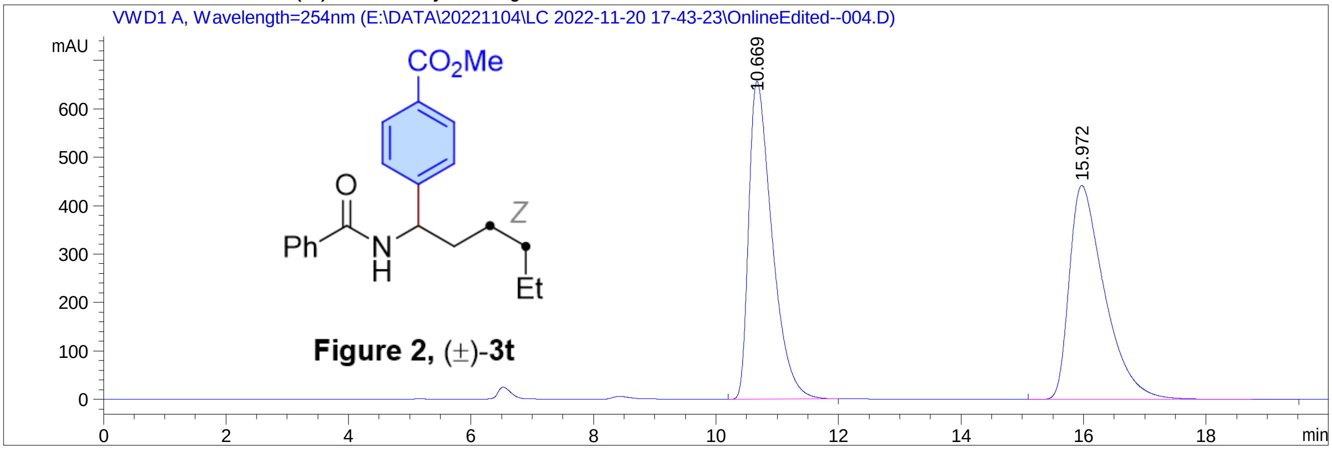

===

Area Percent Report

===

Sorted By : Signal
Multiplier : 1.0000
Dilution : 1.0000
Do not use Multiplier & Dilution Factor with ISTDs

Signal 1: VWD1 A, Wavelength=254 nm

Peak #	RetTime [min]	Type	Width [min]	Area [mAU*s]	Height [mAU]	Area %
1	10.669	BB	0.4003	1.73551e4	656.66211	49.5860
2	15.972	BB	0.5949	1.76449e4	441.80365	50.4140

Totals : 3.50000e4 1098.46576

===

*** End of Report ***

Data File E:\DATA\20221104\LC 2022-11-20 17-43-23\OnlineEdited--005.D
Sample Name: ZJQ-02-50-4

===

Acq. Operator : SYSTEM Seq. Line : 5
Acq. Instrument : HPLC1260 Location : P2-E11
Injection Date : 11/20/2022 7:08:33 PM Inj : 1
Inj Volume : 3.000 µl
Different Inj Volume from Sample Entry! Actual Inj Volume : 1.000 µl
Acq. Method : E:\DATA\20221104\LC 2022-11-20 17-43-23\20IPA-20-0.8-1-ZJQ.M
Last changed : 11/20/2022 5:43:23 PM by SYSTEM
Analysis Method : E:\DATA\20221104\LC 2022-11-20 17-43-23\20IPA-20-0.8-1-ZJQ.M (Sequence Method)
Last changed : 11/21/2022 9:04:49 PM by SYSTEM
(modified after loading)
Additional Info : Peak(s) manually integrated

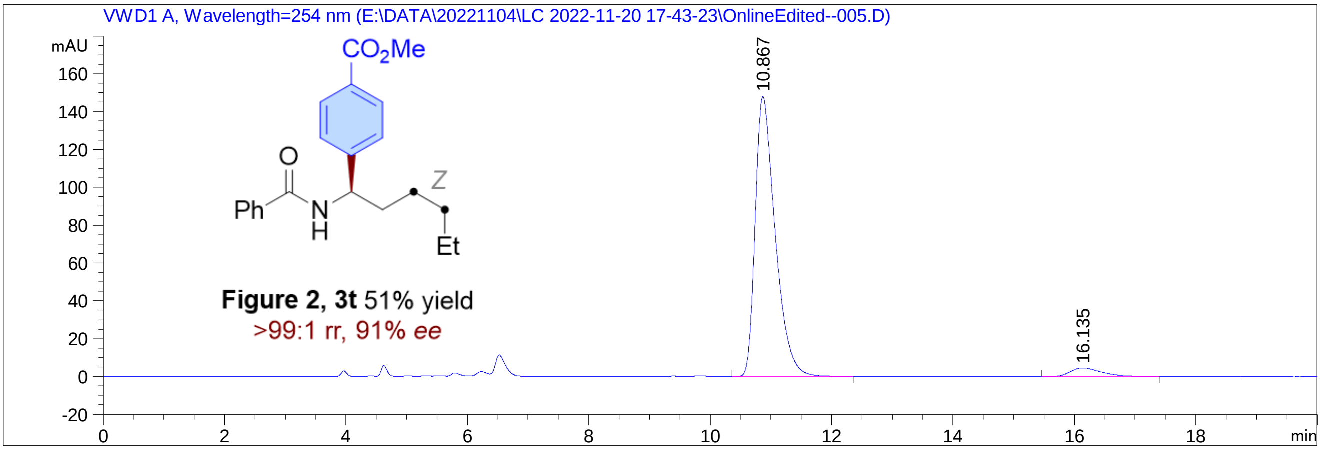

===

Area Percent Report

===

Sorted By : Signal
Multiplier : 1.0000
Dilution : 1.0000
Do not use Multiplier & Dilution Factor with ISTDs

Signal 1: VWD1 A, Wavelength=254 nm

Peak #	RetTime [min]	Type	Width [min]	Area [mAU*s]	Height [mAU]	Area %
1	10.867	BB	0.3442	3391.13403	148.05844	95.3557
2	16.135	BB	0.5240	165.16675	4.62055	4.6443

Totals : 3556.30078 152.67899

===

*** End of Report ***

Data File E:\DATA\20221104\LC 2022-12-09 10-39-59\OnlineEdited--055.D
Sample Name: ZJQ-02-72-5

===

Acq. Operator   : SYSTEM   Seq. Line : 55
Acq. Instrument : HPLC1260   Location : P2-E4
Injection Date : 12/10/2022 8:04:53 AM   Inj : 1
                                               Inj Volume : 3.000 µl
Different Inj Volume from Sample Entry! Actual Inj Volume : 8.000 µl
Acq. Method   : E:\DATA\20221104\LC 2022-12-09 10-39-59\20IPA-20-0.8-1-254-ZJQ.M
Last changed   : 12/9/2022 10:48:07 PM by SYSTEM
Analysis Method : E:\DATA\20221104\LC 2022-12-09 10-39-59\20IPA-20-0.8-1-254-ZJQ.M (Sequence Method)
Last changed   : 12/10/2022 8:38:56 AM by SYSTEM
                  (modified after loading)

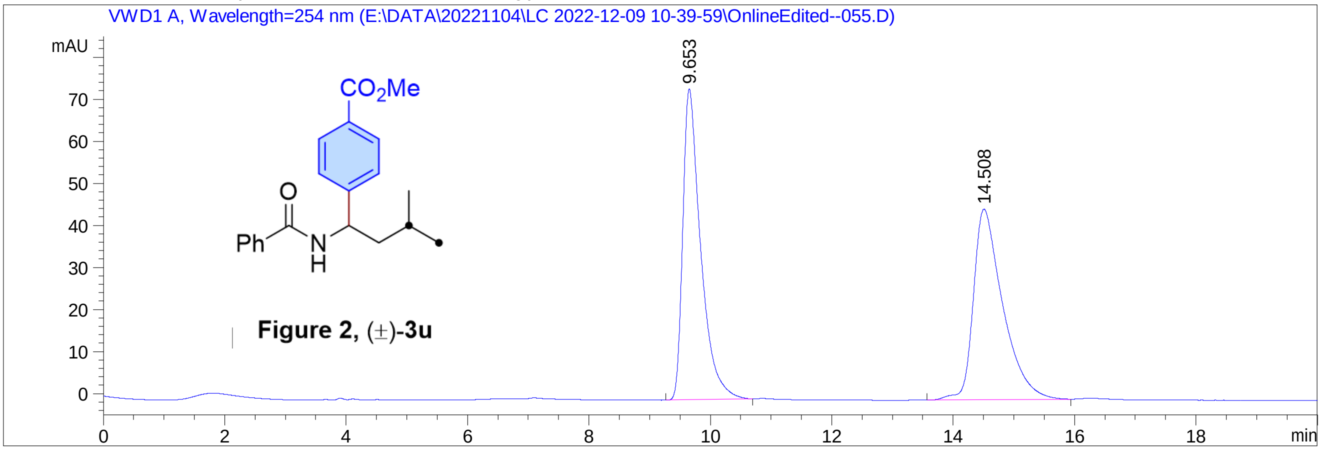

===

Area Percent Report

===

Sorted By : Signal
Multiplier : 1.0000
Dilution : 1.0000
Do not use Multiplier & Dilution Factor with ISTDs

Signal 1: VWD1 A, Wavelength=254 nm

Peak #	RetTime [min]	Type	Width [min]	Area [mAU*s]	Height [mAU]	Area %
1	9.653	BB	0.2990	1513.94641	73.89471	49.8158
2	14.508	BB	0.4906	1525.14026	45.32661	50.1842

Totals : 3039.08667 119.22131

===

*** End of Report ***

Data File E:\DATA\20221104\LC 2022-11-25 08-34-13\OnlineEdited--051.D
Sample Name: ZJQ-02-57-1

===

Acq. Operator : SYSTEM Seq. Line : 51
Acq. Instrument : HPLC1260 Location : P2-A5
Injection Date : 11/26/2022 5:29:56 AM Inj : 1
Inj Volume : 3.000 µl
Different Inj Volume from Sample Entry! Actual Inj Volume : 1.000 µl
Acq. Method : E:\DATA\20221104\LC 2022-11-25 08-34-13\20IPA-20-0.8-1-254-ZJQ.M
Last changed : 11/25/2022 11:21:52 AM by SYSTEM
Analysis Method : E:\DATA\20221104\LC 2022-11-25 08-34-13\20IPA-20-0.8-1-254-ZJQ.M (Sequence Method)
Last changed : 12/8/2022 2:41:05 PM by SYSTEM
(modified after loading)
Additional Info : Peak(s) manually integrated

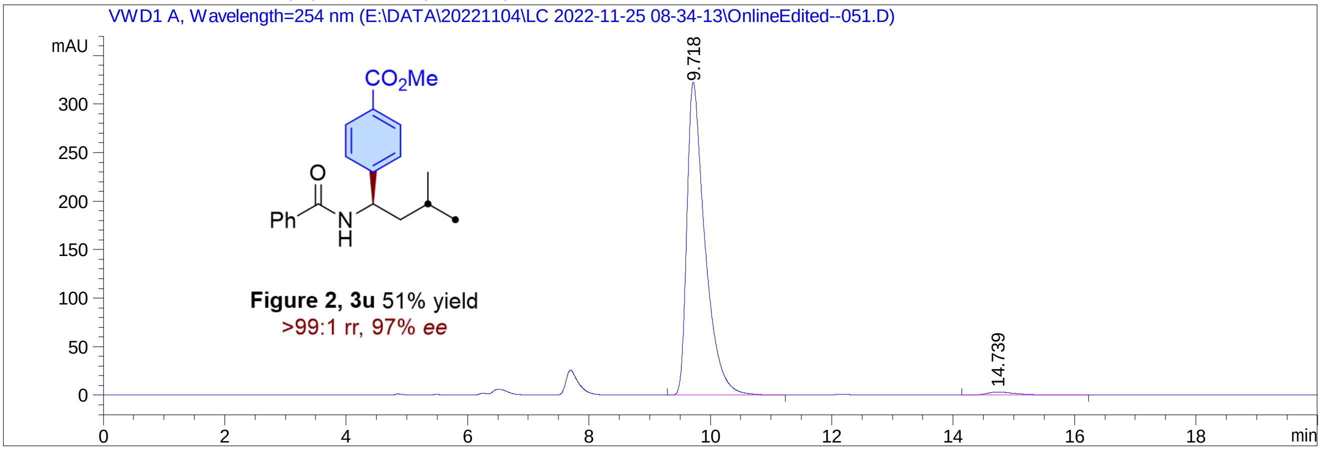

===

Area Percent Report

===

Sorted By : Signal
Multiplier : 1.0000
Dilution : 1.0000
Do not use Multiplier & Dilution Factor with ISTDs

Signal 1: VWD1 A, Wavelength=254 nm

Peak #	RetTime [min]	Type	Width [min]	Area [mAU*s]	Height [mAU]	Area %
1	9.718	BB	0.3010	6661.43359	322.49106	98.3683
2	14.739	BB	0.4783	110.49433	3.33667	1.6317

Totals : 6771.92793 325.82772

===

*** End of Report ***

Data File E:\DATA\20221216\LC 2023-04-12 17-35-12\OnlineEdited--063.D
Sample Name: ZJQ-02-196-1-RAC-195-5

===

Acq. Operator : SYSTEM Seq. Line : 63
Acq. Instrument : HPLC1260 Location : P2-C7
Injection Date : 4/13/2023 7:14:14 PM Inj : 1
Inj Volume : 3.000 µl
Different Inj Volume from Sample Entry! Actual Inj Volume : 10.000 µl
Acq. Method : E:\DATA\20221216\LC 2023-04-12 17-35-12\20IPA-20-0.8-3-254-ZJQ.M
Last changed : 4/12/2023 9:59:39 PM by SYSTEM
Analysis Method : E:\DATA\20221216\LC 2023-04-12 17-35-12\20IPA-20-0.8-3-254-ZJQ.M (Sequence Method)
Last changed : 4/14/2023 3:44:20 PM by SYSTEM
(modified after loading)
Additional Info : Peak(s) manually integrated

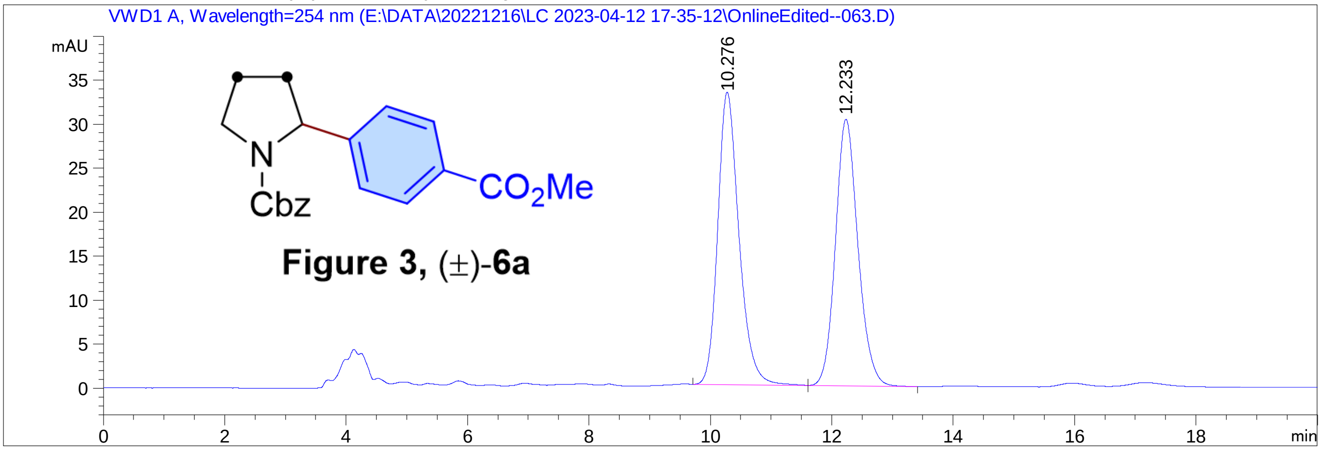

===

Area Percent Report

===

Sorted By : Signal
Multiplier : 1.0000
Dilution : 1.0000
Do not use Multiplier & Dilution Factor with ISTDs

Signal 1: VWD1 A, Wavelength=254 nm

Peak #	RetTime [min]	Type	Width [min]	Area [mAU*s]	Height [mAU]	Area %
1	10.276	BB	0.3675	807.68665	33.23817	50.8190
2	12.233	BB	0.3930	781.65295	30.29801	49.1810

Totals : 1589.33960 63.53619

===

*** End of Report ***

Data File E:\DATA\20221216\LC 2023-04-12 17-35-12\OnlineEdited--062.D
Sample Name: ZJQ-02-196-1

===

Acq. Operator : SYSTEM Seq. Line : 62
Acq. Instrument : HPLC1260 Location : P2-C4
Injection Date : 4/13/2023 6:53:21 PM Inj : 1
Inj Volume : 3.000 µl
Different Inj Volume from Sample Entry! Actual Inj Volume : 1.000 µl
Acq. Method : E:\DATA\20221216\LC 2023-04-12 17-35-12\20IPA-20-0.8-3-254-ZJQ.M
Last changed : 4/12/2023 9:59:39 PM by SYSTEM
Analysis Method : E:\DATA\20221216\LC 2023-04-12 17-35-12\20IPA-20-0.8-3-254-ZJQ.M (Sequence Method)
Last changed : 4/14/2023 3:46:41 PM by SYSTEM
(modified after loading)
Additional Info : Peak(s) manually integrated

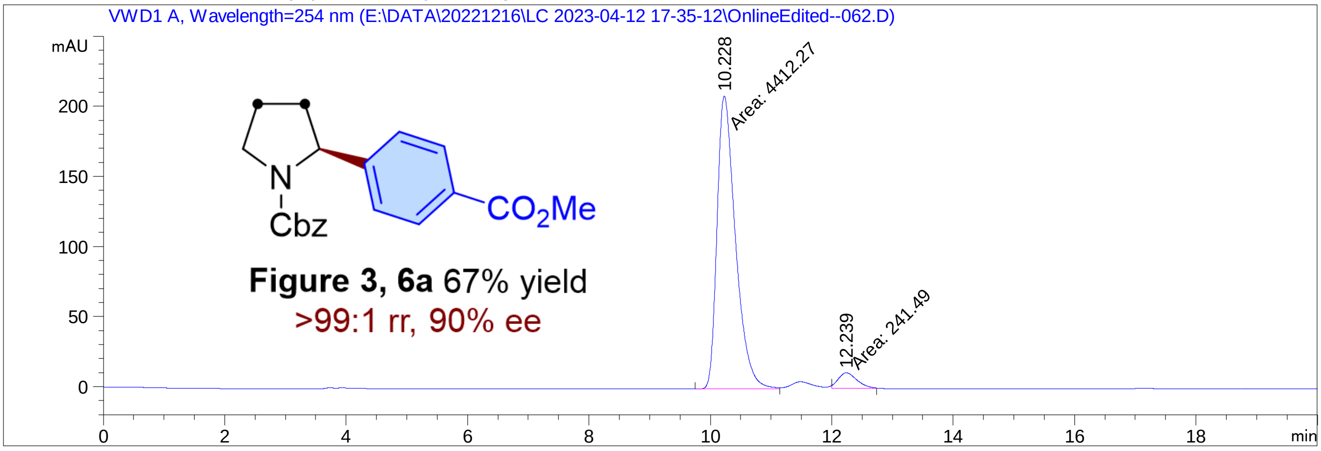

===

Area Percent Report

===

Sorted By : Signal
Multiplier : 1.0000
Dilution : 1.0000
Do not use Multiplier & Dilution Factor with ISTDs

Signal 1: VWD1 A, Wavelength=254 nm

Peak #	RetTime [min]	Type	Width [min]	Area [mAU*s]	Height [mAU]	Area %
1	10.228	MF	0.3520	4412.27002	208.93312	94.8109
2	12.239	MM	0.3611	241.49036	11.14571	5.1891

Totals : 4653.76038 220.07883

===

*** End of Report ***

Data File D:\Chem32\...-XYH 2023-06-14 17-21-14\2023-06-14ZJQ-03-47-2-OD20IPA-20-0.8-1-2.D
Sample Name: ZJQ-03-47-2-OD

===

Acq. Operator : SYSTEM Seq. Line : 14
Acq. Instrument : HPLC-1260 Location : P2-F-01
Injection Date : 6/14/2023 11:02:31 PM Inj : 1
Inj Volume : 3.000 µl
Different Inj Volume from Sample Entry! Actual Inj Volume : 8.000 µl
Acq. Method : D:\Chem32\1\Data\20230601\def_LC-XYH 2023-06-14 17-21-14\20IPA-20-0.8-1-254-ZJQ.M
Last changed : 5/25/2023 10:22:57 PM by SYSTEM
Analysis Method : D:\Chem32\1\Data\20230601\def_LC-XYH 2023-06-14 17-21-14\20IPA-20-0.8-1-254-ZJQ.M (Sequence Method)
Last changed : 6/22/2023 11:11:31 AM by SYSTEM
(modified after loading)
Additional Info : Peak(s) manually integrated

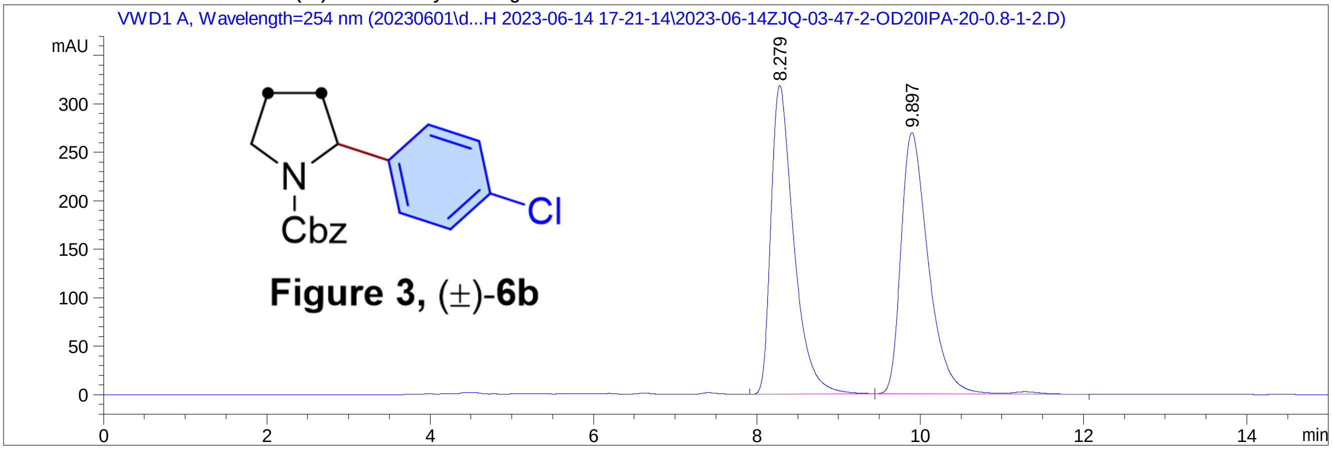

===

Area Percent Report

===

Sorted By : Signal
Multiplier : 1.0000
Dilution : 1.0000
Do not use Multiplier & Dilution Factor with ISTDs

Signal 1: VWD1 A, Wavelength=254 nm

Peak #	RetTime [min]	Type	Width [min]	Area [mAU*s]	Height [mAU]	Area %
1	8.279	BB	0.2939	6197.54834	318.73199	49.8459
2	9.897	MF	0.3856	6235.87891	269.53720	50.1541

Totals : 1.24334e4 588.26920

===

*** End of Report ***

Data File D:\Chem32\...-XYH 2023-06-26 14-21-52\2023-06-26ZJQ-03-5720IPA-15-0.8-1-254-ZJ.D
Sample Name: ZJQ-03-57

===

```
Acq. Operator   : SYSTEM                          Seq. Line :  13
Acq. Instrument : HPLC-1260                        Location :   P2-D-01
Injection Date  : 6/26/2023 6:28:11 PM                  Inj :   1
                                                 Inj Volume : 3.000 µl
Acq. Method     : D:\Chem32\1\Data\20230601\def_LC-XYH 2023-06-26 14-21-52\20IPA-15-0.8-1-254
                  -ZJQ.M
Last changed    : 5/27/2023 10:12:10 AM by SYSTEM
Analysis Method : D:\Chem32\1\Data\20230601\def_LC-XYH 2023-06-26 14-21-52\20IPA-15-0.8-1-254
                  -ZJQ.M (Sequence Method)
Last changed    : 7/27/2023 3:40:38 PM by SYSTEM
                  (modified after loading)
```

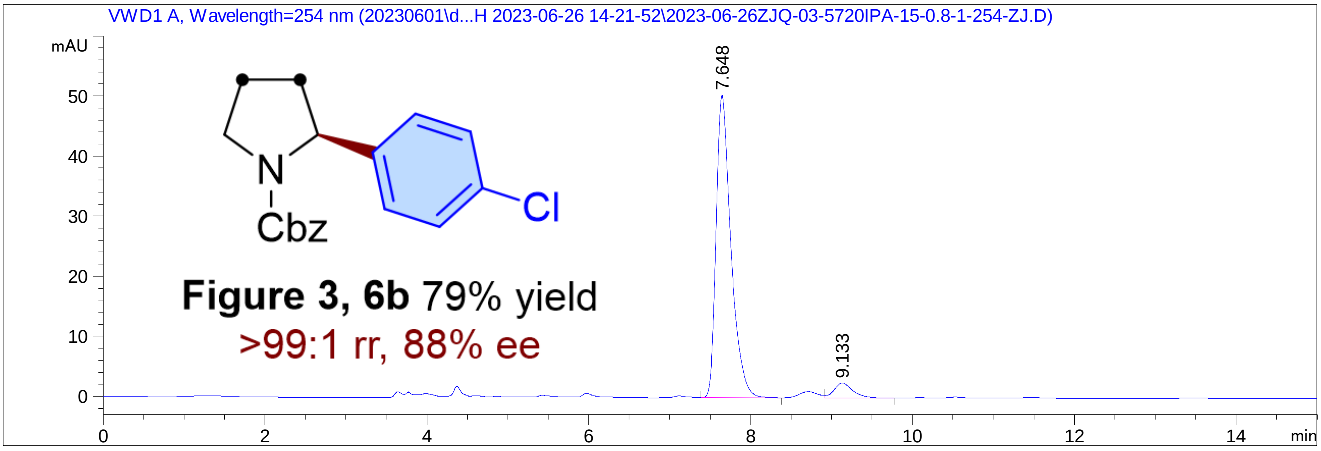

===

Area Percent Report

===

```
Sorted By             :      Signal
Multiplier            :      1.0000
Dilution              :      1.0000
Do not use Multiplier & Dilution Factor with ISTDs
```

Signal 1: VWD1 A, Wavelength=254 nm

Peak #	RetTime [min]	Type	Width [min]	Area [mAU*s]	Height [mAU]	Area %
1	7.648	BB	0.1904	640.40204	50.35560	94.1363
2	9.133	VB	0.2385	39.89042	2.49475	5.8637

Totals : 680.29246 52.85034

===

*** End of Report ***

Data File E:\DATA\20221216\LC 2023-05-11 15-02-03\2FI-0201.D
Sample Name: ZJQ-02-189-RAC-02-111-1

===

Acq. Operator : SYSTEM Seq. Line : 2
Acq. Instrument : HPLC1260 Location : P2-F9
Injection Date : 5/11/2023 3:15:39 PM Inj : 1
Inj Volume : 3.000 µl
Different Inj Volume from Sample Entry! Actual Inj Volume : 10.000 µl
Acq. Method : E:\DATA\20221216\LC 2023-05-11 15-02-03\20IPA-20-0.8-3-254-ZJQ.M
Last changed : 5/11/2023 3:02:05 PM by SYSTEM
Analysis Method : E:\DATA\20221216\LC 2023-05-11 15-02-03\20IPA-20-0.8-3-254-ZJQ.M (Sequence Method)
Last changed : 5/11/2023 4:37:21 PM by SYSTEM
(modified after loading)
Additional Info : Peak(s) manually integrated

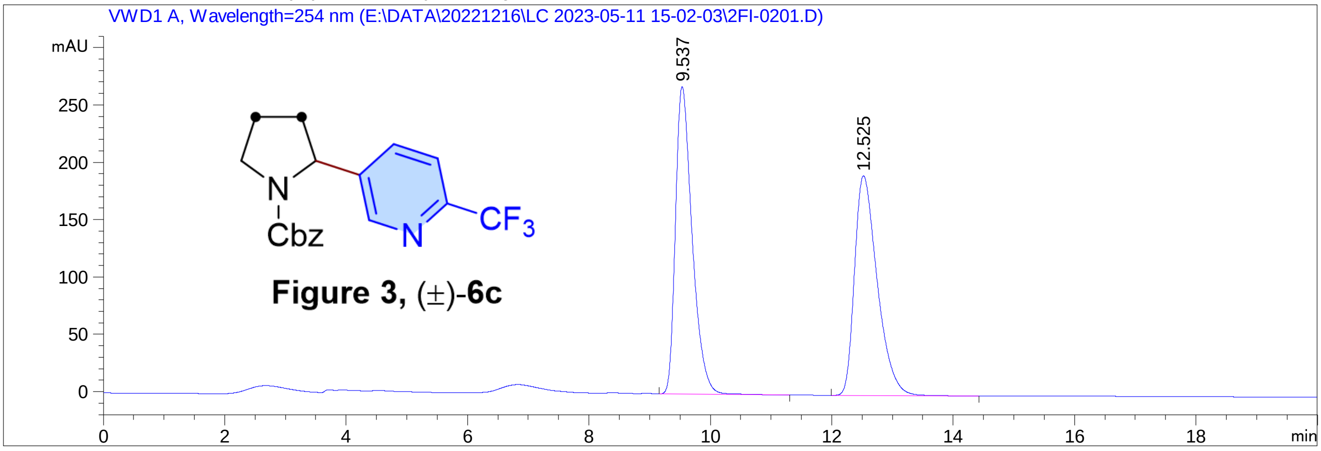

===

Area Percent Report

===

Sorted By : Signal
Multiplier : 1.0000
Dilution : 1.0000
Do not use Multiplier & Dilution Factor with ISTDs

Signal 1: VWD1 A, Wavelength=254 nm

Peak #	RetTime [min]	Type	Width [min]	Area [mAU*s]	Height [mAU]	Area %
1	9.537	BB	0.2901	5126.03467	268.07434	50.7136
2	12.525	BB	0.3964	4981.76709	191.58496	49.2864

Totals : 1.01078e4 459.65930

===

*** End of Report ***

Data File E:\DATA\20221216\LC 2023-05-11 15-02-03\2FJ-0301.D
Sample Name: ZJQ-02-189

===

Acq. Operator : SYSTEM   Seq. Line : 3
Acq. Instrument : HPLC1260   Location : P2-F10
Injection Date : 5/11/2023 3:36:30 PM   Inj : 1
Inj Volume : 3.000 µl
Different Inj Volume from Sample Entry! Actual Inj Volume : 0.800 µl
Acq. Method : E:\DATA\20221216\LC 2023-05-11 15-02-03\20IPA-20-0.8-3-254-ZJQ.M
Last changed : 5/11/2023 3:02:05 PM by SYSTEM
Analysis Method : E:\DATA\20221216\LC 2023-05-11 15-02-03\20IPA-20-0.8-3-254-ZJQ.M (Sequence Method)
Last changed : 5/11/2023 4:38:41 PM by SYSTEM
(modified after loading)
Additional Info : Peak(s) manually integrated

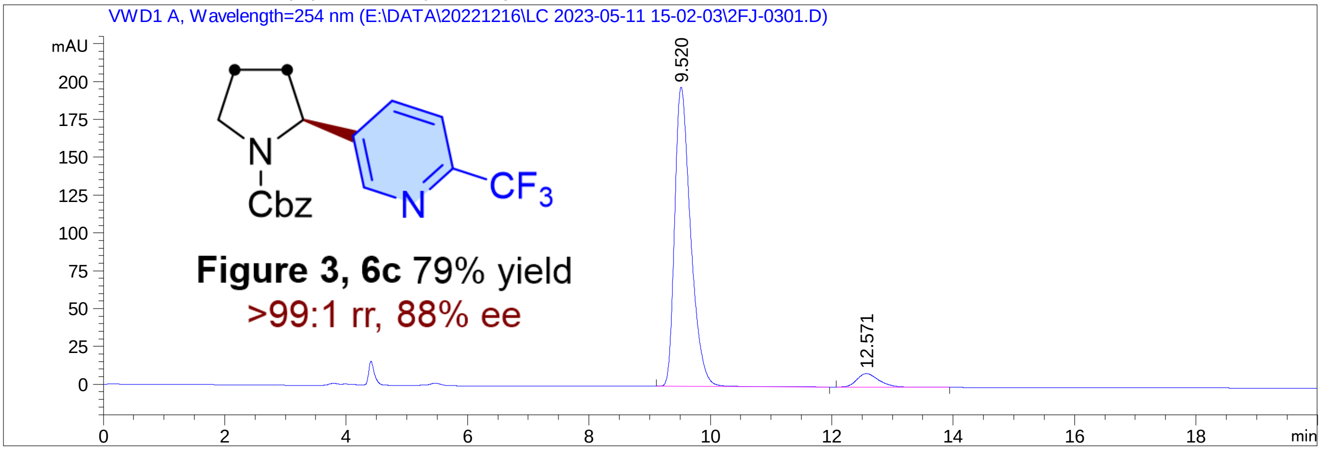

===

Area Percent Report

===

Sorted By : Signal
Multiplier : 1.0000
Dilution : 1.0000
Do not use Multiplier & Dilution Factor with ISTDs

Signal 1: VWD1 A, Wavelength=254 nm

Peak #	RetTime [min]	Type	Width [min]	Area [mAU*s]	Height [mAU]	Area %
1	9.520	BB	0.2771	3611.23682	197.70540	94.1621
2	12.571	BB	0.3809	223.88994	8.91792	5.8379

Totals : 3835.12675 206.62331

===

*** End of Report ***

Data File E:\DATA\20221216\LC 2023-04-04 16-41-21\OnlineEdited--007.D
Sample Name: ZJQ-02-190-1-RAC

===

```
Acq. Operator   : SYSTEM                          Seq. Line :    7
Acq. Instrument : HPLC1260                         Location :    P2-C2
Injection Date  : 4/4/2023 6:35:03 PM                   Inj :    1
                                                 Inj Volume : 3.000 µl
Different Inj Volume from Sample Entry!  Actual Inj Volume : 10.000 µl
Acq. Method     : E:\DATA\20221216\LC 2023-04-04 16-41-21\20IPA-20-0.8-3-254-ZJQ.M
Last changed    : 4/4/2023 5:34:27 PM by SYSTEM
Analysis Method : E:\DATA\20221216\LC 2023-04-04 16-41-21\20IPA-20-0.8-3-254-ZJQ.M (Sequence
                  Method)
Last changed    : 4/4/2023 7:54:45 PM by SYSTEM
                  (modified after loading)
Additional Info : Peak(s) manually integrated
```

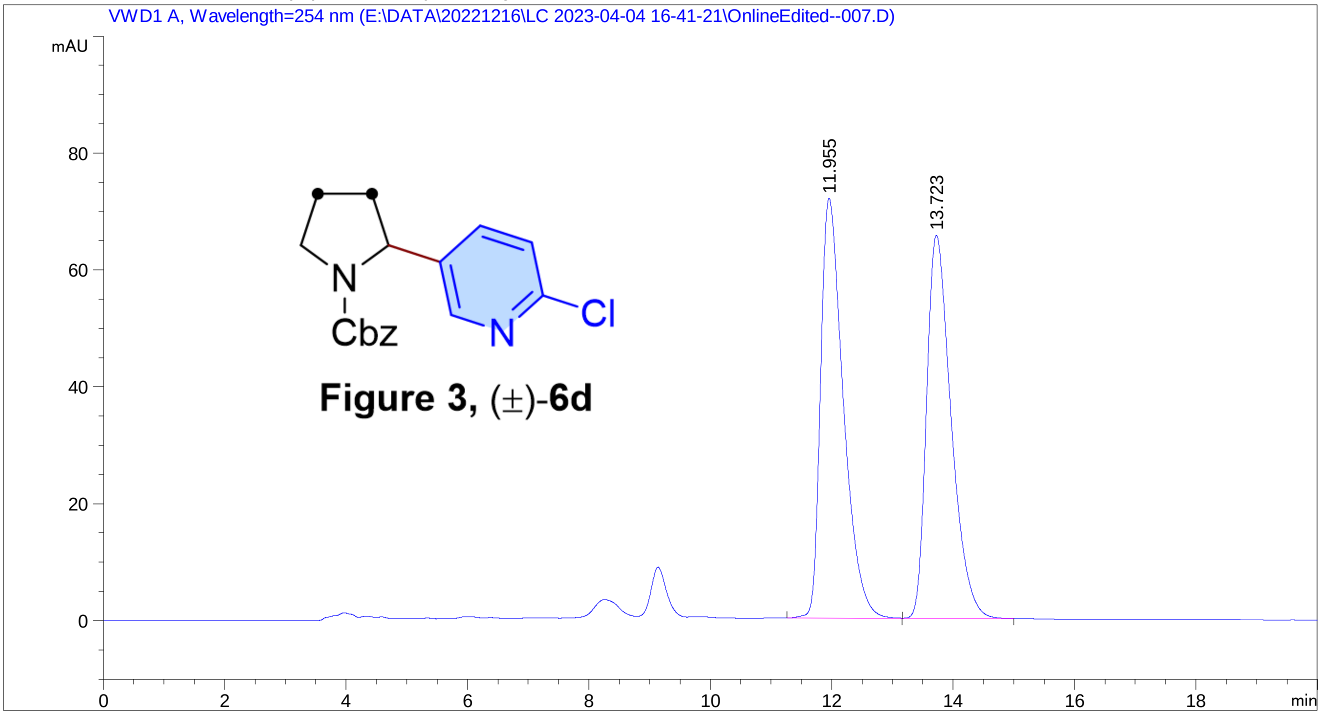

===

Area Percent Report

===

```
Sorted By             :      Signal
Multiplier            :      1.0000
Dilution              :      1.0000
Use Multiplier & Dilution Factor with ISTDs
```

Signal 1: VWD1 A, Wavelength=254 nm

Peak #	RetTime [min]	Type	Width [min]	Area [mAU*s]	Height [mAU]	Area %
1	11.955	BB	0.3959	1870.12244	71.78347	50.3406
2	13.723	BB	0.4294	1844.81262	65.53631	49.6594

Totals : 3714.93506 137.31978

Data File E:\DATA\20221216\LC 2023-04-04 16-41-21\OnlineEdited--002.D
Sample Name: ZJQ-02-190-1

===

Acq. Operator : SYSTEM Seq. Line : 2
Acq. Instrument : HPLC1260 Location : P2-C1
Injection Date : 4/4/2023 4:54:00 PM Inj : 1
Inj Volume : 3.000 µl
Acq. Method : E:\DATA\20221216\LC 2023-04-04 16-41-21\20IPA-20-0.8-3-254-ZJQ.M
Last changed : 4/4/2023 4:41:22 PM by SYSTEM
Analysis Method : E:\DATA\20221216\LC 2023-04-04 16-41-21\20IPA-20-0.8-3-254-ZJQ.M (Sequence Method)
Last changed : 4/4/2023 7:53:10 PM by SYSTEM
(modified after loading)
Additional Info : Peak(s) manually integrated

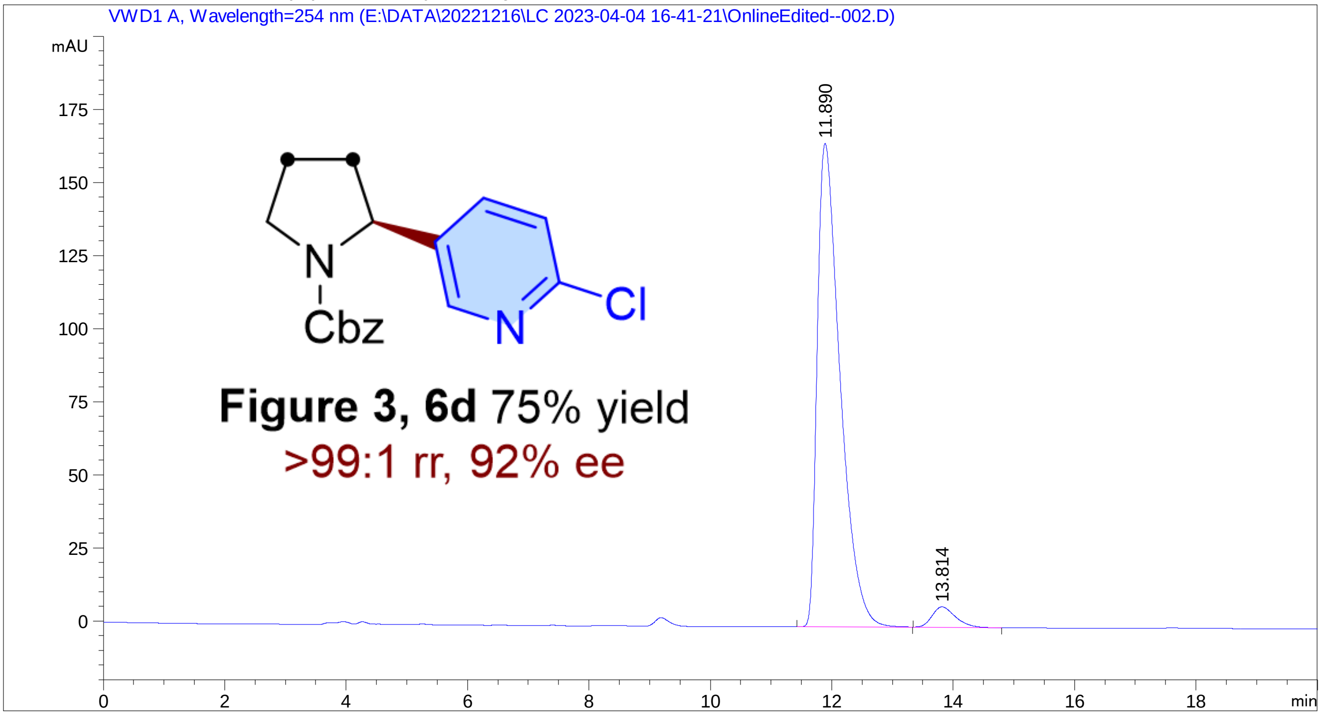

===

Area Percent Report

===

Sorted By : Signal
Multiplier : 1.0000
Dilution : 1.0000
Use Multiplier & Dilution Factor with ISTDs

Signal 1: VWD1 A, Wavelength=254 nm

Peak #	RetTime [min]	Type	Width [min]	Area [mAU*s]	Height [mAU]	Area %
1	11.890	BB	0.3959	4319.79395	165.29918	95.8732
2	13.814	BB	0.4039	185.94501	6.97699	4.1268

Totals : 4505.73895 172.27617

Data File E:\DATA\20221216\LC 2023-04-04 16-41-21\OnlineEdited--005.D
Sample Name: ZJQ-02-190-2-RAC

===

Acq. Operator : SYSTEM Seq. Line : 5
Acq. Instrument : HPLC1260 Location : P2-C4
Injection Date : 4/4/2023 5:53:21 PM Inj : 1
Inj Volume : 3.000 µl
Different Inj Volume from Sample Entry! Actual Inj Volume : 8.000 µl
Acq. Method : E:\DATA\20221216\LC 2023-04-04 16-41-21\20IPA-20-0.8-3-254-ZJQ.M
Last changed : 4/4/2023 5:34:27 PM by SYSTEM
Analysis Method : E:\DATA\20221216\LC 2023-04-04 16-41-21\20IPA-20-0.8-3-254-ZJQ.M (Sequence Method)
Last changed : 4/4/2023 7:56:21 PM by SYSTEM
(modified after loading)

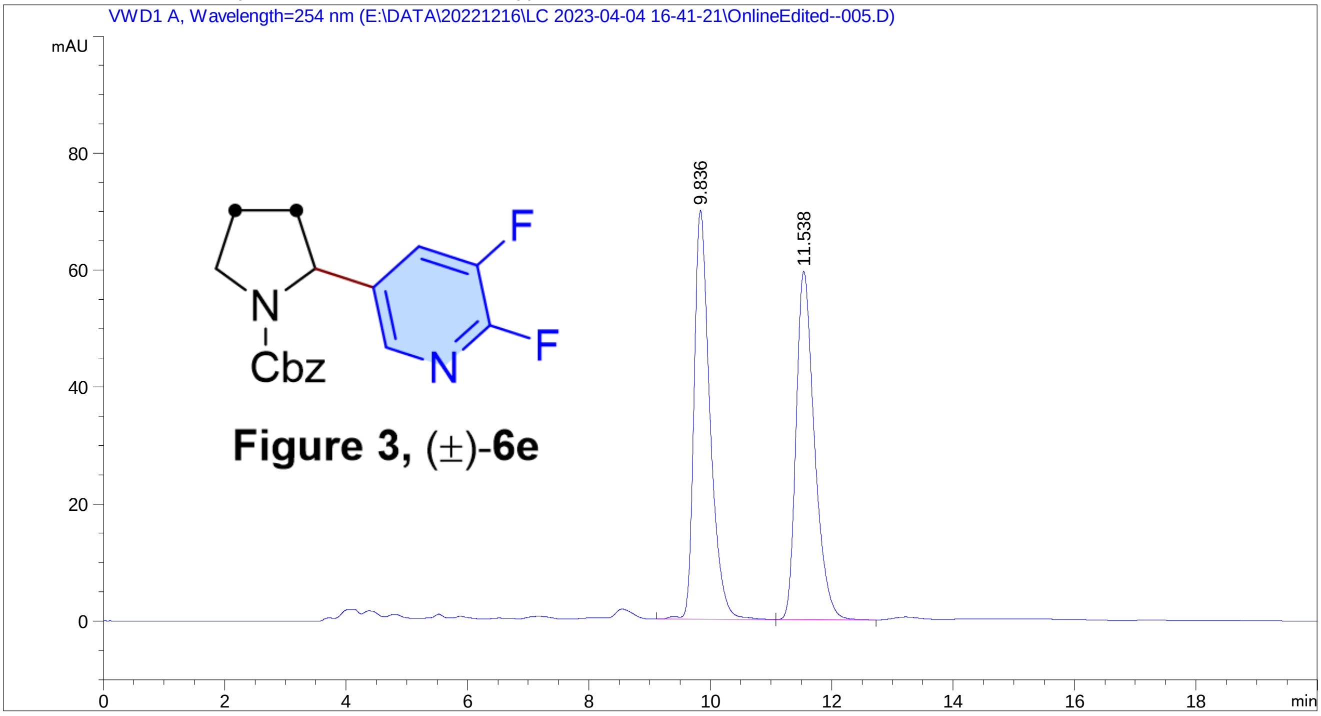

===

Area Percent Report

===

Sorted By : Signal
Multiplier : 1.0000
Dilution : 1.0000
Use Multiplier & Dilution Factor with ISTDs

Signal 1: VWD1 A, Wavelength=254 nm

Peak #	RetTime [min]	Type	Width [min]	Area [mAU*s]	Height [mAU]	Area %
1	9.836	VB R	0.2741	1276.55286	69.89426	50.4601
2	11.538	BB	0.3186	1253.27112	59.53934	49.5399

Totals : 2529.82397 129.43361

```
Data File E:\DATA\20221216\LC 2023-04-10 20-20-53\OnlineEdited--005.D
Sample Name: ZJQ-02-194-4

=====================================================================
Acq. Operator   : SYSTEM                          Seq. Line :   5
Acq. Instrument : HPLC1260                         Location :   P2-C9
Injection Date  : 4/10/2023 9:25:36 PM                  Inj :   1
                                                 Inj Volume : 3.000 µl
Acq. Method     : E:\DATA\20221216\LC 2023-04-10 20-20-53\20IPA-20-0.8-3-254-ZJQ.M
Last changed    : 4/10/2023 8:29:54 PM by SYSTEM
Analysis Method : E:\DATA\20221216\LC 2023-04-10 20-20-53\20IPA-20-0.8-3-254-ZJQ.M (Sequence
                  Method)
Last changed    : 4/10/2023 10:01:28 PM by SYSTEM
                  (modified after loading)
Additional Info : Peak(s) manually integrated
```

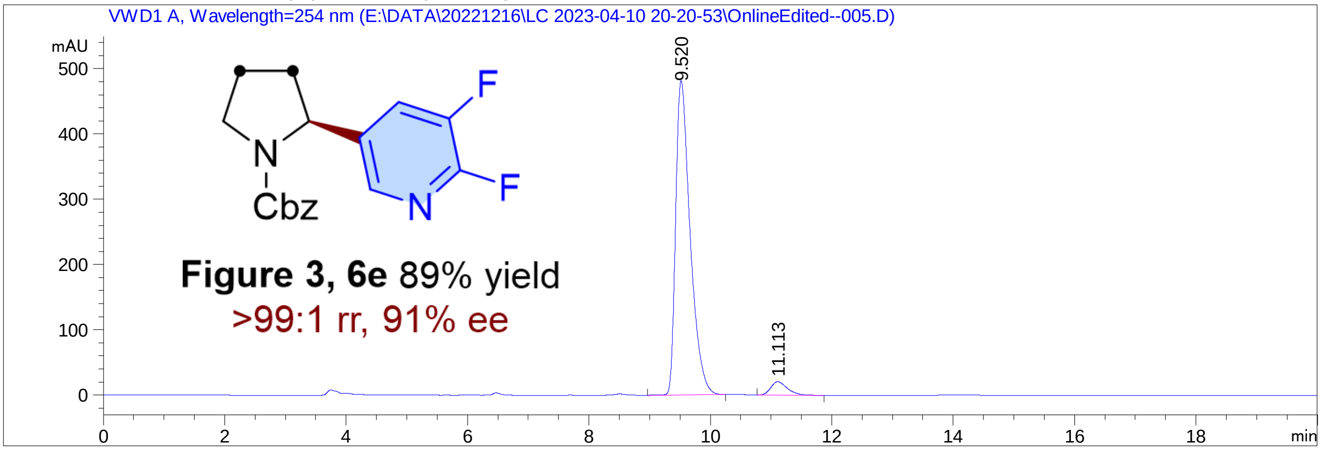

===

Area Percent Report

===

```
Sorted By             :      Signal
Multiplier            :      1.0000
Dilution              :      1.0000
Do not use Multiplier & Dilution Factor with ISTDs
```

Signal 1: VWD1 A, Wavelength=254 nm

Peak #	RetTime [min]	Type	Width [min]	Area [mAU*s]	Height [mAU]	Area %
1	9.520	VB R	0.2507	7996.83447	481.62646	95.3745
2	11.113	BB	0.2844	387.83405	20.62519	4.6255

Totals : 8384.66852 502.25166

===

*** End of Report ***

Data File E:\DATA\20221216\LC 2023-04-14 08-18-20\2CD-0201.D
Sample Name: ZJQ-02-201-2-RAC-202-2

===

Acq. Operator : SYSTEM Seq. Line : 2
Acq. Instrument : HPLC1260 Location : P2-C4
Injection Date : 4/14/2023 8:31:36 AM Inj : 1
Inj Volume : 3.000 µl
Different Inj Volume from Sample Entry! Actual Inj Volume : 20.000 µl
Acq. Method : E:\DATA\20221216\LC 2023-04-14 08-18-20\30IPA-20-0.8-3-254-ZJQ.M
Last changed : 4/14/2023 8:18:22 AM by SYSTEM
Analysis Method : E:\DATA\20221216\LC 2023-04-14 08-18-20\30IPA-20-0.8-3-254-ZJQ.M (Sequence Method)
Last changed : 4/14/2023 10:56:06 AM by SYSTEM
(modified after loading)
Additional Info : Peak(s) manually integrated

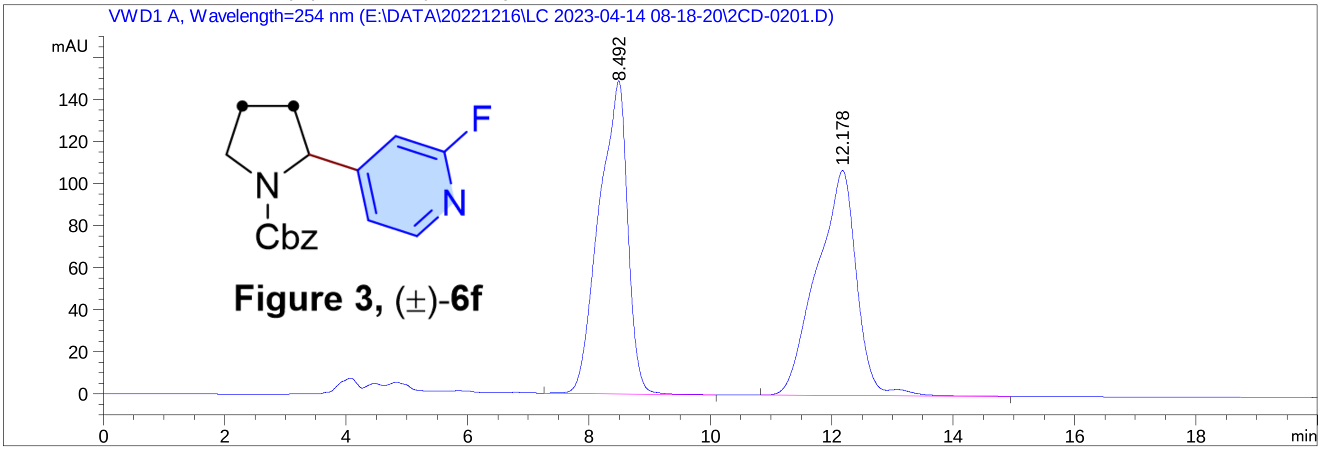

===

Area Percent Report

===

Sorted By : Signal
Multiplier : 1.0000
Dilution : 1.0000
Do not use Multiplier & Dilution Factor with ISTDs

Signal 1: VWD1 A, Wavelength=254 nm

Peak #	RetTime [min]	Type	Width [min]	Area [mAU*s]	Height [mAU]	Area %
1	8.492	BB	0.4405	4867.47949	148.83578	49.9078
2	12.178	BV R	0.6176	4885.46191	107.00351	50.0922

Totals : 9752.94141 255.83929

===

*** End of Report ***

Data File E:\DATA\20221216\LC 2023-04-14 08-18-20\2CC-0301.D
Sample Name: ZJQ-02-201-2

===

Acq. Operator : SYSTEM Seq. Line : 3
Acq. Instrument : HPLC1260 Location : P2-C3
Injection Date : 4/14/2023 8:52:28 AM Inj : 1
Inj Volume : 3.000 µl
Different Inj Volume from Sample Entry! Actual Inj Volume : 2.000 µl
Acq. Method : E:\DATA\20221216\LC 2023-04-14 08-18-20\30IPA-20-0.8-3-254-ZJQ.M
Last changed : 4/14/2023 8:18:22 AM by SYSTEM
Analysis Method : E:\DATA\20221216\LC 2023-04-14 08-18-20\30IPA-20-0.8-3-254-ZJQ.M (Sequence Method)
Last changed : 4/14/2023 10:57:24 AM by SYSTEM
(modified after loading)
Additional Info : Peak(s) manually integrated

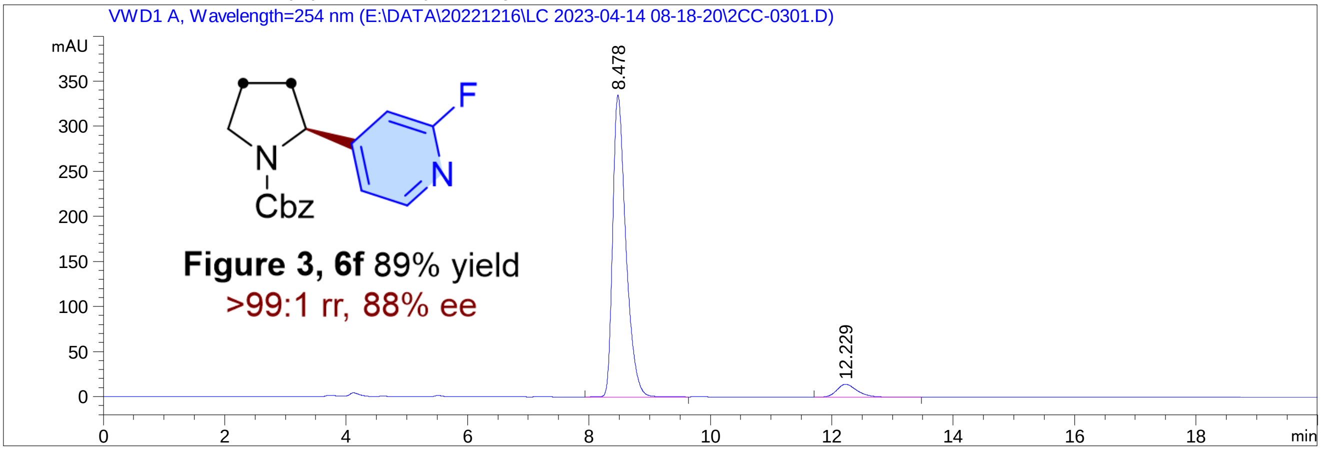

===

Area Percent Report

===

Sorted By : Signal
Multiplier : 1.0000
Dilution : 1.0000
Do not use Multiplier & Dilution Factor with ISTDs

Signal 1: VWD1 A, Wavelength=254 nm

Peak #	RetTime [min]	Type	Width [min]	Area [mAU*s]	Height [mAU]	Area %
1	8.478	BB	0.2275	5040.99756	335.07785	93.8858
2	12.229	BB	0.3514	328.29025	14.16239	6.1142

Totals : 5369.28781 349.24024

===

*** End of Report ***

Data File E:\DATA\20221216\LC 2023-04-15 14-55-27\OnlineEdited--011.D
Sample Name: ZJQ-03-10-2

===

Acq. Operator : SYSTEM Seq. Line : 11
Acq. Instrument : HPLC1260 Location : P1-F8
Injection Date : 4/15/2023 5:47:13 PM Inj : 1
Inj Volume : 3.000 µl
Different Inj Volume from Sample Entry! Actual Inj Volume : 8.000 µl
Acq. Method : E:\DATA\20221216\LC 2023-04-15 14-55-27\20IPA-20-0.8-3-254-ZJQ.M
Last changed : 4/15/2023 4:05:45 PM by SYSTEM
Analysis Method : E:\DATA\20221216\LC 2023-04-15 14-55-27\20IPA-20-0.8-3-254-ZJQ.M (Sequence Method)
Last changed : 4/20/2023 10:14:27 PM by SYSTEM
(modified after loading)

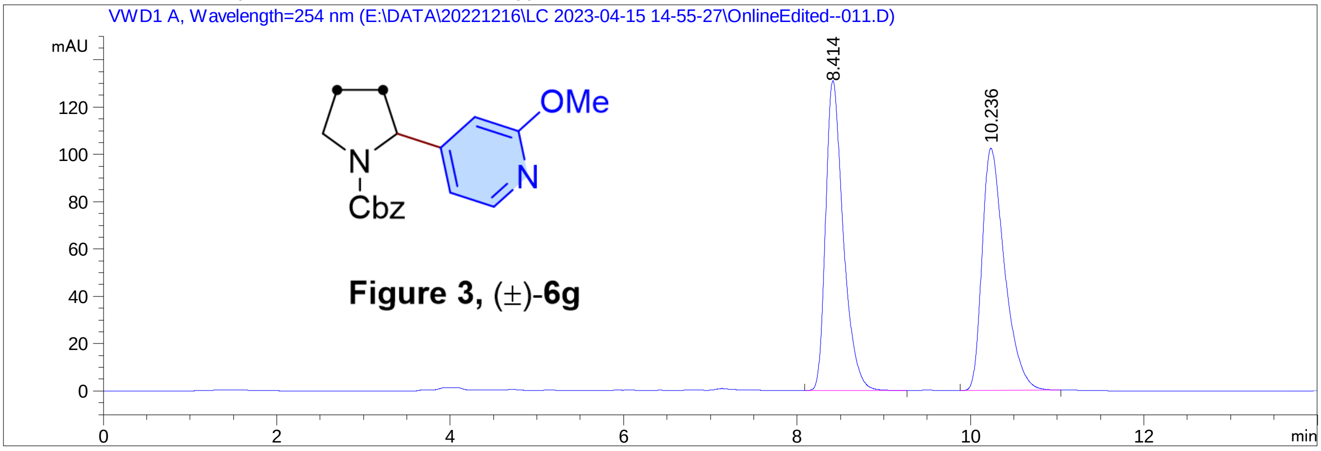

Figure 3, (±)-6g

===

Area Percent Report

===

Sorted By : Signal
Multiplier : 1.0000
Dilution : 1.0000
Do not use Multiplier & Dilution Factor with ISTDs

Signal 1: VWD1 A, Wavelength=254 nm

Peak #	RetTime [min]	Type	Width [min]	Area [mAU*s]	Height [mAU]	Area %
1	8.414	BB	0.2133	1856.69019	131.06975	50.1440
2	10.236	BB	0.2722	1846.02917	102.44252	49.8560

Totals : 3702.71936 233.51227

===

*** End of Report ***

Data File E:\DATA\20221216\LC 2023-04-04 16-41-21\OnlineEdited--006.D
Sample Name: ZJQ-02-190-3

===

Acq. Operator : SYSTEM Seq. Line : 6
Acq. Instrument : HPLC1260 Location : P2-C5
Injection Date : 4/4/2023 6:14:11 PM Inj : 1
Inj Volume : 3.000 µl
Acq. Method : E:\DATA\20221216\LC 2023-04-04 16-41-21\20IPA-20-0.8-3-254-ZJQ.M
Last changed : 4/4/2023 5:34:27 PM by SYSTEM
Analysis Method : E:\DATA\20221216\LC 2023-04-04 16-41-21\20IPA-20-0.8-3-254-ZJQ.M (Sequence Method)
Last changed : 4/20/2023 10:06:15 PM by SYSTEM
(modified after loading)

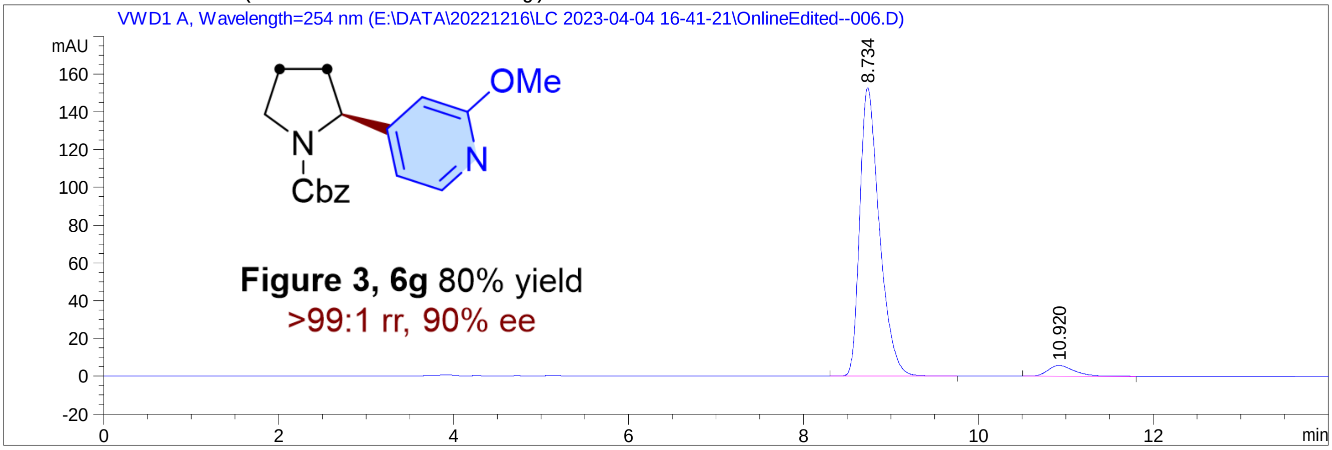

===

Area Percent Report

===

Sorted By : Signal
Multiplier : 1.0000
Dilution : 1.0000
Do not use Multiplier & Dilution Factor with ISTDs

Signal 1: VWD1 A, Wavelength=254 nm

Peak #	RetTime [min]	Type	Width [min]	Area [mAU*s]	Height [mAU]	Area %
1	8.734	BB	0.2362	2382.18286	152.53630	95.2359
2	10.920	BB	0.3150	119.16640	5.74423	4.7641

Totals : 2501.34926 158.28053

===

*** End of Report ***

Data File E:\DATA\20221216\LC 2023-04-07 09-27-27\OnlineEdited--011.D
Sample Name: ZJQ-02-192-3-RAC

===

Acq. Operator : SYSTEM Seq. Line : 11
Acq. Instrument : HPLC1260 Location : P2-C2
Injection Date : 4/7/2023 12:58:24 PM Inj : 1
Inj Volume : 3.000 µl
Different Inj Volume from Sample Entry! Actual Inj Volume : 10.000 µl
Acq. Method : E:\DATA\20221216\LC 2023-04-07 09-27-27\20IPA-20-0.8-1-254-ZJQ.M
Last changed : 4/7/2023 12:03:01 PM by SYSTEM
Analysis Method : E:\DATA\20221216\LC 2023-04-07 09-27-27\20IPA-20-0.8-1-254-ZJQ.M (Sequence Method)
Last changed : 4/8/2023 10:35:34 AM by SYSTEM
(modified after loading)
Additional Info : Peak(s) manually integrated

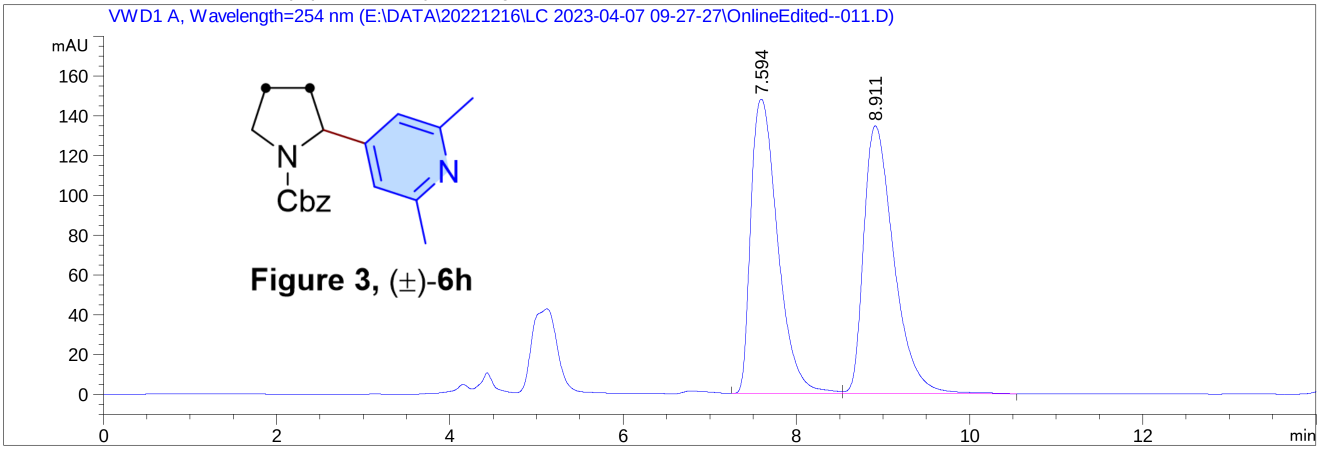

===

Area Percent Report

===

Sorted By : Signal
Multiplier : 1.0000
Dilution : 1.0000
Do not use Multiplier & Dilution Factor with ISTDs

Signal 1: VWD1 A, Wavelength=254 nm

Peak #	RetTime [min]	Type	Width [min]	Area [mAU*s]	Height [mAU]	Area %
1	7.594	BV	0.3368	3191.73853	147.89349	49.5284
2	8.911	VB	0.3719	3252.51929	134.58577	50.4716

Totals : 6444.25781 282.47926

===

*** End of Report ***

Data File E:\DATA\20221216\LC 2023-04-07 09-27-27\OnlineEdited--012.D
Sample Name: ZJQ-02-192-3

===

Acq. Operator : SYSTEM Seq. Line : 12
Acq. Instrument : HPLC1260 Location : P2-C1
Injection Date : 4/7/2023 1:19:14 PM Inj : 1
Inj Volume : 3.000 µl
Acq. Method : E:\DATA\20221216\LC 2023-04-07 09-27-27\20IPA-20-0.8-1-254-ZJQ.M
Last changed : 4/7/2023 12:03:01 PM by SYSTEM
Analysis Method : E:\DATA\20221216\LC 2023-04-07 09-27-27\20IPA-20-0.8-1-254-ZJQ.M (Sequence Method)
Last changed : 4/8/2023 10:34:08 AM by SYSTEM
(modified after loading)
Additional Info : Peak(s) manually integrated

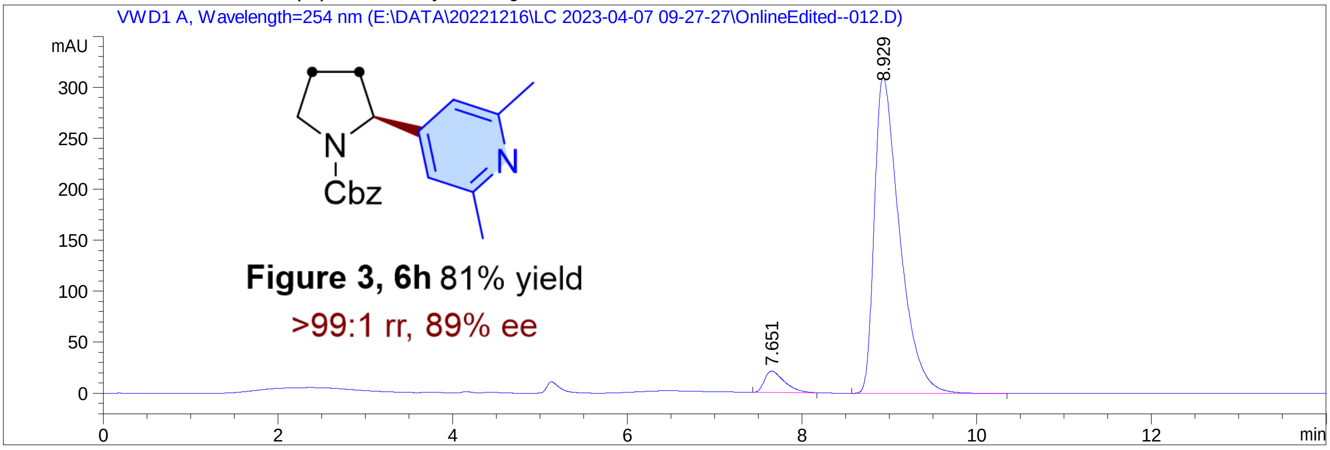

===

Area Percent Report

===

Sorted By : Signal
Multiplier : 1.0000
Dilution : 1.0000
Do not use Multiplier & Dilution Factor with ISTDs

Signal 1: VWD1 A, Wavelength=254 nm

Peak #	RetTime [min]	Type	Width [min]	Area [mAU*s]	Height [mAU]	Area %
1	7.651	MM	0.2625	330.44479	20.98177	5.0376
2	8.929	BB	0.2991	6229.12988	310.48026	94.9624

Totals : 6559.57468 331.46202

===

*** End of Report ***

Data File D:\Chem32\...-XYH 2023-11-16 15-46-41\2023-11-16ZJQ-03-152-120IPA-20-0.8-4-254.D
Sample Name: ZJQ-03-152-1-RAC

===

Acq. Operator : SYSTEM Seq. Line : 11
Acq. Instrument : HPLC-1260 Location : P2-C-11
Injection Date : 11/16/2023 6:55:38 PM Inj : 1
Inj Volume : 3.000 µl
Different Inj Volume from Sample Entry! Actual Inj Volume : 10.000 µl
Acq. Method : D:\Chem32\1\Data\20231013\def_LC-XYH 2023-11-16 15-46-41\20IPA-20-0.8-4-254-ZJQ.M
Last changed : 11/16/2023 3:04:00 PM by SYSTEM
Analysis Method : D:\Chem32\1\Data\20231013\def_LC-XYH 2023-11-16 15-46-41\20IPA-20-0.8-4-254-ZJQ.M (Sequence Method)
Last changed : 11/19/2023 6:22:53 PM by SYSTEM
(modified after loading)
Additional Info : Peak(s) manually integrated

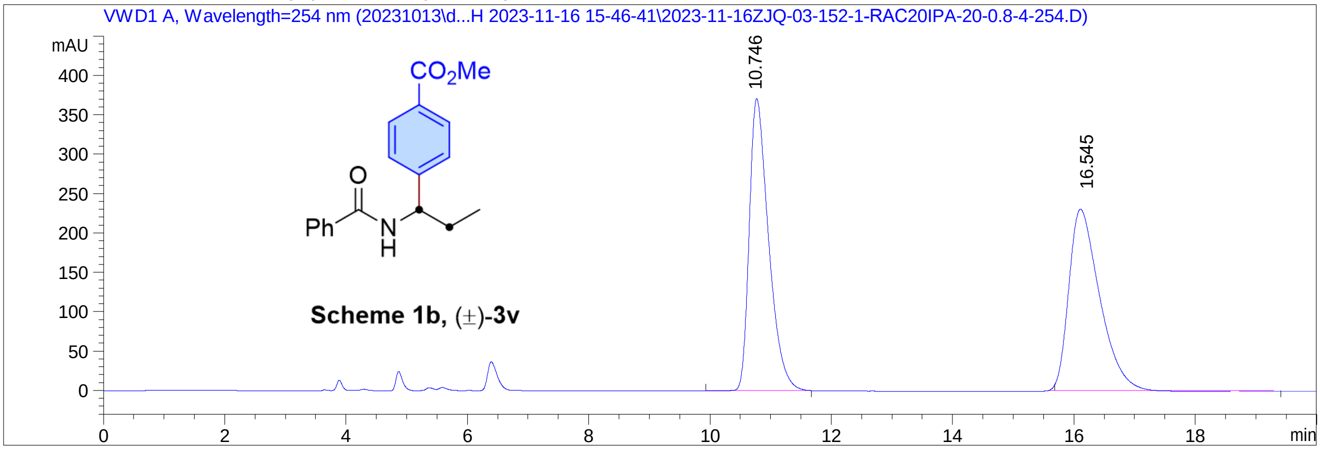

===

Area Percent Report

===

Sorted By : Signal
Multiplier : 1.0000
Dilution : 1.0000
Do not use Multiplier & Dilution Factor with ISTDs

Signal 1: VWD1 A, Wavelength=254 nm

Peak #	RetTime [min]	Type	Width [min]	Area [mAU*s]	Height [mAU]	Area %
1	10.746	BB	0.3164	6950.76204	360.76496	49.9368
2	16.545	BB	0.6472	9790.12518	247.45378	50.0632

Totals : 1.6741e9 608.21874

===

*** End of Report ***

Data File D:\Chem32\...-XYH 2023-11-16 15-46-41\2023-11-16ZJQ-03-152-120IPA-20-0.8-4-254.D
Sample Name: ZJQ-03-152-1

===

Acq. Operator : SYSTEM Seq. Line : 10
Acq. Instrument : HPLC-1260 Location : P2-C-10
Injection Date : 11/16/2023 6:55:38 PM Inj : 1
Inj Volume : 3.000 µl
Different Inj Volume from Sample Entry! Actual Inj Volume : 10.000 µl
Acq. Method : D:\Chem32\1\Data\20231013\def_LC-XYH 2023-11-16 15-46-41\20IPA-20-0.8-4-254-ZJQ.M
Last changed : 11/16/2023 3:24:30 PM by SYSTEM
Analysis Method : D:\Chem32\1\Data\20231013\def_LC-XYH 2023-11-16 15-46-41\20IPA-20-0.8-4-254-ZJQ.M (Sequence Method)
Last changed : 11/19/2023 6:21:33 PM by SYSTEM
(modified after loading)
Additional Info : Peak(s) manually integrated

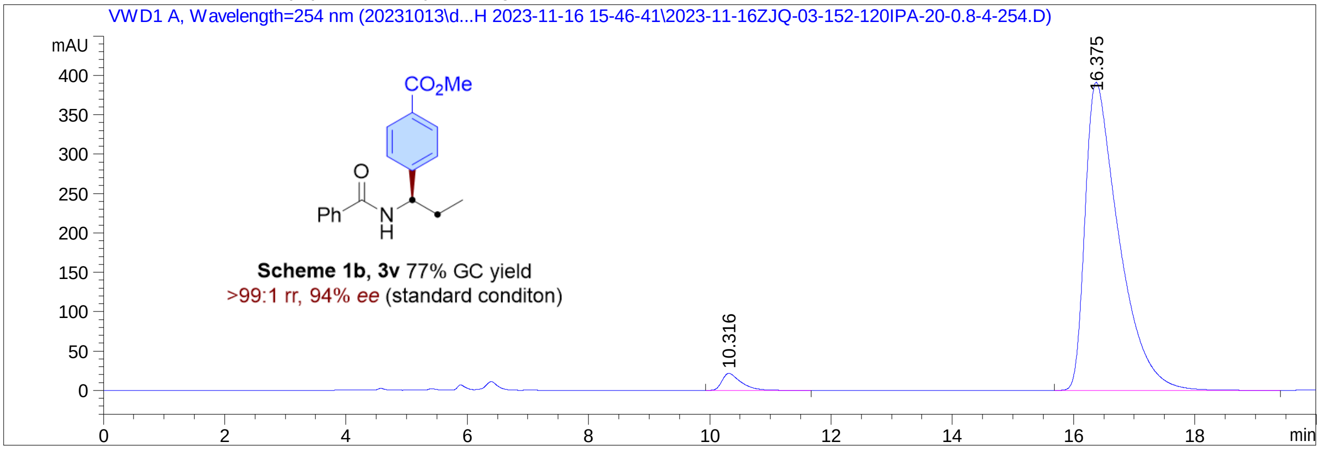

===

Area Percent Report

===

Sorted By : Signal
Multiplier : 1.0000
Dilution : 1.0000
Do not use Multiplier & Dilution Factor with ISTDs

Signal 1: VWD1 A, Wavelength=254 nm

Peak #	RetTime [min]	Type	Width [min]	Area [mAU*s]	Height [mAU]	Area %
1	10.316	BB	0.3164	466.02899	21.54496	2.9830
2	16.375	BB	0.5672	1.51568e4	391.04315	97.0170

Totals : 1.56228e4 412.58811

===

*** End of Report ***

Data File D:\Chem32\...-XYH 2023-11-07 10-10-31\2023-11-08ZJQ-03-146-13IPA-15-0.8-1-254-.D
Sample Name: ZJQ-03-146-1-RAC

===

Acq. Operator : SYSTEM Seq. Line : 72
Acq. Instrument : HPLC-1260 Location : P2-B-02
Injection Date : 11/8/2023 12:33:53 PM Inj : 1
Inj Volume : 3.000 µl
Different Inj Volume from Sample Entry! Actual Inj Volume : 2.000 µl
Acq. Method : D:\Chem32\1\Data\20231013\def_LC-XYH 2023-11-07 10-10-31\3IPA-15-0.8-1-254-ZJQ.M
Last changed : 10/31/2023 9:42:50 AM by SYSTEM
Analysis Method : D:\Chem32\1\Data\20231013\def_LC-XYH 2023-11-07 10-10-31\3IPA-15-0.8-1-254-ZJQ.M (Sequence Method)
Last changed : 11/19/2023 3:04:39 PM by SYSTEM
(modified after loading)
Additional Info : Peak(s) manually integrated

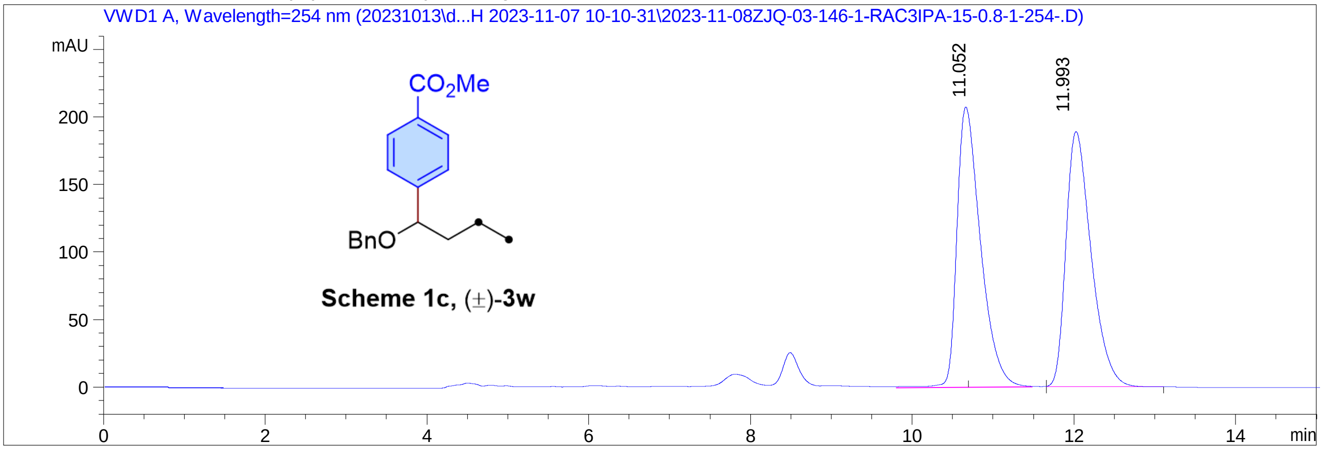

===

Area Percent Report

===

Sorted By : Signal
Multiplier : 1.0000
Dilution : 1.0000
Do not use Multiplier & Dilution Factor with ISTDs

Signal 1: VWD1 A, Wavelength=254 nm

Peak #	RetTime [min]	Type	Width [min]	Area [mAU*s]	Height [mAU]	Area %
1	11.052	BB	0.3959	5250.56833	216.74532	50.4789
2	11.993	BB	0.4037	4680.40238	189.30734	49.5211

Totals : 9930.97071 406.05266

===

*** End of Report ***

Data File D:\Chem32\...-XYH 2023-11-07 10-10-31\2023-11-08ZJQ-03-146-13IPA-15-0.8-1-254-.D
Sample Name: ZJQ-03-146-1

===
Acq. Operator : SYSTEM Seq. Line : 71
Acq. Instrument : HPLC-1260 Location : P2-B-01
Injection Date : 11/8/2023 12:33:53 PM Inj : 1
Inj Volume : 3.000 µl
Different Inj Volume from Sample Entry! Actual Inj Volume : 2.000 µl
Acq. Method : D:\Chem32\1\Data\20231013\def_LC-XYH 2023-11-07 10-10-31\3IPA-15-0.8-1-254-ZJQ.M
Last changed : 10/31/2023 9:21:49 AM by SYSTEM
Analysis Method : D:\Chem32\1\Data\20231013\def_LC-XYH 2023-11-07 10-10-31\3IPA-15-0.8-1-254-ZJQ.M (Sequence Method)
Last changed : 11/19/2023 3:02:19 PM by SYSTEM
(modified after loading)
Additional Info : Peak(s) manually integrated

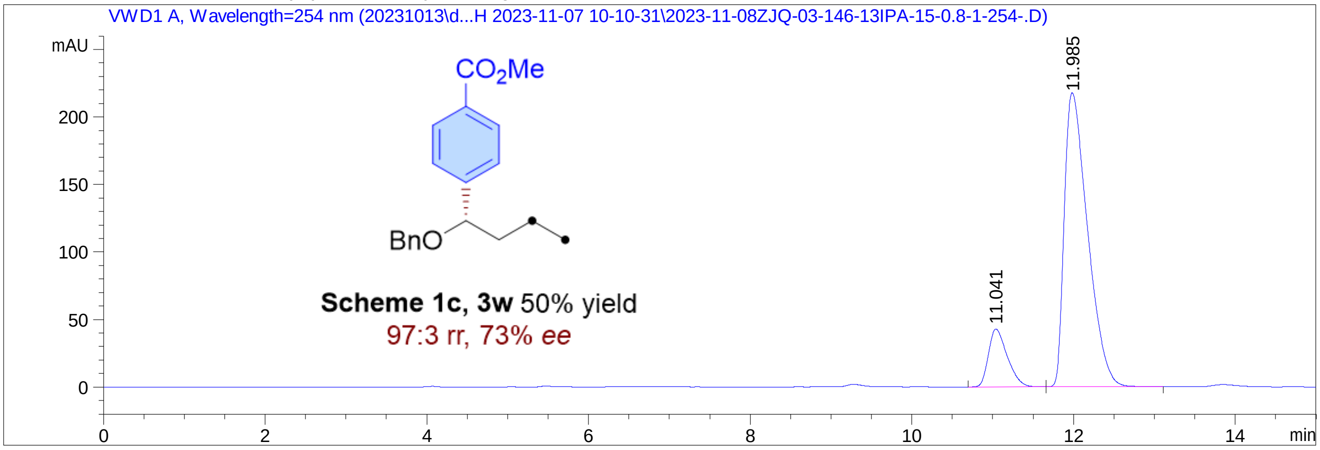

===
Area Percent Report
===

Sorted By : Signal
Multiplier : 1.0000
Dilution : 1.0000
Do not use Multiplier & Dilution Factor with ISTDs

Signal 1: VWD1 A, Wavelength=254 nm

Peak #	RetTime [min]	Type	Width [min]	Area [mAU*s]	Height [mAU]	Area %
1	11.041	BB	0.2446	691.88641	42.79332	13.6917
2	11.985	BB	0.3067	4361.42529	217.70534	86.3083

Totals : 5053.31171 260.49866

===
*** End of Report ***

Data File D:\Chem32\...-XYH 2023-11-19 14-56-16\2023-11-19ZJQ-03-147-1-AD3IPA-20-0.8-4-2.D
Sample Name: ZJQ-03-147-1-RAC

===

Acq. Operator : SYSTEM Seq. Line : 4
Acq. Instrument : HPLC-1260 Location : P1-A-06
Injection Date : 11/19/2023 3:23:31 PM Inj : 1
Inj Volume : 3.000 µl
Different Inj Volume from Sample Entry! Actual Inj Volume : 5.000 µl
Acq. Method : D:\Chem32\1\Data\20231013\def_LC-XYH 2023-11-19 14-56-16\3IPA-20-0.8-4-254-ZJQ.M
Last changed : 11/19/2023 2:37:06 PM by SYSTEM
Analysis Method : D:\Chem32\1\Data\20231013\def_LC-XYH 2023-11-19 14-56-16\3IPA-20-0.8-4-254-ZJQ.M (Sequence Method)
Last changed : 11/19/2023 4:03:28 PM by SYSTEM
(modified after loading)
Additional Info : Peak(s) manually integrated

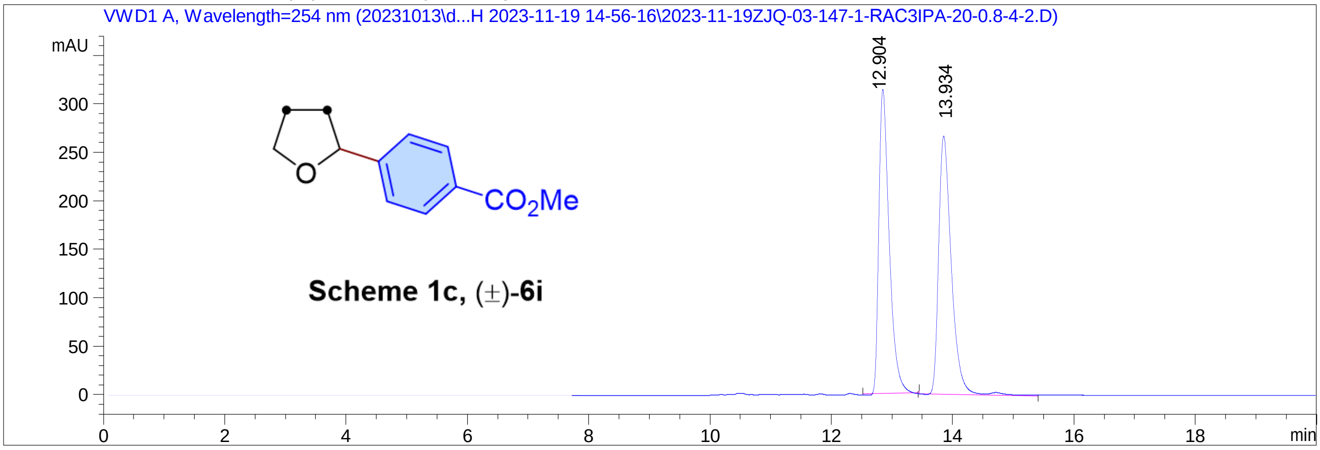

===

Area Percent Report

===

Sorted By : Signal
Multiplier : 1.0000
Dilution : 1.0000
Do not use Multiplier & Dilution Factor with ISTDs

Signal 1: VWD1 A, Wavelength=254 nm

Peak #	RetTime [min]	Type	Width [min]	Area [mAU*s]	Height [mAU]	Area %
1	12.904	MM	0.2939	6187.43628	319.24078	49.0854
2	13.934	MM	0.3657	6542.85805	275.03419	50.9146

Totals : 1.27302e4 594.27497

===

*** End of Report ***

Data File D:\Chem32\...-XYH 2023-11-19 14-56-16\2023-11-19ZJQ-03-147-1-AD3IPA-20-0.8-4-2.D
Sample Name: ZJQ-03-147-1-AD

===

Acq. Operator : SYSTEM Seq. Line : 3
Acq. Instrument : HPLC-1260 Location : P1-A-05
Injection Date : 11/19/2023 3:23:31 PM Inj : 1
Inj Volume : 3.000 µl
Different Inj Volume from Sample Entry! Actual Inj Volume : 5.000 µl
Acq. Method : D:\Chem32\1\Data\20231013\def_LC-XYH 2023-11-19 14-56-16\3IPA-20-0.8-4-254-ZJQ.M
Last changed : 11/19/2023 2:57:36 PM by SYSTEM
Analysis Method : D:\Chem32\1\Data\20231013\def_LC-XYH 2023-11-19 14-56-16\3IPA-20-0.8-4-254-ZJQ.M (Sequence Method)
Last changed : 11/19/2023 4:02:21 PM by SYSTEM
(modified after loading)
Additional Info : Peak(s) manually integrated

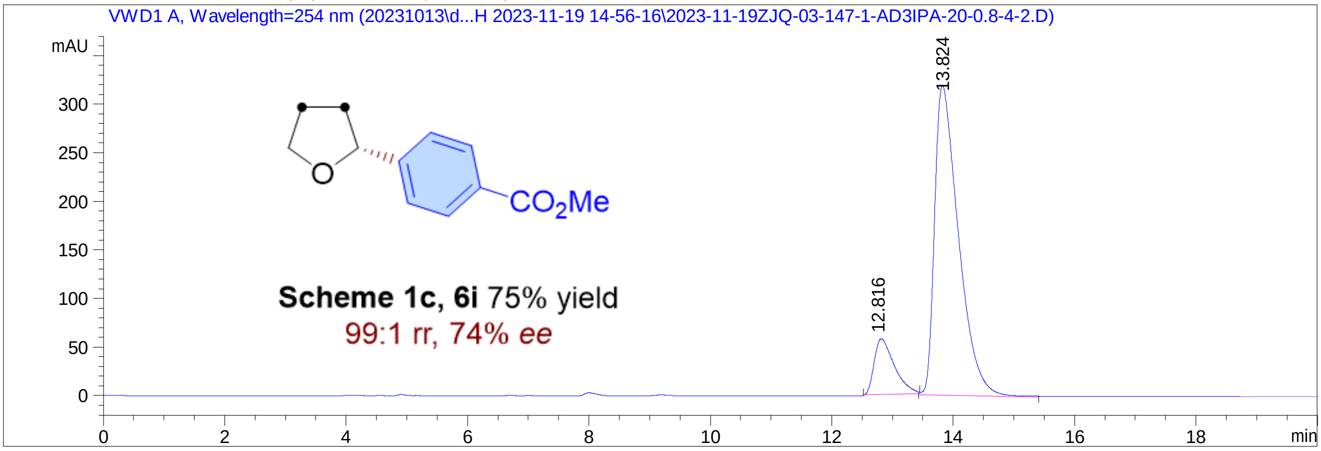

===

Area Percent Report

===

Sorted By : Signal
Multiplier : 1.0000
Dilution : 1.0000
Do not use Multiplier & Dilution Factor with ISTDs

Signal 1: VWD1 A, Wavelength=254 nm

Peak #	RetTime [min]	Type	Width [min]	Area [mAU*s]	Height [mAU]	Area %
1	12.816	MM	0.3821	1312.03296	57.22606	13.0618
2	13.824	MM	0.4563	8732.74805	318.94897	86.9382

Totals : 1.00448e4 376.17503

===

*** End of Report ***

Data File E:\DATA\20221216\LC 2023-05-10 19-50-37\OnlineEdited--026.D
Sample Name: ZJQ-03-168

===

Acq. Operator : SYSTEM Seq. Line : 26
Acq. Instrument : HPLC1260 Location : P2-F9
Injection Date : 5/11/2023 4:31:57 AM Inj : 1
Inj Volume : 3.000 µl
Different Inj Volume from Sample Entry! Actual Inj Volume : 10.000 µl
Acq. Method : E:\DATA\20221216\LC 2023-05-10 19-50-37\3IPA-25-0.8-3-254-ZJQ.M
Last changed : 5/10/2023 7:50:38 PM by SYSTEM
Analysis Method : E:\DATA\20221216\LC 2023-05-10 19-50-37\3IPA-25-0.8-3-254-ZJQ.M (Sequence Method)
Last changed : 5/11/2023 2:46:23 PM by SYSTEM
(modified after loading)
Additional Info : Peak(s) manually integrated

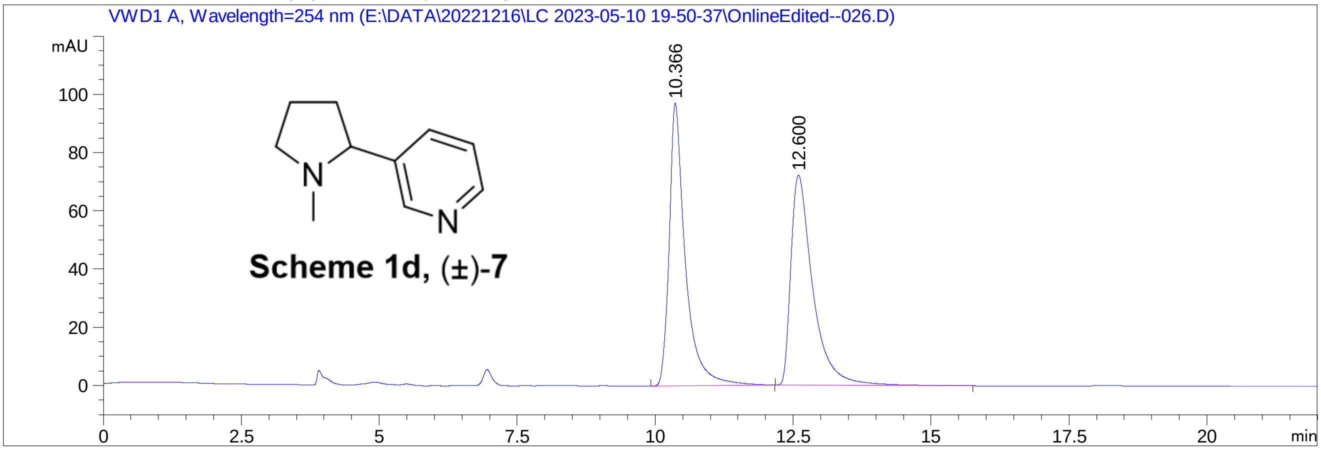

===

Area Percent Report

===

Sorted By : Signal
Multiplier : 1.0000
Dilution : 1.0000
Do not use Multiplier & Dilution Factor with ISTDs

Signal 1: VWD1 A, Wavelength=254 nm

Peak #	RetTime [min]	Type	Width [min]	Area [mAU*s]	Height [mAU]	Area %
1	10.366	BB	0.2877	1969.57104	97.09504	49.8556
2	12.600	BB	0.4088	1980.97839	72.04436	50.1444

Totals : 3950.54944 169.13940

===

*** End of Report ***

Data File E:\DATA\20221216\LC 2023-05-10 19-50-37\OnlineEdited--025.D
Sample Name: ZJQ-03-165

===

Acq. Operator : SYSTEM   Seq. Line : 25
Acq. Instrument : HPLC1260   Location : P2-F10
Injection Date : 5/11/2023 4:05:57 AM   Inj : 1
Inj Volume : 3.000 µl
Different Inj Volume from Sample Entry! Actual Inj Volume : 20.000 µl
Acq. Method : E:\DATA\20221216\LC 2023-05-10 19-50-37\3IPA-25-0.8-3-254-ZJQ.M
Last changed : 5/10/2023 7:50:38 PM by SYSTEM
Analysis Method : E:\DATA\20221216\LC 2023-05-10 19-50-37\3IPA-25-0.8-3-254-ZJQ.M (Sequence Method)
Last changed : 5/11/2023 2:44:19 PM by SYSTEM
(modified after loading)
Additional Info : Peak(s) manually integrated

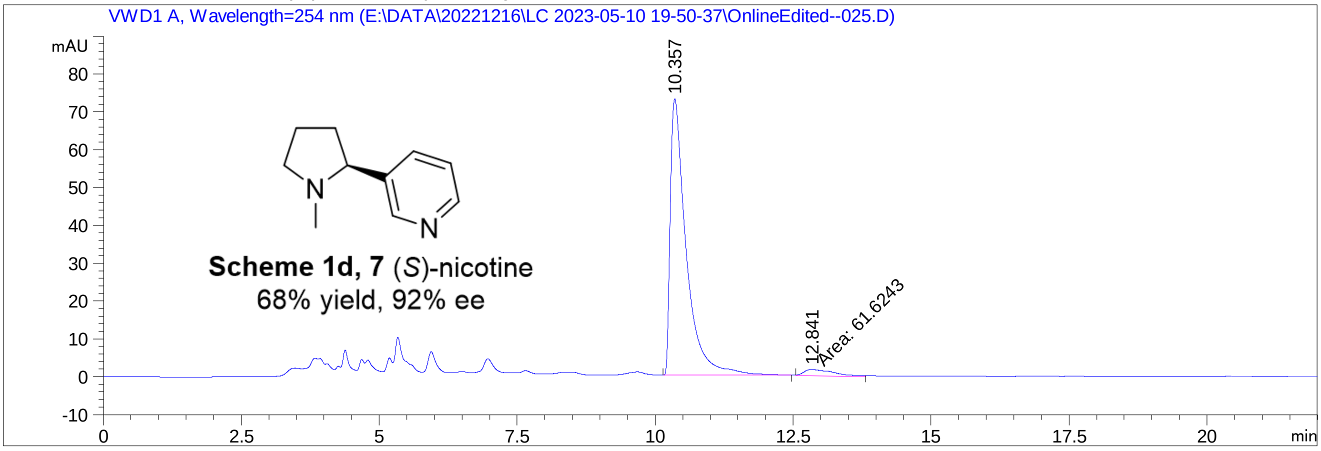

===

Area Percent Report

===

Sorted By : Signal
Multiplier : 1.0000
Dilution : 1.0000
Do not use Multiplier & Dilution Factor with ISTDs

Signal 1: VWD1 A, Wavelength=254 nm

Peak #	RetTime [min]	Type	Width [min]	Area [mAU*s]	Height [mAU]	Area %
1	10.357	BB	0.2915	1468.30261	73.05481	95.9721
2	12.841	MM	0.6208	61.62431	1.65434	4.0279

Totals : 1529.92693 74.70915

===

*** End of Report ***

Data File D:\Chem32\...-XYH 2023-07-20 21-45-00\2023-07-21ZJQ-03-P-RAC-OJ30IPA-20-0.8-31.D
Sample Name: ZJQ-03-P-RAC-OJ

===

Acq. Operator : SYSTEM Seq. Line : 16
Acq. Instrument : HPLC-1260 Location : P1-C-02
Injection Date : 7/21/2023 12:37:46 AM Inj : 2
Inj Volume : 3.000 µl
Different Inj Volume from Sample Entry! Actual Inj Volume : 10.000 µl
Acq. Method : D:\Chem32\1\Data\20230601\def_LC-XYH 2023-07-20 21-45-00\30IPA-20-0.8-3-254-ZJQ.M
Last changed : 7/20/2023 3:50:33 PM by SYSTEM
Analysis Method : D:\Chem32\1\Data\20230601\def_LC-XYH 2023-07-20 21-45-00\30IPA-20-0.8-3-254-ZJQ.M (Sequence Method)
Last changed : 7/21/2023 8:46:24 AM by SYSTEM
(modified after loading)
Additional Info : Peak(s) manually integrated

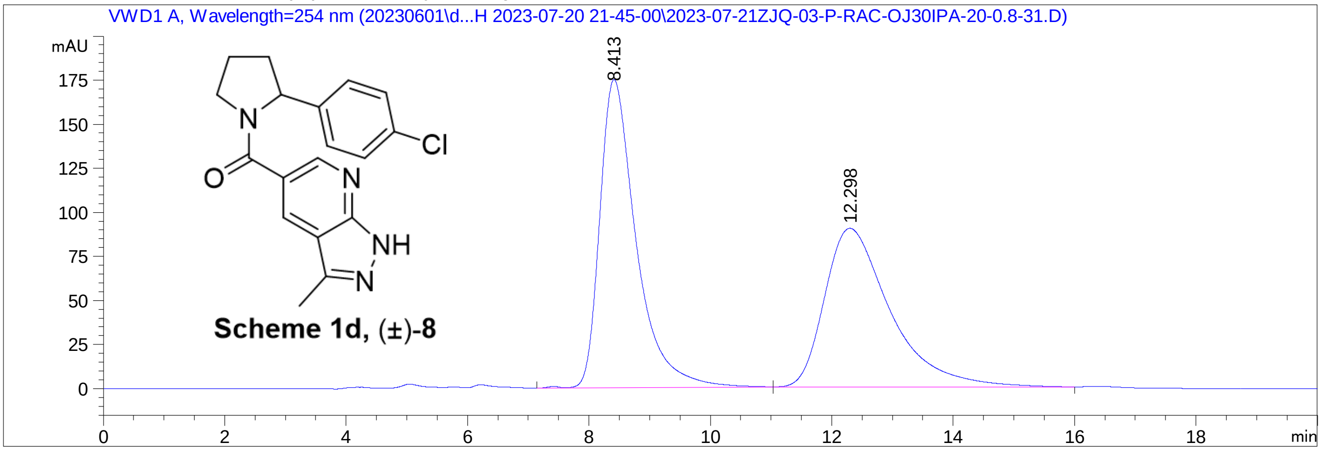

===

Area Percent Report

===

Sorted By : Signal
Multiplier : 1.0000
Dilution : 1.0000
Do not use Multiplier & Dilution Factor with ISTDs

Signal 1: VWD1 A, Wavelength=254 nm

Peak #	RetTime [min]	Type	Width [min]	Area [mAU*s]	Height [mAU]	Area %
1	8.413	VB R	0.6263	7288.31201	174.84718	51.8597
2	12.298	BB	1.1298	6765.60303	90.01867	48.1403

Totals : 1.40539e4 264.86585

===

*** End of Report ***

Data File D:\Chem32\...-XYH 2023-07-20 21-45-00\2023-07-20ZJQ-03-P-OJ30IPA-20-0.8-3-254-.D
Sample Name: ZJQ-03-P-OJ

===
Acq. Operator : SYSTEM Seq. Line : 14
Acq. Instrument : HPLC-1260 Location : P1-C-01
Injection Date : 7/20/2023 11:56:12 PM Inj : 1
Inj Volume : 3.000 µl
Different Inj Volume from Sample Entry! Actual Inj Volume : 1.000 µl
Acq. Method : D:\Chem32\1\Data\20230601\def_LC-XYH 2023-07-20 21-45-00\30IPA-20-0.8-3-254-ZJQ.M
Last changed : 7/20/2023 3:50:33 PM by SYSTEM
Analysis Method : D:\Chem32\1\Data\20230601\def_LC-XYH 2023-07-20 21-45-00\30IPA-20-0.8-3-254-ZJQ.M (Sequence Method)
Last changed : 7/21/2023 8:44:38 AM by SYSTEM
(modified after loading)
Additional Info : Peak(s) manually integrated

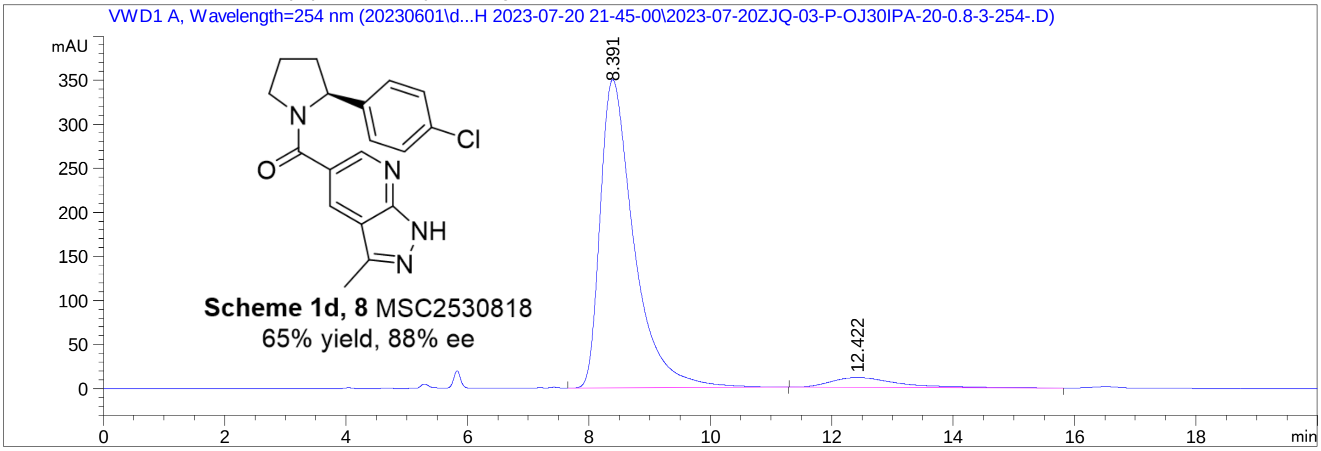

===
Area Percent Report
===

Sorted By : Signal
Multiplier : 1.0000
Dilution : 1.0000
Do not use Multiplier & Dilution Factor with ISTDs

Signal 1: VWD1 A, Wavelength=254 nm

Peak #	RetTime [min]	Type	Width [min]	Area [mAU*s]	Height [mAU]	Area %
1	8.391	BB	0.5784	1.35478e4	350.11133	93.9572
2	12.422	BB	1.1469	871.31708	11.19627	6.0428

Totals : 1.44191e4 361.30759

===
*** End of Report ***
